# Catalytic C–F Bond Activation at Room Temperature: Enolate Cross‐Couplings Enabled by [Pd(1‐MeNAP)TFA]_2_ and LiHMDS

**DOI:** 10.1002/anie.9818208

**Published:** 2026-07-26

**Authors:** Nele Krüger, Jie Shen, Sourav Manna, Lamphiza O. Najiar, Lukas J. Gooßen

**Affiliations:** ^1^ Department of Organic Chemistry I Fakultät für Chemie und Biochemie Ruhr‐Universität Bochum Bochum Germany

**Keywords:** catalysis, C–F‐activation, palladium, α‐arylation

## Abstract

Due to their exceptional stability, only few transition metal catalysts are capable of activating C─F bonds for C–C‐coupling reactions, usually under harsh conditions or with highly reactive nucleophiles. We herein report that a methyl naphthyl (MeNAP) palladium trifluoroacetate/*^t^* BuBrettPhos catalyst with lithium bis(trimethylsilyl)amide (LiHMDS) as the base enables α‐arylations of in situ generated enolates with non‐activated aryl fluorides. The reaction proceeds under mild conditions at room temperature and extends to ketone, ester, nitrile, and even amide enolates. DFT calculations reveal that the coordination of lithium salts to the fluorine atom decisively facilitates the insertion of Pd into the C─F bond, explaining why this so far elusive reaction is now possible at such exceptionally mild conditions.

## Graphical abstract

Catalysts generated from the trifluoroacetate‐bridged methylnaphthyl‐palladium dimer [Pd(1‐MeNAP)TFA]_2_ and biarylphosphine ligands in combination with the base LiHMDS promote α‐arylation reactions of various enolates with non‐activated aryl fluorides at room temperature. Computational studies elucidate the role of Li‐dimers in the oxidative addition step.

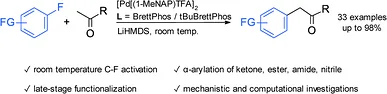

Cross‐coupling reactions between aryl halides and carbon nucleophiles rank among the most widely employed C─C bond‐forming methodologies in both academic research and industrial practice [1, 2]. Over the past decades, advances in cross‐coupling catalysis have enabled a systematic expansion from aryl iodides to the more readily available aryl bromides and aryl chlorides [3]. Extending these transformations to aryl fluorides represents not only a significant conceptual challenge but also an opportunity of considerable practical relevance [4, 5, 6, 7, 8]. Owing to the exceptional inertness of the C─F bond toward most catalytic processes, aryl fluorides can be carried through multistep syntheses, allowing fluorine substituents to persist in highly functionalized intermediates [9]. In principle, the selective late‐stage activation of C─F bonds would enable the diversification of such intermediates through cross‐coupling reactions [10].

Within this broader context, the transition‐metal‐catalyzed α‐arylation of carbonyl compounds has emerged as a particularly powerful and versatile class of cross‐coupling reactions. Since the pioneering work by Semmelhack et al. [11] and through major contributions by Hartwig [12], Buchwald [13], and Miura [14], these transformations have evolved into mature catalytic methods for C─C bond formation. A defining advantage of α‐arylation chemistry is the use of carbon nucleophiles generated in situ by base‐mediated deprotonation of C–H acidic substrates, thereby avoiding the need for preformed, highly reactive organometallic reagents [15]. This progress has been enabled by catalyst systems based on palladium or nickel in combination with electron‐rich, sterically demanding phosphine ligands or *N*‐heterocyclic carbenes, which efficiently promote oxidative addition, enolate transmetalation, and reductive elimination (Scheme 1a) [15, 16, 17, 18, 19, 20, 21, 22, 23, 24, 25, 26, 27]. As a result, modern α‐arylation protocols exhibit broad functional group tolerance and have found widespread application in the synthesis of pharmaceuticals, agrochemicals, and natural products [28, 29, 30].

**SCHEME 1:**
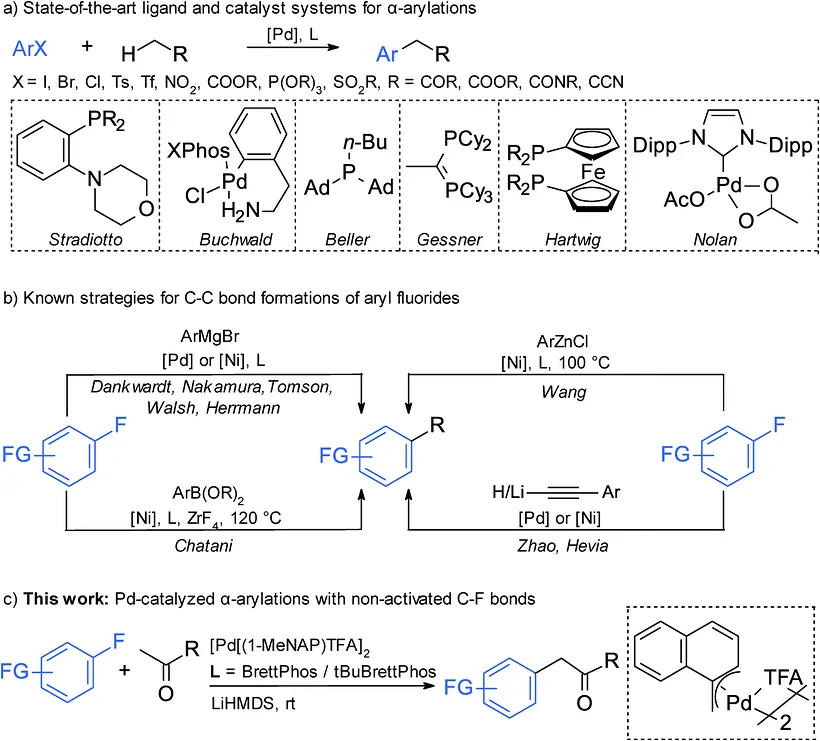
Catalytic C─C bond formations with activation of C─F bonds.

A notable limitation, however, is the inability to engage with aryl fluoride electrophiles. While cross‐couplings of aryl fluorides with highly reactive, preformed organometallic nucleophiles, including organoboron‐ [31, 32, 33, 34, 35], organozinc‐ [36, 37, 38, 39, 40, 41], organotin‐ [35, 42, 43], Grignard reagents [44, 45, 46, 47, 48, 49], and alkynyl salts [50, 51] are known, the coupling of non‐activated aryl fluorides with in situ generated mild carbon nucleophiles, with enolates as a key class, pose a major unresolved challenge (Scheme 1b). To the best of our knowledge, precedents for α‐arylation with aryl fluorides are confined to isolated cases involving activated substrates at temperatures ≥120°C, namely a non‐catalytic S_N_Ar arylation of amides by Chatani [52], and a nickel‐catalyzed arylation of cyclic ketones with 2‐fluoropyridines by Wang [53]. The development of a catalytic α‐arylation of enolates with non‐activated aryl fluorides would establish a fundamentally new reactivity manifold, unlocking new opportunities for the late‐stage modification of functionalized aromatic compounds.

We have recently shown that the efficiency of aryl halide cross‐coupling reactions is markedly enhanced when methylnaphthyl (MeNAP) palladium dimers are employed as precatalysts [54, 55]. Upon treatment with bulky phosphine ligands, these dimers rapidly generate the corresponding monoligated L–Pd(MeNAP)halide complexes. Even in the presence of weak, non‐reducing nucleophiles, fast transmetalation followed by reductive elimination of substituted methylnaphthalenes occurs, resulting in the rapid and efficient formation of catalytically active, monoligated Pd(0) species. The weakly coordinating naphthalene derivatives released during this activation step minimize competitive binding at the metal center, thereby facilitating oxidative addition of aryl electrophiles. Collectively, these features decisively enhance cross‐coupling reactions of aryl chlorides and sometimes even enable their extension to aryl fluorides [56].

Based on these findings, we set out to target the long‐sought cross‐coupling of non‐activated aryl fluorides with enolates. Owing to their high nucleophilicity, enolate salts were anticipated to engage in background reactivity, which was confirmed by the observation of non‐regiospecific nucleophilic aromatic substitution with the enolate and the amide base already at 60°C (Scheme S1). Catalytic screening was therefore conducted at room temperature to maximize selectivity. We used the reaction between *p*‐fluoroanisole and acetophenone as a model system and systematically evaluated a range of catalysts, ligands, bases, and solvents (Tables 1 and S1). As expected, state‐of‐the‐art catalyst systems for enolate couplings of aryl chlorides (e.g., G2‐Pd‐XPhos) were ineffective in promoting this transformation, regardless of whether KO^t^Bu or LiHMDS was used (entries 1 and 2) [20]. A catalyst system based on [Pd(1‐MeNAP)Br]_2_, which we had used in the amination of aryl fluorides at elevated temperatures, was also ineffective (entry 3). However, replacing the palladium precursor with [Pd(1‐MeNAP)TFA]_2_ resulted in a pronounced increase in reactivity, leading to high conversion at room temperature (entry 4). A systematic ligand screening identified *^t^* BuBrettPhos and BrettPhos as the most effective ligands for this transformation (entries 8 and 9). Notably, QPhos, as well as MorDalPhos and CataCXium, that are commonly regarded as ligands of choice for enolate couplings of aryl chlorides proved ineffective under these conditions (Table S1).

**TABLE 1:** Effects of Pd‐precursors, ligands, and bases.^a^

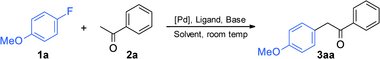					
Entry	Catalyst	Ligand	Base	Solvent	Yield (%)
1^b^	G2‐XPhos	None	KO*^t^* Bu	Toluene	0
2^b^	"	"	LiHMDS	"	0
3	[Pd(1‐MeNAP)Br]_2_	XPhos	"	"	0
4	(Pd(1‐MeNAP)TFA)_2_	"	"	"	58
5	"	*^t^* BuDavePhos	"	"	48
6	"	*^t^* BuXPhos	"	"	85
7	"	AdBrettPhos	"	"	52
8	"	BrettPhos	"	"	>95
9	"	*^t^* BuBrettPhos	"	"	>95
10	"	"	LDA	"	0
11	"	"	LiOMe	"	0
12	"	"	NaHMDS	"	0
13	"	"	KHMDS	"	0
14	"	"	LiHMDS	THF	0
15	"	"	"	*^t^* BuOH	0
16	"	"	"	DCM	0
17	"	"	"	Hexane	0
18	Pd_2_dba_3_	"	"	Toluene	0
19	[Pd(crotyl)Cl]_2_	"	"	"	0
20	[Pd(cinnamyl)Cl]_2_	"	"	"	0
21	[Pd(allyl)Cl]_2_	"	"	"	0

^a^ Conditions: 0.25 mmol **1a**, 1.50 equiv **2a**, 2.0 mol% [Pd], 2.0 mol % ligand, 3.0 equiv base, 2.75 mL solvent, rt, 16 h, yields determined by GC analysis using *n*‐hexadecane as internal standard.

^b^ See literature [20], 1 mol% Pd, 60°C.

The choice of base was found to be critical, with LiHMDS being uniquely effective. Control experiments demonstrated that both the lithium cation and the HMDS anion are essential for productive reactivity (entries 10–13). The reaction was also found to be strongly affected by the solvent, proceeding exclusively in toluene (entries 14–17). Solvents bearing coordinating functional groups, including ethers, amides, esters, nitriles, or alcohols, completely suppressed reactivity (Table S1). The solvent effect is substantially more pronounced than in analogous aryl chloride or bromide couplings, which suggests that oxidative addition of the aryl fluoride may involve a substrate–base–catalyst adduct that is disrupted by competitive coordination from solvent molecules. The fact that non‐functionalized hexane was ineffective as solvent, can be rationalized with the limited solubility of the reaction components.

The choice of catalyst precursor strongly impacts the reaction efficiency. [Pd(1‐MeNAP)TFA]_2_ achieves full conversion within 9 h at room temperature, whereas conventional Pd(II) precatalysts such as allyl‐ or cinnamyl‐based complexes, as well as Pd_2_(dba)_3_, produce no product under the same conditions (Table 1, entries 18–22). We attribute this to the formation of strongly coordinating alkenes during activation of these traditional precatalysts, which likely hinder formation of the catalytically active species. The counterion identity is also critical: [Pd(1‐MeNAP)Br]_2_ shows significant activity only at elevated temperatures. Under fully optimized conditions, several other catalyst precursors, including Pd(OAc)_2_, Hazari's [Pd(^t^Bu‐indenyl)Cl]_2_ precatalyst, and Buchwald ^t^BuBrettPhos‐G3, also give turnover, but only after extended induction periods. MeNAP–TFA precursors stand out for their rapid and reliable activation at room temperature (Figure S1).

Having thus found an effective catalyst system that promotes the previously elusive arylation of enolates with a non‐activated aryl fluoride already at room temperature, we proceeded to evaluate the scope of this transformation (Table 2).

**TABLE 2:** Scope of the coupling of aryl fluorides with enolates.^a^

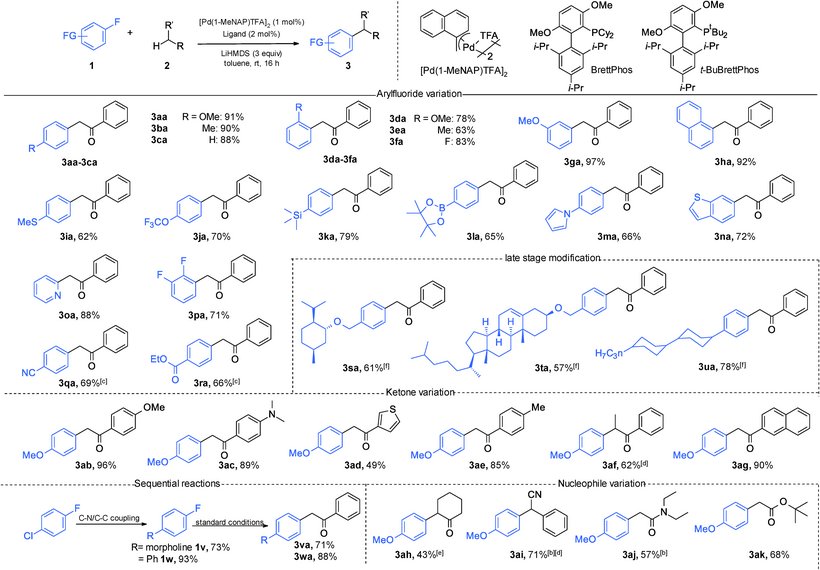

^a^ Conditions: 0.50 mmol **1**, 1.50 equiv **2**, 1.0 mol % [Pd(1‐MeNAP)TFA]_2_, 2.0 mol % BrettPhos, 3.0 equiv LiHMDS, 3.5 mL toluene, rt, 16 h. Isolated yields.

^b^ *^t^* BuBrettPhos as ligand.

^c^ 4.0 equiv LiHMDS.

^d^ 40°C.

^e^ 60°C.

^f^ 0.25 mmol.

The α‐arylation of acetophenone (**2a**) was employed to evaluate the generality of the protocol with respect to aryl fluorides. Both electron‐rich and electron‐deficient arenes participated efficiently, and common functional groups, including methoxy, fluoro, thioether, and trifluoromethoxy substituents, were well tolerated (**3aa**‐**3ja**). Polycyclic and heteroaromatic substrates such as naphthyl, thiophene, and pyridine derivatives likewise proved compatible (**3ha**, **3ma**–**3oa**). Notably, base‐ and nucleophile‐sensitive functionalities, including nitriles and esters (**3qa**–**3ra**), remained intact under the reaction conditions. Functional groups suitable for further derivatization, such as TMS and Bpin moieties (**3ka**–**3la**), were also accommodated. A polyfluorinated substrate (**3pa**) underwent smooth coupling in high yield and with good selectivity. Control experiments established that under such mild conditions even highly activated substrates, such as 2‐fluoropyridine (**3oa**), require the palladium catalyst and do not react via an S_N_Ar pathway. Furthermore, the successful coupling of complex molecules, including menthol‐ and cholesterol‐derived substrates (**3sa‐**‐**3ua**), demonstrates the potential of this methodology for the late‐stage functionalization of fluorinated compounds.

The scope with respect to the enolate coupling partner was examined using the challenging aryl fluoride *p*‐fluoroanisole (**1a**). A broad range of enolates furnished the corresponding coupling products in good yields. α‐Substituted ketones as well as cyclic ketones (**3af**, **3ah**) were efficiently converted. In addition to ketones, esters, amides, and nitriles (**3ai**–**3ak**) also underwent smooth coupling, further underscoring the synthetic utility of the method. Most substrates gave high conversions at room temperature; only the α‐substituted ketone **3af** and the nitrile **3ai** required heating to 40°C, whereas the cyclic ketone **3ah** necessitated a temperature of 60°C.

A sequential coupling of 4‐fluorophenyl chloride illustrates the concept of aryl fluorides as masked arylating agents. While the C─Cl bond undergoes selective cross‐coupling in the first step with the C─F bond remaining inert, the latter can be activated under the present conditions in a subsequent step, enabling installation of an α‐aryl moiety in place of fluorine (Table 2, **3va**–**3wa**). The scalability of the method was demonstrated by performing the synthesis of **3aa** on a 5 mmol scale affording it in 86% (966.8 mg).

Experimental studies were conducted to elucidate the origin of the unique reactivity of the MeNAP precatalyst. The generation of the active Pd(0) species was monitored indirectly by GC analysis of the MeNAP‐derived ketone, which is released upon transmetalation/reductive elimination of the precatalyst with the enolate nucleophile. When mixing *^t^* BuBrettPhos, [Pd(1‐MeNAP)TFA]_2_ and acetophenone, an orange solution formed, which immediately turned purple upon the addition of the LiHMDS. The dependence of the color on the nucleophile suggests that the purple species is a Pd–enolate. Near‐quantitative formation of Pd(0) was observed within 5 min, as evidenced by the formation of the MeNAP‐ketone 4 byproduct (Scheme 2a). No HMDS‐substituted MeNAP was detected, indicating that the enolate is more reactive toward transmetalation.

**SCHEME 2:**
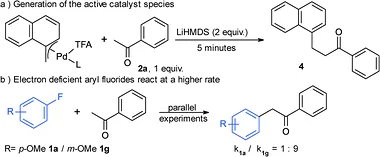
Experimental mechanistic studies.

Kinetic experiments revealed a sigmoidal conversion–time profile with an induction period of approximately 30 min. This was unexpected, as it suggests that the catalyst is not immediately active after reductive activation. We reasoned that the naphthalene moiety of the MeNAP‐derived ketone competes with the aryl fluoride for coordination at the Pd center, thereby inhibiting substrate binding. Consistent with this hypothesis, alkenes such as dba or alkenyl ketones arising from allyl‐ or dba‐derived Pd precursors completely suppressed reactivity, suggesting that these ligands form strongly binding η^2^ complexes, outcompeting the substrate (Figure S2).

Further studies (Scheme 2b) showed that electron‐deficient aryl fluorides react markedly faster than their electron‐rich counterparts, exhibiting a rate of 9:1, indicating that the oxidative addition via C─F bond activation is the limiting step in the actual catalytic cycle.

The full catalytic cycle was evaluated by DFT calculations using ORCA [57] at the B97M‐D4/def2‐SVP level with SMD solvation (toluene), with single‐point energies refined at the def2‐TZVP level and transition states confirmed by IRC. The key findings are summarized in Figure 1, see also Figure S10. As a model, we used the reaction between a simple phenyl fluoride and acetone. The most stable isomer of *^t^* BuBrettPhos MeNAP‐Pd‐TFA (**Int1**) features an η^1^‐coordination of the MeNAP (Figure S5), which explains why the transmetalation with lithium enolate proceeds swiftly with an activation barrier of only 35 kJ/mol. In this and all following steps, we used dimeric lithium species such as LiF‐Li‐acetone enolate as simplified solution models of the lithium reagents. Monomeric lithium species have such a profound tendency to form Li─Li bonds that they are unlikely to be present in solution and would have delivered unrealistically high reactivities (Figure S6) [58, 59].

**FIGURE 1:**
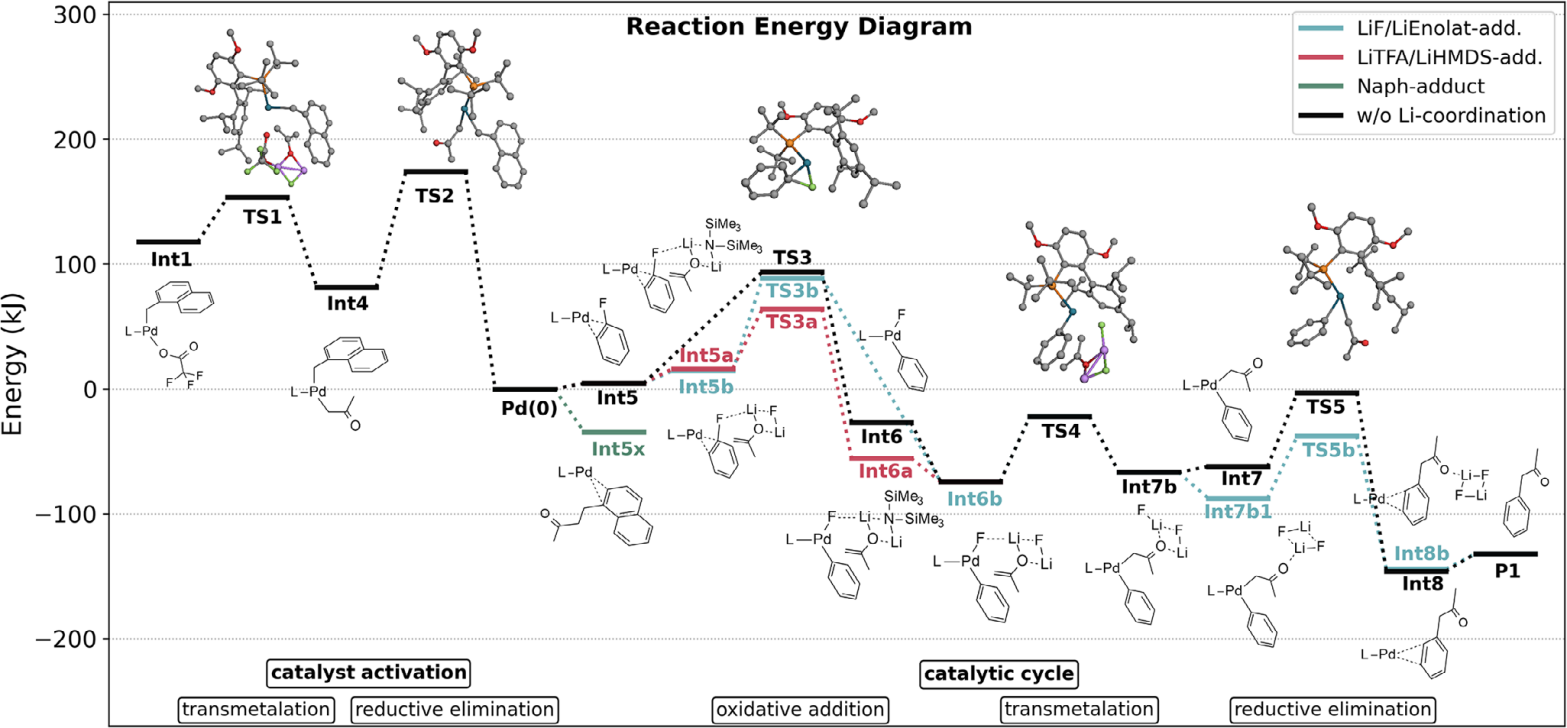
Energy profile diagram (Δ*G* in kJ/mol) for the proposed catalyst activation pathway and subsequent enolate coupling. The oxidative addition step is facilitated by Li dimers (black and red pathways). For detailed computational data, see Figure S10.

The reductive elimination of MeNAP‐acetone (**Int4**→**Pd(0)**) has a considerable barrier of 92 kJ/mol for **TS2**. The IRC corroborates the structural expectation that the naphthalene moiety is unbound and distal to the Pd center after reductive elimination. However, the formation of a L‐Pd(0)/MeNAP‐ketone π‐complex (**Pd(0)**→**Int5x**) is exergonic by −32 kJ/mol. A coordination of the MeNAP‐ketone, while still in the vicinity of the palladium, would explain the experimentally observed induction period beyond Pd(0) formation (see Figure S7).

The actual catalytic cycle starts with the oxidative addition of the aryl fluoride **Int5**→**Int6**. This step has a considerable barrier of 92 kJ for **TS3**, whereas transmetallation **Int6b**→**Int7b** (**TS4**, 53 kJ/mol), de‐coordination of lithium fluoride dimer and reductive elimination (**Int7b**→**Int7**→**Int8**) have a substantially lower barrier (**TS5**, 59 kJ/mol). We next probed whether the coordination of the dilithium species to the fluorine atom would affect the rate‐determining oxidative addition step. The coordination of LiF/LiF, LiF/LiHMDS, LiEnolate/Li‐HMDS, and so forth to the π‐coordinated aryl fluoride turned out to be slightly endergonic (**Int5**→**Int5a**‐**b**, see also Figure S8), but it strongly affected the oxidative addition transition states (**TS3**). Coordination of LiTFA‐LiHMDS lowered the energy span of the overall transformation by as much as 28 kJ/mol, coordination of (LiEnolate)_2_, (LiF)_2_ decreased the reaction barrier only slightly, and LiEnolate‐LiHMDS coordination even increased the overall energy span by 8 kJ/mol (Figure S8). These findings explain why the choice of the base is so critical in this transformation and why the combination of the TFA catalyst with the LiHMDS base is so uniquely effective. The exchange of coordinated dilithium species and their de‐coordination from the ketone products have only moderate barriers and may happen multiple times during a catalytic cycle (Figure S9). To assess the reliability of the chosen computational protocol, we compared one representative reaction step using different functionals, basis sets and solvent models (Figure S11). No significant deviations were observed, and the key finding that lithium coordination lowers the barrier of the rate‐determining oxidative addition step proved to be independent of the computational setup. Nevertheless, the present models involve several simplifications. In particular, all lithium species were treated as dimers, although different aggregation states are likely present in solution, and explicit solvation effects were neglected. Therefore, more elaborate calculations including explicit solvent molecules and more diverse lithium aggregates will be required to fully elucidate the origin of the accelerating effects of lithium, TFA, and HMDS. The mechanistical understanding won in these calculations that the C–F activation, and thereby the entire catalytic reaction, is facilitated by lithium salt coordination allows to understand why this particular reaction proceeds so smoothly already at room temperature. It also explains similar findings, for example, for C–F aminations, and opens up many opportunities for the development of mild C–F functionalizations.

In conclusion, the use of a trifluoroacetate‐bridged methylnaphthyl palladium dimer as precatalyst in combination with bulky bi‐phenyl phosphines as ligands and LiHMDS as the base enables the so far elusive coupling of mild, in situ generated carbon nucleophiles with aryl fluoride substrates. The enabling effect of the MeNAP precatalyst is rationalized with the efficient generation of monoligated palladium complexes and their rapid conversion into catalytically active Pd(0) species. DFT‐calculations suggest that the insertion into the C─F bond is decisively facilitated by the coordination of lithium salts to the fluorine atom.

## Author Contributions

**Nele Krüger**: methodology, investigation, validation, formal analysis, project administration, writing – original draft, writing – review and editing, data curation, visualization. **Jie Shen**: conceptualization, methodology, investigation, validation, formal analysis, project administration, writing – review and editing, data curation, visualization. **Sourav Manna**: methodology, investigation, formal analysis. **Lamphiza O. Najiar**: investigation, formal analysis, writing – review and editing, data curation. **Lukas J. Gooßen**: supervision, project administration, conceptualization, funding acquisition, writing – review and editing, software, resources, methodology, validation, investigation, writing – original draft, visualization, data curation, formal analysis

## Conflicts of Interest

UMICORE AG & Co. KG, who is collaborating with the Gooßen group, has commercialized Pd‐methylnaphthyl catalysts.

## Supporting information




**Supporting File 1**: anie73708‐sup‐0001‐SuppMat.pdf.

**Supporting File 2**: anie73708‐sup‐0002‐data.zip.

## Data Availability

The data that support the findings of this study are openly available in the Sciflection repository (https://identifiers.org/sciflection:2e673a2a‐22df‐450b‐9ed1‐a4fcaaf9f1e4).

## References

[1] C. C. C. Johansson Seechurn , M. O. Kitching , T. J. Colacot , and V. Snieckus , “Palladium‐Catalyzed Cross‐Coupling: A Historical Contextual Perspective to the 2010 Nobel Prize,” Angewandte Chemie International Edition 51 (2012): 5062–5085, 10.1002/anie.201107017. 22573393

[2] M. R. Biscoe , J. Cornella , D. Kalyani , and S. Neufeldt , “From Established to Emerging: Evolution of Cross‐Coupling Reactions,” Journal of Organic Chemistry 89 (2024): 16065–16069, 10.1021/acs.joc.4c02573. 39545380

[3] A. F. Littke and G. C. Fu , “Palladium‐Catalyzed Coupling Reactions of Aryl Chlorides,” Angewandte Chemie International Edition 41 (2002): 4176–4211, 10.1002/1521-3773(20021115)41:22<4176::AID-ANIE4176>3.0.CO;2-U. 12434342

[4] J.‐D. Hamel and J.‐F. Paquin , “Activation of C─F Bonds α to C–C Multiple Bonds,” Chemical Communications 54 (2018): 10224–10239, 10.1039/c8cc05108a. 30124701

[5] T. Ahrens , J. Kohlmann , M. Ahrens , and T. Braun , “Functionalization of Fluorinated Molecules by Transition‐Metal‐Mediated C–F Bond Activation to Access Fluorinated Building Blocks,” Chemical Reviews 115 (2015): 931–972, 10.1021/cr500257c. 25347593

[6] J. L. Kiplinger , T. G. Richmond , and C. E. Osterberg , “Activation of Carbon‐Fluorine Bonds by Metal Complexes,” Chemical Reviews 94 (1994): 373–431, 10.1021/cr00026a005.

[7] E. Clot , O. Eisenstein , N. Jasim , S. A. Macgregor , J. E. McGrady , and R. N. Perutz , “C−F and C−H Bond Activation of Fluorobenzenes and Fluoropyridines at Transition Metal Centers: How Fluorine Tips the Scales,” Accounts of Chemical Research 44 (2011): 333–348, 10.1021/ar100136x. 21410234

[8] H. Amii and K. Uneyama , “C−F Bond Activation in Organic Synthesis,” Chemical Reviews 109 (2009): 2119–2183, 10.1021/cr800388c. 19331346

[9] A. Cerveri , G. Russo , S. Sparascio , et al., “Late‐Stage Functionalization Using a Popular Titrating Agent: Aryl‐Chlorides and ‐Fluorides Activation by the Diphenylacetic Acid Dianion,” Chemistry: An European Journal 31 (2025): e202403597, 10.1002/chem.202403597. 39462191

[10] J. Börgel and T. Ritter , “Late‐Stage Functionalization,” Chemistry 6 (2020): 1877–1887, 10.1016/j.chempr.2020.07.007.

[11] M. F. Semmelhack and T. M. Bargar , “Cyclizations of Enolates Onto Aromatic Rings Via the Photo‐SRN1 Reaction. Preparative and Mechanistic Aspects,” Journal of Organic Chemistry 42 (1977): 1481–1482, 10.1021/jo00428a056.

[12] B. C. Hamann and J. F. Hartwig , “Palladium‐Catalyzed Direct α‐Arylation of Ketones. Rate Acceleration by Sterically Hindered Chelating Ligands and Reductive Elimination From a Transition Metal Enolate Complex,” Journal of the American Chemical Society 119 (1997): 12382–12383, 10.1021/ja9727880.

[13] M. Palucki and S. L. Buchwald , “Palladium‐Catalyzed α‐Arylation of Ketones,” Journal of the American Chemical Society 119 (1997): 11108–11109, 10.1021/ja972593s.

[14] T. Satoh , Y. Kawamura , M. Miura , and M. Nomura , “Palladium‐Catalyzed Regioselective Mono‐ and Diarylation Reactions of 2‐Phenylphenols and Naphthols With Aryl Halides,” Angewandte Chemie International Edition 36 (1997): 1740–1742, 10.1002/anie.199717401.

[15] D. A. Culkin and J. F. Hartwig , “Palladium‐Catalyzed α‐Arylation of Carbonyl Compounds and Nitriles,” Accounts of Chemical Research 36 (2003): 234–245, 10.1021/ar0201106. 12693921

[16] A. Ehrentraut , A. Zapf , and M. Beller , “Progress in the Palladium‐Catalyzed α‐Arylation of Ketones With Chloroarenes,” Advanced Synthesis & Catalysis 344 (2002): 209, 10.1002/1615-4169(200202)344:2<209::AID-ADSC209>3.0.CO;2-5.

[17] K. D. Hesp , R. J. Lundgren , and M. Stradiotto , “Palladium‐Catalyzed Mono‐α‐Arylation of Acetone With Aryl Halides and Tosylates,” Journal of the American Chemical Society 133 (2011): 5194–5197, 10.1021/ja200009c. 21417401

[18] X. Chen , Z. Chen , and C. M. So , “Exploration of Aryl Phosphates in Palladium‐Catalyzed Mono‐α‐Arylation of Aryl and Heteroaryl Ketones,” Journal of Organic Chemistry 84 (2019): 6337–6346, 10.1021/acs.joc.9b00669. 31045364

[19] R. Martín and S. L. Buchwald , “A General Method for the Direct α‐Arylation of Aldehydes With Aryl Bromides and Chlorides,” Angewandte Chemie International Edition 46 (2007): 7236–7239, 10.1002/anie.200703009. 17722128

[20] M. R. Biscoe and S. L. Buchwald , “Selective Monoarylation of Acetate Esters and Aryl Methyl Ketones Using Aryl Chlorides,” Organic Letters 11 (2009): 1773–1775, 10.1021/ol900295u. 19296693 PMC2747740

[21] Z. Li , Y. Peng , and T. Wu , “Palladium‐Catalyzed Denitrative α‐Arylation of Ketones With Nitroarenes,” Organic Letters 23 (2021): 881–885, 10.1021/acs.orglett.0c04104. 33492977

[22] P. M. MacQueen , A. J. Chisholm , B. K. V. Hargreaves , and M. Stradiotto , “Palladium‐Catalyzed Mono‐α‐Arylation of Acetone at Room Temperature,” Chemistry: An European Journal 21 (2015): 11006–11009, 10.1002/chem.201500834. 26119558

[23] X.‐Q. Hu , D. Lichte , I. Rodstein , et al., “Ylide‐Functionalized Phosphine (YPhos)–Palladium Catalysts: Selective Monoarylation of Alkyl Ketones With Aryl Chlorides,” Organic Letters 21 (2019): 7558–7562, 10.1021/acs.orglett.9b02830. 31469570

[24] A. J. DeAngelis , P. G. Gildner , R. Chow , and T. J. Colacot , “Generating Active “L‐Pd(0)” Via Neutral or Cationic π‐Allylpalladium Complexes Featuring Biaryl/Bipyrazolylphosphines: Synthetic, Mechanistic, and Structure–Activity Studies in Challenging Cross‐Coupling Reactions,” Journal of Organic Chemistry 80 (2015): 6794–6813, 10.1021/acs.joc.5b01005. 26035637

[25] R. Singh and S. P. Nolan , “An Efficient and Mild Protocol for the α‐Arylation of Ketones Mediated by an (Imidazol‐2‐ylidene)palladium(acetate) System,” Journal of Organometallic Chemistry 690 (2005): 5832–5840, 10.1016/j.jorganchem.2005.07.083.

[26] E. Marelli , Y. Renault , S. V. Sharma , S. P. Nolan , and R. J. M. Goss , “Mild, Aqueous α‐Arylation of Ketones: Towards New Diversification Tools for Halogenated Metabolites and Drug Molecules,” Chemistry: An European Journal 23 (2017): 3832–3836, 10.1002/chem.201700680. 28195381

[27] M. S. Viciu , R. F. Germaneau , and S. P. Nolan , “Well‐Defined, Air‐Stable (NHC)Pd(Allyl)Cl (NHC = N‐Heterocyclic Carbene) Catalysts for the Arylation of Ketones,” Organic Letters 4 (2002): 4053–4056, 10.1021/ol026745m. 12423084

[28] K. J. Swathy , P. V. Saranya , and G. Anilkumar , “Recent Advances in Palladium‐Catalyzed α‐Arylation Reactions,” Applied Organometallic Chemistry 38 (2024): e7508, 10.1002/aoc.7508.

[29] Y.‐J. Hao , X.‐S. Hu , Y. Zhou , J. Zhou , and J.‐S. Yu , “Catalytic Enantioselective α‐Arylation of Carbonyl Enolates and Related Compounds,” ACS Catalysis 10 (2020): 955–993, 10.1021/acscatal.9b04480.

[30] Y. Xu , T. Su , Z. Huang , and G. Dong , “Practical Direct α‐Arylation of Cyclopentanones by Palladium/Enamine Cooperative Catalysis,” Angewandte Chemie International Edition 55 (2016): 2559–2563, 10.1002/anie.201510638. 26840218

[31] M. R. Cargill , G. Sandford , A. J. Tadeusiak , et al., “Palladium‐Catalyzed C−F Activation of Polyfluoronitrobenzene Derivatives in Suzuki−Miyaura Coupling Reactions,” Journal of Organic Chemistry 75 (2010): 5860–5866, 10.1021/jo100877j. 20704342

[32] M. Tobisu , T. Xu , T. Shimasaki , and N. Chatani , “Nickel‐Catalyzed Suzuki–Miyaura Reaction of Aryl Fluorides,” Journal of the American Chemical Society 133 (2011): 19505–19511, 10.1021/ja207759e. 22023167

[33] T. Schaub , M. Backes , and U. Radius , “Catalytic C−C Bond Formation Accomplished by Selective C−F Activation of Perfluorinated Arenes,” Journal of the American Chemical Society 128 (2006): 15964–15965, 10.1021/ja064068b. 17165711

[34] Z.‐J. Luo , H.‐Y. Zhao , and X. Zhang , “Highly Selective Pd‐Catalyzed Direct C–F Bond Arylation of Polyfluoroarenes,” Organic Letters 20 (2018): 2543–2546, 10.1021/acs.orglett.8b00692. 29667829

[35] Y. M. Kim and S. Yu , “Palladium(0)‐Catalyzed Amination, Stille Coupling, and Suzuki Coupling of Electron‐Deficient Aryl Fluorides,” Journal of the American Chemical Society 125 (2003): 1696–1697, 10.1021/ja028966t. 12580584

[36] D. Wu and Z.‐X. Wang , “P,N,N‐Pincer Nickel‐Catalyzed Cross‐Coupling of Aryl Fluorides and Chlorides,” Organic & Biomolecular Chemistry 12 (2014): 6414, 10.1039/C4OB01041H. 25012049

[37] T. Wang , B. J. Alfonso , and J. A. Love , “Platinum(II)‐Catalyzed Cross‐Coupling of Polyfluoroaryl Imines,” Organic Letters 9 (2007): 5629–5631, 10.1021/ol702591b. 18047367

[38] Y. Nakamura , N. Yoshikai , L. Ilies , and E. Nakamura , “Nickel‐Catalyzed Monosubstitution of Polyfluoroarenes With Organozinc Reagents Using Alkoxydiphosphine Ligand,” Organic Letters 14 (2012): 3316–3319, 10.1021/ol301195x. 22691135

[39] A. D. Sun , K. Leung , A. D. Restivo , N. A. LaBerge , H. Takasaki , and J. A. Love , “Nickel‐Catalyzed Csp^2^─Csp^3^ Bond Formation by Carbon─Fluorine Activation,” Chemistry: An European Journal 20 (2014): 3162–3168, 10.1002/chem.201303809. 24522982

[40] S.‐H. Xiao , Y. Xiong , X.‐X. Zhang , and S. Cao , “Nickel‐Catalyzed N‐Heterocycle‐Directed Cross‐Coupling of Fluorinated Arenes With Organozinc Reagents,” Tetrahedron 70 (2014): 4405–4411, 10.1016/j.tet.2014.04.052.

[41] M. Ohashi , R. Doi , and S. Ogoshi , “Palladium‐Catalyzed Coupling Reaction of Perfluoroarenes With Diarylzinc Compounds,” Chemistry: An European Journal 20 (2014): 2040–2048, 10.1002/chem.201303451. 24431191

[42] T. Braun , R. N. Perutz , and M. I. Sladek , “Catalytic C–F Activation of Polyfluorinated Pyridines by Nickel‐Mediated Cross‐Coupling Reactions,” Chemical Communications 21 (2001): 2254–2255, 10.1039/b106646c. 12240137

[43] S. Bahmanyar , B. C. Borer , Y. M. Kim , D. M. Kurtz , and S. Yu , “Proximity Effects in the Palladium‐Catalyzed Substitution of Aryl Fluorides,” Organic Letters 7 (2005): 1011–1014, 10.1021/ol047413f. 15760126

[44] J. W. Dankwardt , “Transition Metal Catalyzed Cross‐Coupling of Aryl Grignard Reagents With Aryl Fluorides Via Pd‐ or Ni‐Activation of the C–F Bond: An Efficient Synthesis of Unsymmetrical Biaryls—Application of Microwave Technology in Ligand and Catalyst Screening,” Journal of Organometallic Chemistry 690 (2005): 932–938, 10.1016/j.jorganchem.2004.10.037.

[45] N. Yoshikai , H. Mashima , and E. Nakamura , “Nickel‐Catalyzed Cross‐Coupling Reaction of Aryl Fluorides and Chlorides With Grignard Reagents Under Nickel/Magnesium Bimetallic Cooperation,” Journal of the American Chemical Society 127 (2005): 17978–17979, 10.1021/ja056327n. 16366529

[46] K. Manabe and S. Ishikawa , “Ortho‐Selective Cross‐Coupling of Fluorobenzenes With Grignard Reagents: Acceleration by Electron‐Donating Ortho‐Directing Groups,” Synthesis 2008 (2008): 2645–2649, 10.1055/s-2008-1067182.

[47] C. Wu , S. P. McCollom , Z. Zheng , et al., “Aryl Fluoride Activation Through Palladium–Magnesium Bimetallic Cooperation: A Mechanistic and Computational Study,” ACS Catalysis 10 (2020): 7934–7944, 10.1021/acscatal.0c01301. 34221636 PMC8248268

[48] V. P. W. Böhm , C. W. K. Gstöttmayr , T. Weskamp , and W. A. Herrmann , “Catalytic C−C Bond Formation Through Selective Activation of C−F Bonds,” Angewandte Chemie International Edition 40 (2001): 3387–3389, 10.1002/1521-3773(20010917)40:18<3387::AID-ANIE3387>3.0.CO;2-6. 11592146

[49] J. Wei , K.‐M. Liu , and X.‐F. Duan , “Cobalt‐Catalyzed Biaryl Couplings via C–F Bond Activation in the Absence of Phosphine or NHC Ligands,” Journal of Organic Chemistry 82 (2017): 1291–1300, 10.1021/acs.joc.6b02354. 27778507

[50] J. He , K. Yang , J. Zhao , and S. Cao , “LiHMDS‐Promoted Palladium‐Catalyzed Sonogashira Cross‐Coupling of Aryl Fluorides With Terminal Alkynes,” Organic Letters 21 (2019): 9714–9718, 10.1021/acs.orglett.9b03815. 31724867

[51] A. M. Borys , L. Vedani , and E. Hevia , “Stoichiometric and Catalytic Lithium Nickelate‐Mediated C–F Bond Alkynylation of Fluoroarenes,” Journal of the American Chemical Society 146 (2024): 10199–10205, 10.1021/jacs.4c02606. 38545862

[52] A. Matsuura , Y. Ano , and N. Chatani , “Nucleophilic Aromatic Substitution of Non‐Activated Aryl Fluorides With Aliphatic Amides,” Chemical Communications 58 (2022): 9898–9901, 10.1039/D2CC02999E. 35975693

[53] X. Gu , K. Liu , L. Yang , C. Xie , M. Li , and J. J. Wang , “Nickel‐Catalyzed Enantioselective α‐Heteroarylation of Ketones Via C–F Bond Activation to Construct All‐Carbon Quaternary Stereocenters,” Chemical Science 13 (2022): 12498–12502, 10.1039/d2sc03409c. 36382277 PMC9629005

[54] N. Sivendran , N. Pirkl , Z. Hu , A. Doppiu , and L. J. Gooßen , “Halogen‐Bridged Methylnaphthyl Palladium Dimers as Versatile Catalyst Precursors in Coupling Reactions,” Angewandte Chemie International Edition 60 (2021): 25151–25160, 10.1002/anie.202110450. 34520603 PMC9293455

[55] S. Manna , H. F. Janning , N. V. Tzouras , et al., “Unlocking Catalyst Activation as a Critical Bottleneck in Cross‐Coupling Reactions: Room‐Temperature Couplings of Weak Nucleophiles Enabled by [Pd(1‐MeNAP)TFA]_2_ Precatalysts,” Angewandte Chemie International Edition 65 (2026): e3359287, 10.1002/anie.3359287. 42126509 PMC13327566

[56] J. Shen , J. Stemmer , S. Manna , N. V. Tzouras , and L. J. Gooßen , “Amination and Para ─C─H Arylation of Aryl Fluorides Enabled by α‐Methylnaphthyl (MeNAP) Palladium Catalysts,” Angewandte Chemie International Edition 65 (2026): e21597, 10.1002/anie.202521597. 41277082

[57] F. Neese , “Software Update: The ORCA Program System—Version 6.0,” WIREs Computational Molecular Science 15 (2025): e70019, 10.1002/wcms.70019.

[58] H. J. Reich , “Role of Organolithium Aggregates and Mixed Aggregates in Organolithium Mechanisms,” Chemical Reviews 113 (2013): 7130–7178, 10.1021/cr400187u. 23941648

[59] B. L. Lucht and D. B. Collum , “Lithium Ion Solvation: Amine and Unsaturated Hydrocarbon Solvates of Lithium Hexamethyldisilazide (LiHMDS),” Journal of the American Chemical Society 118 (1996): 2217–2225, 10.1021/ja953029p.

