# Supplementary material for: Catalytic C–F Bond Activation at Room Temperature: Enolate Cross‐Couplings Enabled by [Pd(1‐MeNAP)TFA]2 and LiHMDS
Source: Angew Chem Int Ed Engl. 2026 Jul 26;65(38):e9818208. doi: 10.1002/anie.9818208 (PMC13573135; doi:10.1002/anie.9818208)
Supplement: Supplementary file 1 [file ANIE-65-e9818208-s001.pdf]

Supporting Information
©Wiley-VCH 2021
69451 Weinheim, Germany

Catalytic C–F Bond Activation at Room Temperature: Enolate Cross-Couplings Enabled by [Pd(1-MeNAP)TFA]2 and LiHMDS

Nele Krüger†[a], Jie Shen†[a], Sourav Manna,[a] Lamphiza O Najiar,[a] and Lukas J. Gooßen*[a]

*Department of Chemistry and Biochemistry, Ruhr-Universität Bochum, NC 2/170, Universitätsstr. 150, 44801 Bochum, Germany.

*E-mail: lukas.goossen@rub.de

Abstract: Due to their exceptional stability, only few transition metal catalysts are capable of activating C–F bonds for C–C-coupling reactions, usually under harsh conditions or with highly reactive nucleophiles. We herein report that a methyl naphthyl (MeNAP) palladium trifluoroacetate / tBuBrettPhos catalyst with lithium bis(trimethylsilyl)amide (LiHMDS) as the base enables α-arylations of in situ generated enolates with non-activated aryl fluorides. The reaction proceeds under mild conditions at room temperature and extends to ketone, ester, nitrile and even amide enolates. DFT calculations reveal that the coordination of lithium salts to the fluorine atom decisively facilitates the insertion of Pd into the C-F bond, explaining why this so far elusive reaction is now possible at such exceptionally mild conditions.

Table of Contents

General Methods.......2
General Procedures.......3
General procedure A for the C−F arylation of acetophenone (scope).......3
General procedure B.......3
General procedure C Screening.......3
Procedure D: Synthesis of aryl fluoride derivatives for late-stage application.......4
Additional Screening Results.......5
Mechanistic Investigations.......8
Computational Studies.......10
Validation of the level of theory.......14
Synthesis and Characterization of Products.......16
NMR-Spectra of Products.......27
References.......74

General Methods

All reactions were performed in oven-dried 20 mL headspace vials containing a Teflon-coated stirring bar that were closed and crimped with aluminum caps fitted with a Teflon-coated butyl rubber septum under nitrogen atmosphere. The vials were heated to the given temperature in an aluminum block fitted to the hot plate of a laboratory stirrer. Optimization reactions were monitored by GC analysis using n-hexadecane as internal standard. ^{1}H and ^{13}C NMR spectra were recorded on a Avance-Neo-400 spectrometer at 25 °C if not stated otherwise. ^{19}F NMR spectra were recorded on a Avance-Neo-400 spectrometer and a Spinsolve Benchtop NMR (MAGRITEK) spectrometers at 25 °C. All values of the chemical shift are in ppm regarding the *δ*-scale. To display multiplicities and signal forms correctly the following abbreviations were used: s = singlet, d = doublet, t=triplet, q = quartet, spt = septet, m = multiplet, dd = doublet of doublet, dt = doublet of triplet, dq = doublet of quartet, ddd = doublet of doublet of doublet, tt = triplet of triplet, br = broad signal. GC analyses were carried out using an HP-5 capillary column (Phenyl methyl siloxane, 30 m × 320 × 0.25, 100/2.3-30-300/3, 2 min at 60 °C, heating rate 30 °C/min, 3 or 10 min at 300 °C). Column chromatography was performed on a Reveleris® X2 or a Pure C-815 (BÜCHI) Flash Chromatography System. Mass spectrometric data were acquired on a GC-MS Agilent 5977B MSD. HRMS analyses were acquired using a GC-MS system consisting of an Agilent 7250 GC/Q-TOF, in which ionization was achieved by EI, or an HPLC-MS system consisting of an UltiMate 3000 HPLC (ThermoFisher) with an EC 4/2 Universal RP pre-column (Macherey-Nagel), eluting at 40 °C with acetonitrile/water/formic acid (3:1:0.01) and a flow rate of 0.3 mL/min, coupled with a maXisCompact high-resolution mass spectrometer (Bruker Daltonik GmbH). The MS ionization was achieved by ESI. Infrared spectra were recorded on Bruker Vertex 70 Spectrometer with Universal ATR Sampling Accessory. Melting points were measured on a METTLER Toledo MP70. Commercial substrates were used as received unless otherwise stated. Solvents were purchased (puriss p.A.) from commercial suppliers and purified following standard literature techniques[1] and stored over 3Å or 4Å molecular sieves in Schlenk bottles under nitrogen or argon. All solvents were degassed by argon purge prior to use.

General Procedures

General procedure A for the C−F arylation of acetophenone (scope)

An oven-dried vial (20 mL) was charged with $[Pd(MeNAP)TFA]_2$ (0.01 mmol, 3.61 mg, 1 mol%), and BrettPhos (0.02 mmol, 5.53 mg, 2 mol%) or ^{t}BuBrettPhos (0.02 mmol, 4.95 mg, 2 mol%). The vial was purged in three alternating vacuum/nitrogen cycles and 1 mL of toluene was added and the mixture was pre-stirred for 5 minutes. 1 mL of a stock solution of the aryl fluoride (0.5 mmol, 1.0 equiv.), the nucleophile (0.75 mmol, 1.5 equiv.) and internal standard in toluene, was added. Lithium bis(trimethylsilyl)amide 1M in toluene (1.5 mmol, 1.5 mL, 3.0 equiv.) was added, and the resulting mixture was stirred at 30 °C (unless stated otherwise) for 16 h. After completion of the reaction, the mixture was quenched with NH_4Cl, diluted with EtOAc and washed with $NaHCO_3$, water and brine. The combined organic phase was dried over $MgSO_4$ and purified by flash column chromatography (SiO_2, Cy/EtOAc) to yield the product.

General procedure B

An oven-dried vial (20 mL) was charged with $[Pd(MeNAP)TFA]_2$ (0.01 mmol, 3.61 mg, 1 mol%), and BrettPhos (0.02 mmol, 5.53 mg, 2 mol%) or ^{t}BuBrettPhos (0.02 mmol, 4.95 mg, 2 mol%). The vial was purged in three alternating vacuum/nitrogen cycles and 1 mL of toluene was added and the mixture was pre-stirred for 5 minutes. 1 mL of a stock solution of the aryl fluoride (0.5 mmol, 1.0 equiv.) and internal standard in toluene, was added. Lithium bis(trimethylsilyl)amide 1M in toluene (1.5 mmol, 1.5 mL, 4.0 equiv.) was added, and finally the nucleophile (0.75 mmol, 1.5 equiv.). The resulting mixture was stirred at 30 °C for 16 h. After completion of the reaction, the mixture was quenched with NH_4Cl, diluted with EtOAc and washed with $NaHCO_3$, water and brine. The combined organic phase was dried over $MgSO_4$ and purified by flash column chromatography (SiO_2, Cy/EtOAc) to yield the product.

General procedure C Screening

An oven-dried vial (20 mL) was charged with the catalyst (0.02 mmol, 2 mol%), and ligand (0.02 mmol, 2 mol%). The vial was purged in three alternating vacuum/nitrogen cycles and 1 mL of toluene was added and the mixture was pre-stirred for 5 minutes. 1 mL of a stock solution of the 4-fluoroanisole (0.25 mmol, 28.4 µl, 1.0 equiv.), as well as the acetophenone (0.375 mmol, 44.7 µL 1.5 equiv.) and internal standard in toluene were added. The base (0.75 mmol, 3.0 equiv.) was added, and the resulting mixture was stirred at 30 °C for 16 h. After completion of the reaction, the mixture was quenched with NH_4Cl, diluted with EtOAc and washed with $NaHCO_3$, and extracted with EtOAc. The product formation was analyzed by gas chromatography using *n*-hexadecane as an internal standard.

Procedure D: Synthesis of aryl fluoride derivatives for late-stage application

Adapted from literature-reported procedure:[2] To the bioactive compound (1 equiv.) was added dry THF and sodium hydride (1.2 equiv.) at 0 °C. The mixture was stirred at 0 °C for 30 minutes. After that time, 4-fluorobenzyl bromide (1.5 equiv.) was added and the mixture was stirred overnight while warming to room temperature. After completion of the reaction, the mixture was quenched with NH_4Cl, diluted with EtOAc and washed with $NaHCO_3$, water and brine. The combined organic phase was dried over $MgSO_4$ and purified by flash column chromatography (SiO_2, Cy/EtOAc 10 %).

Gram-Scale reaction

An oven-dried vial (100 mL) was charged with $[Pd(MeNAP)TFA]_2$ (0.1 mmol, 36.1 mg, 1 mol%), and BrettPhos (0.2 mmol, 55.3 mg, 2 mol%) The vial was purged in three alternating vacuum/nitrogen cycles and 10 mL of toluene was added and the mixture was pre-stirred for 5 minutes. The aryl fluoride (5 mmol, 1.0 equiv., 0.568 mL), acetophenone (7.5 mmol, 1.5 equiv., 0.894 mL) was added. Lithium bis(trimethylsilyl)amide 1M in toluene (15 mmol, 15 mL, 3.0 equiv.) was added, and the resulting mixture was stirred at 30 °C for 16 h. After completion of the reaction, the mixture was quenched with NH_4Cl, diluted with EtOAc and washed with $NaHCO_3$, water and brine. The combined organic phase was dried over $MgSO_4$ and purified by flash column chromatography (SiO_2, Cy/EtOAc) to yield the product **3aa** in 86 % yield (966.8 mg).

Additional Screening Results

Table S1. Screening Table of the *α*-arylation of aryl fluoride

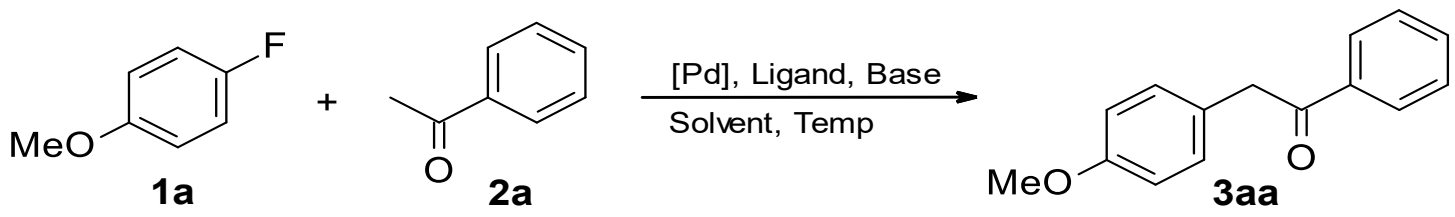

entry	[Pd]	Ligand	Base	Solv.	T (°C)	Yield **[%]**
1	[Pd(1-MeNAP)TFA]$_2$	Catacxium	LiHMDS	Toluene	30	0
2	"	MorDalPhos	"	"	"	0
3	"	QPhos	"	"	"	0
4	"	XantPhos	"	"	"	0
5	"	NiXantPhos	"	"	"	0
6	"	^{t}Bu$_3$P * HBF$_4$	"	"	"	0
7	"	CMPhos	"	"	"	0
8	"	AdBrettPhos	"	"	"	52
9	"	AdBippyPhos	"	"	"	0
10	"	^{t}BuXPhos	"	"	"	85
11	"	SPhos	"	"	"	0
12	"	RuPhos	"	"	"	9
13	"	^{t}BuDavePhos	"	"	"	48
14	"	JackiePhos	"	"	"	0
15	"	BippyPhos	"	"	"	0
16	"	RockPhos	"	"	"	94
17	"	MePhos	"	"	"	0
18	"	^{t}BuBrettPhos	"	MeCN	"	0
19	"	"	"	Dioxane	"	0
20	"	"	"	EtOH	"	0
21	"	"	"	DMF	"	0
22	"	"	LiHMDS (2 eq.)	Toluene	"	18
23	"	"	LiHMDS (1.5 eq.)	"	"	0
24	"	"	LiOTMS	"	"	0
25	"	"	*n*-BuLi	"	"	0
26	"	"	NaO^{t}Bu	"	"	0
27	"	"	K_2CO_3	"	"	0
28	"	"	Cs_2CO_3	"	"	0
29	"	"	K_3PO_4	"	"	0
30	[Pd(MeNAP)TFA]$_2$ (1 mol% Pd)	^{t}BuBrettPhos	LiHMDS	"	"	72
31	[Pd(MeNAP)TFA]$_2$ (0.5 mol% Pd)	"	"	"	"	0

Reaction conditions: [a] 0.25 mmol **1a**, 1.50 equiv. **2a**, 2 mol% [Pd], 2.0 mol % Ligand, 3.0 equiv. base, 2.75 mL solvent, rt, 16 h, yields determined by GC analysis using *n*-hexadecane as internal standard.

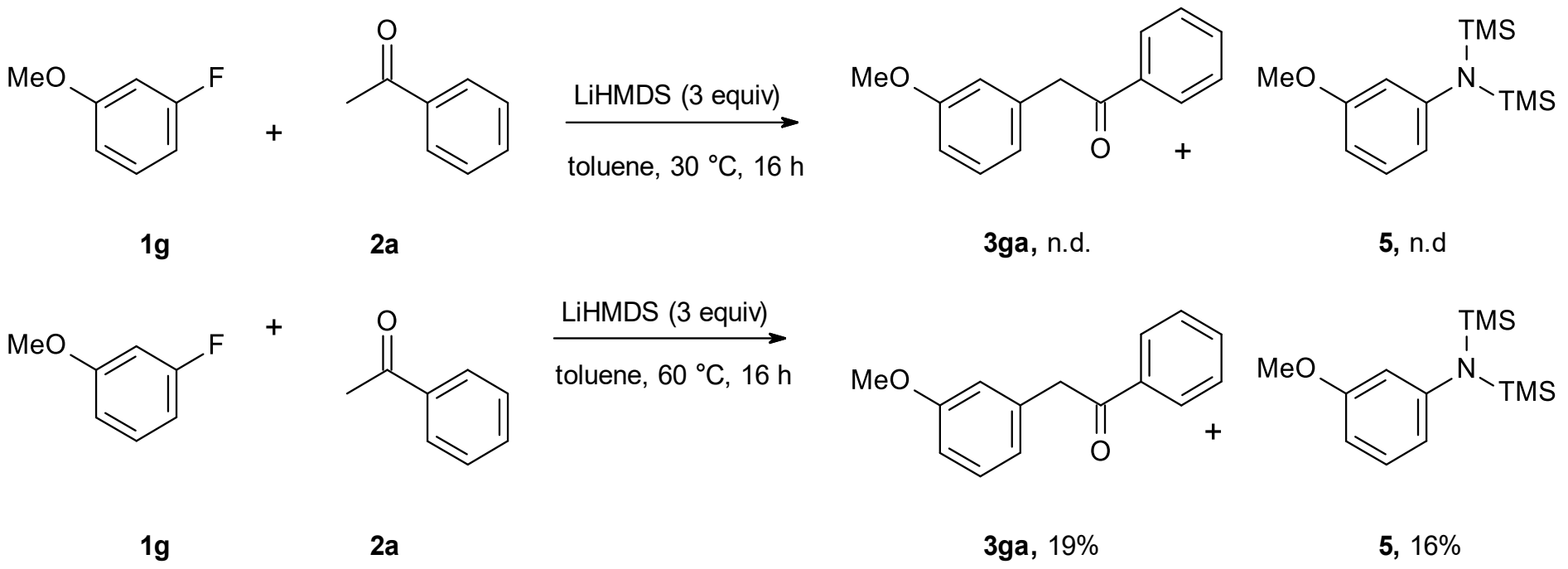

Scheme S1. Control experiments: temperature-dependent background reactivity

The reaction of 3-methoxyfluorobenzene **1g** with acetophenone **2a** was carried out in the absence of Pd catalyst and ligand using LiHMDS (3 equiv.) in toluene at 30 and 60 °C. At 30 °C, no S_NAr products formation were observed. At 60 °C, formation of **3ga** (19%) and the silylated aniline **5** (16%) was detected. These results show that no background S_NAr pathway is operative at 30 °C, while side reactions occur at elevated temperature.

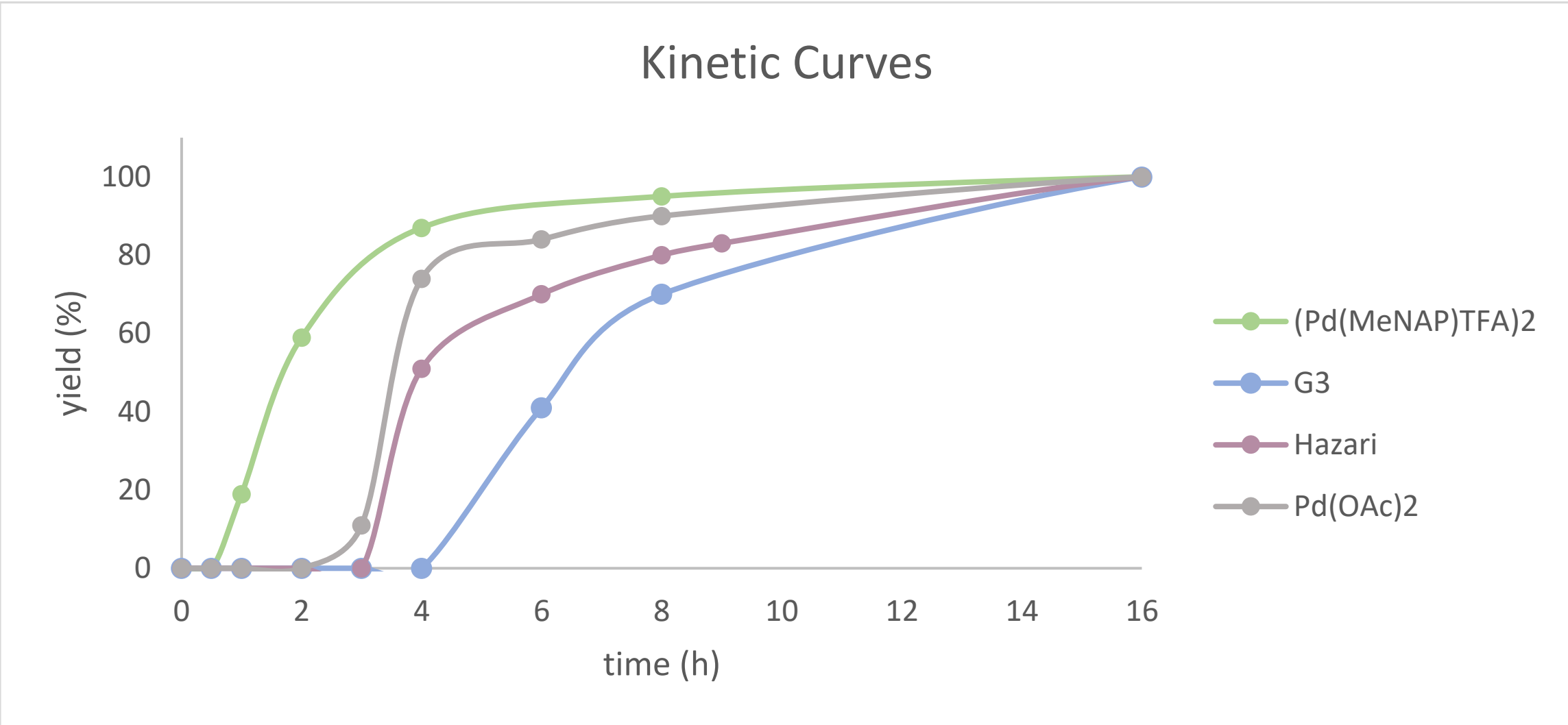

Figure S1. Kinetic comparison of $[Pd(1\text{-}MeNAP)TFA]_2$, Buchwald ^{t}BuBrettPhos G3, $[Pd(^t\text{Bu-indenyl})Cl]_2$, and $Pd(OAc)_2$.

Kinetic profiles obtained with different palladium precursors reveal a pronounced dependence of the reaction rate on the precursor structure. The $(Pd(MeNAP)TFA)_2$ system exhibits a significantly faster initial rate and reaches high conversion within a short time, whereas G3, Hazari, and $Pd(OAc)_2$ show slower reaction profiles with an increased induction period. These results highlight the critical role of the palladium precursor in controlling catalyst activation and overall reaction efficiency (Figure S1).[3]

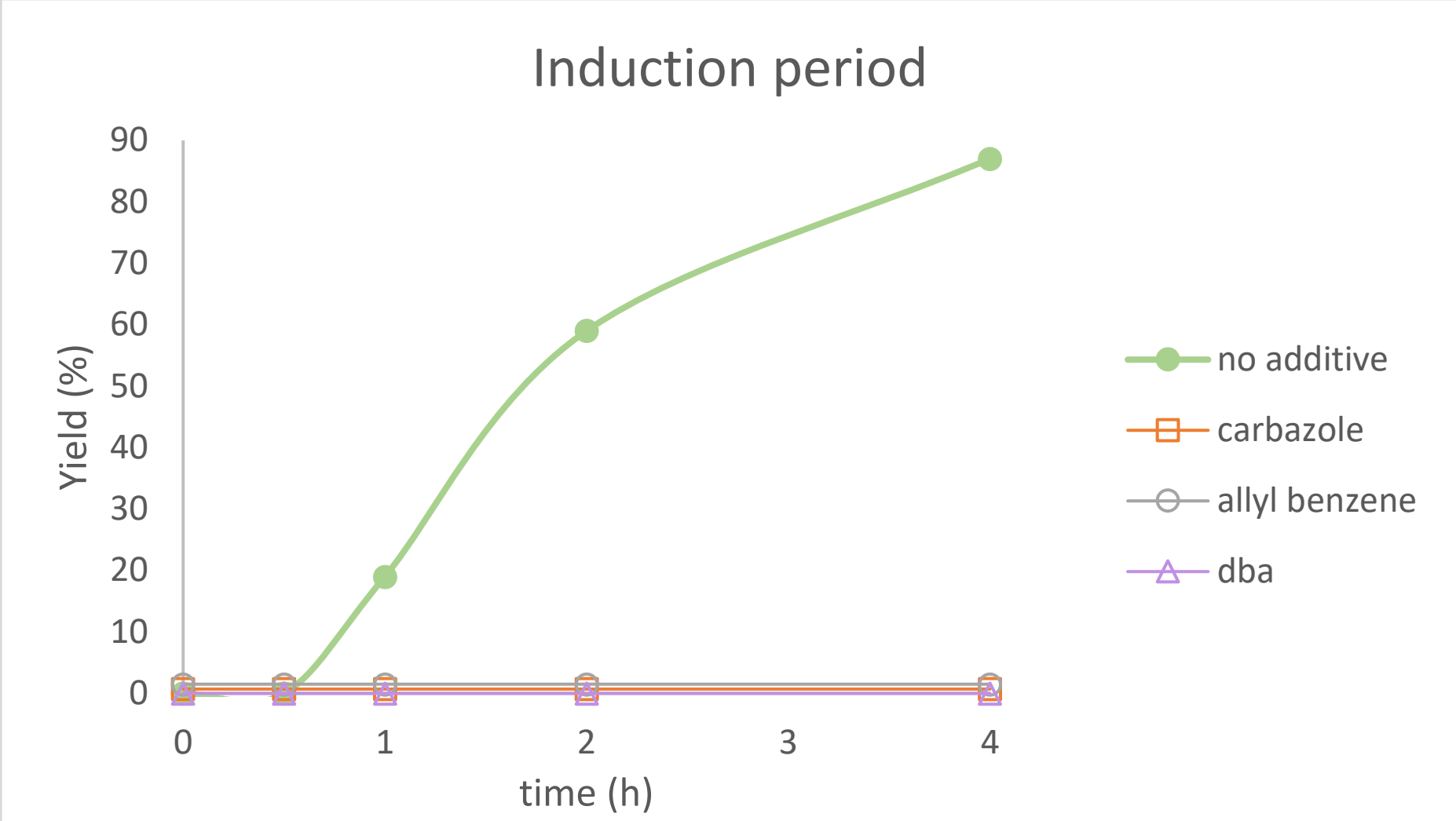

Figure S2. Kinetic comparison of the induction period in the standard reaction in the presence of dba (purple), allyl benzene (gray) and carbazole (orange). Standard conditions + 0.02 equiv. additive.

Figure S2 shows that the induction period under standard reaction conditions is markedly prolonged by the addition of small amounts of carbazole, allylbenzene, or dba, which are all species that arise as derivatives during the activation of common precatalysts. After 4 hours, no product is formed at all for all three additives. This observation suggests that the efficient activation of the MeNAP-TFA catalyst stems from the relatively rapid and facile decoordination of the MeNAP–ketone adduct, as opposed to other π-coordinating species.

Mechanistic Investigations

Competition experiments between electron rich and electron deficient aryl fluorides: An oven-dried vial (20 mL) was charged with $[Pd(MeNAP)TFA]_2$ (0.01 mmol, 1.8 mg, 1 mol%), and ^{t}BuBrettPhos (0.02 mmol, 2.42 mg, 2 mol%). The vial was purged in three alternating vacuum/nitrogen cycles and 1 mL of toluene was added and the mixture was pre-stirred for 5 minutes. Two separate stock solutions were prepared, both containing acetophenone **2a** (0.35 mmol, 1.5 equiv.) and internal standard. One contains also the 3-fluoroanisole **1a** (0.25 mmol, 1.0 equiv.), the other contains 4-fluoroanisole **1g** (0.25 mmol, 1.0 equiv.) 1 mL of these stock solutions was added to the respective catalyst/ligand mixtures and Lithium bis(trimethylsilyl)amide 1M in toluene (0.75 mmol, 3.0 equiv.) was added. The resulting mixture was stirred at 30 °C. The reactions were analyzed via gas chromatography with *n*-hexadecane as internal standard at specific time points within one hour.

Parallel experiments

t (min)	0	10	20	30	40
n(**1a**) (mol)	0.25	0.2379	0.23482	0.2348	0.2286
c(**1a**) (mol/L)	0.0909	0.0865	0.0854	0.08538	0.0831
$\ln(c(\mathbf{1a}))$	-2.39790	-2.44740	-2.46052	-2.46062	-2.48760
n(**1g**) (mol)	0.25	0.2136	0.1527	0.1269	0.1078
c(**1g**) (mol/L)	0.0909	0.0777	0.0555	0.0461	0.0392
$\ln(c(\mathbf{1g}))$	-2.39790	-2.55537	-2.89072	-3.07615	-3.23954

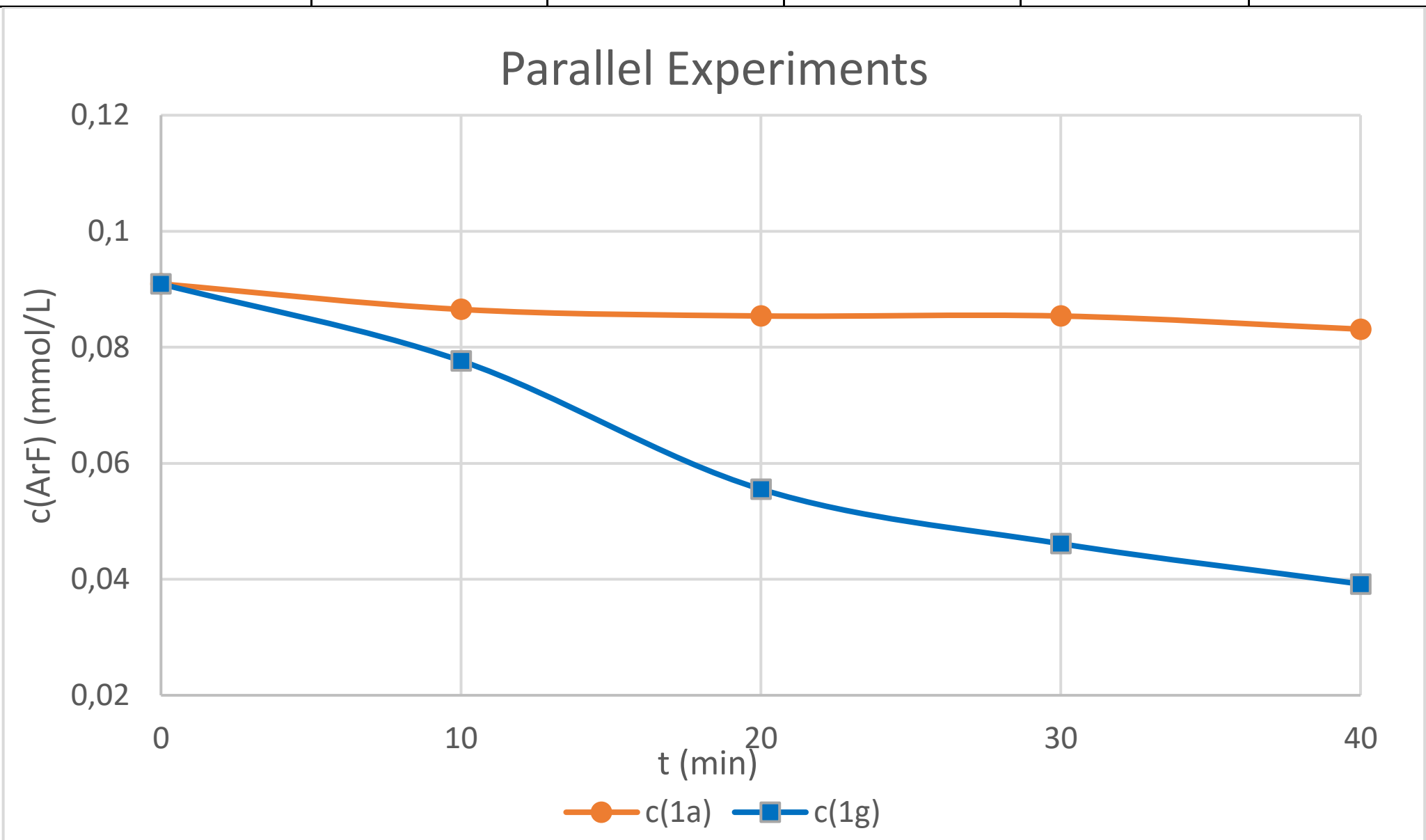

Figure S3. Time-dependent concentration profiles of **1a** and **1g** in separate parallel reactions.

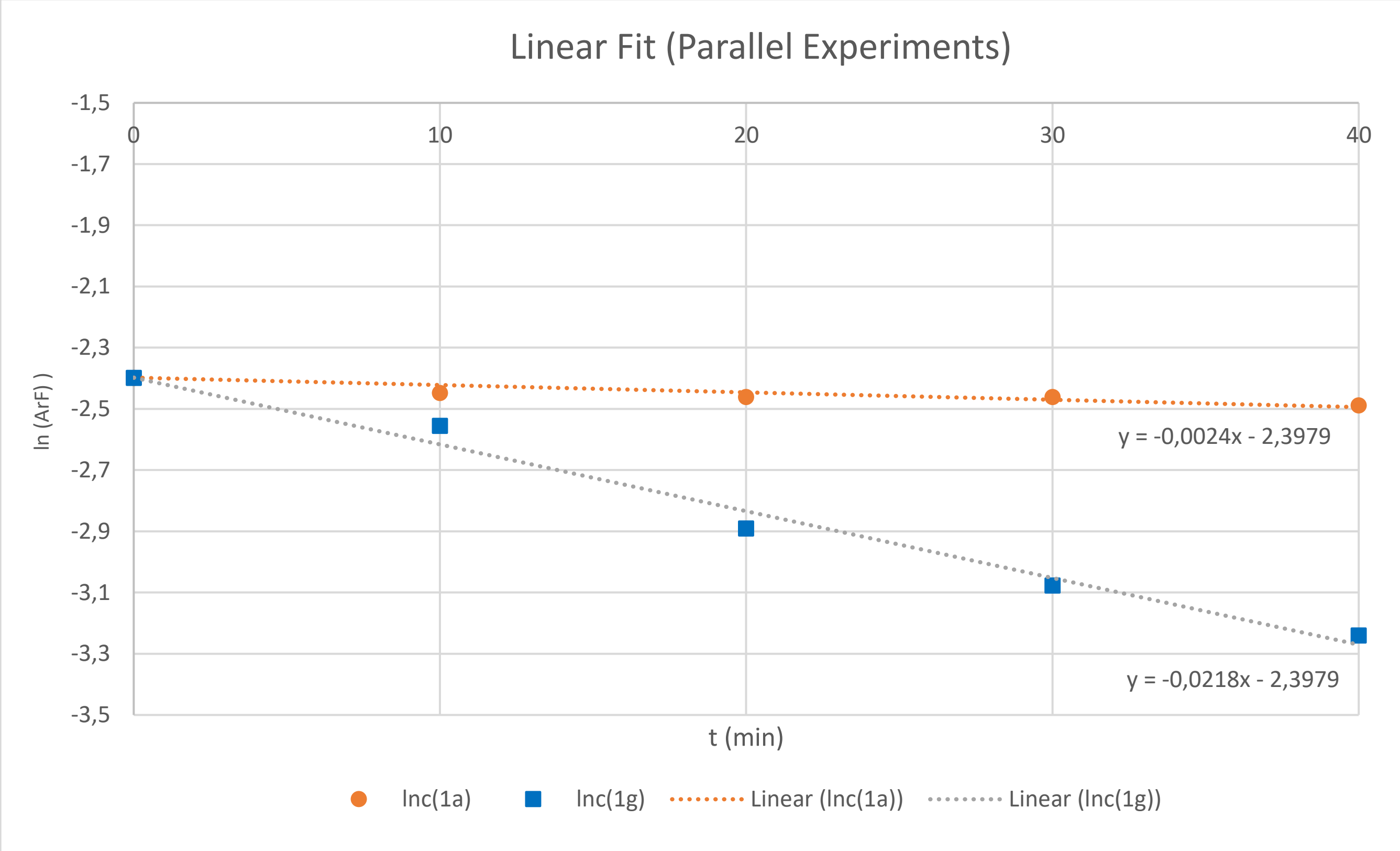

Figure S4. Linear fits of ln(c) versus time for **1a** and **1g** in parallel experiments.

Plotting the ln([ArF](t)) vs. t (min) for early to mid-stage data points should give a straight line with slope = - k´ArF. For the linear fit only the data points within the first 40 minutes were considered:

The parallel reaction of aryl fluoride **1a** with acetophenone **2a** results in k'_{1a} = 0.0024 min^{-1}, while the parallel experiment of **1g** with acetophenone **2a** yields k'_{1g} = 0.0218 min^{-1}.

The ratio of both reaction rates is defined as follows:

$$\frac{k'_{1g}}{k'_{1a}} \approx 9.1$$

<u>**Table S2**. Activation experiment:</u> An oven-dried vial (20 mL) was charged with $[Pd(MeNAP)TFA]_2$ (0.2 equiv.), and ^{t}BuBrettPhos (0.4 mmol, 4 equiv.). The vial was purged in three alternating vacuum/nitrogen cycles and 4 mL of toluene were added and the mixture was pre-stirred for 5 minutes. Acetophenone (1 equiv.) and LiHMDS (2 equiv.) were added and the reaction was stirred at 30 °C. At a 5 minute interval, the reaction was quenched with acid, and after GC workup analyzed via gas chromatography using *n*-hexadecane as internal standard.

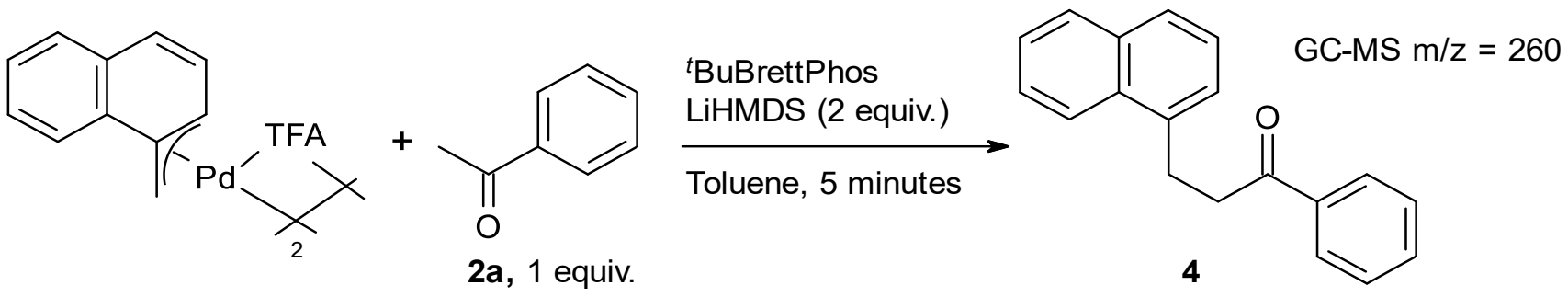

Computational Studies

Density functional theory (DFT) calculations were performed using the ORCA program package (version 6.1.).[4–6] Geometry optimizations of all intermediates and transition states were carried out using the B97M-D4 exchange–correlation functional in combination with the def2-SVP basis set and auxiliary basis def2/J.[7,8] Frequency calculations were performed at the same level of theory to verify the stationary points. Solvent effects were included using the SMD implicit solvation model with Toluene as the solvent.[9] Thermal corrections were obtained from the frequency calculations at 298.15 K. Single-point energy calculations were performed using the def2-TZVP basis set to obtain more accurate electronic energies. Reported energies correspond to Gibbs free energies obtained by combining these single-point electronic energies with thermal corrections derived from the B97M-D4/def2-SVP frequency calculations. Transition states contain exactly one imaginary frequency, and IRC calculations were carried out to confirm that each transition state connects the reactant and product minima.

The Cartesian coordinates (XYZ files) are provided in the accompanying ZIP archive, and the computed energies are summarized in the corresponding Excel files.

The ligated Pd(0) complex was computed to validate the preferred coordination geometry of the ligand. Figure S4 shows the π-coordination of Pd to the biphenyl unit of *ᵗ*BuBrettPhos is significantly more stable than the twisted methoxy-coordinated analogue, by -118 kJ/mol (Figure S5, a). A similar trend is observed for the initial L-Pd(MeNAP)(TFA) complex (-16 kJ/mol), which motivated the use of this geometry throughout the catalytic cycle calculations. Moreover, *π*-coordination of the ligand appears to stabilize the *η*¹-coordination of the MeNAP fragment (Figure S5, b).

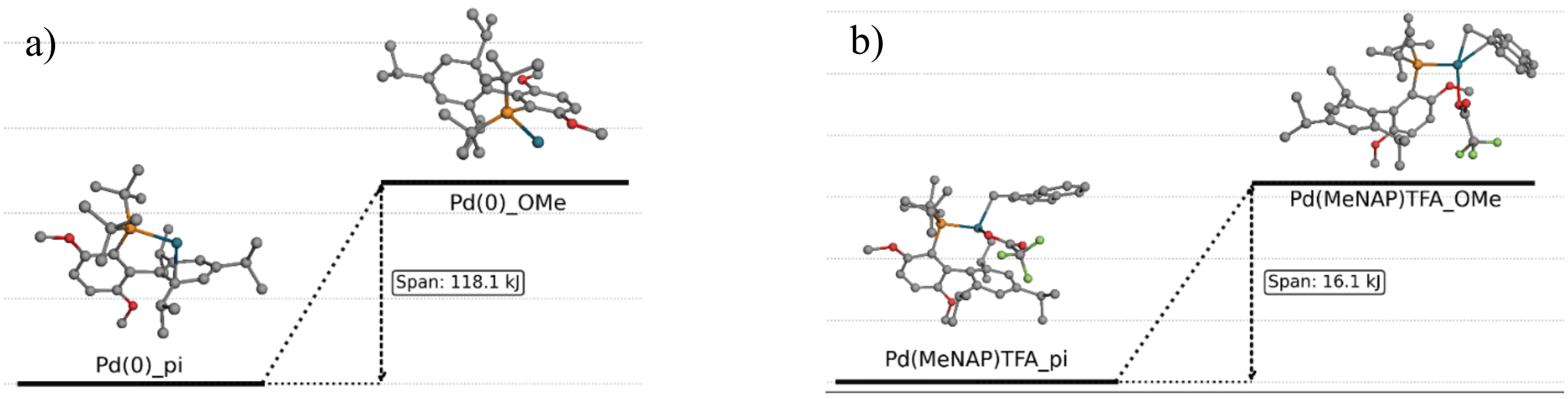

Figure S5. Comparison of the ligated Pd⁰ and Pd² geometries, revealing a pronounced preference for *π*-coordination, as opposed to the less favorable twisted methoxy-coordination.

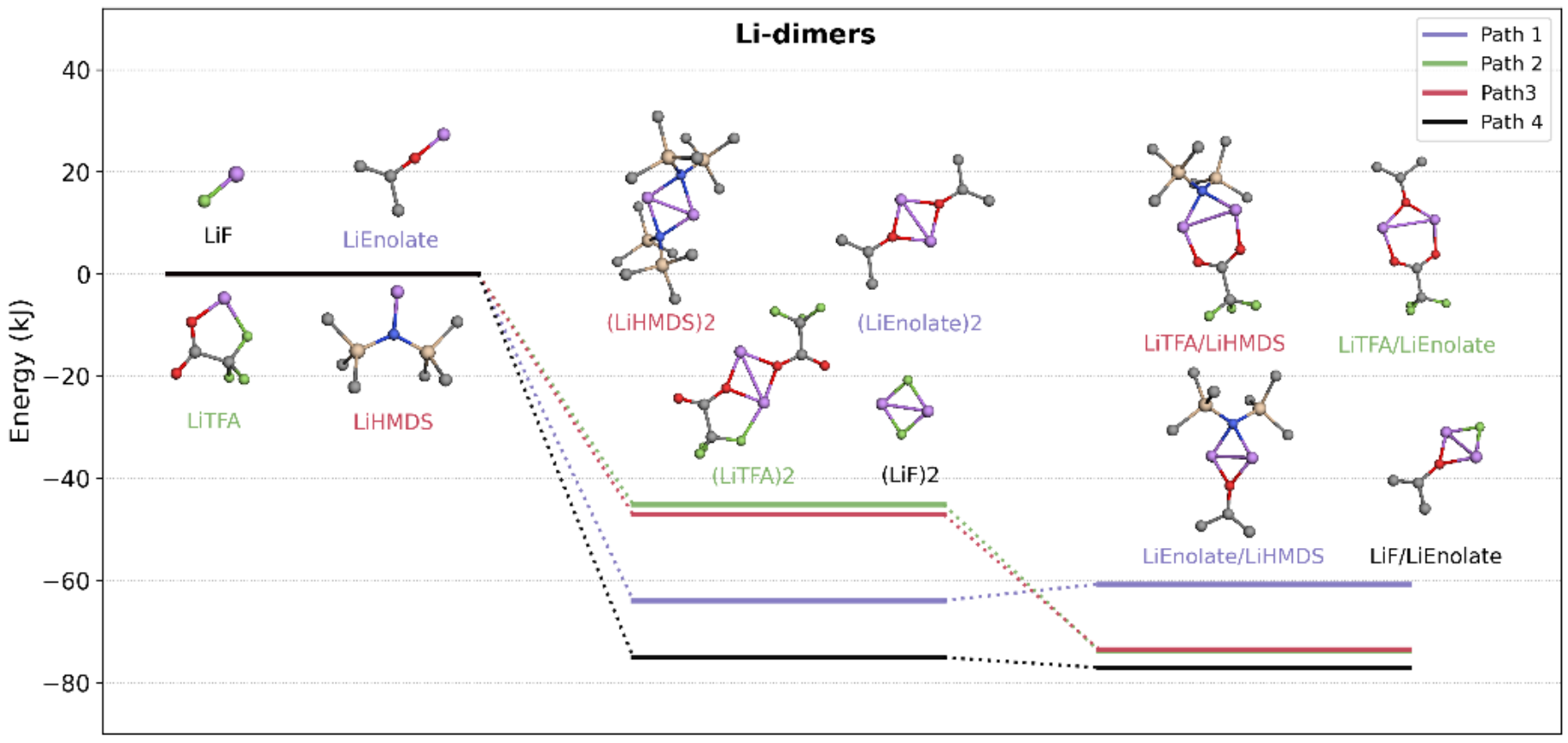

Figure S6. Comparison of Li-monomers with their corresponding Li-homodimers and Li-heterodimers.

Figure S6 shows that the formation of the lithium homodimers from the monomers is strongly exergonic in toluene solvent. In contrast, the exchange between homo- and heterodimers is only slightly exergonic or thermoneutral.

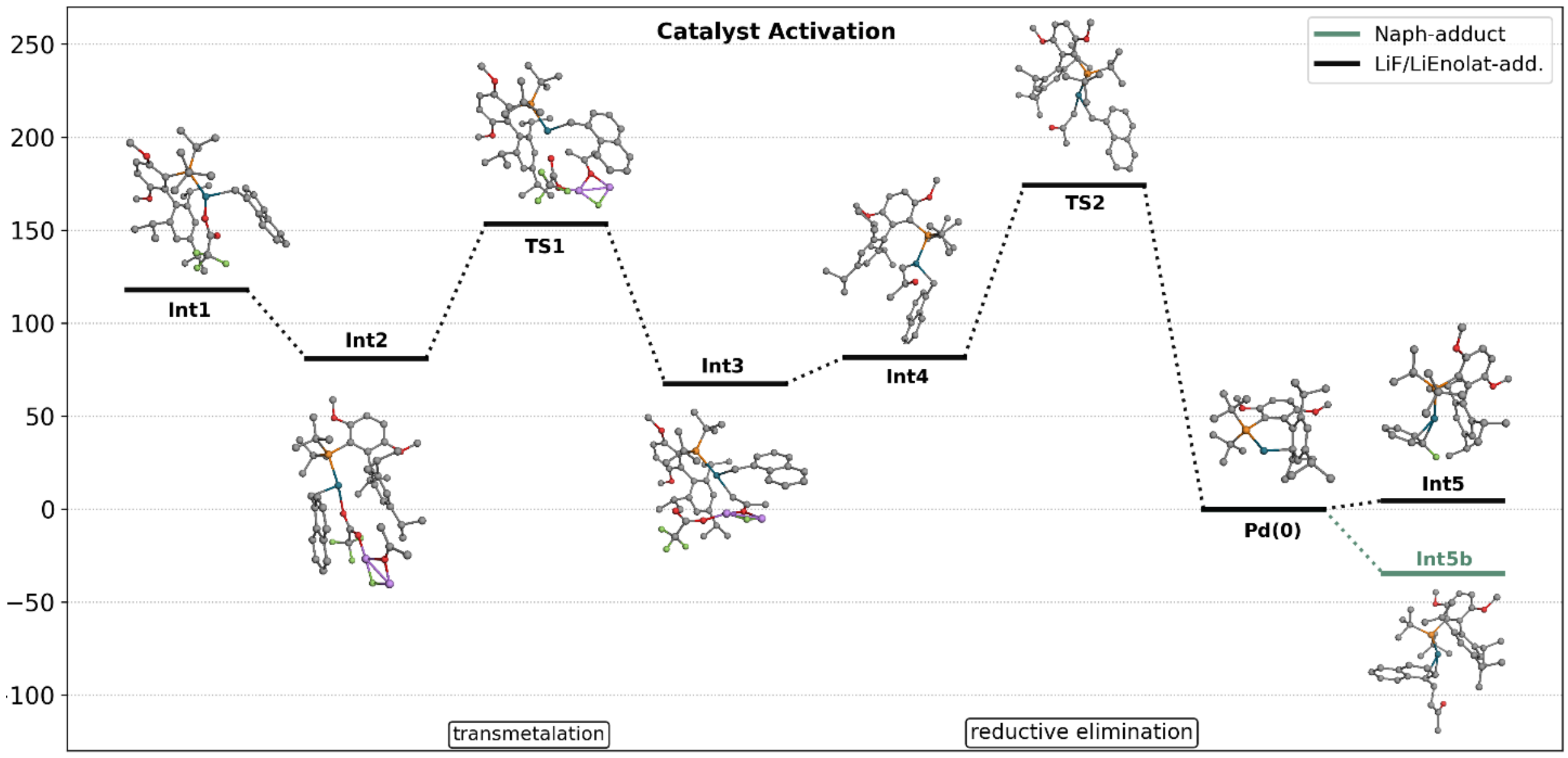

Figure S7. Catalyst activation following transmetalation of the MeNAP fragment by the enolate, and subsequent reductive elimination. An unproductive pathway by naphthyl-coordination (green) may compete with the coordination of the aryl fluoride.

We calculated the catalyst activation to begin with coordination of an enolate-containing Li-dimer to the ligated Pd–MeNAP–TFA complex **Int1**, initiating the required transmetalation step. For computational efficiency, a simplified LiF/LiEnolate heterodimer was employed. Coordination of this dimer is exergonic (**Int2**, −37 kJ/mol). The corresponding transmetalation transition state (**TS1**) lies 72 kJ/mol above the preceding intermediate and leads to the enolate-containing Pd–MeNAP complex (**Int3**). Subsequently, a slightly endergonic de-coordination of the LiTFA/LiF dimer affords **Int4**, enabling reductive elimination. The associated transition state (**TS2**) is 92 kJ/mol in energy and, according to IRC calculations, leads to the reductively eliminated MeNAP-ketone weakly coordinated to Pd(0) via the carbonyl oxygen. As this interaction is higher in energy than the separated Pd(0) and MeNAP

ketone (+7 kJ/mol), direct de-coordination is expected. The liberated MeNAP-ketone can, however, re-coordinate to **Pd(0)** through the aryl group to form a naphthyl-adduct (**Int5x**) that is stabilized by -35 kJ/mol. This species represents an unproductive pathway that competes with, and potentially inhibits, the productive catalytic cycle.

The catalytic cycle is initiated by π-coordination of the aryl fluoride to the ligated Pd(0) complex, which is slightly endergonic by 5 kJ/mol (**Int5**). It is well described that oxidative additions of aryl halides often proceed though initial coordination to a more distal π-bond, then move closer to the substituent and finally insert, so that we have simplified the calculations by starting directly from 1-coordinated fluorobenzene. The oxidative addition was first computed for the non-assisted pathway, yielding a barrier of 92 kJ/mol (**TS3**), which is somewhat high for a reaction proceeding at room temperature. The presence of lithium salt (hetero)dimers markedly lowers this barrier. The coordination of lithium dimers to fluorobenzene coordinated to Pd(0) is only slightly uphill in energy, e.g. (**Int5a**: +12 kJ/mol; **Int5b**: +10 kJ/mol).

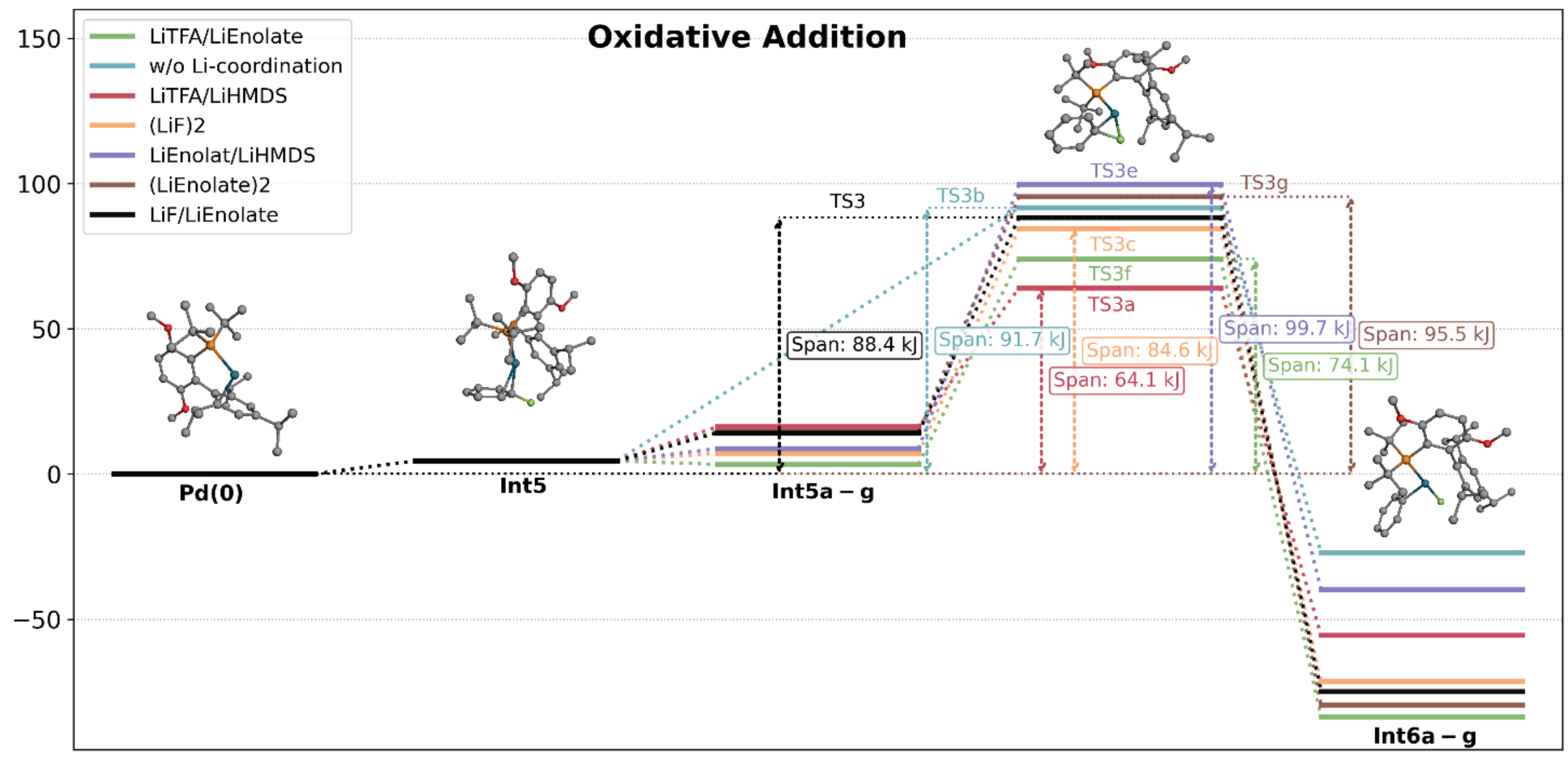

Figure S8. Oxidative addition barriers computed for different Li-dimer adducts, showing the energy span in respect to Pd(0).

The barrier for oxidative addition was computed for a series of Li-salt dimers (Figure S8). The transition state **TS3**, which lacks Li coordination, lies at 92 kJ/mol. Coordination by a Li enolate dimer (**TS3g**) or a mixed LiEnolate/LiHMDS dimer (**TS3e**) increases the barrier to 96 kJ/mol and 100 kJ/mol, respectively. Coordination by LiF-containing dimers leads to only modest stabilization: the LiF/LiEnolate adduct (**TS3b**) exhibits a barrier of 89 kJ/mol, comparable to the simple LiF dimer (**TS3c**, 85 kJ/mol). A pronounced stabilization is observed for LiTFA-containing dimers. The LiTFA/Li enolate adduct (**TS3f**) lowers the barrier to 74 kJ/mol, while in combination with LiHMDS, the LiTFA/LiHMDS coordination provides the greatest effect, with **TS3a** exhibiting a barrier of just 64 kJ/mol. For the computed transition state **TS3f**, a small residual imaginary frequency (-7.79 cm^{-1}) persisted, despite repeated optimizations with tightened convergence criteria. As this mode corresponds to a low-energy torsional motion, it is considered an artefact and does not affect the validity of the results.

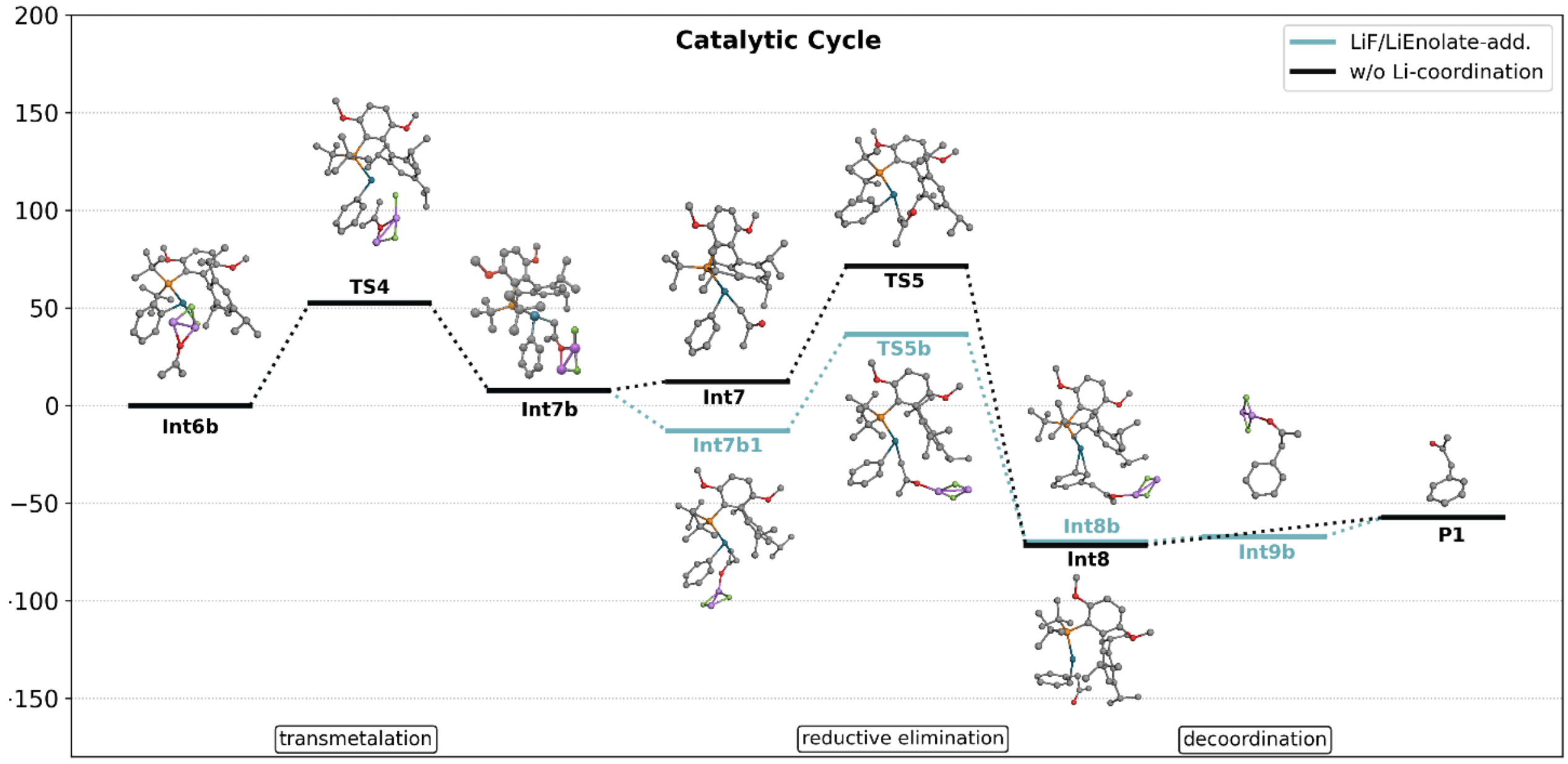

Figure S9. Continuation of the catalytic cycle, exhibiting the transmetalation, reductive elimination and final product release.

Following oxidative addition, lithium dimers can readily associate and dissociate, allowing coordination of an enolate-containing lithium dimer to promote transmetalation (**Int6b**). A simplified LiF/LiEnolate dimer was used in the calculations to reduce computational cost. The transmetalation transition state **TS4** (ΔG= 53 kJ/mol) was located and leads, as confirmed by IRC analysis, to intermediate **Int7b**. This species can then readily rearrange to **Int7b1** via a change in the coordination mode of the lithium dimer. In **Int7b1**, the carbonyl oxygen adopts an η^1-coordination to lithium, which is energetically preferred over the corresponding bridged coordination mode by 21 kJ/mol. The associated transition state for this conformational change is very shallow and could not be reliably characterized. Alternatively, the lithium dimer may dissociate fully to give **Int7**. From this point, reductive elimination can proceed either with the lithium dimer still coordinated or after its slightly endergonic dissociation from the Pd(**II**) intermediate, leading to **Int8** and **Int8b**, respectively. The Li-dimer-coordinated **TS5b** lies slightly lower in energy with 50 kJ/mol than the **TS5** with no coordination, which has an energy of 59 kJ/mol. IRC calculations confirm the direct connection between **Int7** and **Int8**, as well as between **Int7b** and **Int8b**. Following reductive elimination, the ketone product is likely to remain coordinated to palladium until it is displaced by a new aryl fluoride, releasing product **P1**, thus regenerating the catalytically active species, and completing the catalytic cycle.

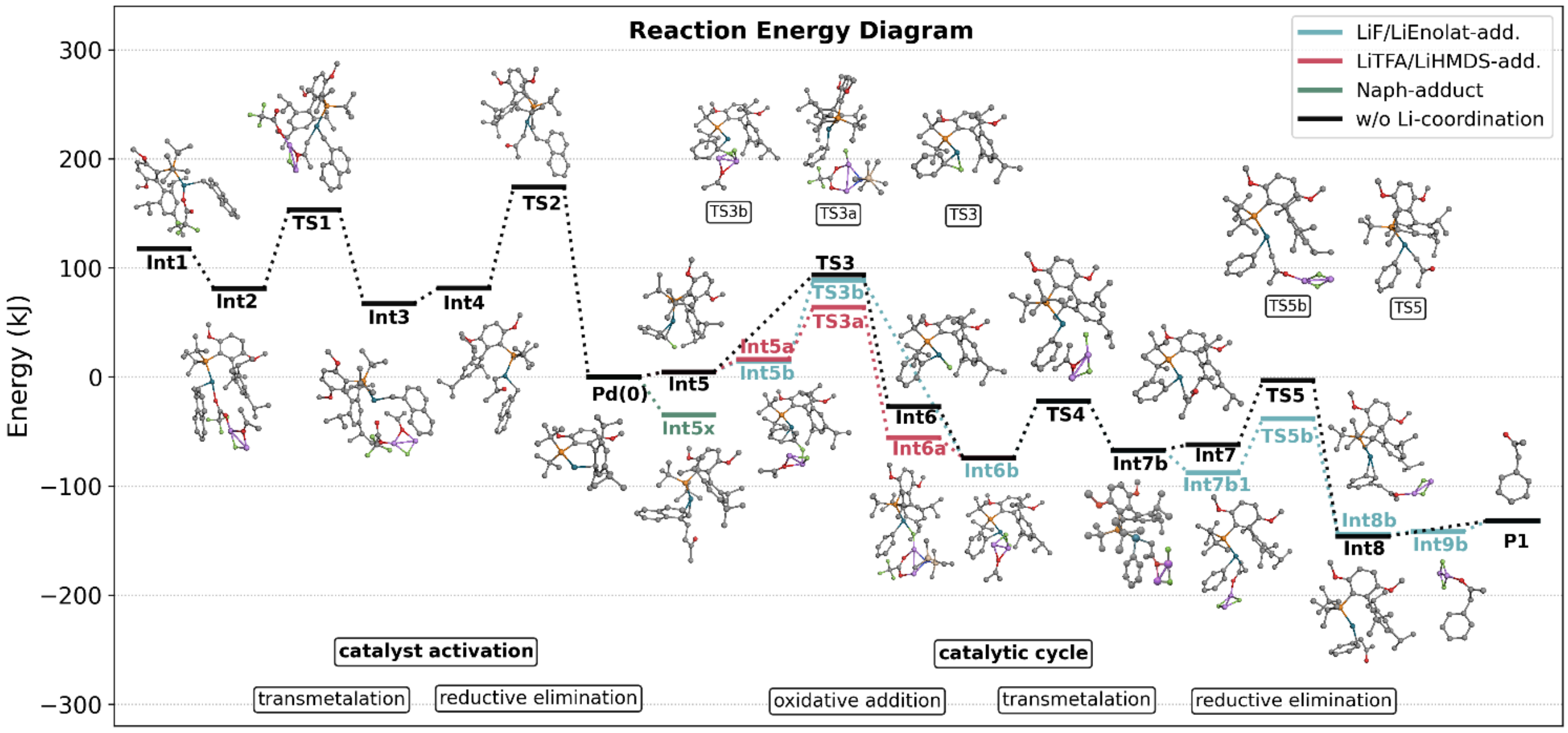

Figure S10. Computational investigations of the full energy profile of the catalyst activation, and enolate cross-coupling reaction by aryl fluoride activation. Li-salt dimers (black, red) facilitate the oxidative addition step.

Validation of the level of theory

To assess the reliability of the chosen computational protocol, we investigated the influence of different functionals (B97m-D4 and PBE0), solvent models (SMD and CPCM), and basis sets (def2-SVP and def2-TZVP) for one representative reaction step, namely the oxidative addition with and without Li_2F_2 coordination. No significant deviations were observed, and the principal conclusion, namely that coordination of the Li_2F_2 unit lowers the barrier of the rate-determining oxidative addition step, was found to be independent of the computational setup employed. For the def2-TZVP calculations, the changes in relative energies were below 5 kJ/mol. The largest deviations were obtained with the hybrid functional PBE0, which increased the absolute barrier heights by approximately 15 kJ/mol. Nevertheless, the difference between the transition states with and without Li_2F_2 coordination remained essentially unchanged compared to the B97m-D4 results. These findings indicate that the B97m-D4/def2-SVP level with def2-TZVP single-point corrections and the SMD solvent model provides a reasonable compromise between accuracy and computational cost.

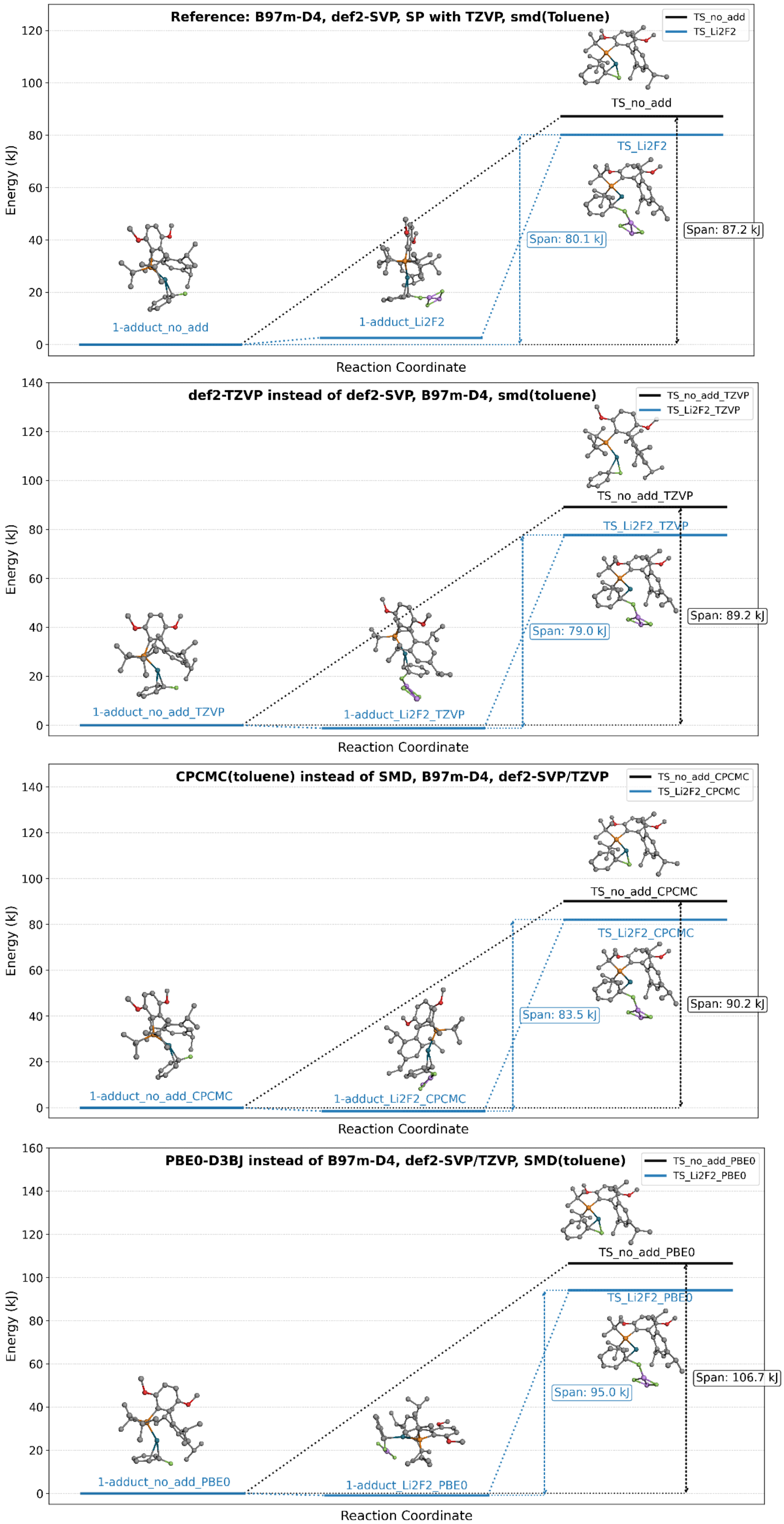

Figure S11. Comparison of relative energies of one representative reaction step under different computational methods.

Synthesis and Characterization of Products

2-(4-methoxyphenyl)-1-phenylethanone (**3aa**) [CAS: 24845-40-7]

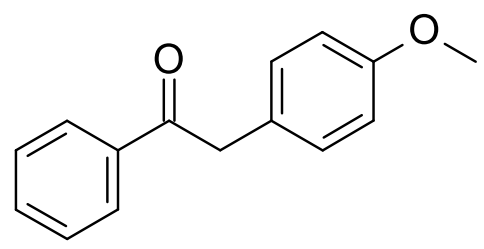

Following the general procedure **A**, 4-fluoroanisol (63.1 mg, 0.5 mmol, 1 equiv.) and acetophenone (92 mg, 0.750 mmol, 1.5 equiv.) yielded the product **3aa** (103.2 mg, 91%) as white solid, purified *via* column chromatography (SiO_2, cyclohexane/EtOAc 10%).

m.p.: 87 °C
TLC: R_f = 0.31 (Cy/EA 10%)
^{1}H NMR (400 MHz, $CDCl_3$): *δ* = 7.98 - 8.06 (m, 2 H), 7.53 - 7.60 (m, 1 H), 7.43 - 7.50 (m, 2 H), 7.15 - 7.22 (m, 2 H), 6.85 - 6.91 (m, 2 H), 4.24 (s, 2 H), 3.80 (s, 3 H) ppm.
^{13}C NMR (101 MHz, $CDCl_3$): *δ* = 197.9, 158.5, 136.6, 133.1, 130.4, 128.6, 128.6, 126.5, 114.1, 55.2, 44.6 ppm.
The NMR match those reported in literature.[10]

2-(4-Methylphenyl)-1-phenylethanone (**3ba**) [CAS: 2430-99-1]

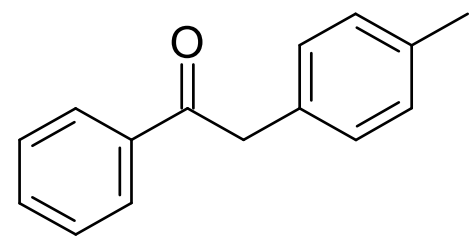

Following the general procedure **A**, 4-fluorotoluene (55.1 mg, 0.5 mmol, 1 equiv.) and acetophenone (92 mg, 0.750 mmol, 1.5 equiv.) yielded the product **3ba** (94.5 mg, 90%) as white solid, purified *via* column chromatography (SiO_2, cyclohexane/EtOAc 10%).

m.p.: 95 °C
TLC: R_f = 0.32 (Cy/EA 10%)
^{1}H NMR (400 MHz, $CDCl_3$): *δ* = 7.99 - 8.07 (m, 2 H), 7.53 - 7.61 (m, 1 H), 7.42 - 7.51 (m, 2 H), 7.11 - 7.22 (m, 4 H), 4.27 (s, 2 H), 2.34 (s, 3 H) ppm.
^{13}C NMR (101 MHz, $CDCl_3$): *δ* = 197.8, 136.6, 136.4, 133.0, 131.4, 129.4, 129.3, 128.6, 45.1, 21.0 ppm.
The NMR match those reported in literature.[11]

1,2-diphenylethanone (**3ca**) [CAS: 451-40-1]

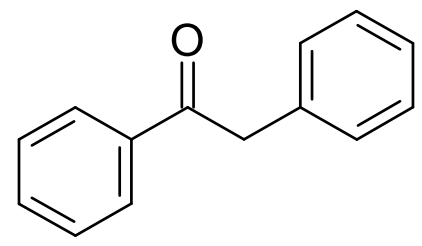

Following the general procedure **A**, fluorobenzene (48.5 mg, 0.5 mmol, 1 equiv.) and acetophenone (92 mg, 0.750 mmol, 1.5 equiv.) yielded the product **3ca** (85.9 mg, 88%) as white solid, purified *via* column chromatography (SiO_2, cyclohexane/EtOAc 10%).

m.p.: 60 °C
TLC: R_f = 0.32 (Cy/EA 10%)
^{1}H NMR (400 MHz, $CDCl_3$): *δ* = 7.99 - 8.05 (m, 2 H), 7.53 - 7.58 (m, 1 H), 7.43 - 7.48 (m, 2 H), 7.30 - 7.35 (m, 2 H), 7.22 - 7.29 (m, 3 H), 4.29 (s, 2 H) ppm.
^{13}C NMR (101 MHz, $CDCl_3$): *δ* = 197.5, 136.5, 134.5, 133.1, 129.4, 128.6, 128.6, 128.5, 126.8, 45.4 ppm.
The NMR match those reported in literature.[12]

2-(2-Methoxyphenyl)-1-phenylethanone (**3da**) [CAS: 27356-33-8]

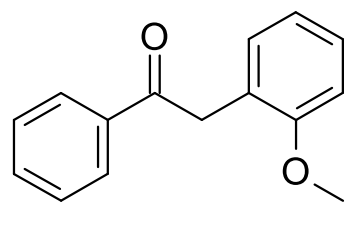

Following the general procedure **A**, 2-fluoroanisol (63.1 mg, 0.50 mmol, 1 equiv.) and acetophenone (92 mg, 0.750 mmol, 1.5 equiv.) yielded the product **3da** (88.4 mg, 78%) as white solid, purified *via* column chromatography (SiO_2, cyclohexane/EtOAc 10%).

m.p.: 58 °C
TLC: R_f = 0.42 (Cy/EA 10%)
^{1}H NMR (400 MHz, $CDCl_3$): *δ* = 8.05 - 8.13 (m, 2 H), 7.54 - 7.64 (m, 1 H), 7.44 - 7.54 (m, 2 H), 7.30 (td, *J* = 7.8, 1.7 Hz, 1 H), 7.22 (dd, *J* = 7.4, 1.5 Hz, 1 H), 6.88 - 7.03 (m, 2 H), 4.32 (s, 2 H), 3.81 (s, 3 H) ppm.
^{13}C NMR (101 MHz, $CDCl_3$): *δ* = 197.8, 157.1, 136.9, 132.8, 130.9, 128.4, 128.3, 128.3, 123.6, 120.5, 110.4, 55.2, 39.9 ppm.
The NMR match those reported in literature.[12]

2-(2-Methylphenyl)-1-phenylethanone (**3ea**) [CAS: 5033-67-0]

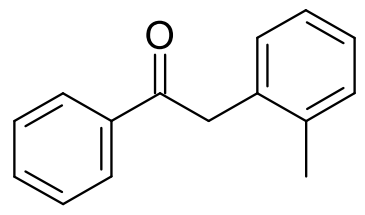

Following the general procedure **A**, 2-fluorotoluene (55.1 mg, 0.5 mmol, 1 equiv.) and acetophenone (92 mg, 0.750 mmol, 1.5 equiv.) yielded the product **3ea** (65.8 mg, 63%) as white solid, purified *via* column chromatography (SiO_2, cyclohexane/EtOAc 10%).

m.p.: 65 °C
TLC: R_f = 0.30 (Cy/EA 10%)
^{1}H NMR (400 MHz, $CDCl_3$): δ = 8.01 - 8.10 (m, 2 H), 7.60 (t, *J* = 7.4 Hz, 1 H), 7.50 (t, *J* = 7.6 Hz, 2 H), 7.09 - 7.26 (m, 4 H), 4.33 (s, 2 H), 2.28 (s, 3 H) ppm.
^{13}C NMR (101 MHz, $CDCl_3$): δ = 197.4, 136.9, 133.4, 133.1, 130.3, 130.3, 128.6, 128.3, 127.2, 126.1, 43.5, 19.8 ppm.
The NMR match those reported in literature.[12]

2-(2-Fluorophenyl)-1-phenylethanone (**3fa**) [CAS: 3826-47-9]

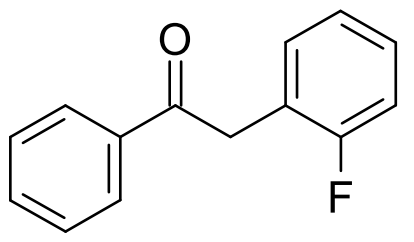

Following the general procedure **A**, 1,2-difluorobenzene (57 mg, 0.5 mmol, 1 equiv.) and acetophenone (92 mg, 0.750 mmol, 1.5 equiv.) yielded the product **3fa** (88.6 mg, 83%) as white solid, purified *via* column chromatography (SiO_2, cyclohexane/EtOAc 10%).

m.p.: 77 °C
TLC: R_f = 0.53 (Cy/EA 10%)
^{1}H NMR (400 MHz, $CDCl_3$): δ = 8.02 - 8.08 (m, 2 H), 7.56 - 7.63 (m, 1 H), 7.46 - 7.53 (m, 2 H), 7.27 - 7.32 (m, 1 H), 7.22 - 7.27 (m, 1 H), 7.05 - 7.17 (m, 2 H), 4.35 (s, 2 H) ppm.
^{13}C NMR (101 MHz, $CDCl_3$): δ = 196.3, 160.9 (d, *J* = 245.6 Hz), 136.4, 133.3, 131.6 (d, *J* = 4.4 Hz), 128.9 (d, *J* = 8.0 Hz), 128.7, 128.4, 124.1 (d, *J* = 3.6 Hz), 121.8 (d, *J* = 16.0 Hz), 115.4 (d, *J* = 21.9 Hz) 38.6 (d, *J*=2.2 Hz) ppm.
^{19}F NMR (377 MHz, $CDCl_3$): δ = -117.16 ppm.
The NMR match those reported in literature.[13]

2-(3-Methoxyphenyl)-1-phenylethanone (**3ga**) [CAS: 29955-26-8]

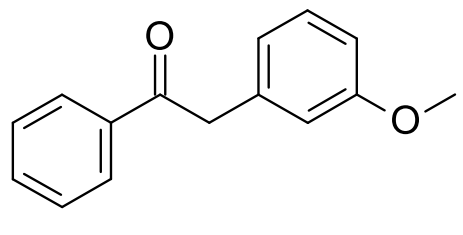

Following the general procedure **A**, 3-fluoroanisol (63.7 mg, 0.5 mmol, 1 equiv.) and acetophenone (92 mg, 0.750 mmol, 1.5 equiv.) yielded the product **3ga** (109.5 mg, 97%) as clear oil, purified *via* column chromatography (SiO_2, cyclohexane/EtOAc 10%).

TLC: R_f = 0.47 (Cy/EA 10%)
^{1}H NMR (400 MHz, $CDCl_3$): δ = 7.99 - 8.05 (m, 2 H), 7.53 - 7.60 (m, 1 H), 7.43 - 7.51 (m, 2 H), 7.21 - 7.27 (m, 1 H), 6.77 - 6.91 (m, 3 H), 4.27 (s, 2 H), 3.80 (s, 3 H) ppm.
^{13}C NMR (101 MHz, $CDCl_3$): δ = 197.5, 159.8, 136.6, 136.0, 133.1, 129.6, 128.6, 121.8, 115.1, 112.4, 55.2, 45.6 ppm.
The NMR match those reported in literature.[14]

2-(1-Naphthalenyl)-1-phenylethanone (**3ha**) [CAS: 16216-08-3]

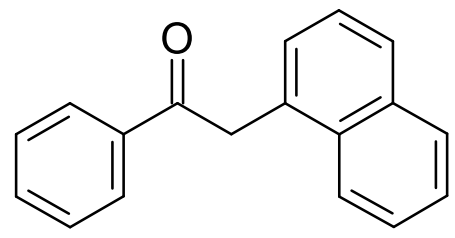

Following the general procedure **A**, 1-fluoronaphthalene (73.1 mg, 0.5 mmol, 1 equiv.) and acetophenone (92 mg, 0.750 mmol, 1.5 equiv.) yielded the product **3ha** (113.5 mg, 92%) as white solid, purified *via* column chromatography (SiO_2, cyclohexane/EtOAc 10%).

m.p.: 106 °C
TLC: R_f = 0.30 (Cy/EA 10%)
^{1}H NMR (400 MHz, $CDCl_3$): δ = 8.06 - 8.15 (m, 2 H), 7.86 - 7.94 (m, 2 H), 7.82 (d, *J* = 8.1 Hz, 1 H), 7.58 - 7.64 (m, 1 H), 7.42 - 7.55 (m, 5 H), 7.35 - 7.41 (m, 1 H), 4.76 (s, 2 H) ppm.
^{13}C NMR (101 MHz, $CDCl_3$): δ = 197.6, 136.7, 133.9, 133.2, 132.2, 131.3, 128.8, 128.7, 128.5, 128.0, 127.9, 126.3, 125.7, 125.4, 123.8, 43.1 ppm.
The NMR match those reported in literature.[15]

2-[4-(Methylthio)phenyl]-1-phenylethanone (**3ia**) [CAS: 177560-74-6]

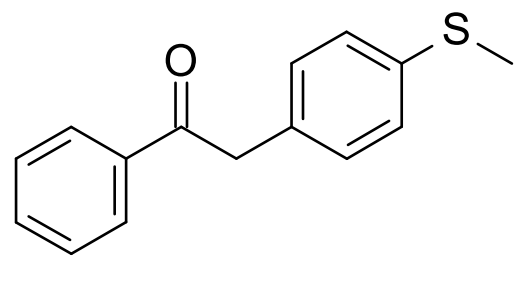

Following the general procedure **A**, 4-fluorothioanisole (71.1 mg, 0.5 mmol, 1 equiv.) and acetophenone (92 mg, 0.750 mmol, 1.5 equiv.) yielded the product **3ia** (75.0 mg, 62%) as white solid, purified *via* column chromatography (SiO_2, cyclohexane/EtOAc 10%).

m.p.: 131 °C
TLC: R_f = 0.45 (Cy/EA 10%)
^{1}H NMR (400 MHz, $CDCl_3$): δ = 7.98 - 8.05 (m, 2 H), 7.54 - 7.61 (m, 1 H), 7.44 - 7.50 (m, 2 H), 7.16 - 7.26 (m, 4 H), 4.26 (s, 2 H), 2.47 (s, 3 H) ppm.
^{13}C NMR (101 MHz, $CDCl_3$): δ = 197.5, 136.9, 136.5, 133.2, 131.3, 129.9, 128.6, 128.6, 127.0, 44.9, 15.9 ppm.
The NMR match those reported in literature.[16]

1-Phenyl-2-[4-(trifluoromethoxy)phenyl]ethanone (**3ja**) [CAS: 1496328-62-1]

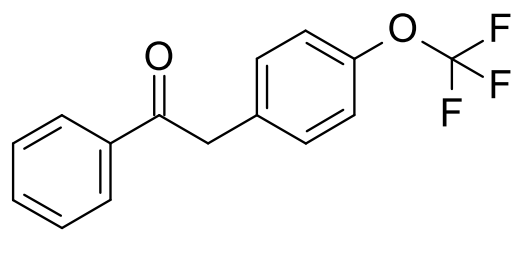

Following the general procedure **A**, 1-fluoro-4-(trifluoromethoxy)benzene (90.1 mg, 0.5 mmol, 1 equiv.) and acetophenone (92 mg, 0.750 mmol, 1.5 equiv.) yielded the product **3ja** (97.4 mg, 70%) as white solid, purified *via* column chromatography (SiO_2, cyclohexane/EtOAc 10%).

TLC: R_f = 0.49 (Cy/EA 10%)
^{1}H NMR (400 MHz, $CDCl_3$): δ = 8.00 - 8.06 (m, 2 H), 7.58 - 7.63 (m, 1 H), 7.47 - 7.53 (m, 2 H), 7.28 - 7.32 (m, 2 H), 7.19 (m, 2 H), 4.31 (s, 2 H) ppm.
^{19}F NMR (377 MHz, $CDCl_3$): δ = -57.9 ppm.
^{13}C NMR (101 MHz, $CDCl_3$): δ = 197.0, 148.2, 136.4, 133.4, 133.1, 130.9, 128.7, 128.5, 121.1, 120.5 (q, J = 255.2 Hz) 44.5 ppm.
The NMR match those reported in literature.[17]

1-phenyl-2-[4-(trimethylsilyl)phenyl]ethanone (**3ka**) [CAS: 1884595-69-0]

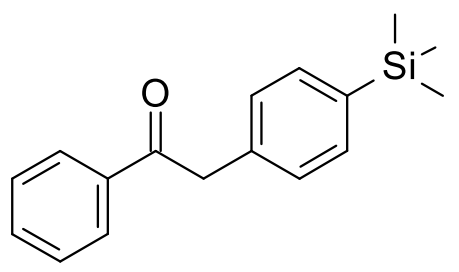

Following the general procedure **A**, (4-fluorophenyl)trimethylsilane (84.1 mg, 0.5 mmol, 1 equiv.) and acetophenone (92 mg, 0.750 mmol, 1.5 equiv.) yielded the product **3ka** (106.1 mg, 79%) as white solid, purified *via* column chromatography (SiO_2, cyclohexane/EtOAc 10%).

TLC: R_f = 0.65 (Cy/EA 10%)
^{1}H NMR (400 MHz, $CDCl_3$): δ = 8.05 - 8.12 (m, 2 H), 7.54 - 7.62 (m, 3 H), 7.47 - 7.52 (m, 2 H), 7.33 (d, J = 8.0 Hz, 2 H), 4.33 (s, 2 H), 0.32 (s, 9 H) ppm.
^{13}C NMR (101 MHz, $CDCl_3$): δ = 197.4, 138.6, 136.5, 135.0, 133.6, 133.1, 128.8, 128.5, 128.5, 45.3, -1.2 ppm.
The NMR match those reported in literature.[18]

1-phenyl-2-[4-(4,4,5,5-tetramethyl-1,3,2-dioxaborolan-2-yl)phenyl]ethanone (**3la**) [CAS: 1802939-37-2]

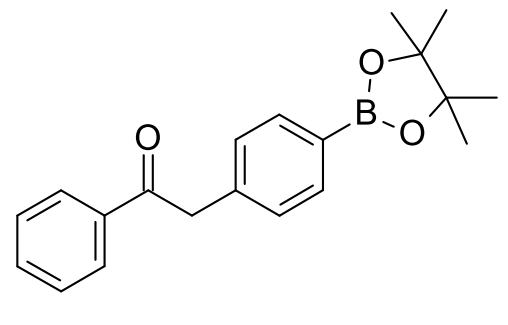

Following the general procedure **A**, 2-(4-fluorophenyl)-4,4,5,5-tetramethyl-1,3,2-dioxaborolane (111 mg, 0.5 mmol, 1 equiv.) and acetophenone (92 mg, 0.750 mmol, 1.5 equiv.) yielded the product **3la** (104 mg, 65%) as white solid, purified *via* column chromatography (SiO_2, cyclohexane/EtOAc 10%).

m.p.: 99 °C
TLC: R_f = 0.13 (Cy/EA 5%)
^{1}H NMR (400 MHz, $CDCl_3$): δ = 7.97 - 8.03 (m, 2 H), 7.78 (d, J = 8.0 Hz, 2 H), 7.54 (d, J = 7.4 Hz, 1 H), 7.42 - 7.49 (m, 2 H), 7.29 (d, J = 8.0 Hz, 2 H), 4.31 (s, 2 H), 1.34 (s, 12 H) ppm.
^{13}C NMR (101 MHz, $CDCl_3$): δ = 197.4, 137.7, 136.5, 135.1, 133.1, 128.8, 128.6, 128.6, 128.6, 83.7, 45.8, 24.8 ppm.
IR (ATR): $\tilde{\nu}$= 1975 (m), 1670 (s), 1611 (m), 1361 (s), 1142 (s), 692 (s), 653 (s) cm^{-1}.
HRMS (EI-TOF) $[M]^+$ calcd. for $C_{20}H_{23}BO_3$: 321.1776, found: 321.1773.
The NMR match those reported in literature.[19]

2-(4-(1H-pyrrol-1-yl)phenyl)-1-phenylethan-1-one (**3ma**)

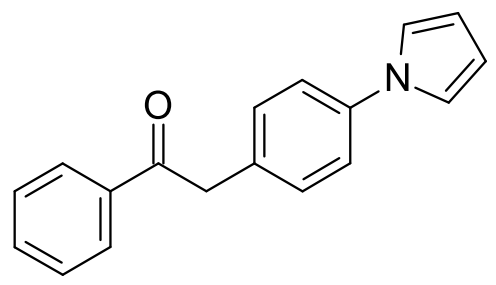

Following the general procedure **A**, 1-(4-fluorophenyl)-1*H*-pyrrole (80.6 mg, 0.5 mmol, 1 equiv.) and acetophenone (92 mg, 0.750 mmol, 1.5 equiv.) yielded the product **3ma** (86.6 mg, 66%) as white solid, purified *via* column chromatography (SiO_2, cyclohexane/EtOAc 10%).

m.p.: 166 °C
TLC: R_f = 0.38 (Cy/EA 10%)
^{1}H NMR (400 MHz, $CDCl_3$): δ = 8.03 - 8.10 (m, 2 H), 7.61 (t, *J* = 7.4 Hz, 1 H), 7.51 (t, *J* = 7.7 Hz, 2 H), 7.32 - 7.41 (m, 4 H), 7.10 (t, *J* = 2.1 Hz, 2 H), 6.37 (t, *J* = 2.1 Hz, 2 H), 4.34 (s, 2 H) ppm.
^{13}C NMR (101 MHz, $CDCl_3$): δ = 197.3, 139.6, 136.5, 133.3, 131.8, 130.6, 128.7, 128.5, 120.7, 119.3, 110.3, 44.7 ppm.
IR (ATR): $\tilde{\nu}$= 2049 (m), 1972 (m), 1687 (s), 1523 (s), 1323 (s), 1201 (s), 722 (s) 693 (s) cm^{-1}.
HRMS (EI-TOF) $[M]^+$ calcd. for $C_{18}H_{15}NO$: 261.1153, found: 261.1152.

2-(benzo[b]thiophen-6-yl)-1-phenylethan-1-one (**3na**)

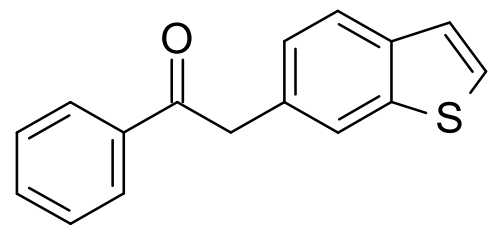

Following the general procedure **A**, 6-fluorobenzo[*b*]thiophene (76.1 mg, 0.5 mmol, 1 equiv.) and acetophenone (92 mg, 0.750 mmol, 1.5 equiv.) yielded the product **3na** (90.7 mg, 72%) as white solid, purified *via* column chromatography (SiO_2, cyclohexane/EtOAc 10%).

m.p.: 97 °C
TLC: R_f = 0.45 (Cy/EA 10%)
^{1}H NMR (400 MHz, $CDCl_3$): δ = m 8.03 - 8.09 (m, 2 H) 7.76 - 7.82 (m, 2 H) 7.53 - 7.61 (m, 1 H) 7.45 - 7.51 (m, 2 H) 7.41 (d, *J* = 5.5 Hz, 1 H) 7.27 - 7.34 (m, 2 H) 4.42 (s, 2 H) ppm.
^{13}C NMR (101 MHz, $CDCl_3$): δ = 197.5, 140.2, 138.4, 136.5, 133.2, 130.6, 128.6, 128.6, 126.2, 125.9, 123.6, 123.5, 123.0, 45.4 ppm.
IR (ATR): $\tilde{\nu}$= 2038 (m), 1688 (s), 1448 (m), 1333 (s), 1218 (s), 989 (m), 749 (s) 687 (s) cm^{-1}.
HRMS (EI-TOF) $[M]^+$ calcd. for $C_{16}H_{12}OS$: 252.0608, found: 252.0605.

1-Phenyl-2-(2-pyridinyl)ethanone (**3oa**) [CAS: 1620-53-7]

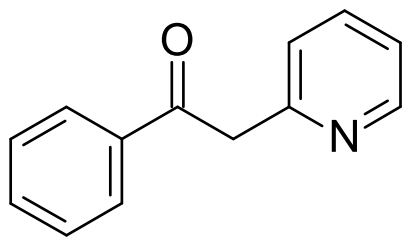

Following the general procedure **A**, 2-fluoropyridine (49.5 mg, 0.5 mmol, 1 equiv.) and acetophenone (92 mg, 0.750 mmol, 1.5 equiv.) yielded the product **3oa** (86.4 mg, 88%) as orange solid, purified *via* column chromatography (SiO_2, cyclohexane/EtOAc 20%).

m.p.: 55 °C
TLC: R_f = 0.46 (Cy/EA 20%)
^{1}H NMR (400 MHz, $CDCl_3$): δ = 8.58 (d,*J* = 4.1 Hz, 1 H), 8.05 - 8.12 (m, 2 H), 7.39 - 7.51 (m, 4 H), 7.32 (d, *J* = 7.9 Hz, 1 H), 7.19 (dd,*J* = 7.3, 5.4 Hz, 1 H), 4.51 (s, 2 H) ppm.
^{1}H NMR (400 MHz, $CDCl_3$) of the tautomer: δ =15.48 (s, 1 H), 8.31 (d, *J* = 5.1 Hz, 1 H), 7.86 (dd, *J* = 8.1, 1.6 Hz, 2 H), 7.51 - 7.71 (m, 4 H), 7.09 (d, *J* = 8.1 Hz, 1 H), 7.00 (ddd, *J* = 6.8, 5.8, 0.9 Hz, 1 H), 6.09 (s, 1 H) ppm.
^{13}C NMR (101 MHz, $CDCl_3$): δ = 196.9, 164.4, 158.6, 155.2, 149.6, 144.2, 137.1, 136.5, 136.5, 133.3, 129.3, 128.8, 128.6, 128.3, 125.5, 124.2, 121.9, 121.5, 118.5, 94.1, 48.4 ppm.
The molecule tautomerizes in solution, which is also reported in literature. H-NMRs are given separately for the tautomerized molecule. The NMR match those reported in literature.[20]

2-(2,3-difluorophenyl)-1-phenylethanone (**3pa**) [CAS: 1498370-47-0]

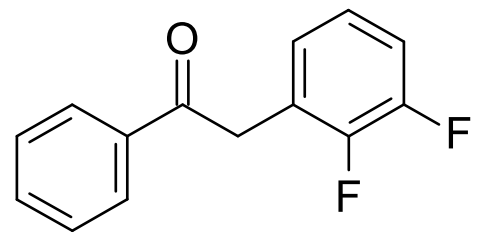

Following the general procedure **A**, 1,2,3-trifluorobenzene (66 mg, 0.5 mmol, 1 equiv.) and acetophenone (92 mg, 0.750 mmol, 1.5 equiv.) yielded the product **3pa** (82.5 mg, 71%) as a white solid, purified *via* column chromatography (SiO_2, cyclohexane/EtOAc 10%).

m.p.: 40 °C
TLC: R_f = 0.42 (Cy/EA 10%)
^{1}H NMR (400 MHz, $CDCl_3$): δ = 8.01 - 8.08 (m, 2 H), 7.59 - 7.64 (m, 1 H), 7.48 - 7.54 (m, 2 H), 6.98 - 7.15 (m, 3 H), 4.35 - 4.39 (m, 2 H) ppm.
^{13}C NMR (101 MHz, $CDCl_3$): δ = 195.5, 151.9 (dd, J=159.8, 12.5 Hz) 148.6 (dd, J=159.8, 12.5 Hz) 136.2, 133.5, 128.8, 128.4, 126.3 (t, J=3.3 Hz) 124.3 (d, J=13.1 Hz) 124.0 (dd, J=6.9, 4.7 Hz) 116.2 (d, J=16.7 Hz) 38.4 ppm.
^{19}F NMR (377 MHz, $CDCl_3$): δ = -141.95 - -141.80 (m, 1 F), -138.40 - -137.83 (m, 1 F) ppm.
IR (ATR): $\tilde{\nu}$= 1690 (s), 1595 (m), 1488 (s), 1281 (s), 1218 (s), 1018 (s), 754 (s) 685 (s) cm^{-1}.
HRMS (EI-TOF) $[M]^+$ calcd. for $C_{14}H_{10}OF_2$: 232.0699, found: 232.0689.

4-(2-Oxo-2-phenylethyl)benzonitrile (**3qa**) [CAS: 59824-23-6]

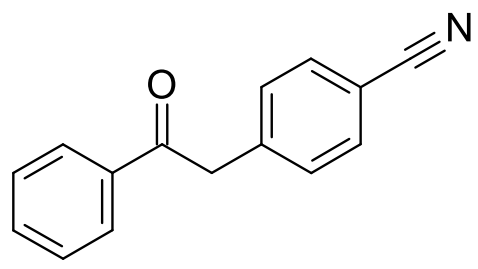

Following the general procedure **B**, 4-fluorobenzonitrile (60.6 mg, 0.5 mmol, 1 equiv.) and acetophenone (92 mg, 0.750 mmol, 1.5 equiv.) yielded the product **3qa** (76.5 mg, 69%) as white solid, purified *via* column chromatography (SiO_2, cyclohexane/EtOAc 20%).

m.p.: 111 °C
TLC: R_f = 0.36 (Cy/EA 20%)
^{1}H NMR (400 MHz, $CDCl_3$): δ = 7.98 - 8.05 (m, 2 H), 7.59 - 7.67 (m, 3 H), 7.48 - 7.54 (m, 2 H), 7.36 - 7.42 (m, 2 H), 4.37 (s, 2 H) ppm.
^{13}C NMR (101 MHz, $CDCl_3$): δ = 196.1, 139.9, 136.2, 133.7, 132.3, 130.5, 128.8, 128.4, 118.8, 111.0, 45.2 ppm.
The NMR match those reported in literature.[21]

Ethyl 4-(2-oxo-2-phenylethyl)benzoate (**3ra**) [CAS: 898776-62-0]

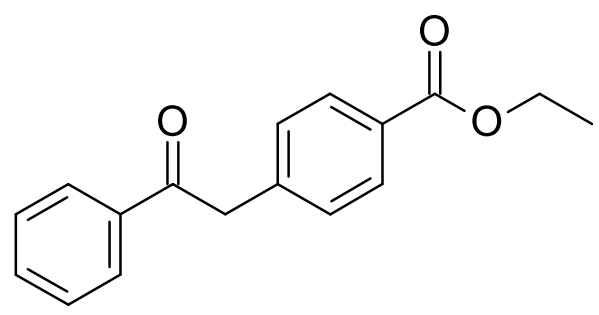

Following the general procedure **B**, ethyl-4-fluorobenzoate (84.1 mg, 0.5 mmol, 1 equiv.) and acetophenone (92 mg, 0.750 mmol, 1.5 equiv.) yielded the product **3ra** (87.9 mg, 66%) as white solid, purified *via* column chromatography (SiO_2, cyclohexane/EtOAc 20%).

m.p.: 89 °C
TLC: R_f = 0.40 (Cy/EA 20%)
^{1}H NMR (400 MHz, $CDCl_3$): δ = 7.98 - 8.06 (m, 4 H), 7.59 (t, J = 7.4 Hz, 1 H), 7.48 (t, J = 7.7 Hz, 2 H), 7.35 (d, J = 8.1 Hz, 2 H), 4.34 - 4.41 (m, 4 H), 1.39 (t, J = 7.1 Hz, 3 H) ppm.
^{13}C NMR (101 MHz, $CDCl_3$): δ = 196.8, 166.4, 139.6, 136.4, 133.4, 129.9, 129.5, 129.2, 128.7, 128.5, 60.9, 45.4, 14.3 ppm.
The NMR match those reported in literature.[22]

2-[4-[[(1R,2S,5R)-2-isopropyl-5-methyl-cyclohexoxy]methyl]cyclohexa-1,5-dien-1-yl]-1-phenyl-ethanone (**3sa**)

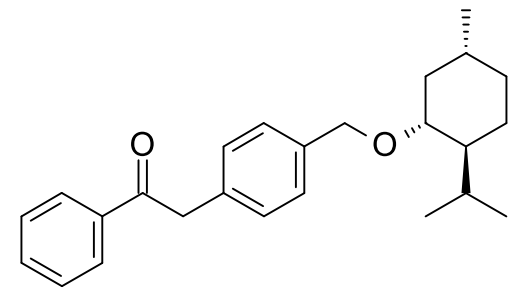

Following the general procedure **A**, 2-fluoro-5-[[(1R,2S,5R)-2-isopropyl-5-methyl-cyclohexoxy]methyl]cyclohexa-1,3-diene (66.1 mg, 0.25 mmol, 1 equiv.) and acetophenone (46 mg, 0.375 mmol, 1.5 equiv.) yielded the product **3sa** (55.8 mg, 61%) as beige solid, purified *via* column chromatography (SiO_2, cyclohexane/EtOAc 10%).

TLC: R_f = 0.41 (Cy/EA 10%)
^{1}H NMR (400 MHz, $CDCl_3$): δ = 7.99 - 8.03 (m, 2 H), 7.52 - 7.58 (m, 1 H), 7.42 - 7.49 (m, 2 H), 7.32 (d, J = 8.1 Hz, 2 H), 7.25 (d, J = 8.1 Hz, 2 H), 4.64 (d, J = 11.5 Hz, 1 H), 4.38 (d, J = 11.5 Hz, 1 H), 4.28 (s, 2 H), 3.17 (td, J = 10.6, 4.1 Hz, 1 H), 2.29 (td, J = 7.0, 2.7 Hz, 1 H), 2.14 - 2.23 (m, 1 H), 1.60 - 1.69 (m, 2 H), 1.25 - 1.41 (m, 2 H), 0.85 - 0.99 (m, 9 H), 0.70 (d, J = 7.0 Hz, 3 H) ppm.
^{13}C NMR (101 MHz, $CDCl_3$): δ = 197.6, 137.8, 136.5, 133.6, 133.1, 129.3, 128.6, 128.6, 128.2, 78.7, 70.1, 48.3, 45.3, 40.3, 34.5, 31.5, 25.5, 23.2, 22.3, 21.0, 16.0 ppm.
IR (ATR): $\tilde{\nu}$= 2954(br), 2918(br), 1687(s), 1448(s), 1083(s), 998(s), 755(s), 689(s) cm^{-1}.
HRMS (ESI-TOF) $[M + Na]^+$ calcd. for $C_{25}H_{31}NaO_2$: 387.2293, found: 387.2295.

2-[4-[[(3S,8S,9S,10R,13R,14S,17R)-17-[(1R)-1,5-dimethylhexyl]-10,13-dimethyl-2,3,4,7,8,9,11,12,14,15,16,17-dodecahydro-1H-cyclopenta[a]phenanthren-3-yl]oxymethyl]phenyl]-1-phenyl-ethanone (**3ta**)

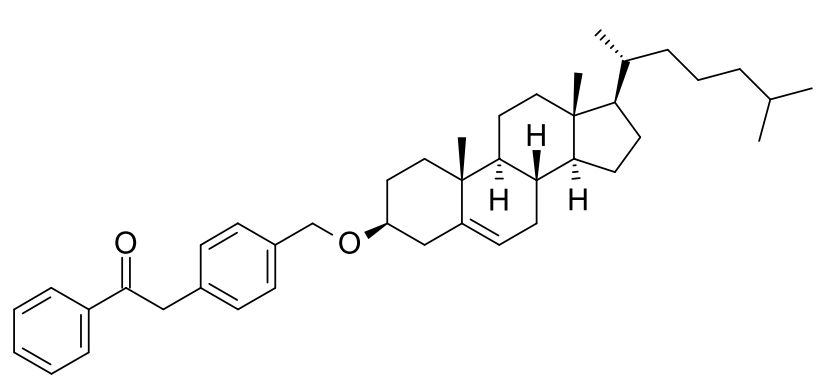

Following the general procedure **A**, (3S,8S,9S,10R,13R,14S,17R)-17-[(1R)-1,5-dimethylhexyl]-3-[(4-fluorophenyl)methoxy]-10,13-dimethyl 2,3,4,7,8,9,11,12,14,15,16,17-dodecahydro-1H-cyclopenta[a]phenanthrene (124 mg, 0.25 mmol, 1 equiv.) and acetophenone (46 mg, 0.375 mmol, 1.5 equiv.) yielded the product **3ta** (85.2 mg, 57%) as white solid, purified *via* column chromatography (SiO_2, cyclohexane/EtOAc 10%).

m.p.: 154 °C
TLC: R_f = 0.41 (Cy/EA 10%)
^{1}H NMR (400 MHz, $CDCl_3$): δ = 7.98 - 8.04 (m, 2 H), 7.53 - 7.58 (m, 1 H), 7.43 - 7.48 (m, 2 H), 7.32 (d, J = 8.0 Hz, 2 H), 7.25 (d, J = 8.1 Hz, 2 H), 5.35 (d, J = 5.1 Hz, 1 H), 4.54 (s, 2 H), 4.28 (s, 2 H), 3.23 - 3.32 (m, 1 H), 2.39 - 2.45 (m, 1 H), 2.23 - 2.31 (m, 1 H), 1.92 - 2.04 (m, 3 H), 1.79 - 1.92 (m, 2 H), 1.43 - 1.59 (m, 7 H), 1.25 - 1.41 (m, 4 H), 0.98 - 1.18 (m, 12 H), 0.86 - 0.95 (m, 10 H), 0.68 (s, 3 H) ppm.
^{13}C NMR (101 MHz, $CDCl_3$): δ = 197.6, 140.9, 137.7, 136.5, 133.6, 133.1, 129.4, 128.6, 128.6, 127.9, 121.6, 78.5, 69.6, 56.7, 56.1, 50.1, 45.3, 42.3, 39.8, 39.5, 39.1, 37.2, 36.9, 36.2, 35.8, 31.9, 31.9, 28.4, 28.2, 28.0, 24.3, 23.8, 22.8, 22.5, 21.0, 19.4, 18.7, 11.8 ppm.
IR (ATR): $\tilde{\nu}$= 2931(br), 2009 (m), 1686 (s), 1332 (s), 1084 (s), 957 (m), 755(s), 691(s) cm^{-1}.
HRMS (EI-TOF) $[M]^+$ calcd. for $C_{42}H_{58}O_2$: 594.4436, found: 594.4418.

1-phenyl-2-[4-[4-(4-propylcyclohexyl)cyclohexyl]phenyl]ethanone (**3ua**)

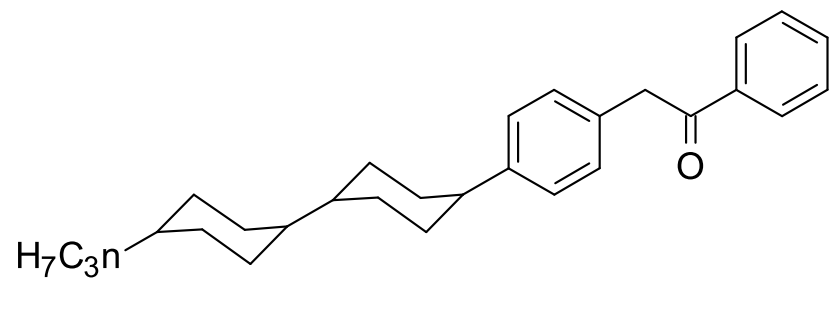

Following the general procedure **A**, 1-fluoro-4-[4-(4-propylcyclohexyl)cyclohexyl]benzene (75.6 mg, 0.25 mmol, 1 equiv.) and acetophenone (46 mg, 0.375 mmol, 1.5 equiv.) yielded the product **3ua** (78.6 mg, 78%) as white solid, purified *via* column chromatography (SiO_2, cyclohexane/EtOAc 10%).

m.p.: 145 °C
TLC: R_f = 0.38 (Cy/EA 10%)
^{1}H NMR (400 MHz, $CDCl_3$): δ = 7.98 - 8.09 (m, 2 H), 7.53 - 7.60 (m, 1 H), 7.43 - 7.51 (m, 2 H), 7.13 - 7.23 (m, 4 H), 4.26 (s, 2 H), 2.36 - 2.48 (m, 1 H), 1.71 - 1.94 (m, 8 H), 1.28 - 1.46 (m, 4 H), 1.10 - 1.20 (m, 6 H), 0.95 - 1.09 (m, 3 H), 0.82 - 0.93 (m, 5 H) ppm.
^{13}C NMR (101 MHz, $CDCl_3$): δ = 197.8, 146.5, 136.7, 133.1, 131.7, 129.3, 128.6, 128.6, 127.1, 45.1, 44.3, 43.4, 42.9, 39.8, 37.6, 34.6, 33.6, 30.3, 30.1, 20.0, 14.4 ppm.
IR (ATR): $\tilde{\nu}$= 2912 (s), 2847 (s), 1686 (s), 1449 (s), 1336 (s), 1197 (s), 757 (s) 691 (s) cm^{-1}.
HRMS (EI-TOF) $[M]^+$ calcd. for $C_{29}H_{38}O$: 402.2923, found: 402.2922.

2-[4-(4-morpholinyl)phenyl]-1-phenyl-]ethanone (**3va**) [CAS: 884504-56-7]

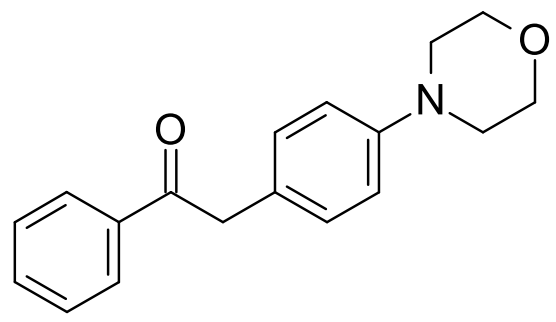

Following the general procedure **A**, 4-(4-fluorophenyl)morpholine (90.6 mg, 0.5 mmol, 1 equiv.) and acetophenone (92 mg, 0.75 mmol, 1.5 equiv.) yielded the product **3va** (100.5 mg, 71%) as white solid, purified *via* column chromatography (SiO_2, cyclohexane/EtOAc 20%).

m.p.: 158 °C
TLC: R_f = 0.36 (Cy/EA 20%)
^{1}H NMR (400 MHz, $CDCl_3$): δ = 7.97 - 8.06 (m, 2 H), 7.51 - 7.62 (m, 1 H), 7.38 - 7.51 (m, 2 H), 7.14 - 7.23 (m, 2 H), 6.86 - 6.92 (m, 2 H), 4.22 (s, 2 H), 3.82 - 3.88 (m, 4 H), 3.09 - 3.17 (m, 4 H) ppm.
^{13}C NMR (75 MHz, $CDCl_3$): δ = 198.0, 150.1, 136.6, 133.0, 130.2, 128.6, 128.6, 125.8, 115.9, 66.9, 49.3, 44.6 ppm.
IR (ATR): $\tilde{\nu}$= 2952 (m), 2160 (m), 1689 (s), 1514 (m), 1119 (s), 926 (s), 765 (s) 693 (s) cm^{-1}.
HRMS (EI-TOF) $[M]^+$ calcd. for $C_{18}H_{19}NO_2$: 281.1415, found: 281.1413.

2-[1,1'-Biphenyl]-4-yl-1-phenylethanone (**3wa**) [CAS: 27644-00-4]

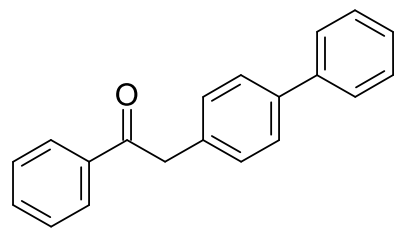

Following the general procedure **A**, 4-fluorobiphenyl (86.1 mg, 0.5 mmol, 1 equiv.) and acetophenone (92 mg, 0.75 mmol, 1.5 equiv.) yielded the product **3wa** (119.5 mg, 88%) as white solid, purified *via* column chromatography (SiO_2, cyclohexane/EtOAc 10%).

TLC: R_f = 0.32 (Cy/EA 10%)
^{1}H NMR (400 MHz, $CDCl_3$): δ = 8.05 - 8.13(m, 2 H), 7.57 – 7.65 (m, 5 H), 7.43 – 7.54 (m, 4 H), 7.34 – 7.42 (m, 3 H), 4.37 (s, 2 H) ppm.
^{13}C NMR (75 MHz, $CDCl_3$): δ = 197.6, 140.8, 139.9, 136.6, 133.5, 133.2, 129.9, 128.73, 128.67, 128.61, 127.4, 127.2, 127.1, 45.1 ppm.
The NMR match those reported in literature.[23]

1,2-Bis(4-methoxyphenyl)ethanone (**3ab**) [CAS: 120-44-5]

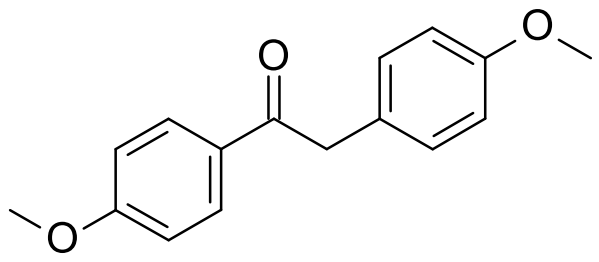

Following the general procedure **A**, 4-fluoroanisole (63.1 mg, 0.5 mmol, 1 equiv.) and 4-methoxyacetophenone (114 mg, 0.750 mmol, 1.5 equiv.) yielded the product **3ab** (122.8 mg, 96%) as white solid, purified *via* column chromatography (SiO_2, cyclohexane/EtOAc 20%).

m.p.: 110 °C
TLC: R_f = 0.32 (Cy/EA 20%)
^{1}H NMR (400 MHz, $CDCl_3$): δ = 7.97 - 8.05 (m, 2 H), 7.20 (m, 2 H), 6.91 - 6.98 (m, 2 H), 6.83 - 6.90 (m, 2 H), 4.18 (s, 2 H), 3.85 (s, 3 H), 3.78 (s, 3 H) ppm.
^{13}C NMR (101 MHz, $CDCl_3$): δ = 196.4, 163.4, 158.4, 130.8, 130.3, 129.5, 126.9, 114.0, 113.7, 55.3, 55.1, 44.2 ppm.
The NMR match those reported in literature.[10]

1-[4-(Dimethylamino)phenyl]-2-(4-methoxyphenyl)ethanone (**3ac**) [CAS: 97606-30-9]

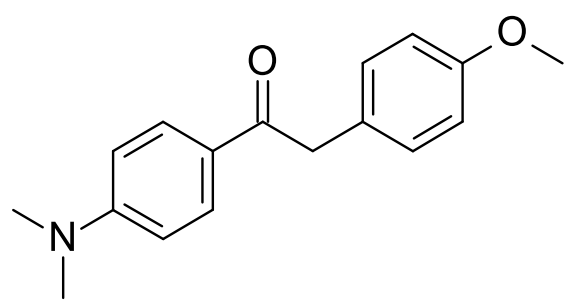

Following the general procedure **A**, 4-fluoroanisol (63.1 mg, 0.5 mmol, 1 equiv.) and 4-dimethylaminoacetophenone (126 mg, 0.750 mmol, 1.5 equiv.) yielded the product **3ac** (119.3 mg, 89%) as white solid, purified *via* column chromatography (SiO_2, cyclohexane/EtOAc 20%).

TLC: R_f = 0.31 (Cy/EA 20%)
^{1}H NMR (400 MHz, $CDCl_3$): δ = 7.94 (d,J = 9.0 Hz, 2 H), 7.21 (d,J = 8.6 Hz, 2 H), 6.86 (d,J = 8.6 Hz, 2 H), 6.65 (d, J = 9.0 Hz, 2 H), 4.14 (s, 2 H), 3.78 (s, 3 H), 3.04 (s, 6 H) ppm.
^{13}C NMR (101 MHz, $CDCl_3$): δ = 196.0, 158.2, 153.3, 130.8, 130.2, 127.7, 124.4, 113.9, 110.6, 55.1, 43.9, 39.9 ppm.
The NMR match those reported in literature.[24]

2-(4-Methoxyphenyl)-1-(2-thienyl)ethanone (**3ad**) [CAS: 67947-62-0]

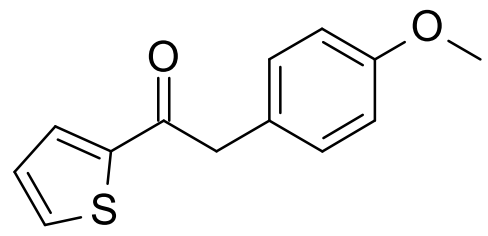

Following the general procedure **A**, 4-fluoroanisole (63.1 mg, 0.5 mmol, 1 equiv.) and 3-acetylthiophene (96.6 mg, 0.750 mmol, 1.5 equiv.) yielded the product **3ad** (57.1 mg, 49%) as white solid, purified *via* column chromatography (SiO_2, cyclohexane/EtOAc 10%).

m.p.: 79 °C
TLC: R_f = 0.29 (Cy/EA 10%)
^{1}H NMR (400 MHz, $CDCl_3$): δ = 8.06 - 8.13 (m, 1 H), 7.55 - 7.59 (m, 1 H), 7.31 (dd, J = 5.1, 2.9 Hz, 1 H), 7.18 - 7.22 (m, 2 H), 6.85 - 6.90 (m, 2 H), 4.12 (s, 2 H), 3.80 (s, 3 H) ppm.
^{13}C NMR (101 MHz, $CDCl_3$): δ = 192.2, 158.6, 141.8, 132.5, 130.4, 127.3, 126.4, 126.3, 114.1, 55.2, 46.0 ppm.
The NMR match those reported in literature.[25]

2-(4-Methoxyphenyl)-1-(4-methylphenyl)ethanone (**3ae**) [CAS: 345617-51-8]

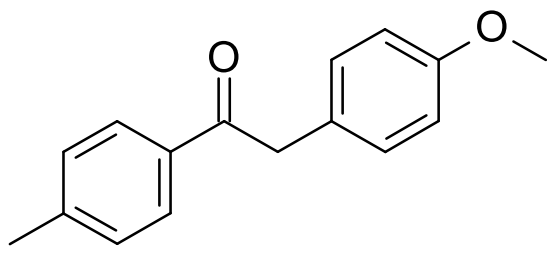

Following the general procedure **A**, 4-fluoroanisole (63.1 mg, 0.5 mmol, 1 equiv.) and 4-methylacetophenone (106 mg, 0.750 mmol, 1.5 equiv.) yielded the product **3ae** (102.5 mg, 85%) as white solid, purified *via* column chromatography (SiO_2, cyclohexane/EtOAc 10%).

m.p.: 99 °C
TLC: R_f = 0.30 (Cy/EA 10%)
^{1}H NMR (400 MHz, $CDCl_3$): δ = 7.91 (d, J = 8.3 Hz, 2 H), 7.25 (d, J = 7.9 Hz, 2 H), 7.18 (d, J = 8.6 Hz, 2 H), 6.83 - 6.89 (m, 2 H), 4.19 (s, 2 H), 3.77 (s, 3 H), 2.40 (s, 3 H) ppm.
^{13}C NMR (75 MHz, $CDCl_3$): δ = 197.5, 158.4, 143.8, 134.1, 130.3, 129.2, 128.7, 126.7, 114.0, 55.1, 44.4, 21.5 ppm.
The NMR match those reported in literature.[10]

2-(4-Methoxyphenyl)-1-phenyl-1-propanone (**3af**) [CAS: 84839-92-9]

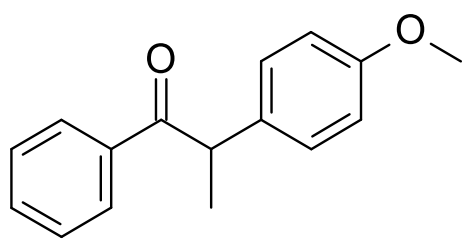

Following the general procedure **A** at 40°C, 4-fluoroanisol (63.1 mg, 0.5 mmol, 1 equiv.) and propiophenone (101 mg, 0.750 mmol, 1.5 equiv.) yielded the product **3af** (74.6 mg, 62%) as white solid, purified *via* column chromatography (SiO_2, cyclohexane/EtOAc 10%).

TLC: R_f = 0.55 (Cy/EA 10%)
^{1}H NMR (400 MHz, $CDCl_3$): δ = 7.91 - 8.01 (m, 2 H), 7.44 - 7.53 (m, 1 H), 7.39 (t, J = 7.6 Hz, 2 H), 7.17 - 7.25 (m, 2 H), 6.79 - 6.89 (m, 2 H), 4.65 (q, J = 6.8 Hz, 1 H), 3.76 (s, 3 H), 1.52 (d, J = 6.9 Hz, 3 H) ppm.
^{13}C NMR (101 MHz, $CDCl_3$): δ = 200.5, 158.5, 136.5, 133.5, 132.7, 128.8, 128.7, 128.4, 114.4, 55.2, 47.0, 19.5 ppm.
The NMR match those reported in literature.[26]

2-(4-Methoxyphenyl)-1-(2-naphthalenyl)ethanone (**3ag**) [CAS: 1184749-94-7]

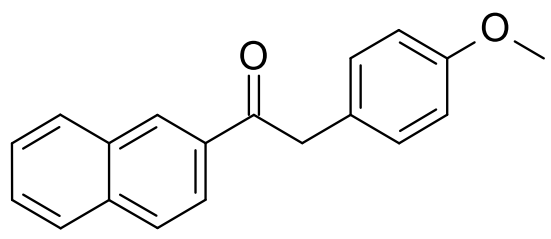

Following the general procedure **A**, 4-fluoroanisol (63.1 mg, 0.5 mmol, 1 equiv.) and 2'-Acetonaphtone (129 mg, 0.750 mmol, 1.5 equiv.) yielded the product **3ag** (124.1 mg, 90%) as white solid, purified *via* column chromatography (SiO_2, cyclohexane/EtOAc 10%).

m.p.: 74 °C
TLC: R_f = 0.28 (Cy/EA 10%)
^{1}H NMR (400 MHz, $CDCl_3$): δ = 8.55 (s, 1 H), 8.07 (dd, J = 8.6, 1.8 Hz, 1 H), 7.96 (d, J = 8.0 Hz, 1 H), 7.82 - 7.91 (m, 2 H), 7.52 - 7.62 (m, 2 H), 7.23 - 7.28 (m, 2 H), 6.82 - 6.96 (m, 2 H), 4.35 (s, 2 H), 3.78 (s, 3 H) ppm.
^{13}C NMR (101 MHz, $CDCl_3$): δ = 197.8, 158.4, 135.4, 133.9, 132.4, 130.4, 130.2, 129.5, 128.4, 128.4, 127.7, 126.7, 126.5, 124.2, 114.1, 77.3, 76.7, 55.1, 44.5 ppm.
The NMR match those reported in literature.[27]

2-(4-Methoxyphenyl)cyclohexanone (**3ah**) [CAS: 37087-68-6]

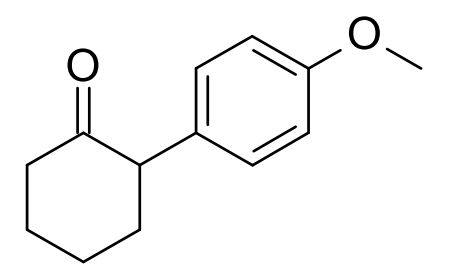

Following the general procedure **A** at 60°C, 4-fluoroanisole (63.1 mg, 0.5 mmol, 1 equiv.) and cyclohexanone (74.4 mg, 0.750 mmol, 1.5 equiv.) yielded the product **3ah** (44.0 mg, 43%) as beige solid, purified *via* column chromatography (SiO_2, cyclohexane/EtOAc 20%).

m.p.: 85 °C
TLC: R_f = 0.34 (Cy/EA 20%)
^{1}H NMR (400 MHz, $CDCl_3$): δ = 7.04 - 7.10 (m, 2 H), 6.86 - 6.92 (m, 2 H), 3.78 - 3.83 (m, 3 H), 3.58 (dd, J = 12.3, 5.4 Hz, 1 H), 2.43 - 2.57 (m, 2 H), 2.22 - 2.31 (m, 1 H), 2.11 - 2.20 (m, 1 H), 1.95 - 2.06 (m, 2 H), 1.78 - 1.89 (m, 2 H) ppm.
^{13}C NMR (101 MHz, $CDCl_3$): δ = 210.7, 158.4, 130.8, 129.4, 113.8, 56.5, 55.2, 42.2, 35.3, 27.8, 25.4 ppm.
The NMR match those reported in literature.[28]

4-Methoxy-α-phenylbenzeneacetonitrile (**3ai**) [CAS: 4578-79-4]

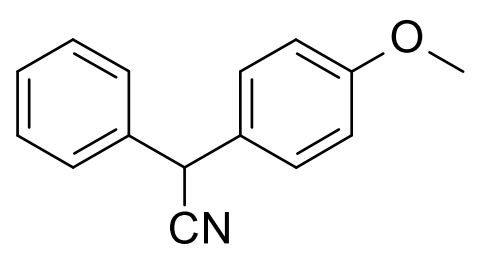

Following the general procedure **A** with ^{t}BuBrettPhos as ligand at 40°C, 4-fluoroanisole (63.1 mg, 0.5 mmol, 1 equiv.) and benzyl cyanide (87.9 mg, 0.750 mmol, 1.5 equiv.) yielded the product **3ai** (79.1 mg, 71%) as white solid, purified *via* column chromatography (SiO_2, cyclohexane/EtOAc 10%).

m.p.: 160 °C
TLC: R_f = 0.39 (Cy/EA 10%)
^{1}H NMR (400 MHz, $CDCl_3$): δ = 7.21 - 7.33 (m, 5 H), 7.16 - 7.19 (m, 2 H), 6.79 - 6.85 (m, 2 H), 5.02 (s, 1 H), 3.73 (s, 3 H) ppm.
^{13}C NMR (101 MHz, $CDCl_3$): δ = 159.4, 136.2, 129.1, 128.9, 128.1, 127.9, 127.6, 119.9, 114.5, 55.3, 41.8 ppm.
The NMR match those reported in literature.[29]

N,N-Diethyl-4-methoxybenzeneacetamide (**3aj**) [CAS: 115348-15-7]

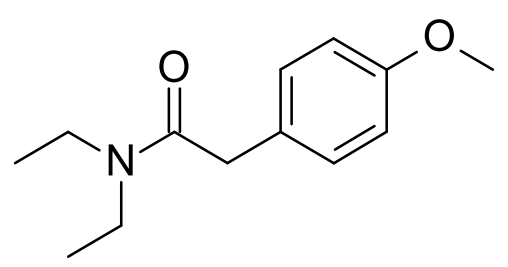

Following the general procedure **A** with ^{t}BuBrettPhos as ligand**,** 4-fluoroanisole (63.1 mg, 0.5 mmol, 1 equiv.) and *N,N*-diethylacetamide (87.3 mg, 0.75 mmol, 1.5 equiv.) yielded the product **3aj** (63.1 mg, 57%) as brown oil, purified *via* column chromatography (SiO_2, cyclohexane/EtOAc 40%).

TLC: R_f = 0.42 (Cy/EA 40%)
^{1}H NMR (400 MHz, $CDCl_3$): δ = 7.15 - 7.22 (m, 2 H), 6.82 - 6.90 (m, 2 H), 3.80 (s, 3 H), 3.65 (s, 2 H), 3.39 (q, J = 7.0 Hz, 2 H), 3.31 (q, J = 7.2 Hz, 2 H), 1.08 - 1.15 (m, 6 H) ppm.
^{13}C NMR (101 MHz, $CDCl_3$): δ = 170.5, 158.4, 129.6, 127.5, 114.0, 55.2, 42.3, 40.2, 39.9, 14.2, 12.9 ppm.
The NMR match those reported in literature.[30]

1,1-Dimethylethyl 4-methoxybenzeneacetate (**3ak**) [CAS: 33155-61-2]

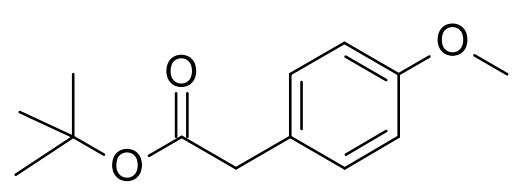

Following the general procedure **A**, 4-fluoroanisole (63.1 mg, 0.5 mmol, 1 equiv.) and tert-butyl acetate (88 mg, 0.750 mmol, 1.5 equiv.) yielded the product **3ak** (75.4 mg, 68%) as clear oil, purified *via* column chromatography (SiO_2, cyclohexane/EtOAc 10%).

TLC: R_f = 0.61 (Cy/EA 10%)
^{1}H NMR (400 MHz, $CDCl_3$): δ = 7.19 (d, J = 8.6 Hz, 2 H), 6.83 - 6.90 (m, 2 H), 3.80 (s, 3 H), 3.47 (s, 2 H), 1.44 (s, 9 H) ppm.
^{13}C NMR (101 MHz, $CDCl_3$): δ = 171.3, 158.5, 130.2, 126.8, 113.9, 80.6, 55.2, 41.7, 28.0 ppm.
The NMR match those reported in literature.[31]

1-fluoro-4-[[(1R,2S,5R)-2-isopropyl-5-methyl-cyclohexoxy]methyl]benzene (**1s**) [CAS: 3060493-85-5]

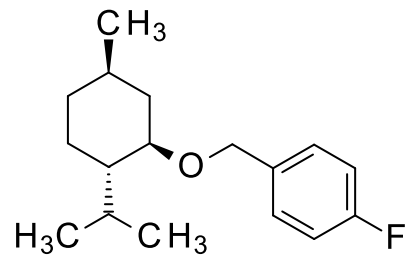

Following procedure **D**, L(-)-Menthol (789 mg, 5 mmol, 1 equiv.) yielded the product **1s** (150 mg, 11%) as white solid, purified *via* column chromatography (SiO_2, cyclohexane/EtOAc 10%).

TLC: R_f = 0.29 (Cyclohexane)
^{1}H NMR (400 MHz, $CDCl_3$): δ = 7.30 - 7.34 (m, 2 H), 7.00 - 7.06 (m, 2 H), 4.63 (d, J = 11.4 Hz, 1 H), 4.37 (d, J = 11.3 Hz, 1 H), 3.17 (td, J = 10.5, 4.2 Hz, 1 H), 2.13 - 2.34 (m, 2 H), 1.58 - 1.73 (m, 2 H), 1.23 - 1.44 (m, 2 H), 0.84 - 1.00 (m, 9 H), 0.72 (d, J = 7.0 Hz, 3 H) ppm.
^{13}C NMR (101 MHz, $CDCl_3$): δ = 162.2 (d, J = 244.9 Hz) 134.9 (d, J = 3.6 Hz) 129.5 (d, J = 8.0 Hz) 115.1 (d, J = 21.1 Hz) 78.7, 69.7, 48.3, 40.3, 34.5, 31.6, 25.5, 23.2, 22.4, 21.0, 16.0 ppm.
^{19}F NMR (377 MHz, $CDCl_3$): δ = = -115.27 - -115.41 (m, 1F) ppm.
The NMR match those reported in literature.[2]

(3S,8S,9S,10R,13R,14S,17R)-17-[(1R)-1,5-dimethylhexyl]-3-[(4-fluorophenyl)methoxy]-10,13-dimethyl-2,3,4,7,8,9,11,12,14,15,16,17-dodecahydro-1H-cyclopenta[a]phenanthrene (**1t**) [CAS: 3060493-87-7]

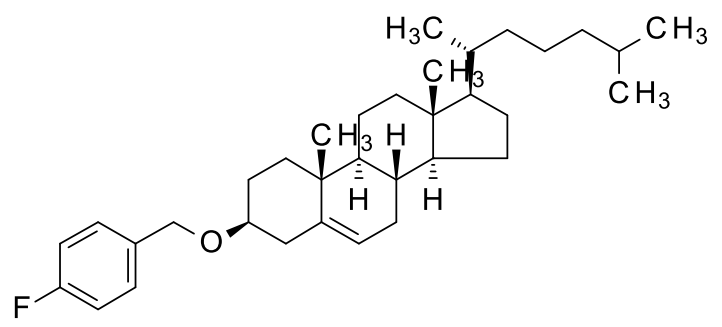

Following procedure **D**, Cholesterol (1953 mg, 5 mmol, 1 equiv.) yielded the product **1t** (613 mg, 25%) as white solid, purified *via* column chromatography (SiO_2, cyclohexane/EtOAc 10%).

TLC: R_f = 0.58 (Cy/EA 5%)
^{1}H NMR (400 MHz, $CDCl_3$): δ = 7.29 - 7.35 (m, 2 H), 7.02 (tt, J = 8.6, 2.5 Hz, 2 H), 5.34 - 5.38 (m, 1 H), 4.51 - 4.55 (m, 2 H), 3.21 - 3.33 (m, 1 H), 2.37 - 2.47 (m, 1 H), 2.22 - 2.34 (m, 1 H), 1.92 - 2.06 (m, 3 H), 1.77 - 1.91 (m, 2 H), 1.44 - 1.64 (m, 7 H), 1.26 - 1.43 (m, 4 H), 0.98 - 1.22 (m, 12 H), 0.93 (d, J = 6.5 Hz, 4 H), 0.88 (dd, J = 6.6, 1.8 Hz, 6 H), 0.69 (s, 3 H) ppm.
^{13}C NMR (101 MHz, $CDCl_3$): δ = 162.4 (d, J = 244.9 Hz), 140.9, 134.8 (d, J = 2.9 Hz), 129.3 (d, J = 8.0 Hz), 121.7, 115.2 (d, J = 21.1 Hz), 78.7, 69.2, 56.8, 56.1, 50.2, 42.3, 39.8, 39.5, 39.1, 37.2, 36.9, 36.2, 35.8, 31.9, 31.9, 28.4, 28.2, 28.0, 24.3, 23.8, 22.8, 22.6, 21.1, 19.4, 18.7, 11.9 ppm.
^{19}F NMR (377 MHz, $CDCl_3$): δ = -115.26 - -115.40 (m, 1F) ppm.
The NMR match those reported in literature.[32]

4-(4-fluorophenyl)morpholine (**1v**) [CAS: 4280-40-4]

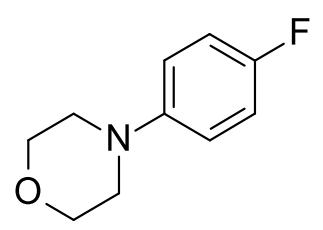

Inspired by literature-reported procedure for this Buchwald-Hartwig amination reaction of similar substrates[33]: An oven-dried vial (20 mL) was charged with $Pd(OAc)_2$ (0.04 mmol, 8.9 mg, 2 mol%), JohnPhos (0.04 mmol, 11.9 mg, 2 mol%), and KO^{t}Bu (2.8 mmol, 321 mg, 1.4 equiv.). The vial was purged in three alternating vacuum/nitrogen cycles and 1 mL of toluene was added. 1 mL of a stock solution of 4-bromofluorobenzene (2 mmol, 1.0 equiv.), morpholine (2.4 mmol, 1.2 equiv.) and internal standard in toluene, was added. The resulting mixture was stirred at 30 °C for 16 h. After completion of the reaction, the mixture was quenched with NH_4Cl, diluted with EtOAc and washed with $NaHCO_3$, water and brine. The combined organic phase was dried over $MgSO_4$ and purified by flash column chromatography (SiO_2, Cy/EtOAc) to yield the product **1v** as a beige solid (264.2 mg, 73 %).

TLC: R_f = 0.32 (Cy/EA 20%)
^{1}H NMR (400 MHz, $CDCl_3$): δ = 6.94 - 7.04 (m, 2 H), 6.83 - 6.91 (m, 2 H), 3.84 - 3.89 (m, 4 H), 3.07 - 3.12 (m, 4 H) ppm.
^{13}C NMR (101 MHz, $CDCl_3$): δ = 157.3 (d, J = 239.0 Hz) 147.9 (d, J = 2.1 Hz) 117.4 (d, J = 8.0 Hz) 115.6 (d, J = 21.8 Hz) 66.9 (s) 50.3 (s) ppm.
^{19}F NMR (377 MHz, $CDCl_3$): δ = -124.2 (spt, J = 4.1 Hz, 1F) ppm.
The NMR match those reported in literature.[34]

1-fluoro-4-phenyl-benzene (**1w**) [CAS: 324-74-3]

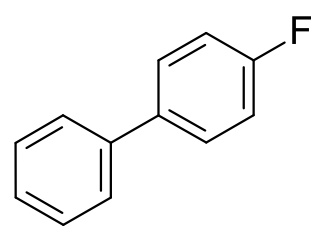

Inspired by literature-reported procedure for this Suzuki coupling reaction of similar substrates:[35] In an oven-dried vial, $Pd(OAc)_2$ (0.025 mmol, 5.61 mg, 1 mol%), 2-(Dicyclohexylphosphino)biphenyl (0.05 mmol, 17.5 mg, 2 mol%), Phenylboronic acid (3.75 mmol, 467 mg, 1.5 equiv.) and K_3PO_4 (5 mmol, 1083 mg, 2 equiv.) were added and evacuated under a vacuum/nitrogen cycle. 7.5 mL of a stock solution of 1-Chloro-4-fluorobenzene (2.5 mmol, 326 mg, 1 equiv.) and internal standard in toluene were added. The reaction mixture was stirred at 80 °C for 12 hours. After completion (monitored by GC), the reaction mixture was cooled to room temperature, quenched with NaOH, diluted with EtOAc and washed with NaOH, water and brine. The combined organic phase was dried over MgSO4 and purified by flash column chromatography (SiO2, Cy/EtOAc) to yield the product **1w** as a white solid (402.1 mg, 93 %).

TLC: R_f = 0.38 (Cyclohexane)
^{1}H NMR (400 MHz, $CDCl_3$): *δ* = 7.55 - 7.62 (m, 4 H), 7.48 (t, *J*=7.6 Hz, 2 H), 7.39 (t, *J*=7.0 Hz, 1 H), 7.17 (t, *J*=8.6 Hz, 2 H) ppm.
^{13}C NMR (101 MHz, $CDCl_3$): *δ* = 162.3 (d, *J* = 245.5 Hz), 140.2, 137.3 (d, *J* = 3.0 Hz), 128.8, 128.7 (d, *J* = 8.1 Hz), 127.2, 127.0, 115.6 (d, *J* = 21.1 Hz) ppm.
^{19}F NMR (76 MHz, $CDCl_3$): *δ* = -113.8 (spt, *J*= 4.7 Hz, 1F) ppm.
The NMR match those reported in literature.[36]

Synthesis of $[Pd(1\text{-}MeNAP)TFA]_2$

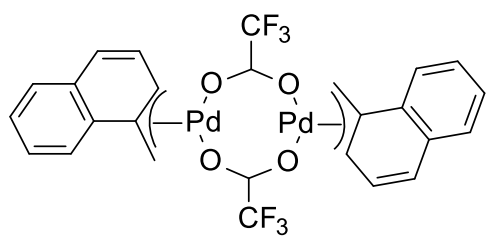

Adapted from literature-reported procedure:[37] An oven-dried vial (20 mL) was charged with $[Pd(1\text{-}MeNAP)Br]_2$ (1 equiv.) and purged with three alternating vacuum/nitrogen cycles. In the glovebox, silver trifluoroacetic acid (2 equiv.) was added, together with 10 mL of dry THF. The mixture was stirred in the dark for 1 hour. Then, the catalyst solution was filtered with syringe filters and the solvent was removed under reduced pressure. With only 1/10 of solvent remaining in the vial, 10 mL of dry pentane was added, and the precipitate was washed with pentane three times. Removing excess solvent under reduced pressure afforded the $[Pd(1\text{-}MeNAP)TFA]_2$ precatalyst as a bright yellow powder.

^{1}H NMR (400 MHz, DMSO-d_6): δ = 8.35 (d,*J* = 8.4 Hz, 2 H), 7.94 (t, *J* = 7.1 Hz, 4 H), 7.54 - 7.67 (m, 4 H), 7.29 - 7.35 (m, 2 H), 7.22 - 7.27 (m, 2 H), 3.79 (s, 4 H)
^{13}C NMR (101 MHz, DMSO-d_6): *δ* = 159.4 (q, *J* = 34.1 Hz) 137.6 (s) 133.9 (s) 131.0 (s) 128.2 (s) 126.9 (s) 126.8 (s) 126.6 (s) 125.9 (s) 125.1 (s) 119.0 (s) 116.4 (q, *J* = 294.1 Hz) 30.6 (s) ppm.
^{19}F NMR (76 MHz, DMSO-d_6): δ = -73.5 ppm.
The NMR match those reported in literature.[37]

NMR-Spectra of Products

2-(4-methoxyphenyl)-1-phenylethanone (**3aa**) [CAS: 24845-40-7]

^{1}H NMR (400 MHz, $CDCl_3$)

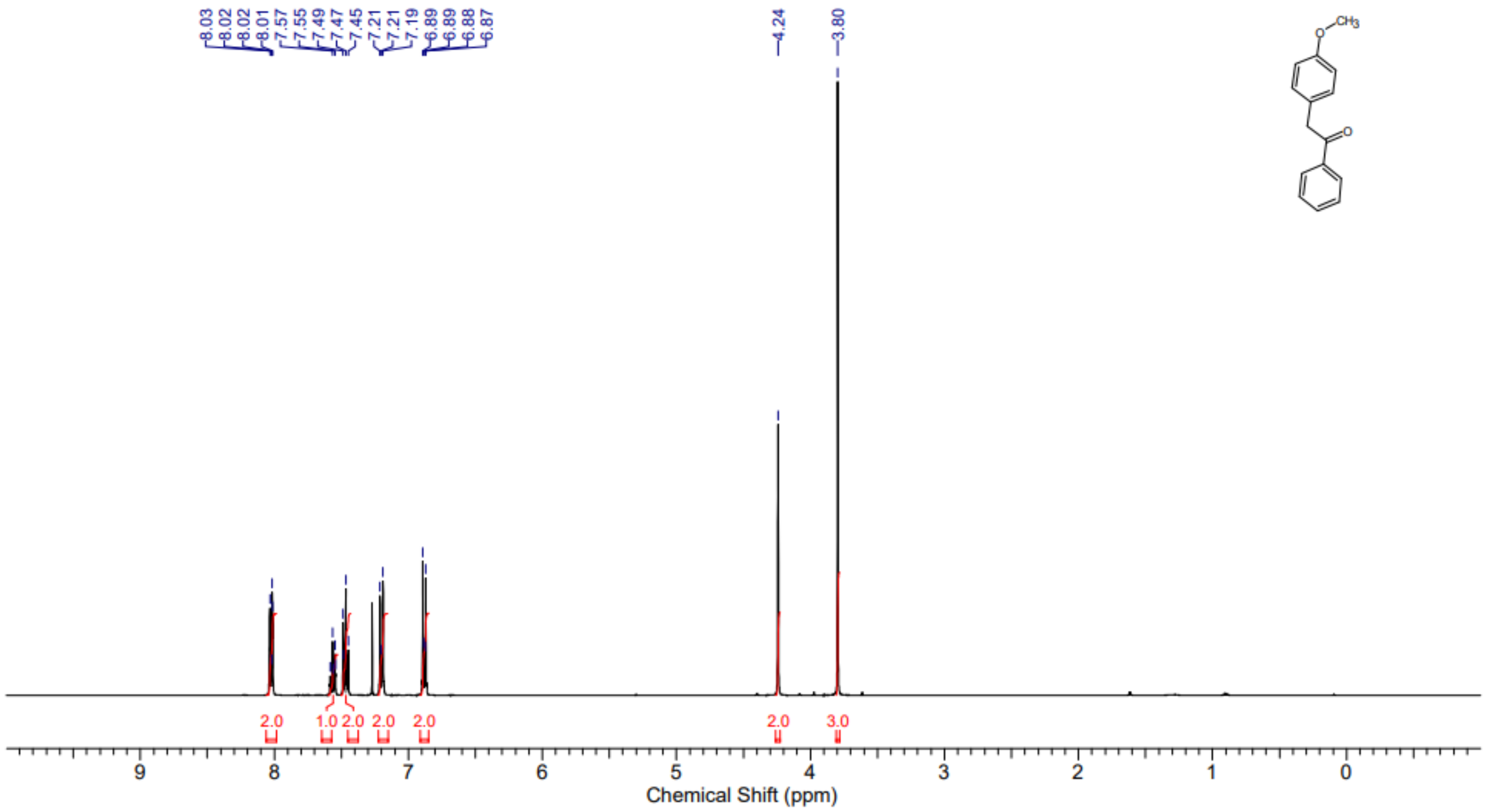

^{13}C NMR (101 MHz, $CDCl_3$)

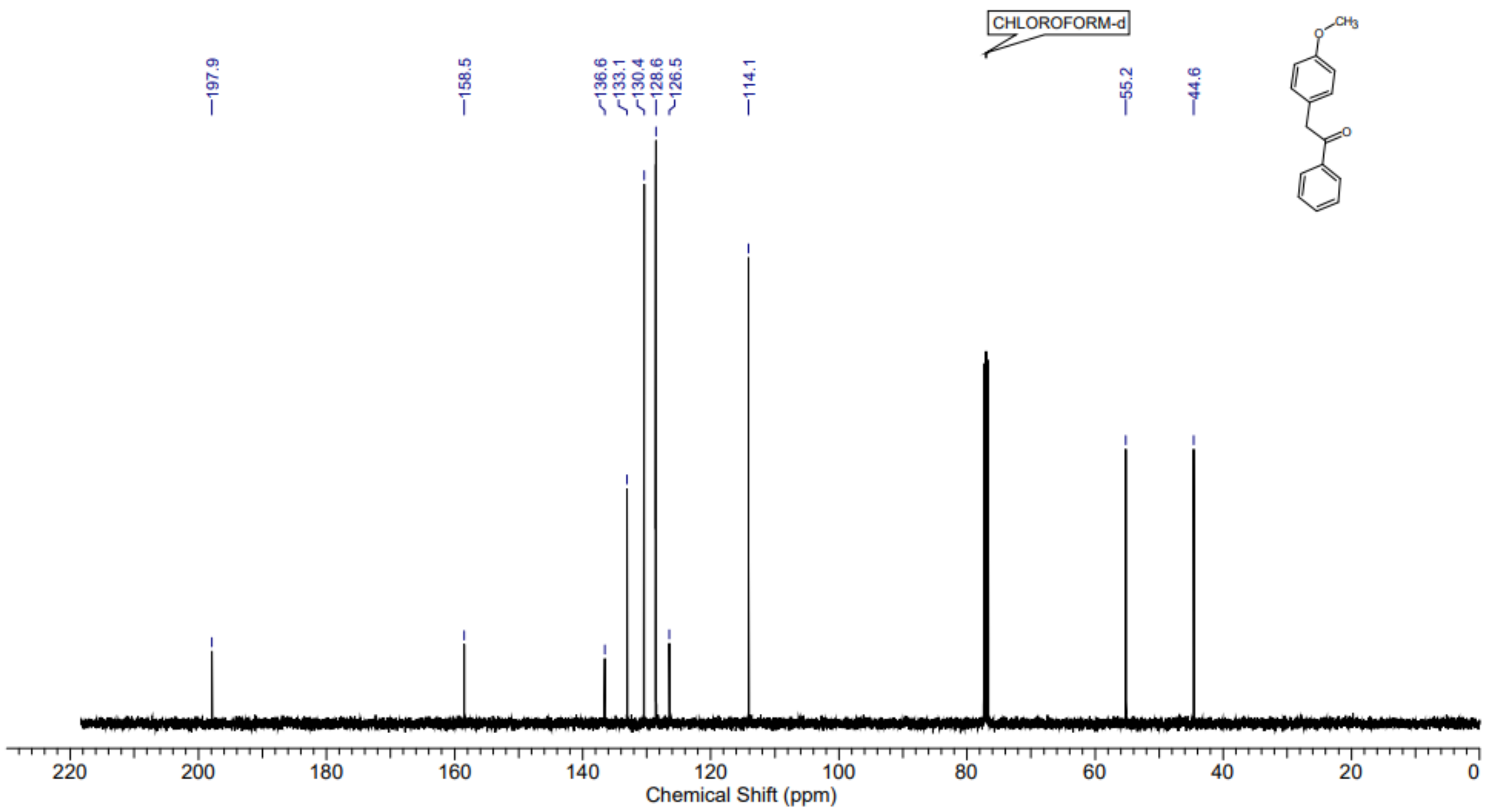

2-(4-Methylphenyl)-1-phenylethanone (**3ba**) [CAS: 2430-99-1]

^{1}H NMR (400 MHz, $CDCl_3$)

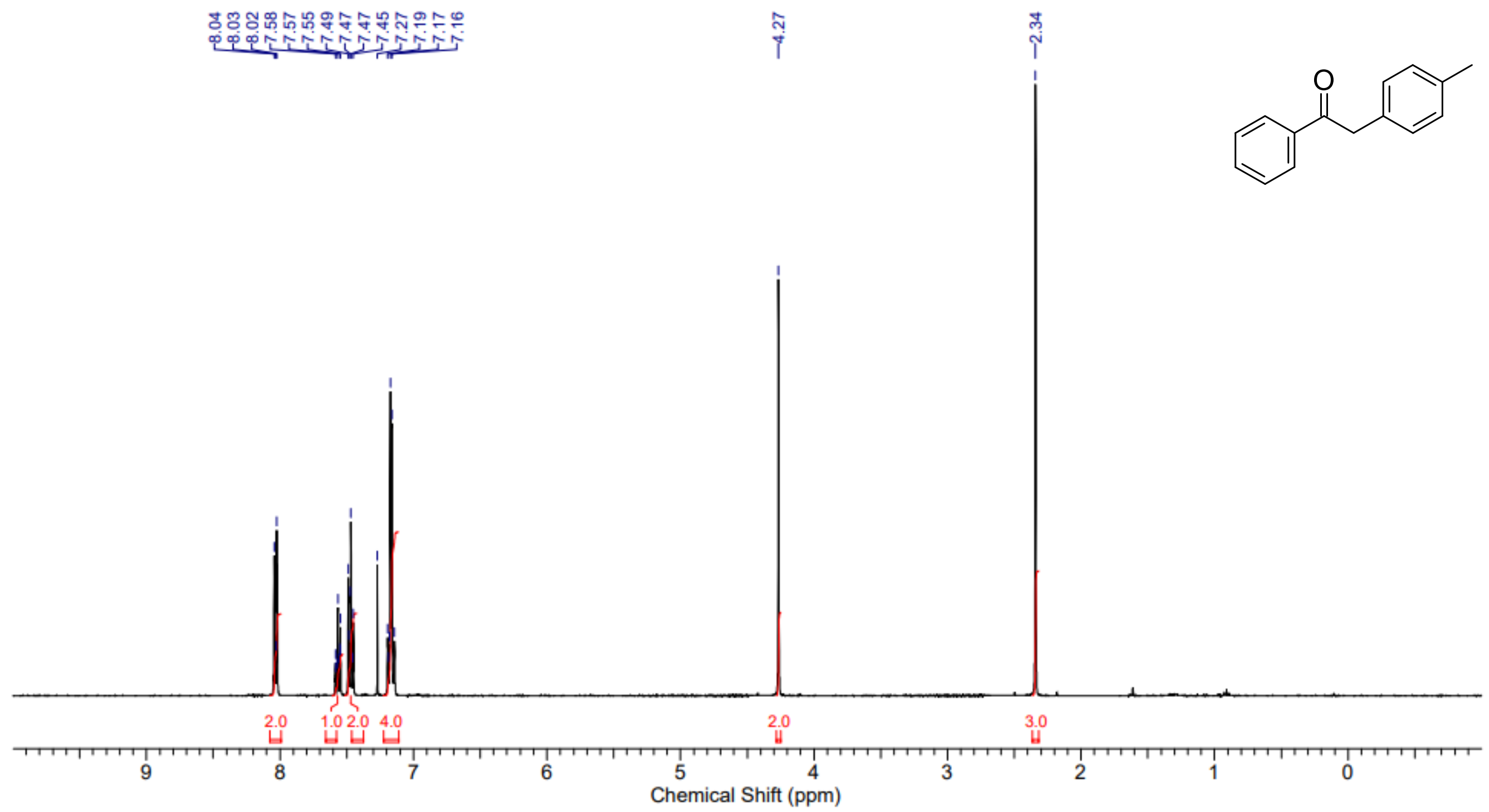

^{13}C NMR (101 MHz, $CDCl_3$)

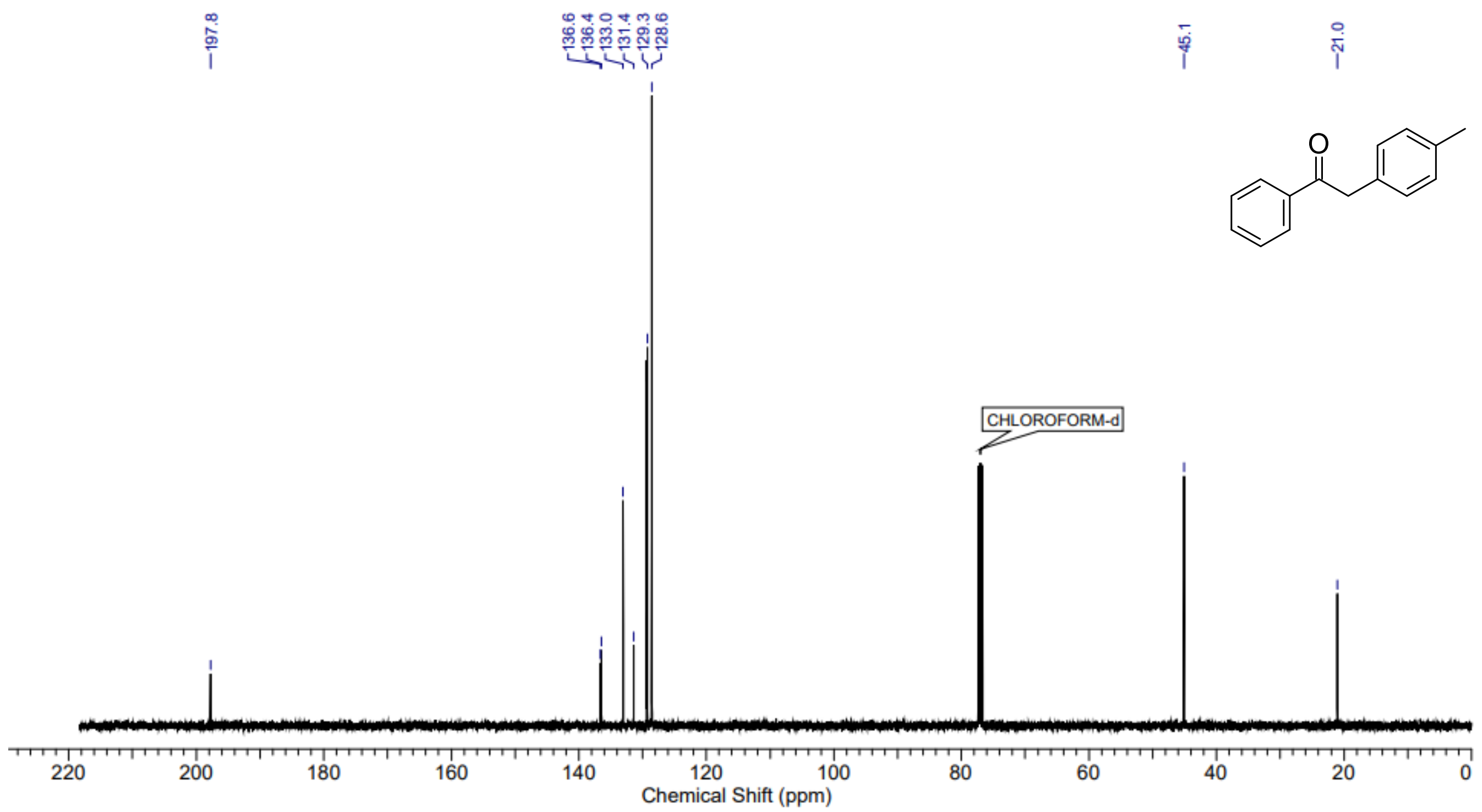

1,2-diphenylethanone (**3ca**) [CAS: 451-40-1]

^{1}H NMR (400 MHz, $CDCl_3$)

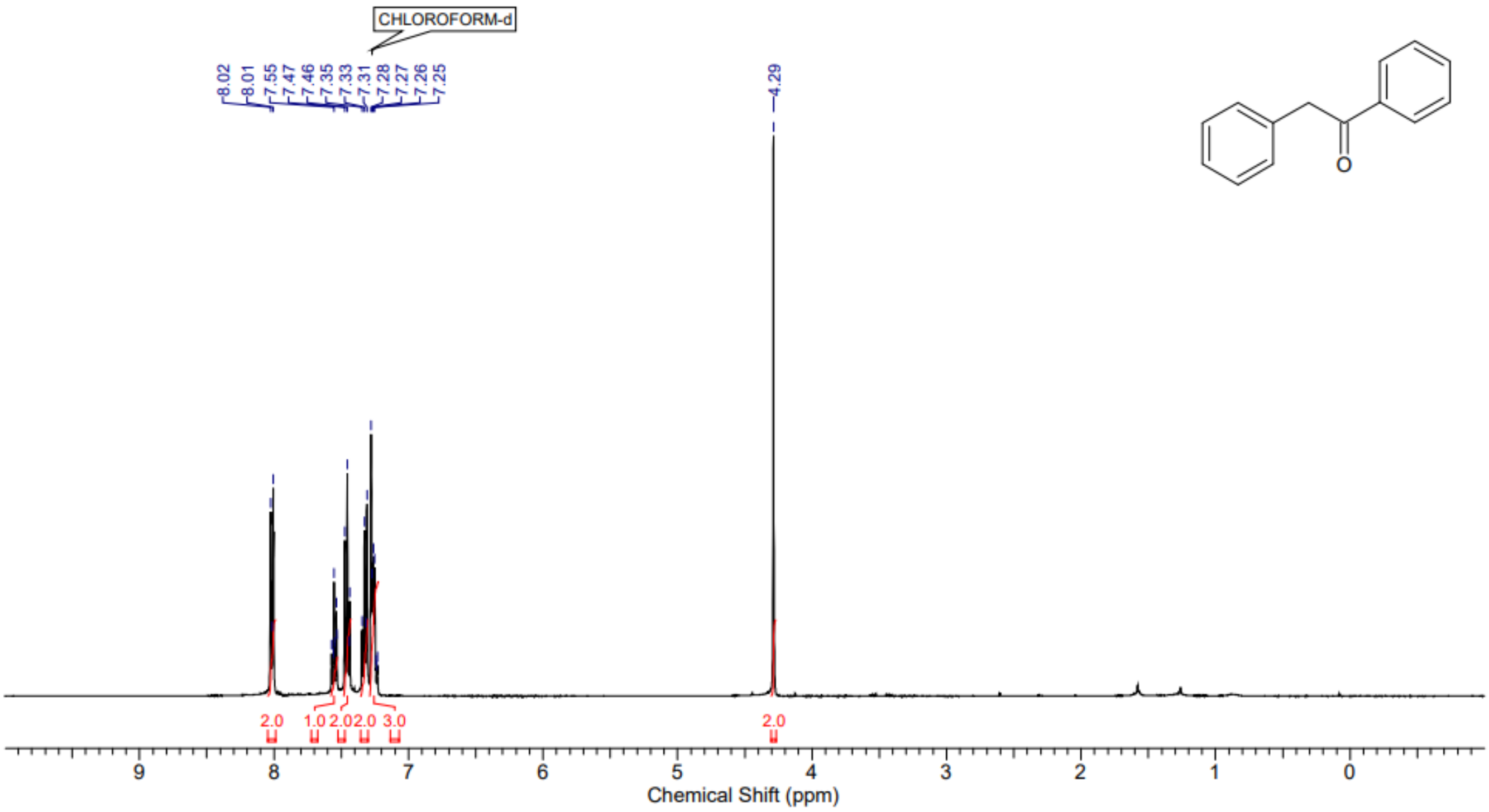

^{13}C NMR (101 MHz, $CDCl_3$)

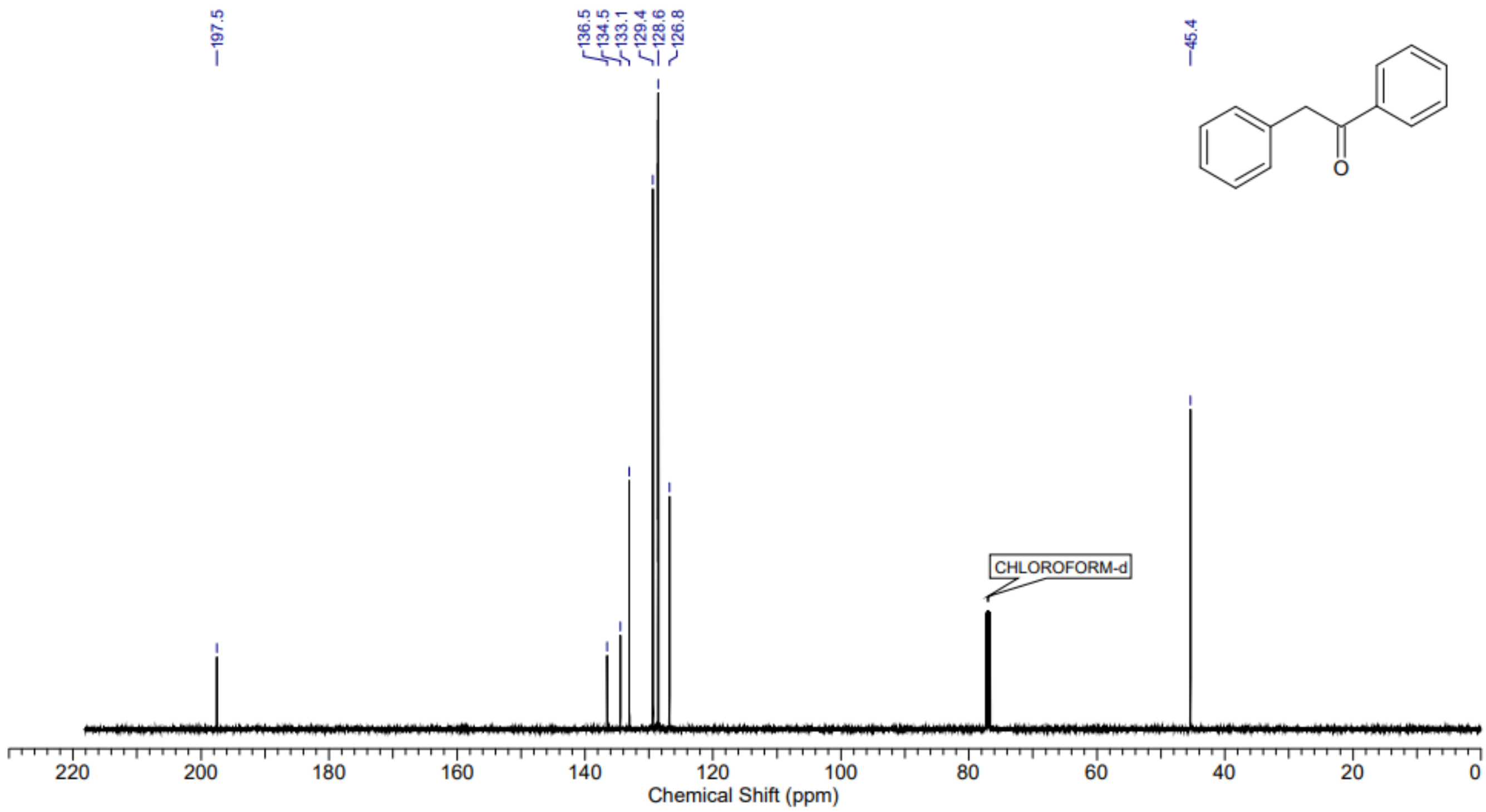

2-(2-Methoxyphenyl)-1-phenylethanone (**3da**) [CAS: 27356-33-8]

^{1}H NMR (400 MHz, $CDCl_3$)

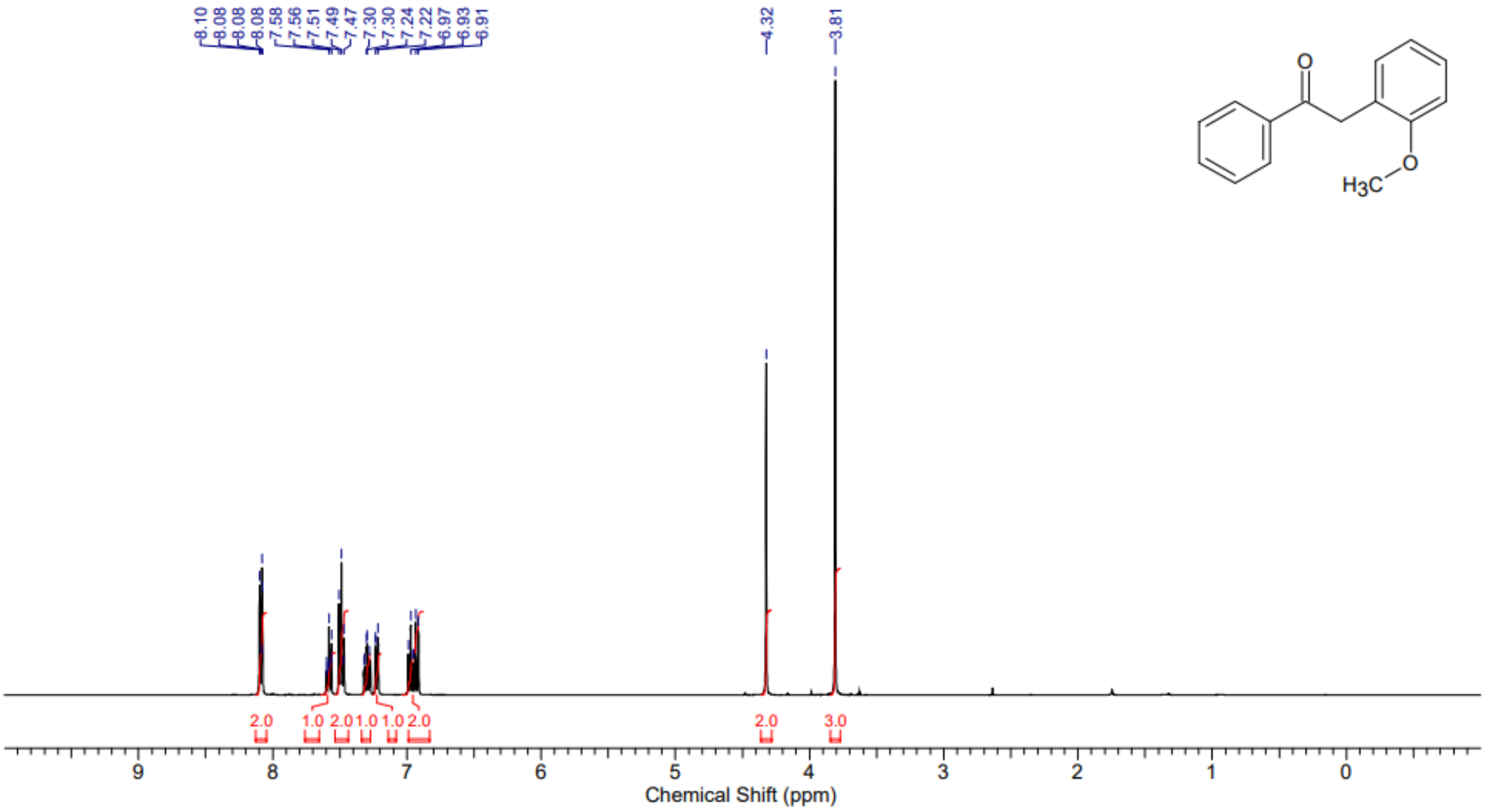

^{13}C NMR (101 MHz, $CDCl_3$)

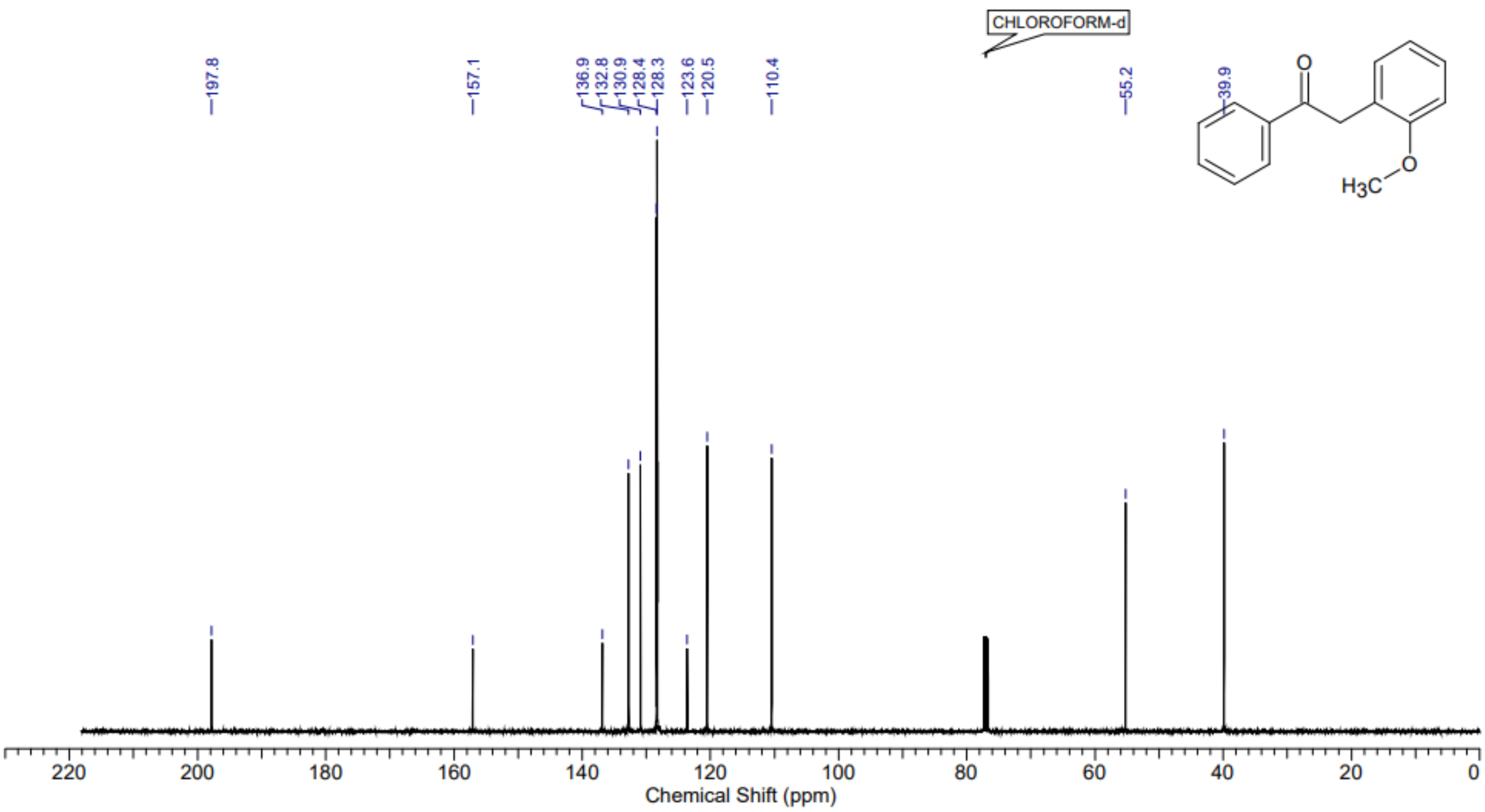

2-(2-Methylphenyl)-1-phenylethanone (**3ea**) [CAS: 5033-67-0]

^{1}H NMR (400 MHz, $CDCl_3$)

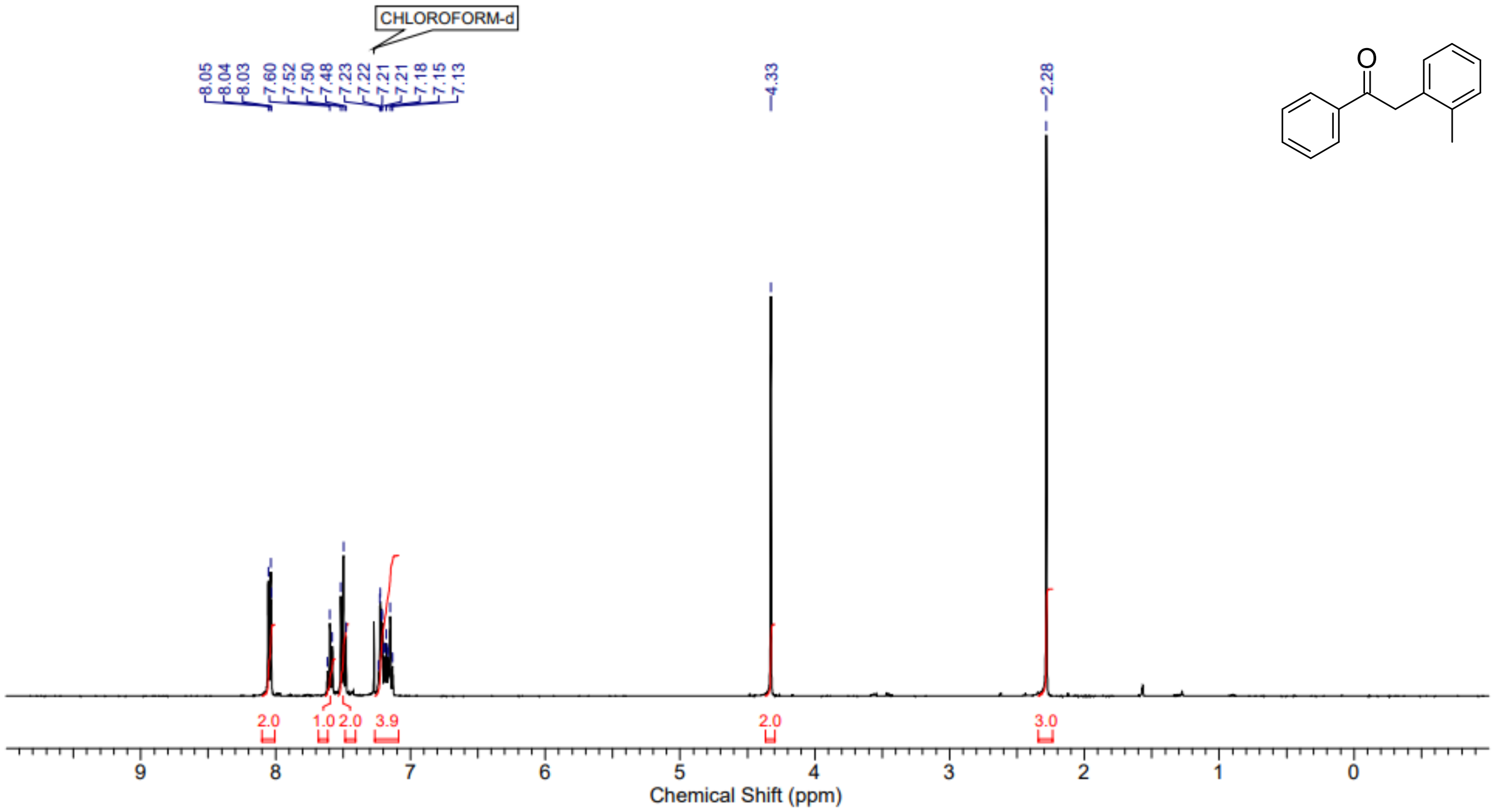

^{13}C NMR (101 MHz, $CDCl_3$)

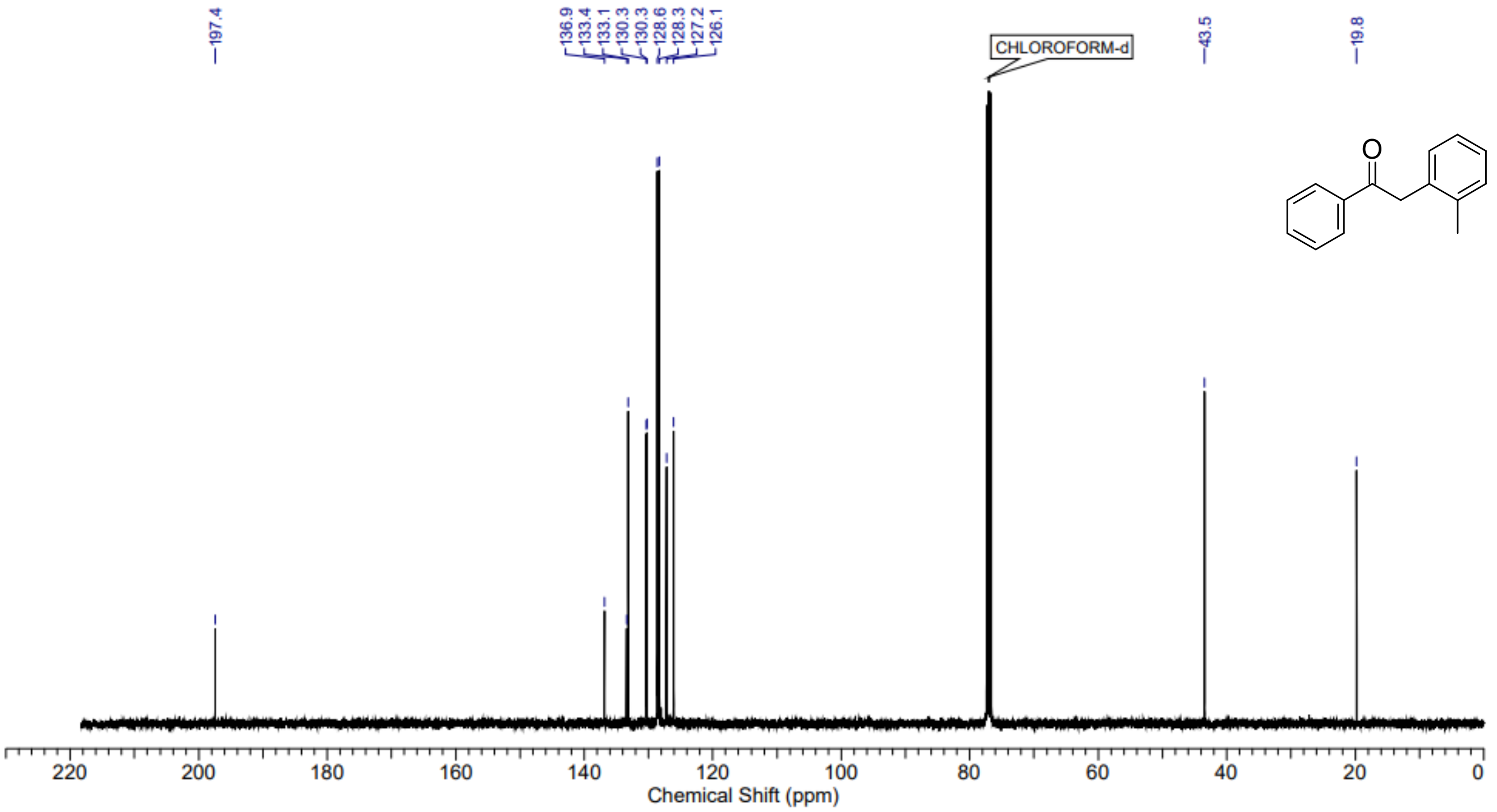

2-(2-Fluorophenyl)-1-phenylethanone (**3fa**) [CAS: 3826-47-9]

^{1}H NMR (400 MHz, $CDCl_3$)

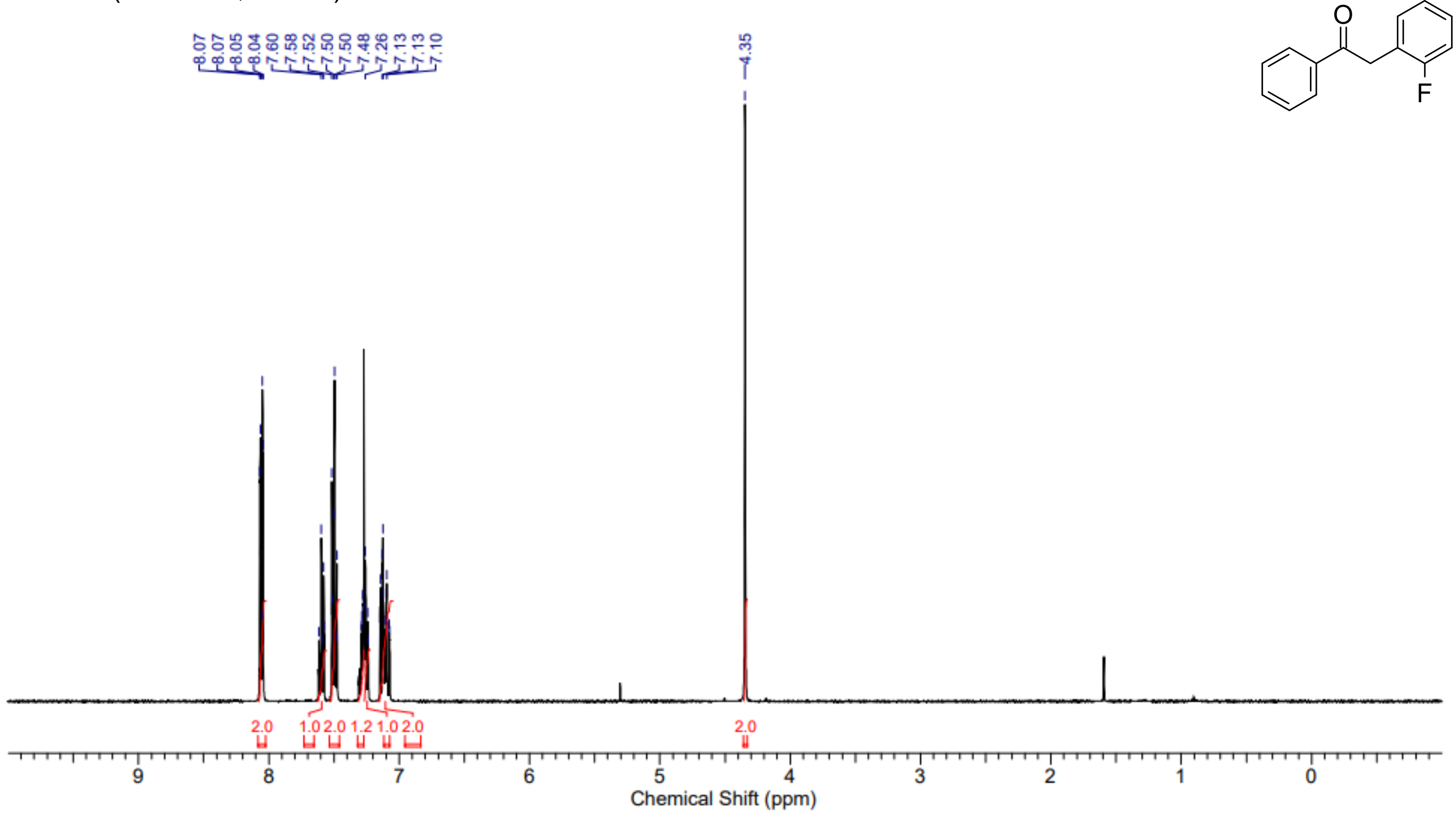

^{13}C NMR (101 MHz, $CDCl_3$)

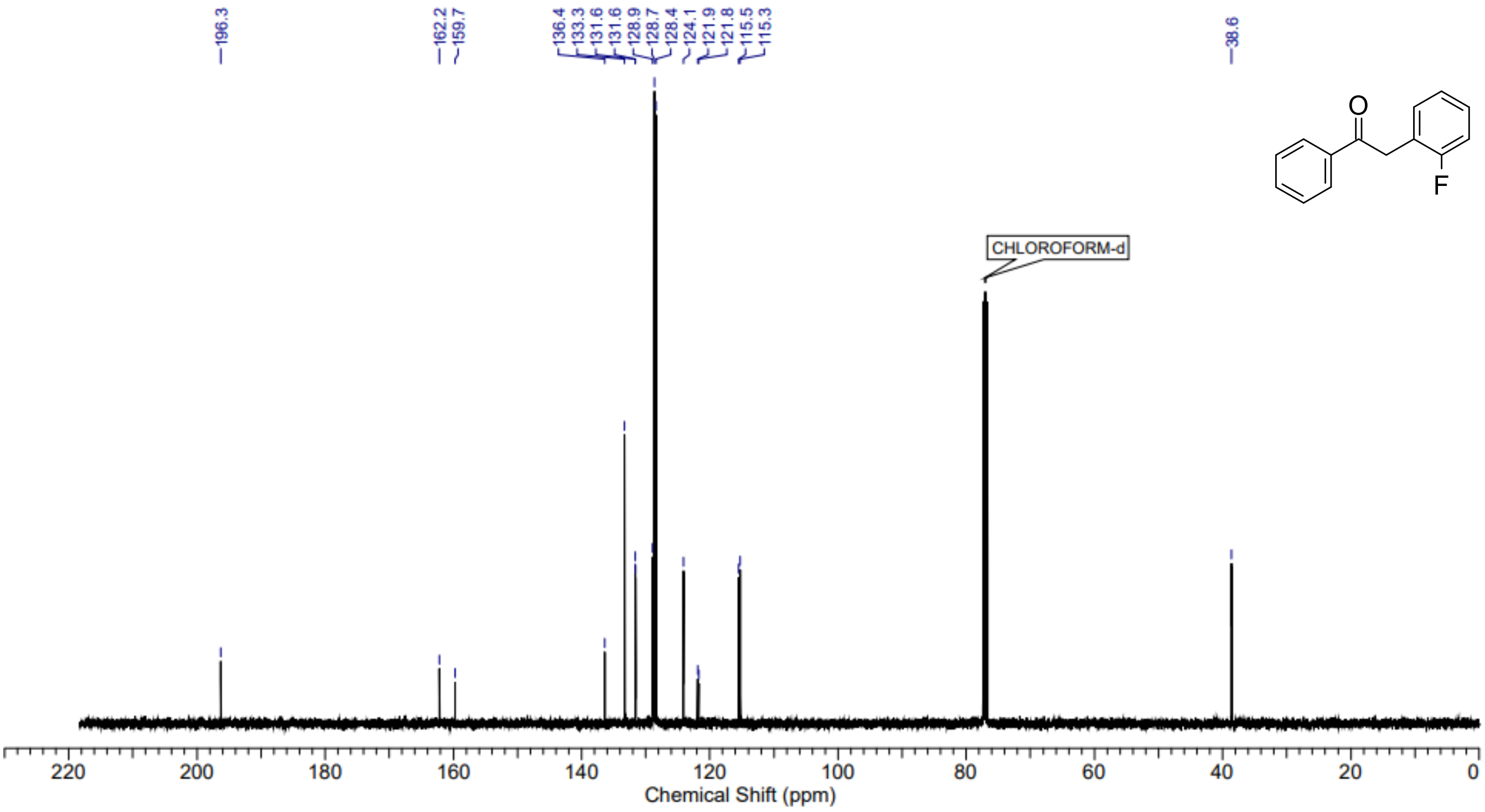

^{19}F NMR (377 MHz, $CDCl_3$)

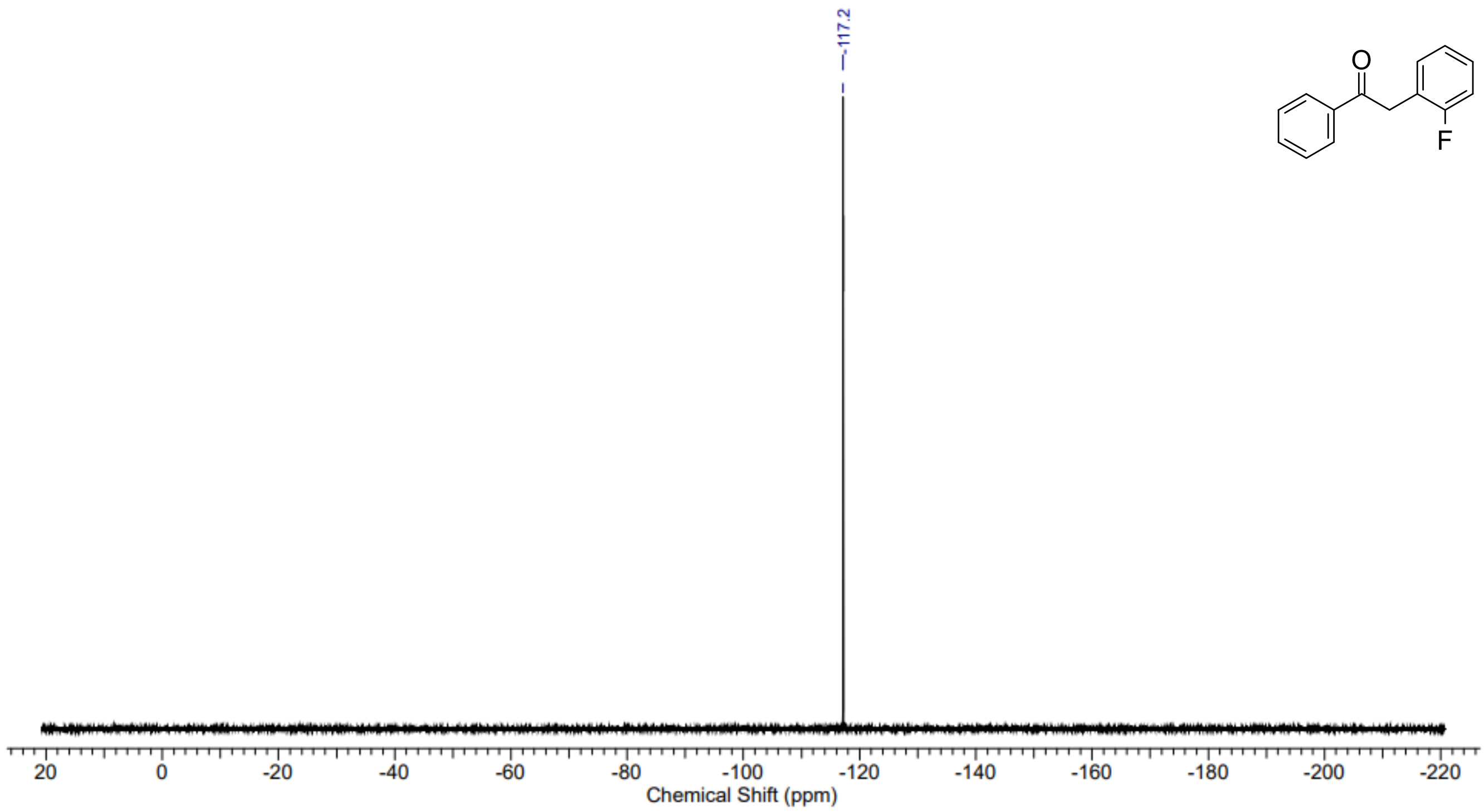

2-(3-Methoxyphenyl)-1-phenylethanone (**3ga**) [CAS: 29955-26-8]

^1H NMR (400 MHz, $CDCl_3$)

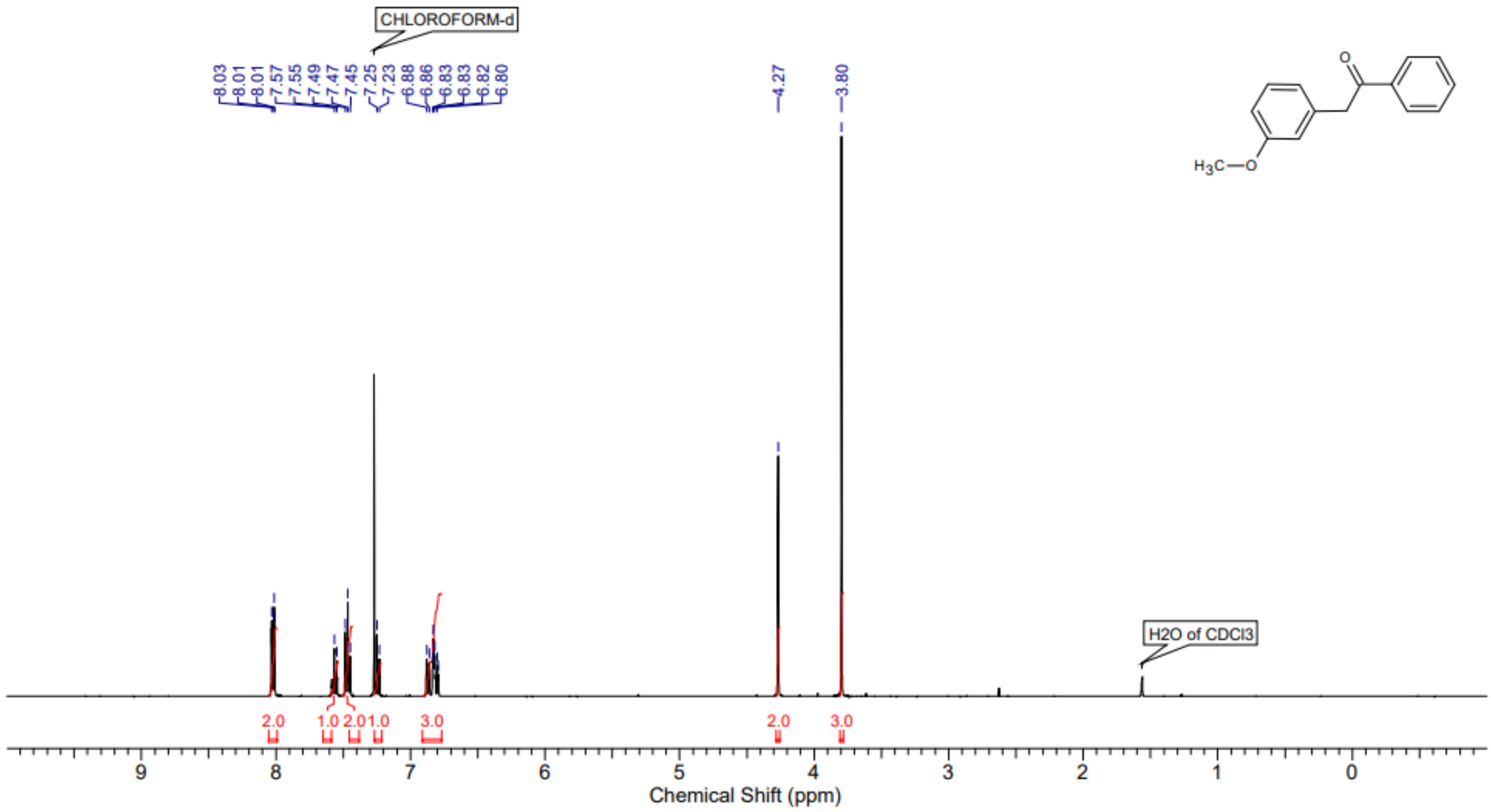

^{13}C NMR (101 MHz, $CDCl_3$)

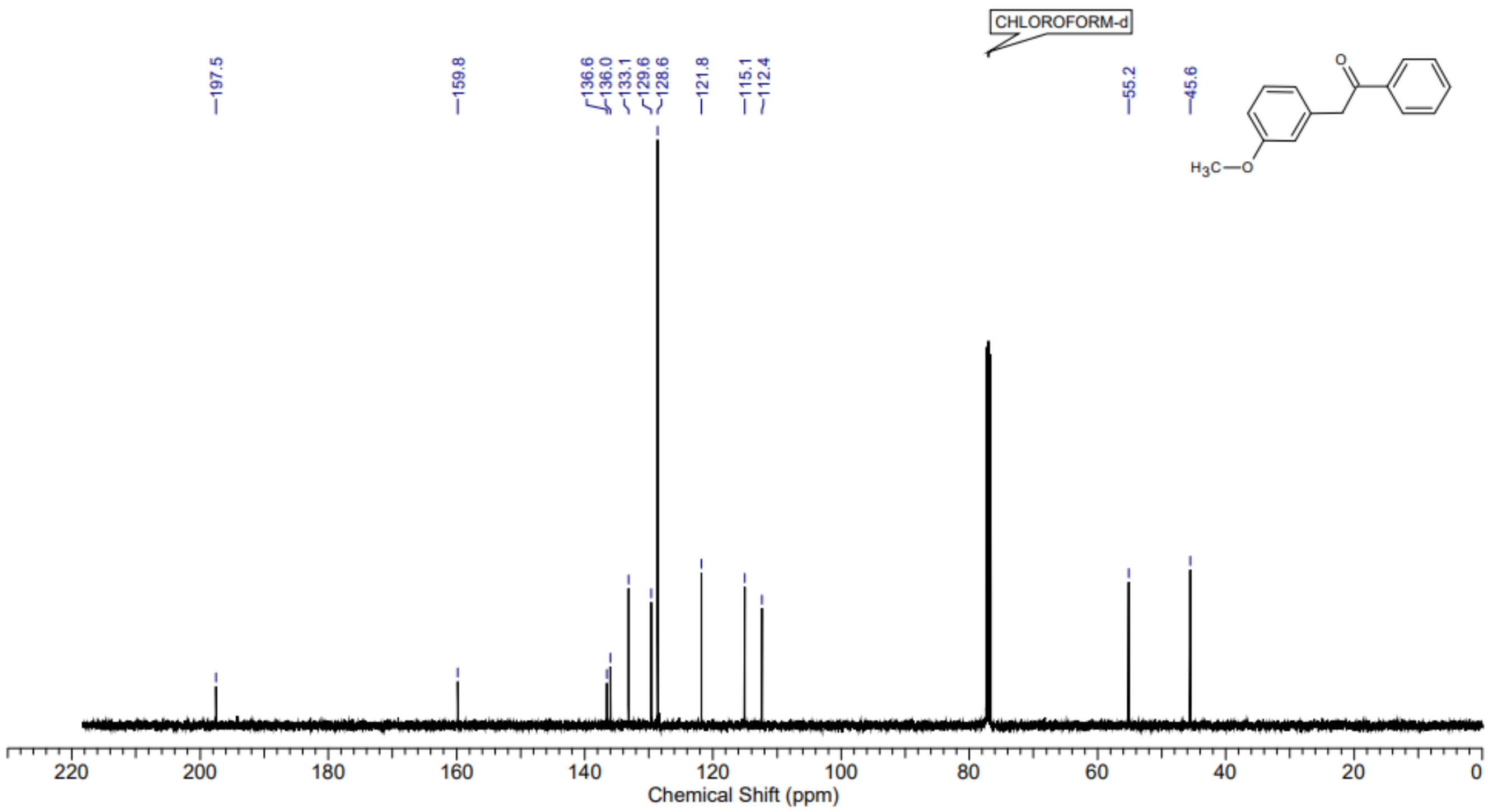

2-(1-Naphthalenyl)-1-phenylethanone (**3ha**) [CAS: 16216-08-3]

^{1}H NMR (400 MHz, $CDCl_3$)

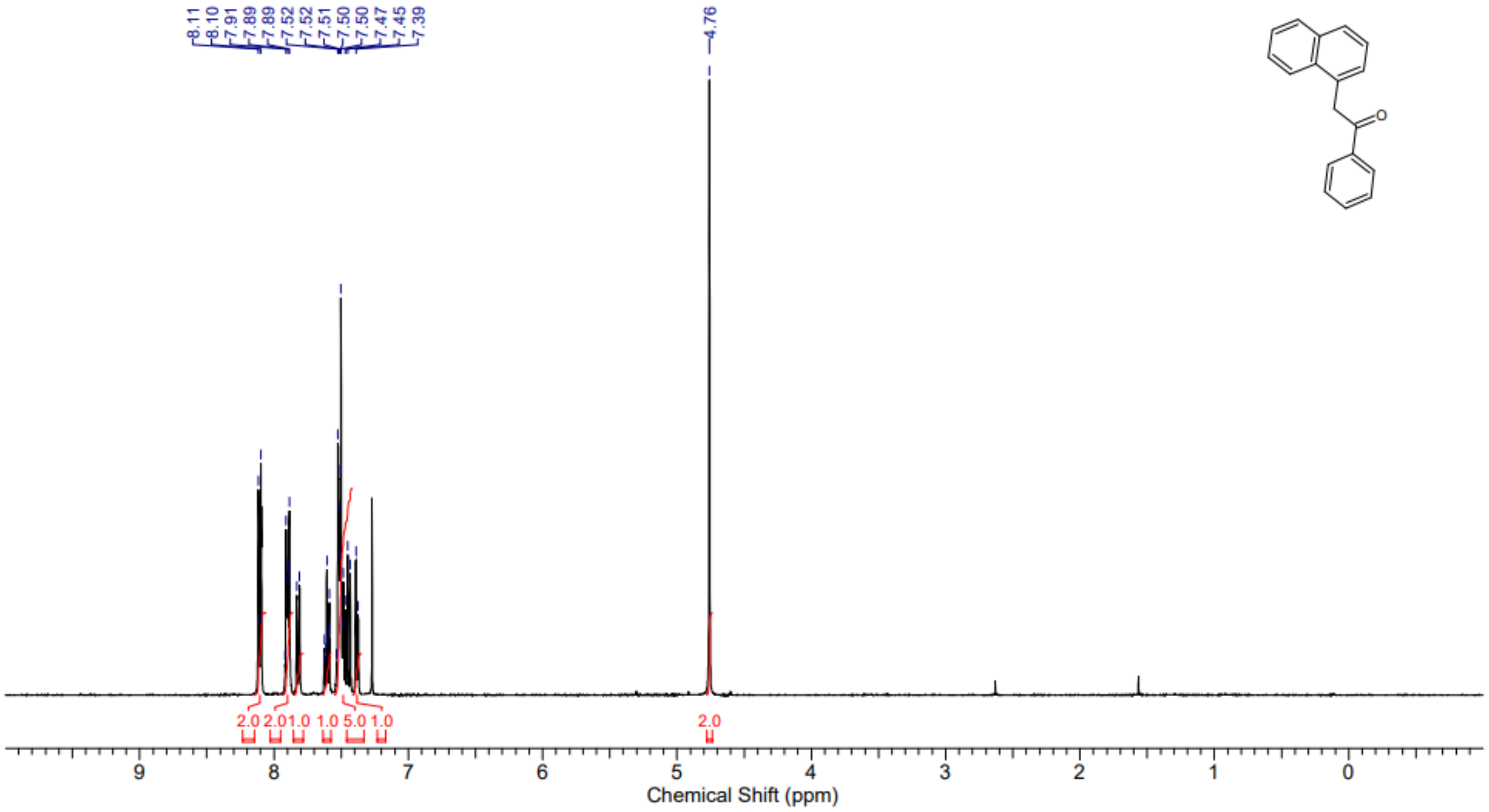

^{13}C NMR (101 MHz, $CDCl_3$)

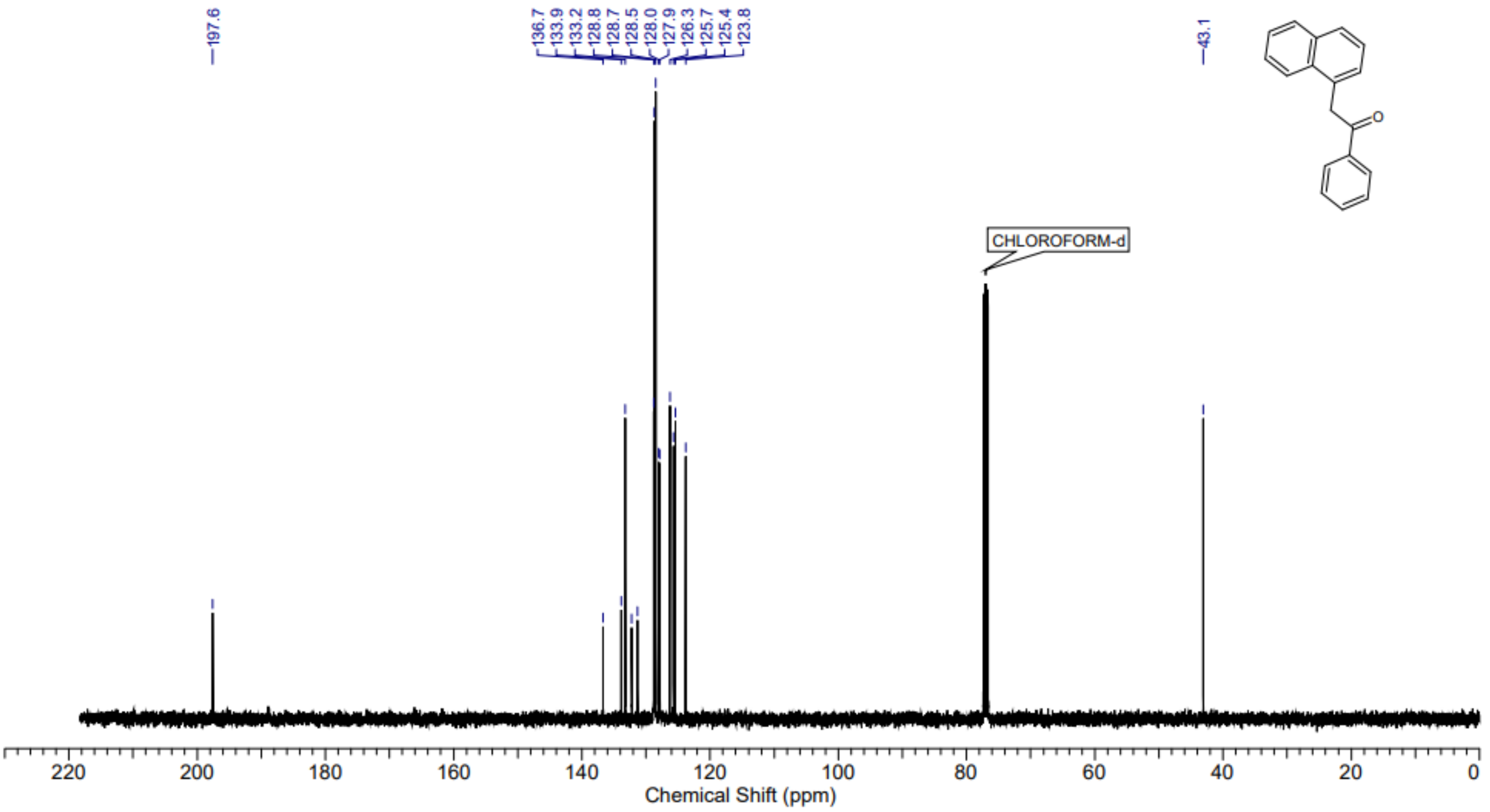

2-[4-(Methylthio)phenyl]-1-phenylethanone (**3ia**) [CAS: 177560-74-6]

^{1}H NMR (400 MHz, $CDCl_3$)

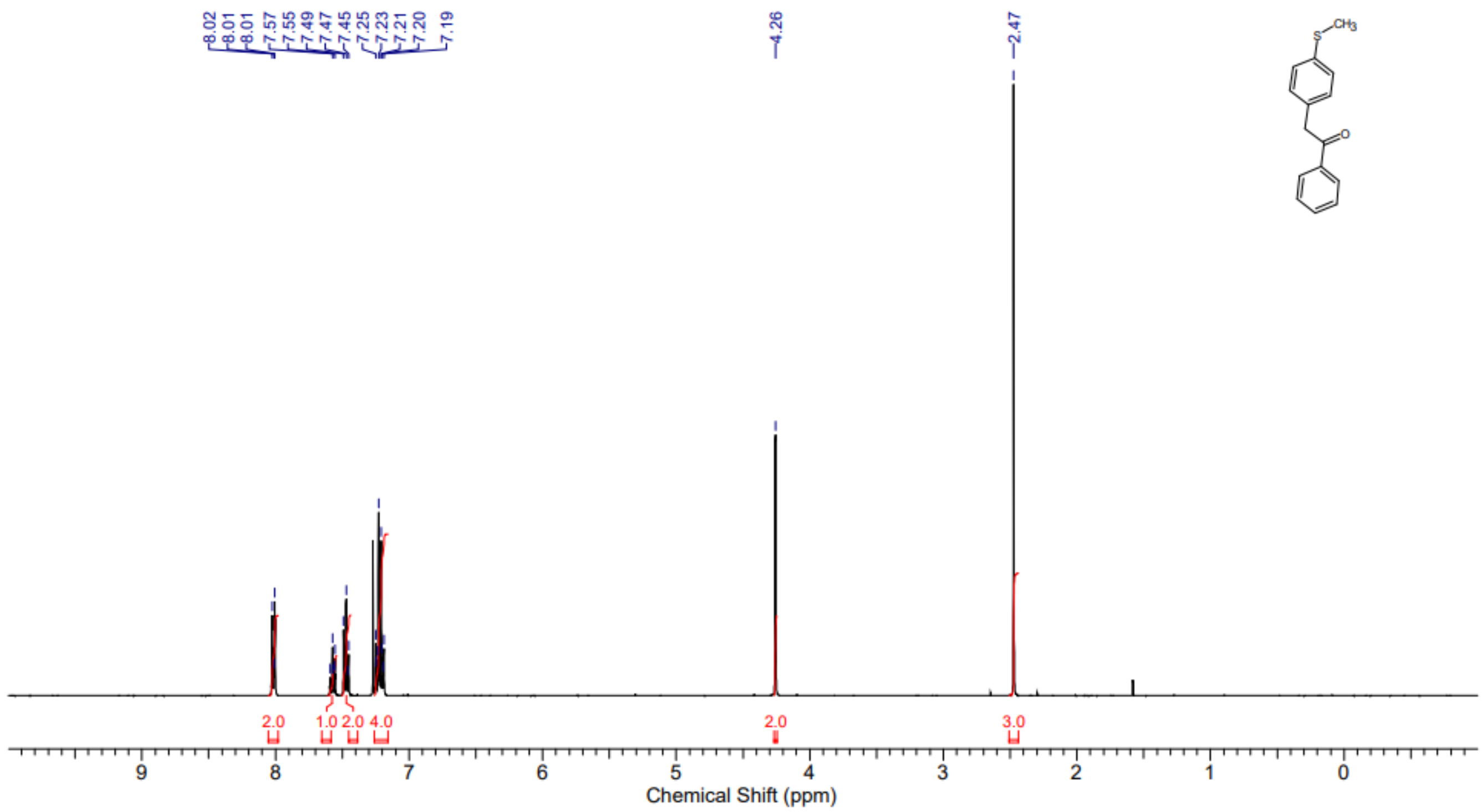

^{13}C NMR (101 MHz, $CDCl_3$)

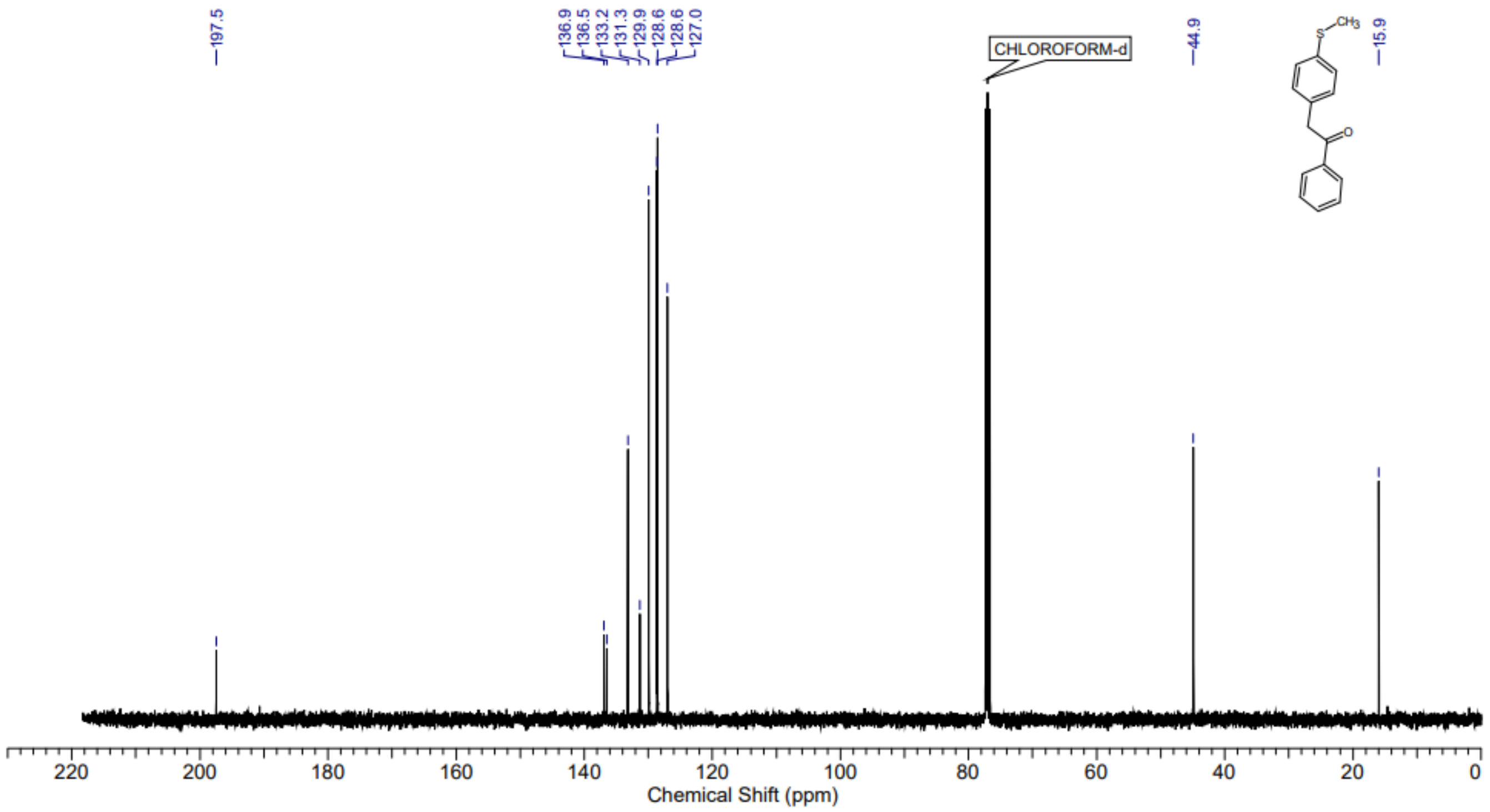

1-Phenyl-2-[4-(trifluoromethoxy)phenyl]ethanone (**3ja**) [CAS: 1496328-62-1]

^{1}H NMR (400 MHz, $CDCl_3$)

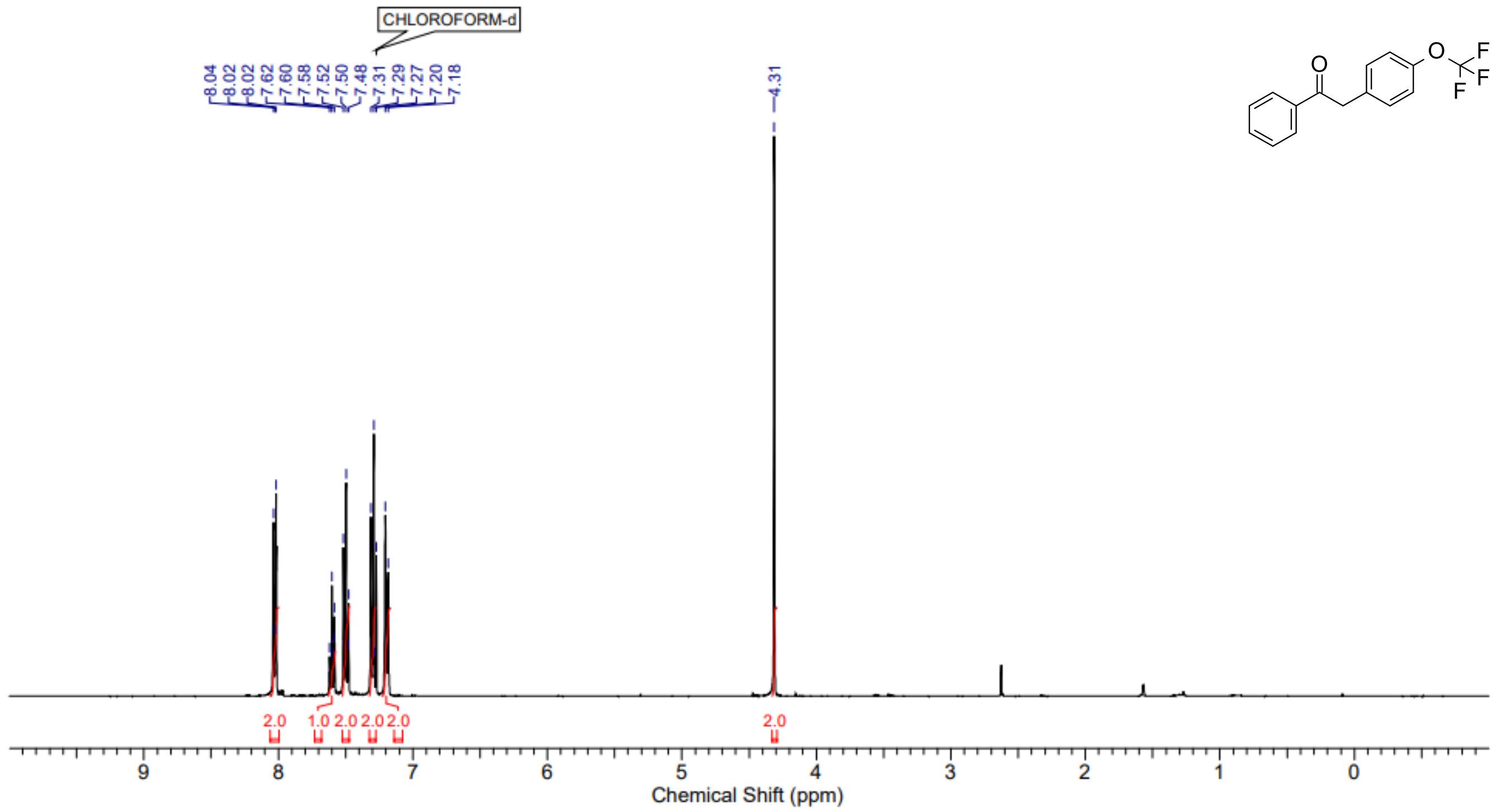

^{13}C NMR (101 MHz, $CDCl_3$)

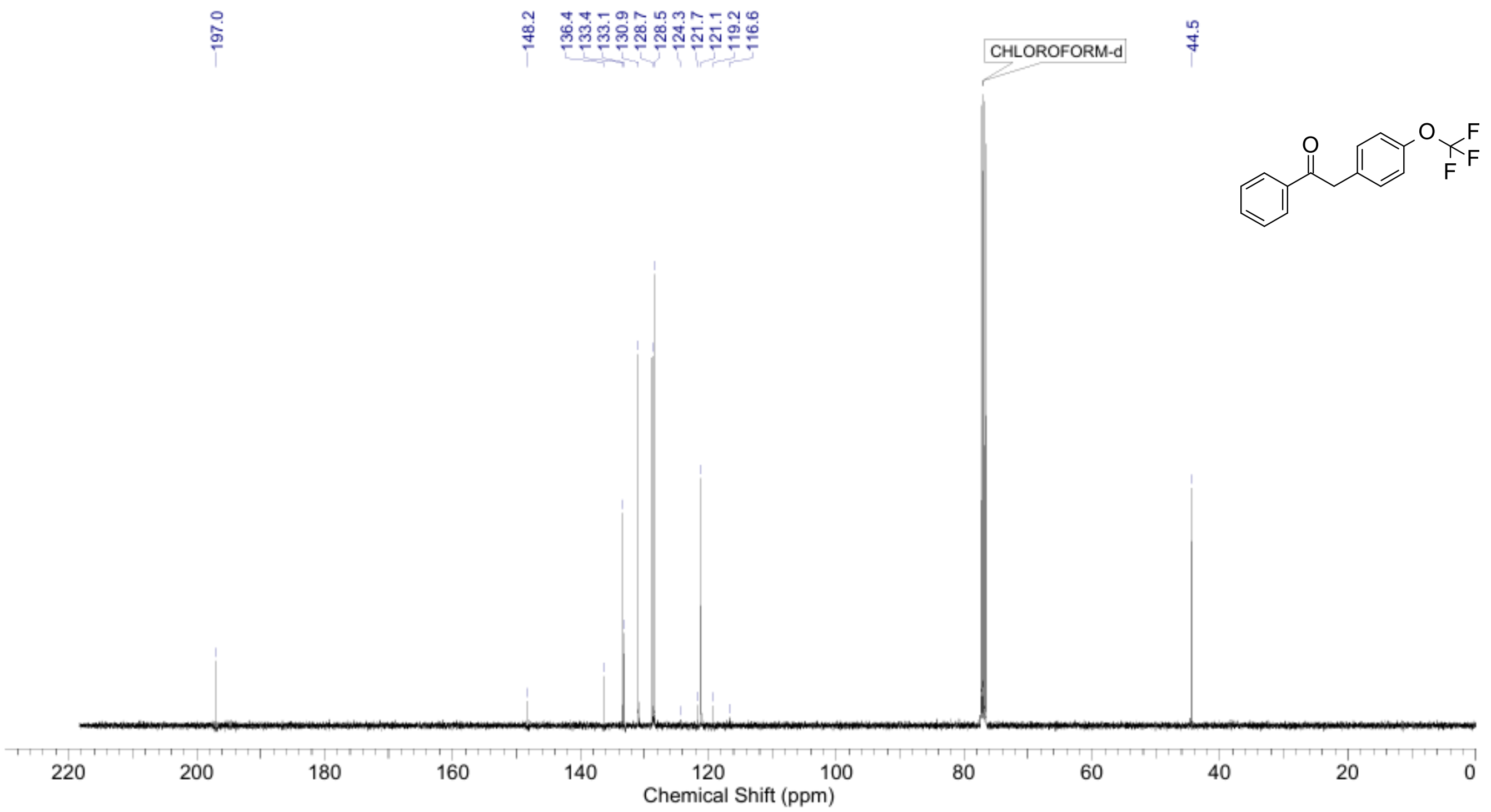

^{19}F NMR (377 MHz, $CDCl_3$)

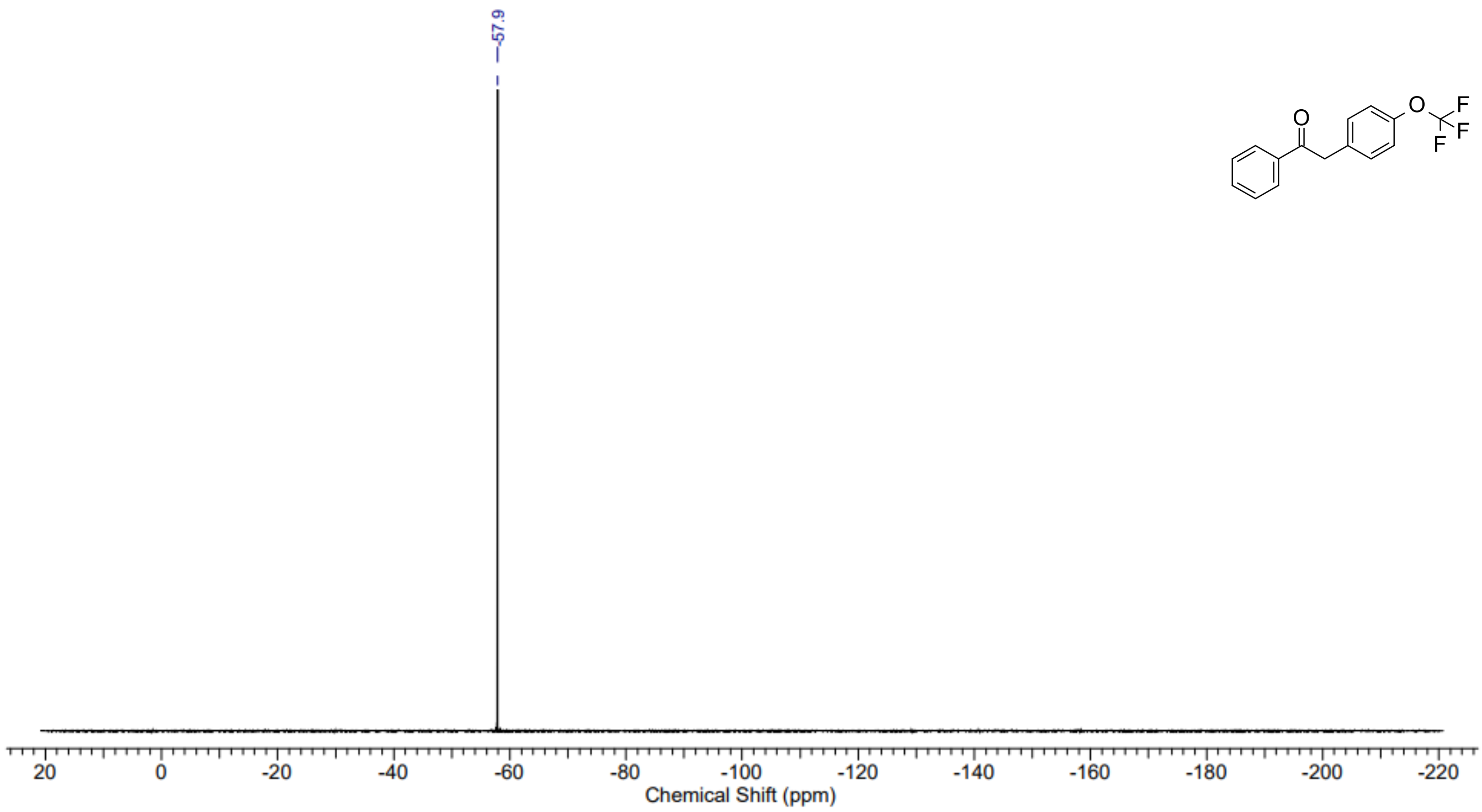

1-phenyl-2-[4-(trimethylsilyl)phenyl]ethanone (**3ka**) [CAS: 1884595-69-0]

^{1}H NMR (400 MHz, $CDCl_3$)

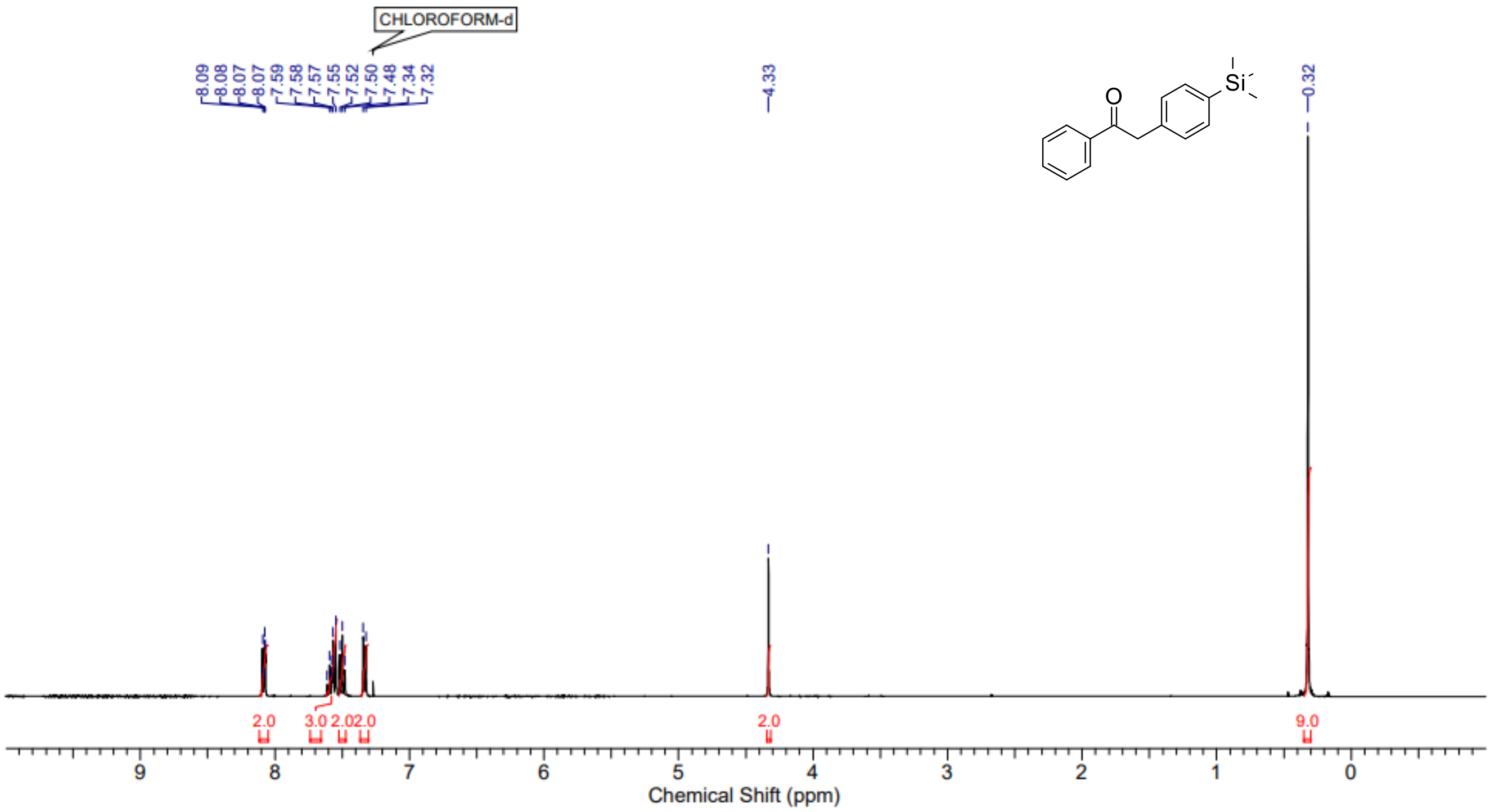

^{13}C NMR (101 MHz, $CDCl_3$)

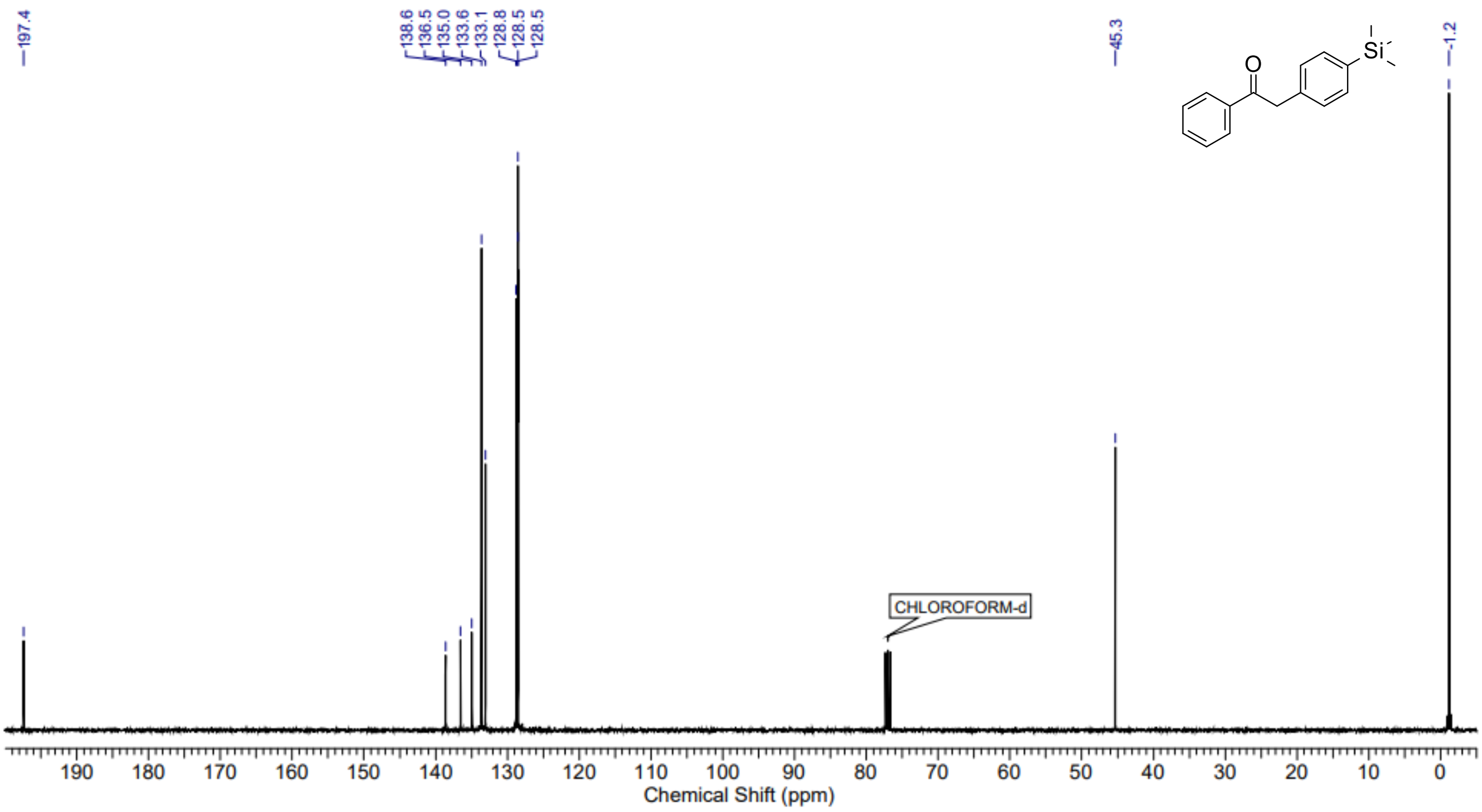

1-phenyl-2-[4-(4,4,5,5-tetramethyl-1,3,2-dioxaborolan-2-yl)phenyl]ethanone (**3la**) [CAS: 1802939-37-2]

^{1}H NMR (400 MHz, $CDCl_3$)

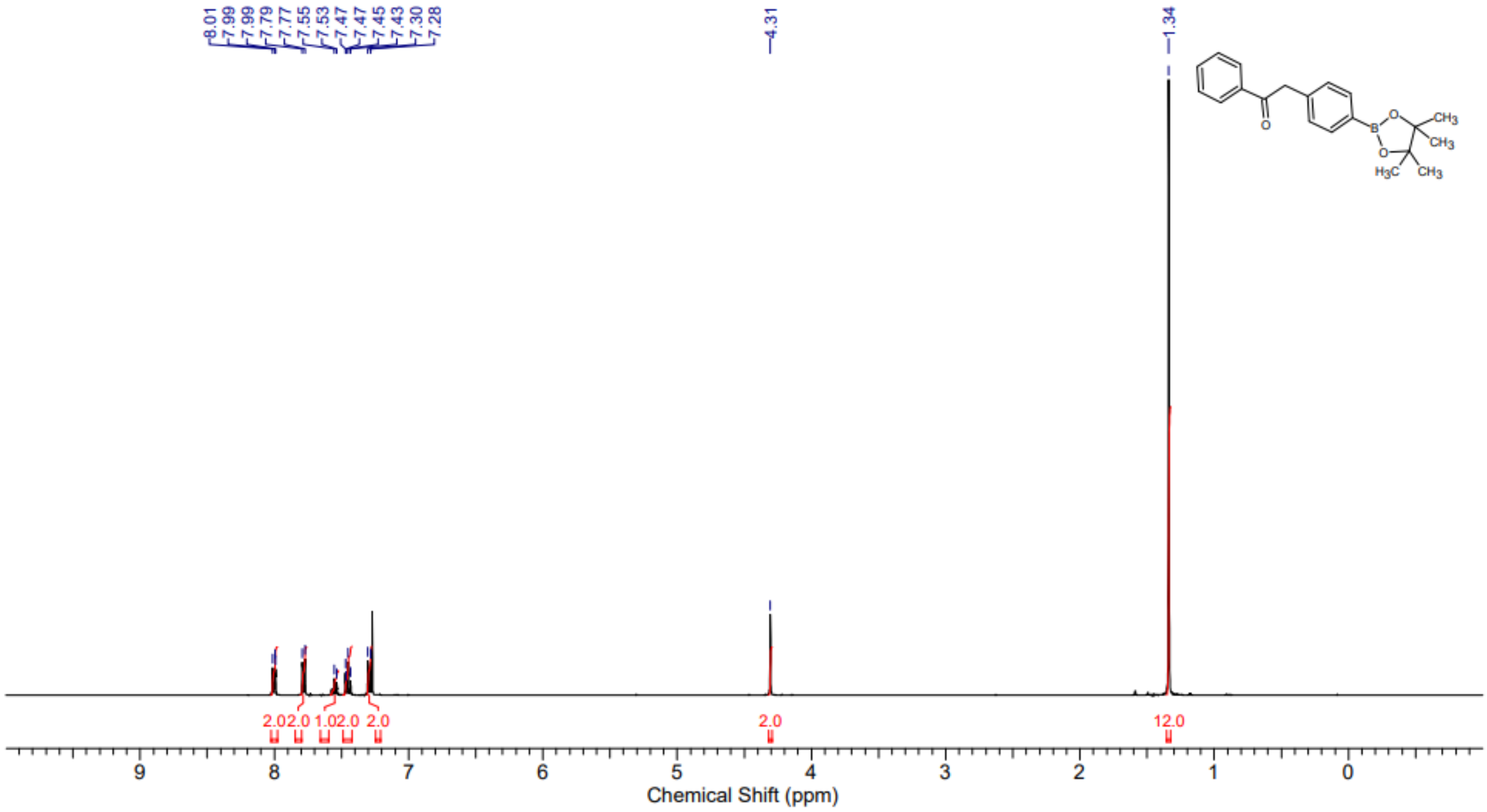

^{13}C NMR (101 MHz, $CDCl_3$)

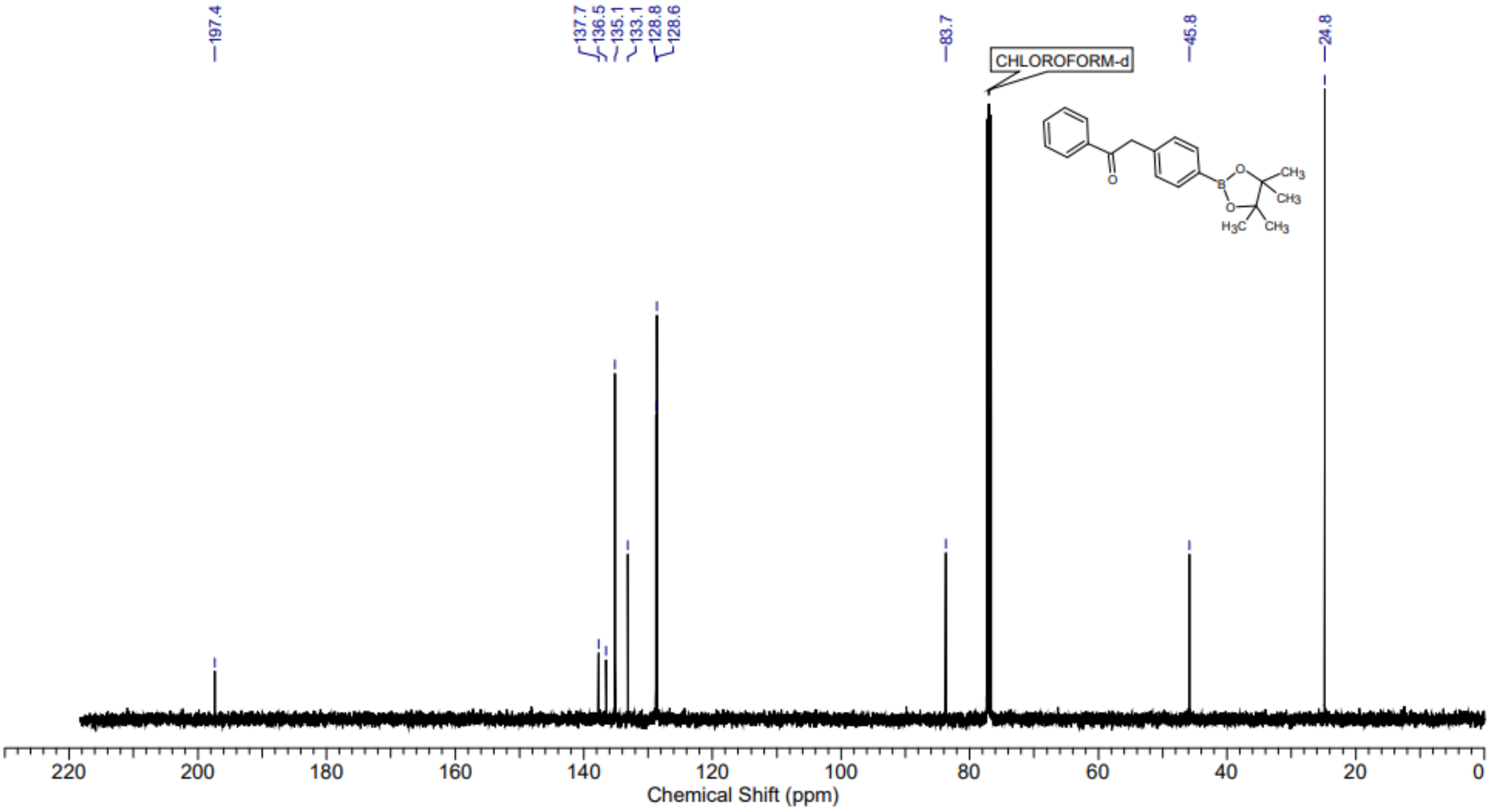

2-(4-(1H-pyrrol-1-yl)phenyl)-1-phenylethan-1-one (**3ma**)

^{1}H NMR (400 MHz, $CDCl_3$)

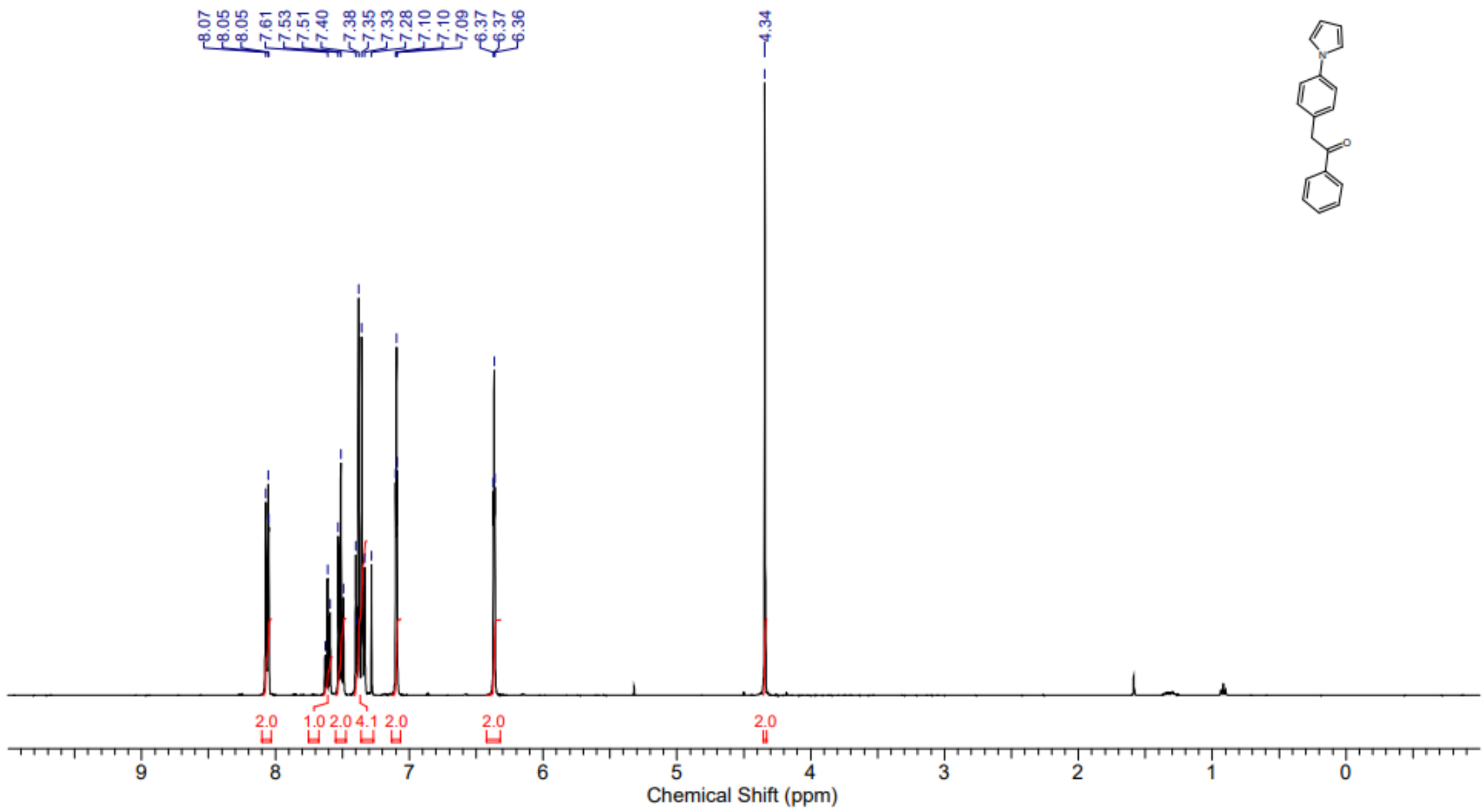

^{13}C NMR (101 MHz, $CDCl_3$)

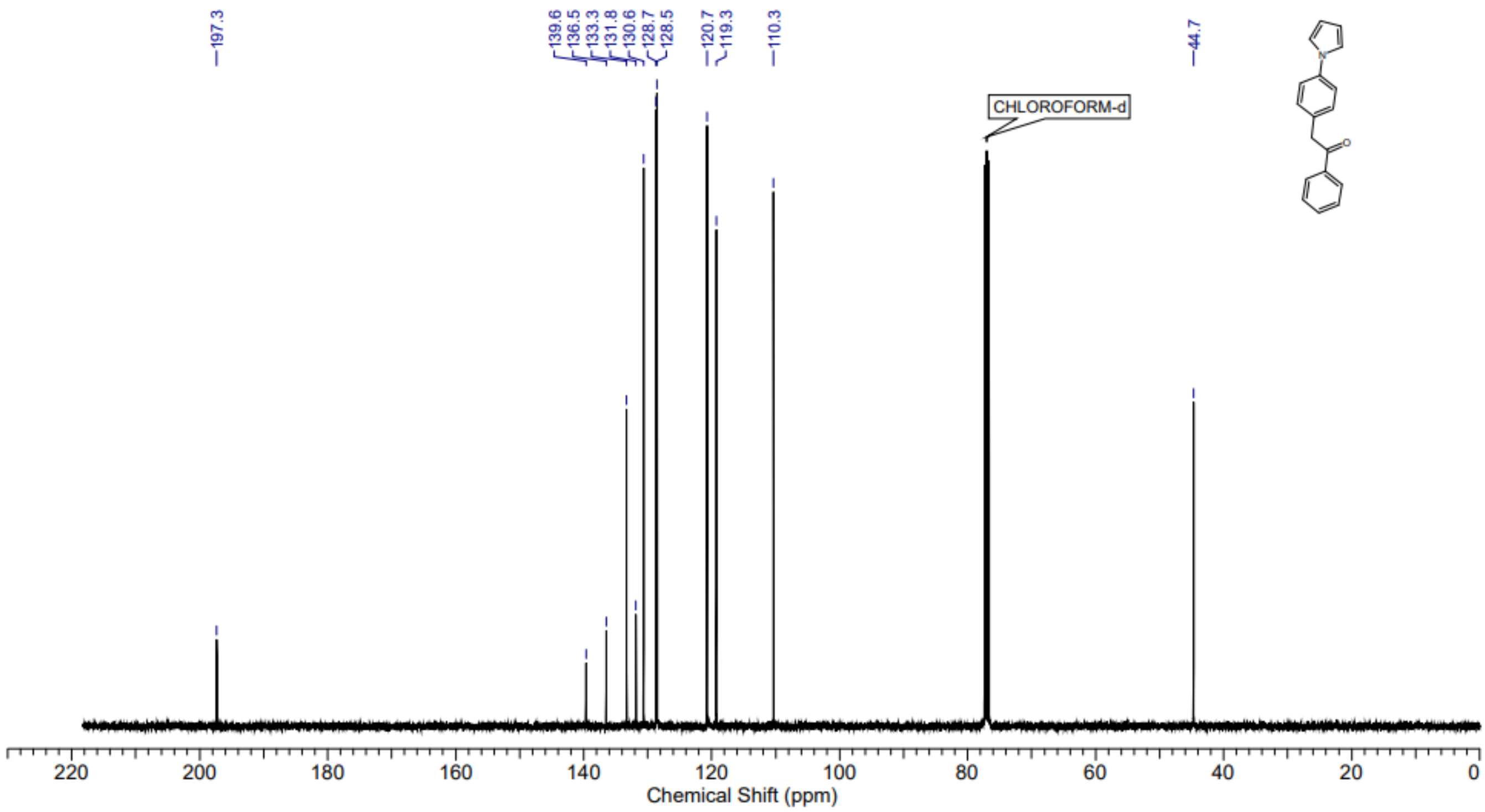

2-(benzo[b]thiophen-6-yl)-1-phenylethan-1-one (**3na**)

^{1}H NMR (400 MHz, $CDCl_3$)

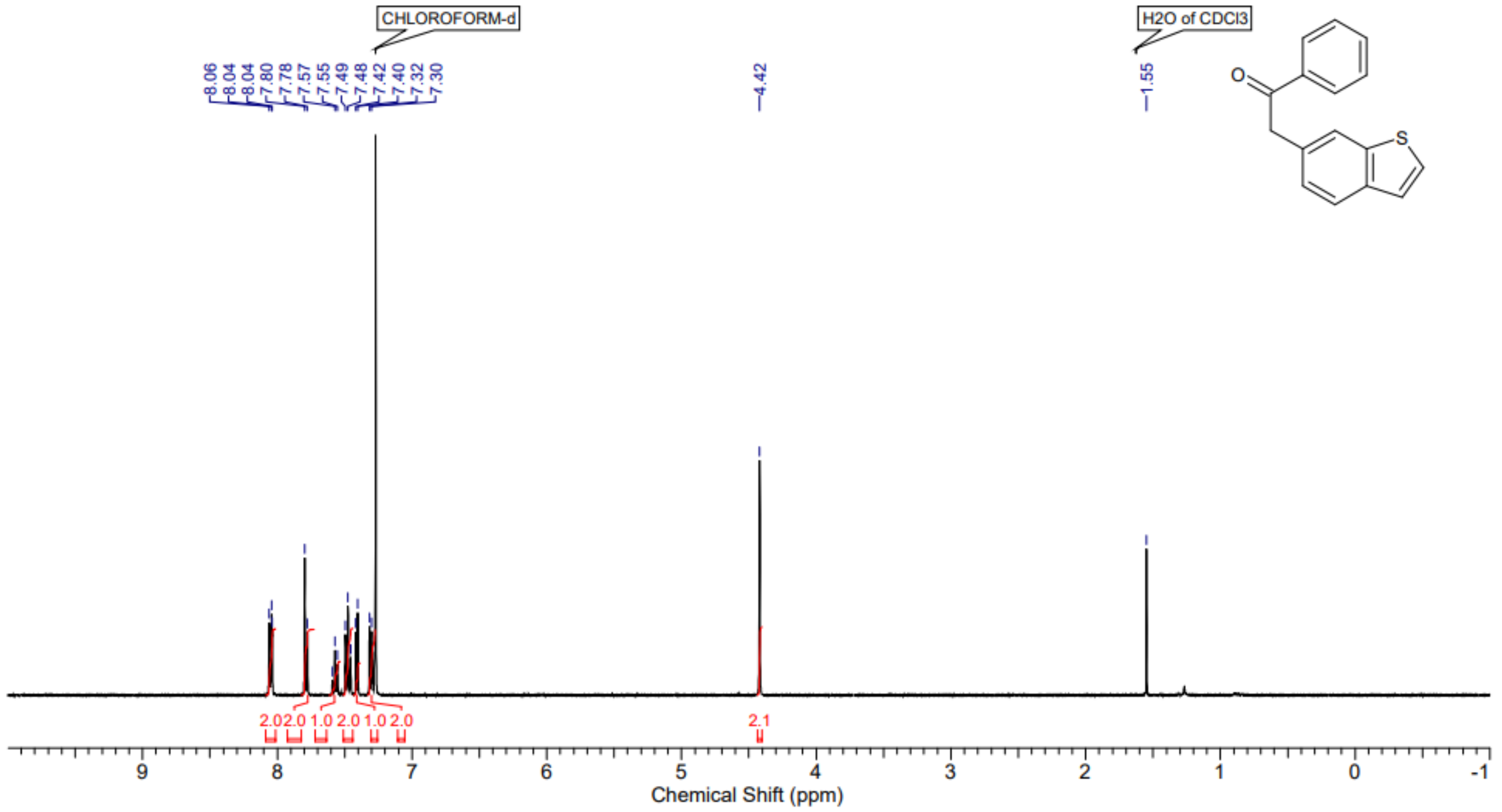

^{13}C NMR (101 MHz, $CDCl_3$)

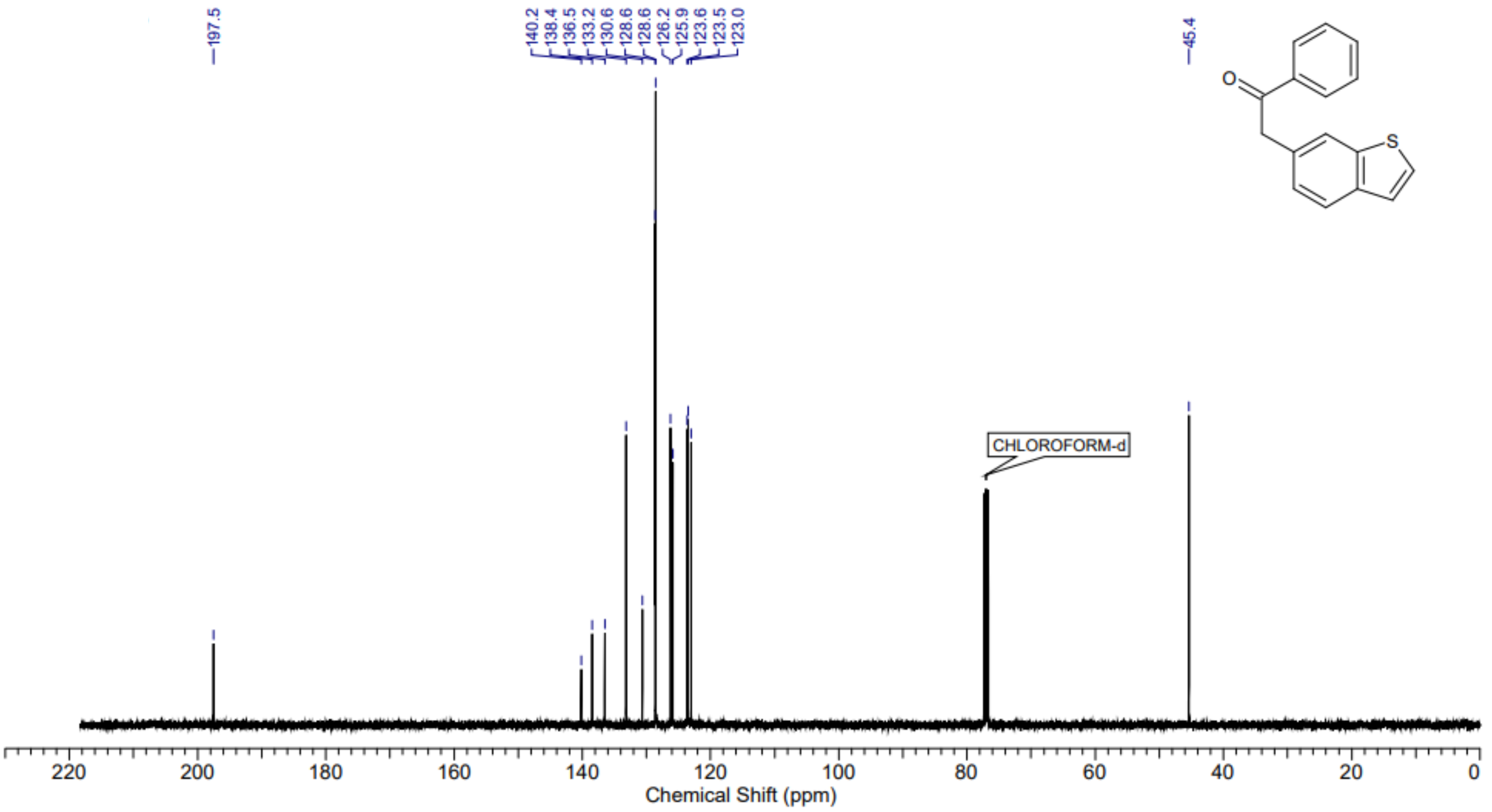

1-Phenyl-2-(2-pyridinyl)ethanone (**3oa**) [CAS: 1620-53-7]

^{1}H NMR (400 MHz, $CDCl_3$)

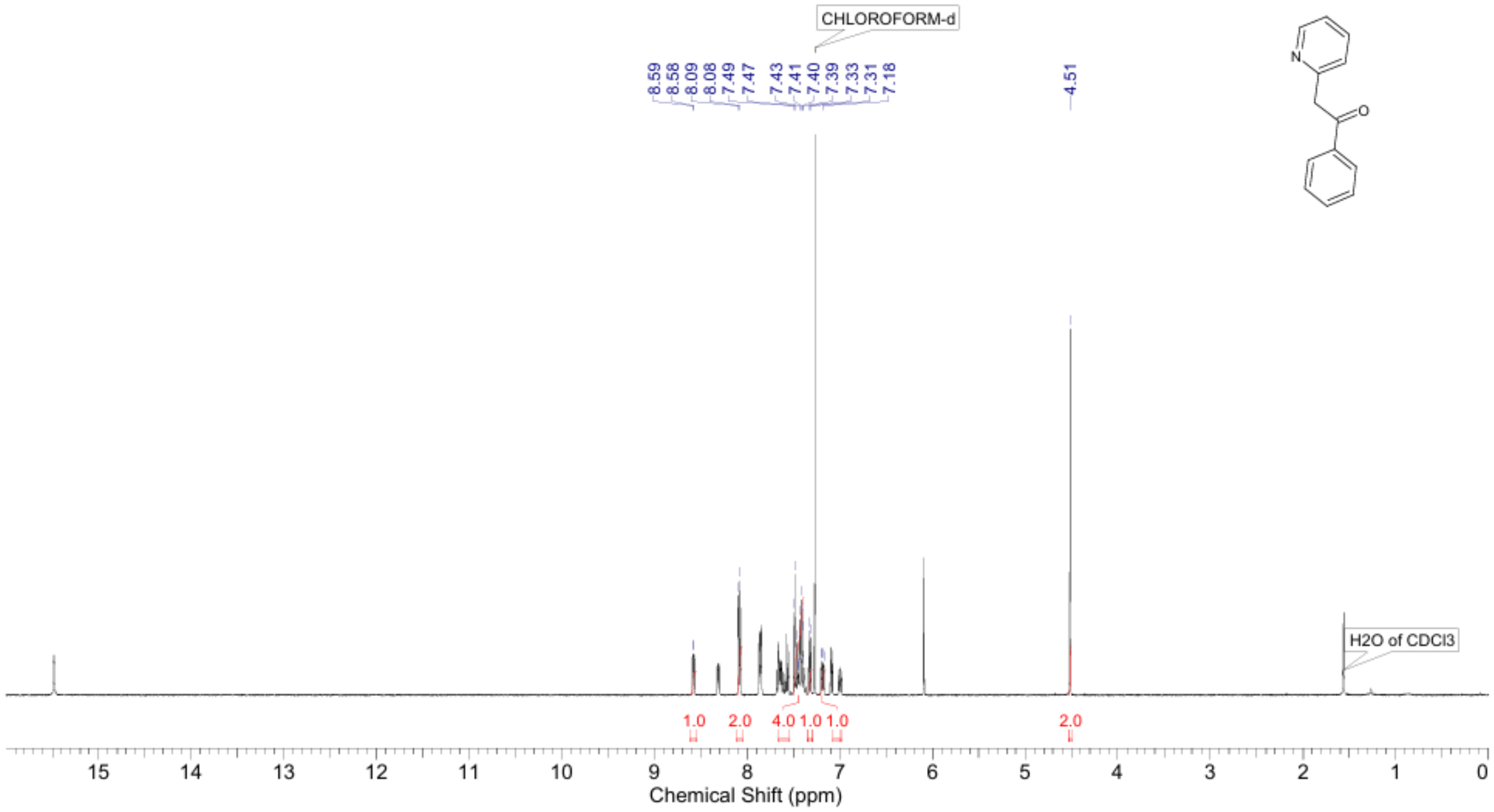

^{1}H NMR (400 MHz, $CDCl_3$) of the tautomer:

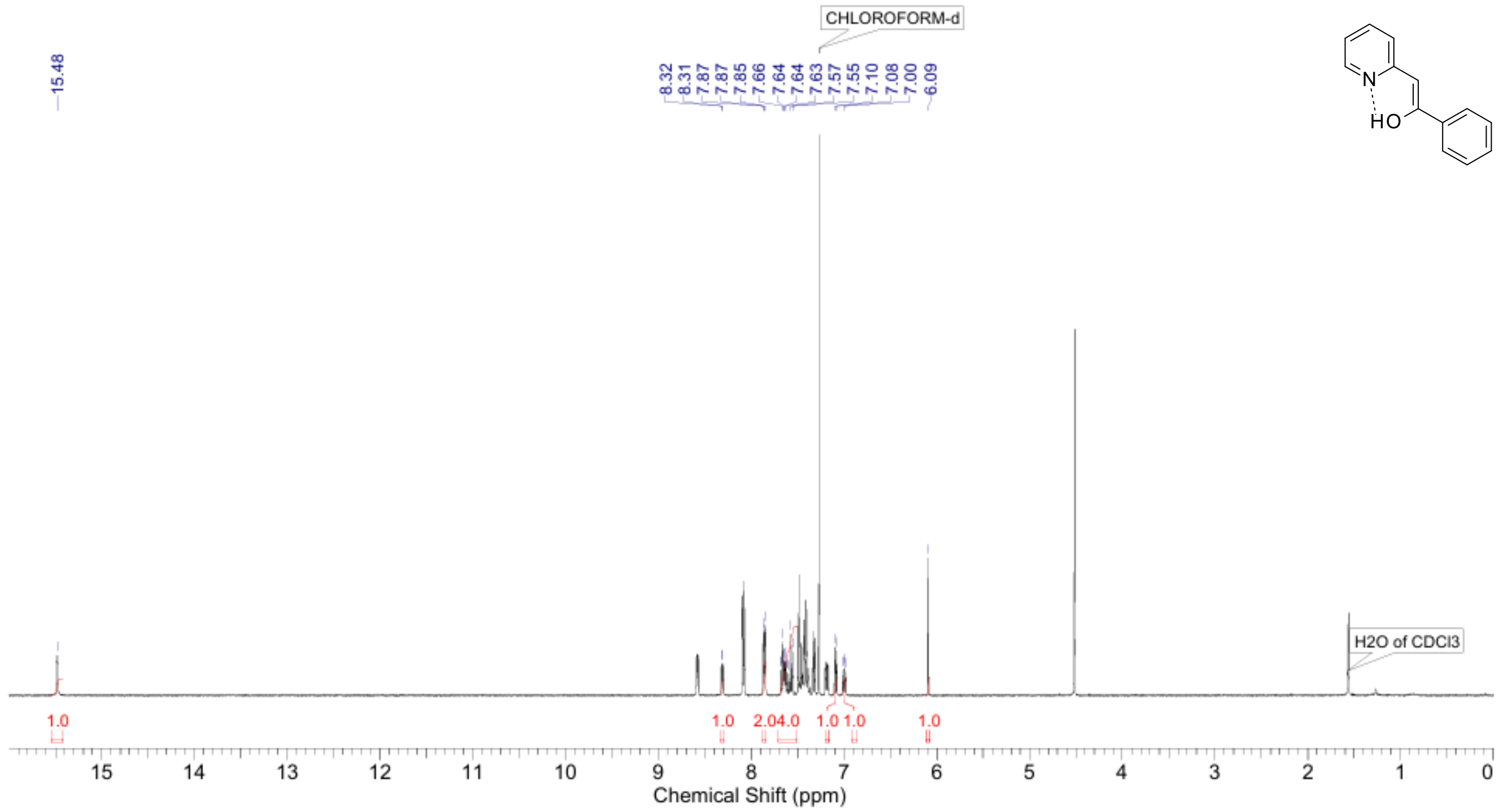

^{13}C NMR (101 MHz, $CDCl_3$)

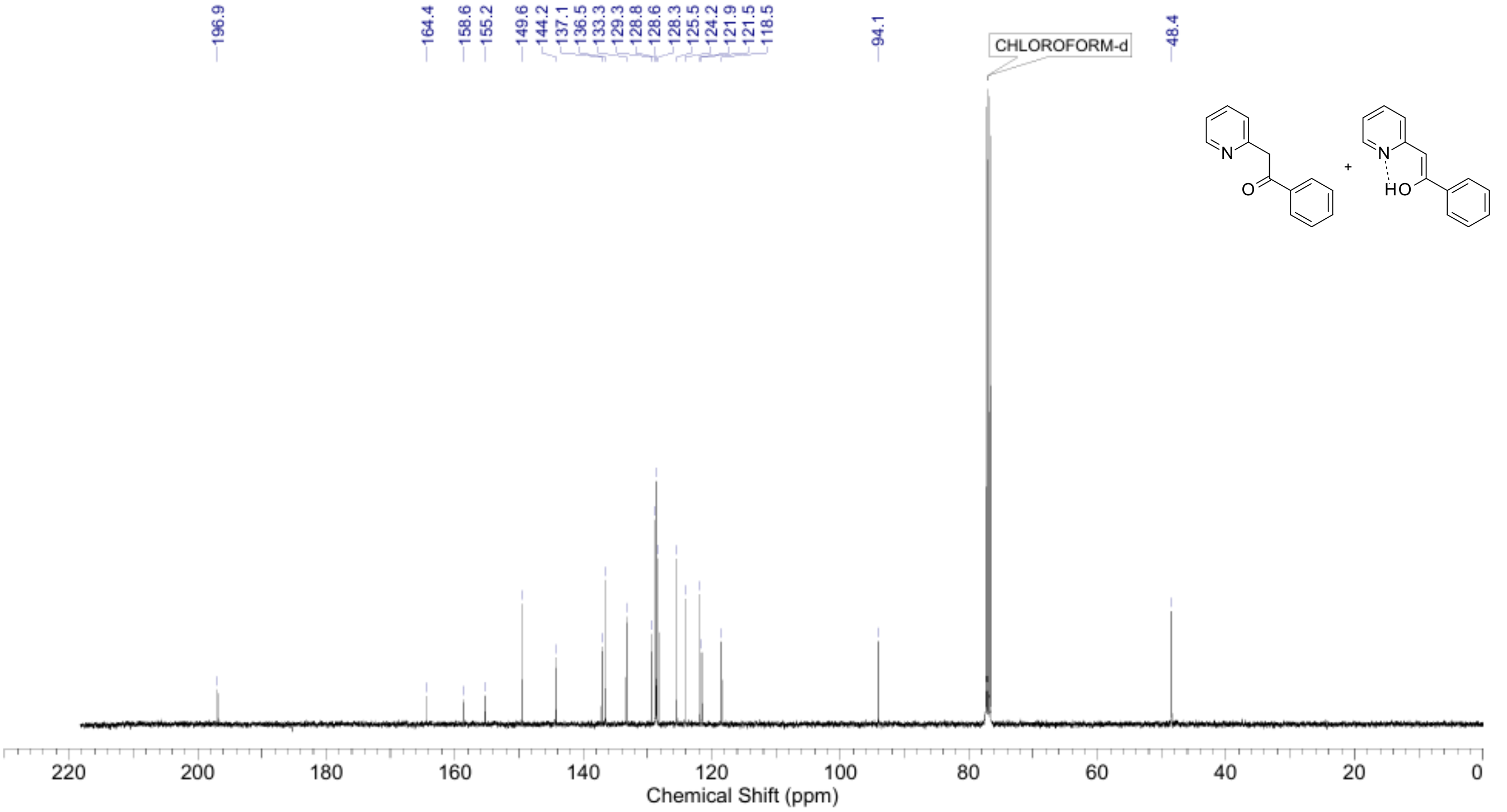

2-(2,3-difluorophenyl)-1-phenylethanone (**3pa**) [CAS: 1498370-47-0]

^{1}H NMR (400 MHz, $CDCl_3$)

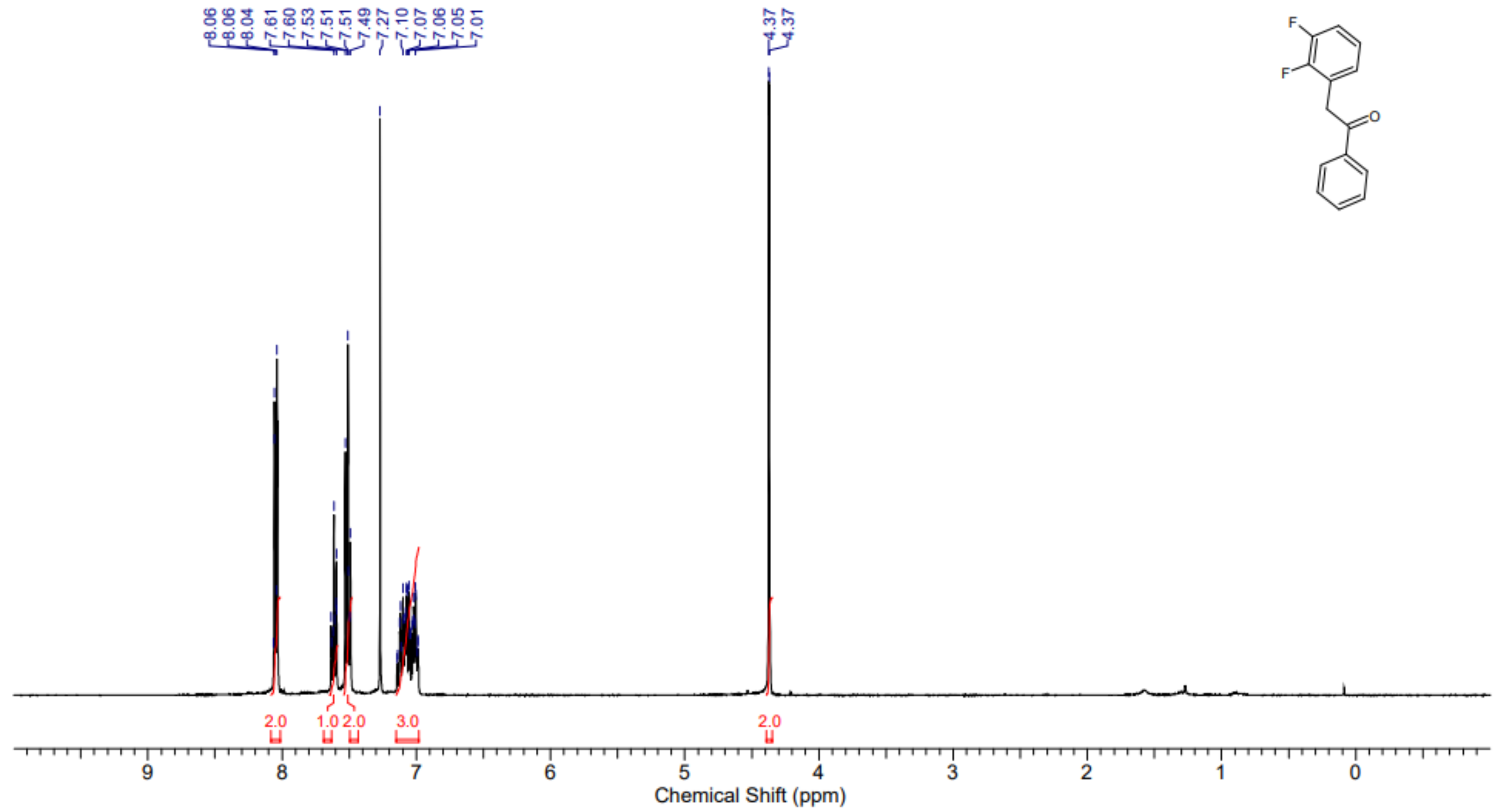

^{13}C NMR (101 MHz, $CDCl_3$)

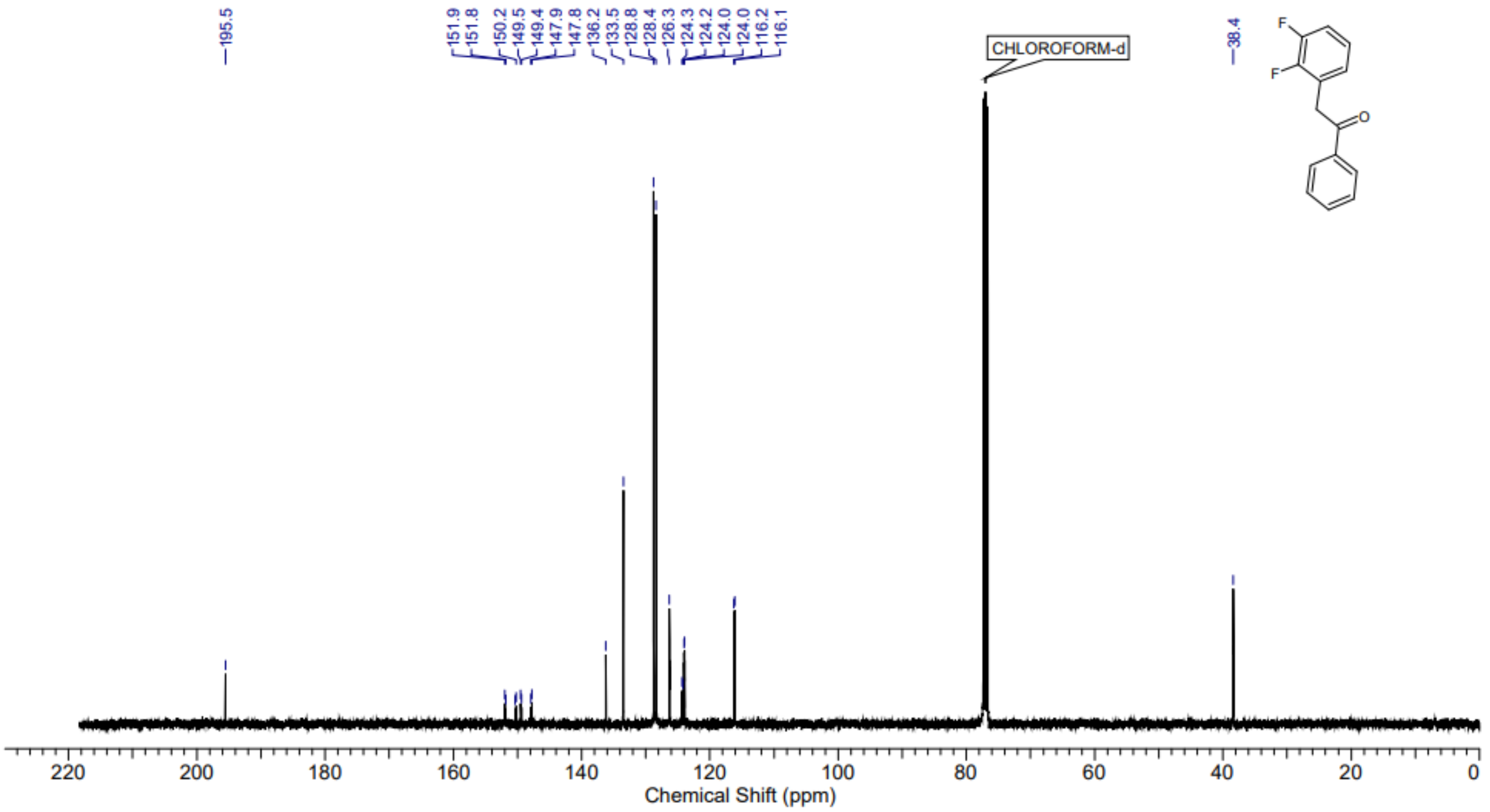

^{19}F NMR (377 MHz, $CDCl_3$)

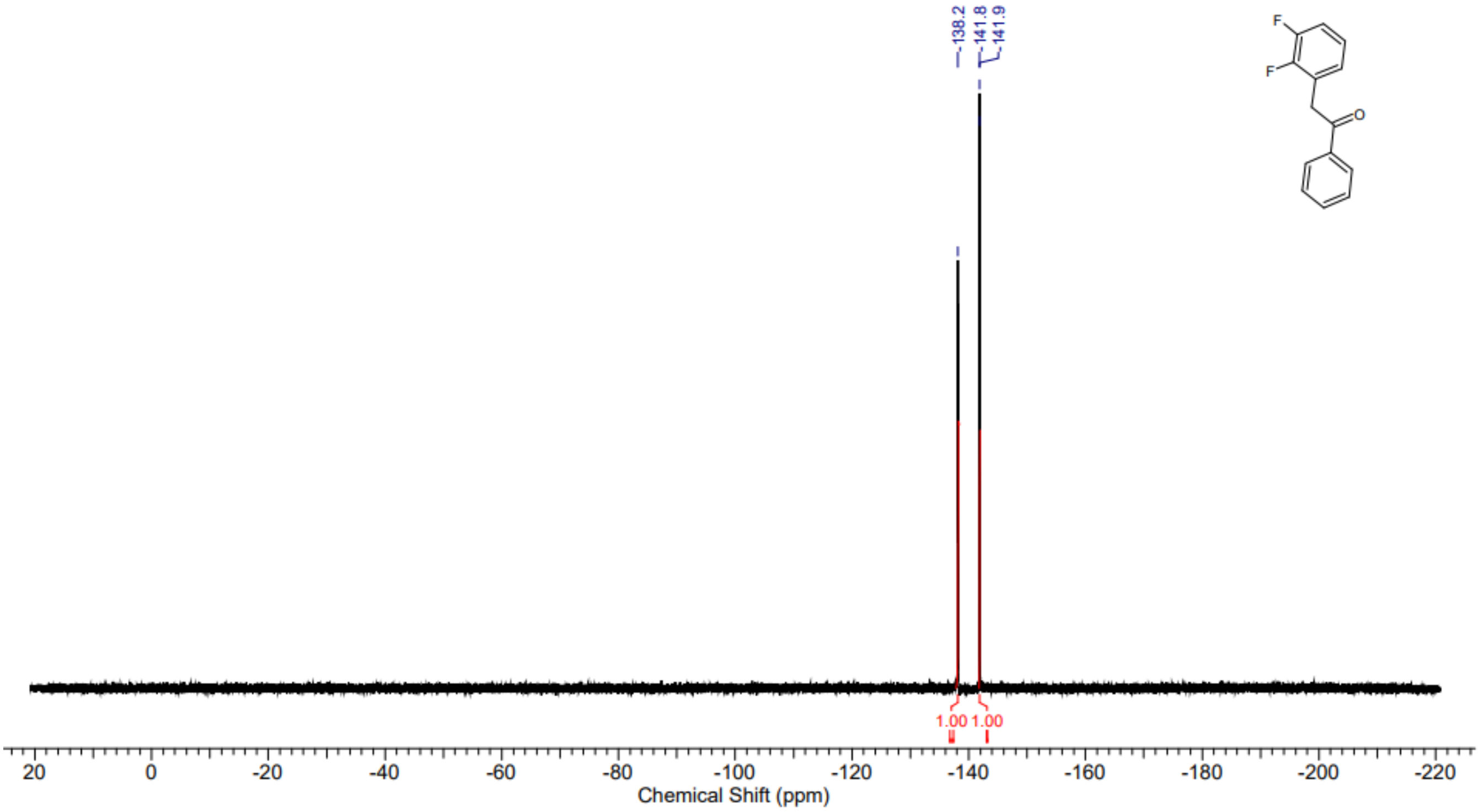

4-(2-Oxo-2-phenylethyl)benzonitrile (**3qa**) [CAS: 59824-23-6]

^1H NMR (400 MHz, $CDCl_3$)

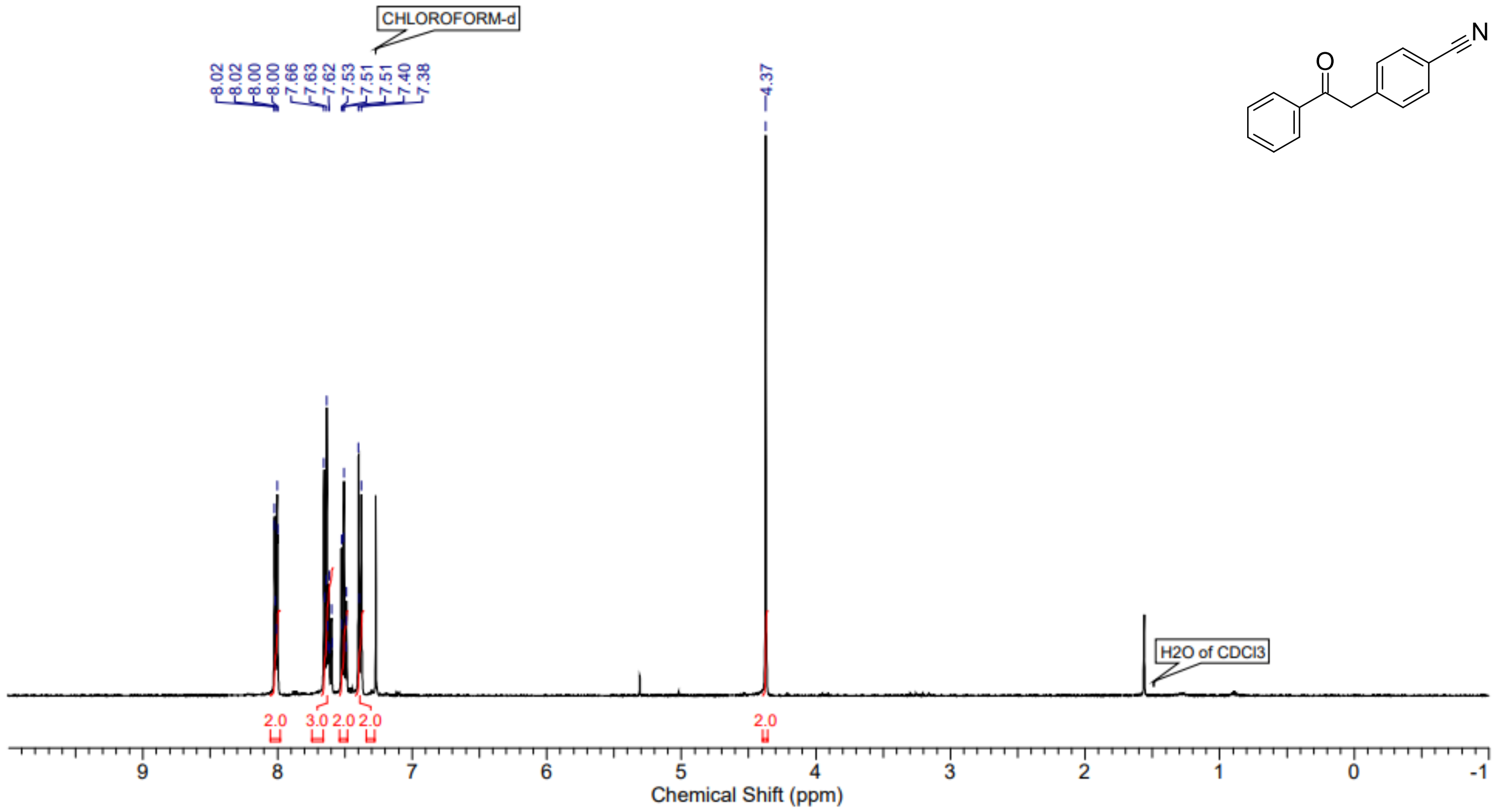

^{13}C NMR (101 MHz, $CDCl_3$)

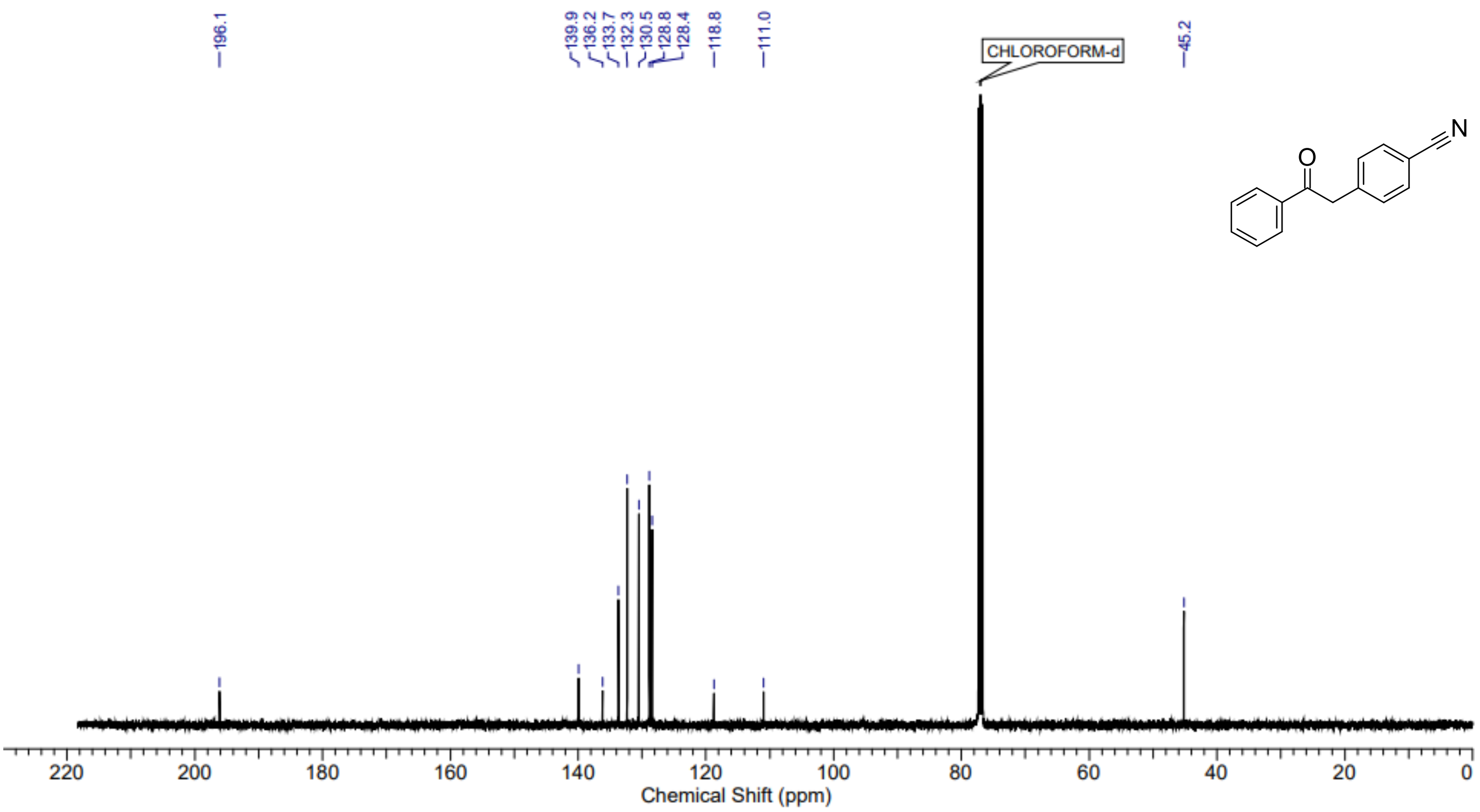

Ethyl 4-(2-oxo-2-phenylethyl)benzoate (**3ra**) [CAS: 898776-62-0]

^{1}H NMR (400 MHz, $CDCl_3$)

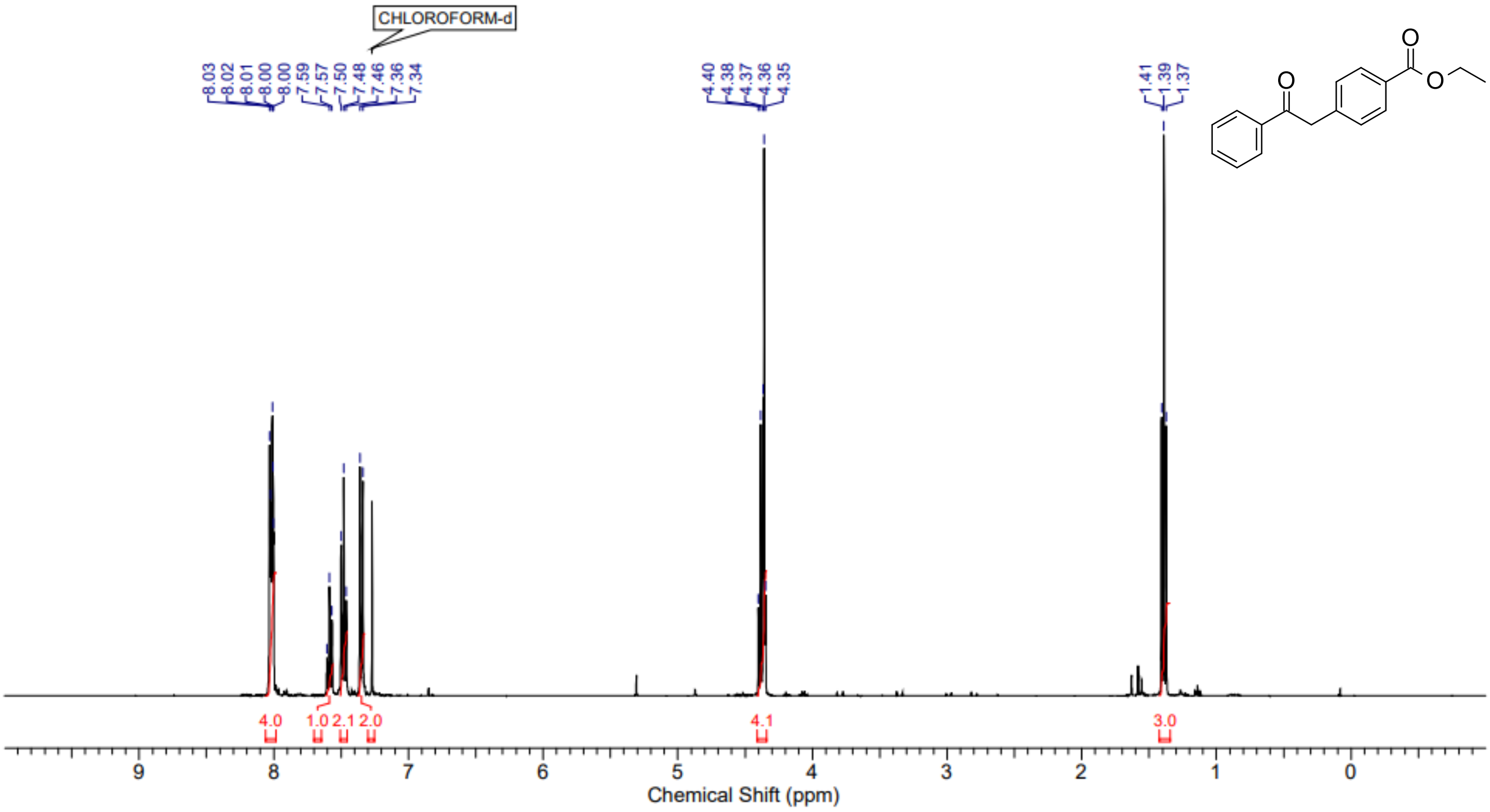

^{13}C NMR (101 MHz, $CDCl_3$)

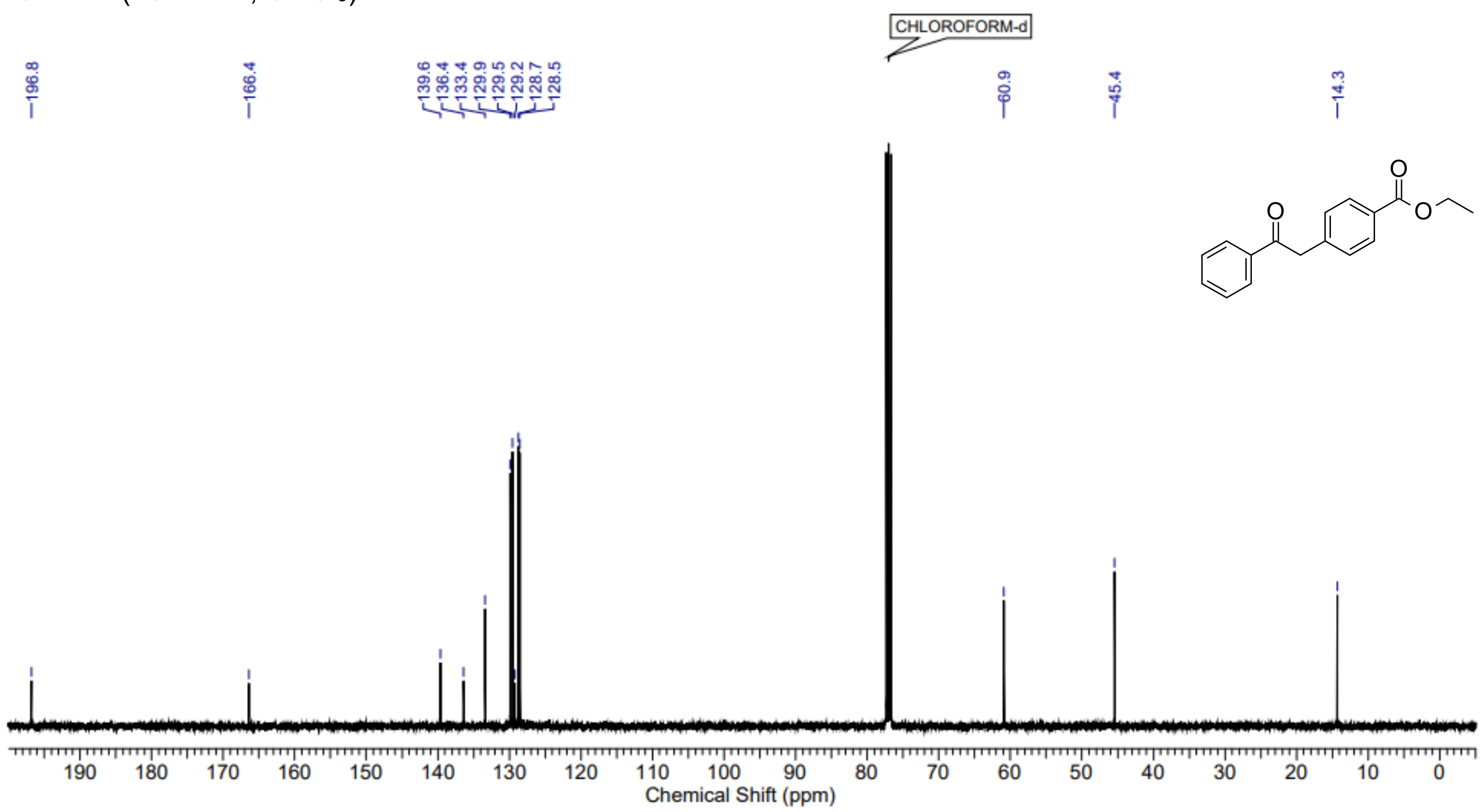

2-[4-[[(1R,2S,5R)-2-isopropyl-5-methyl-cyclohexoxy]methyl]cyclohexa-1,5-dien-1-yl]-1-phenyl-ethanone (**3sa**)

^{1}H NMR (400 MHz, $CDCl_3$)

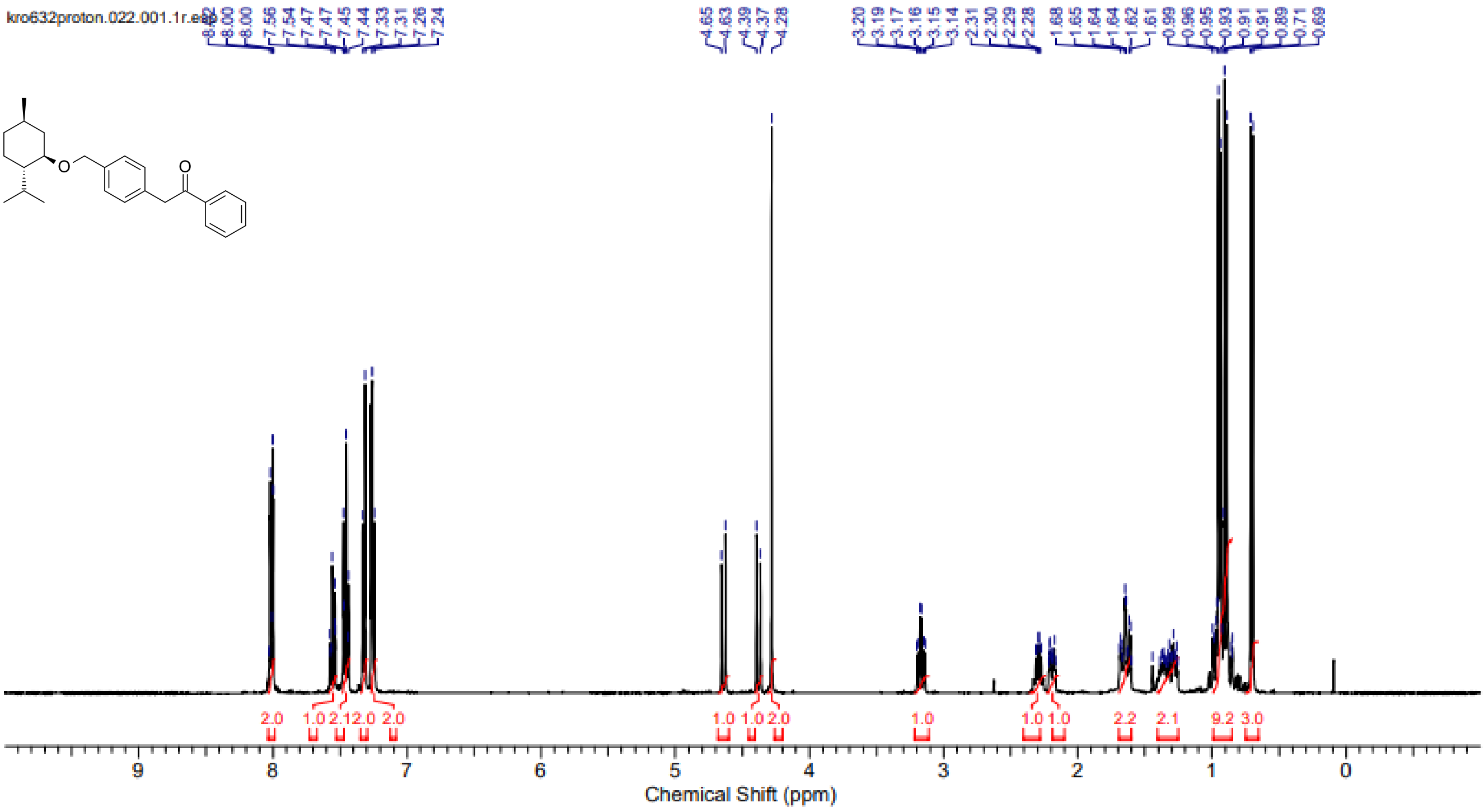

^{13}C NMR (101 MHz, $CDCl_3$)

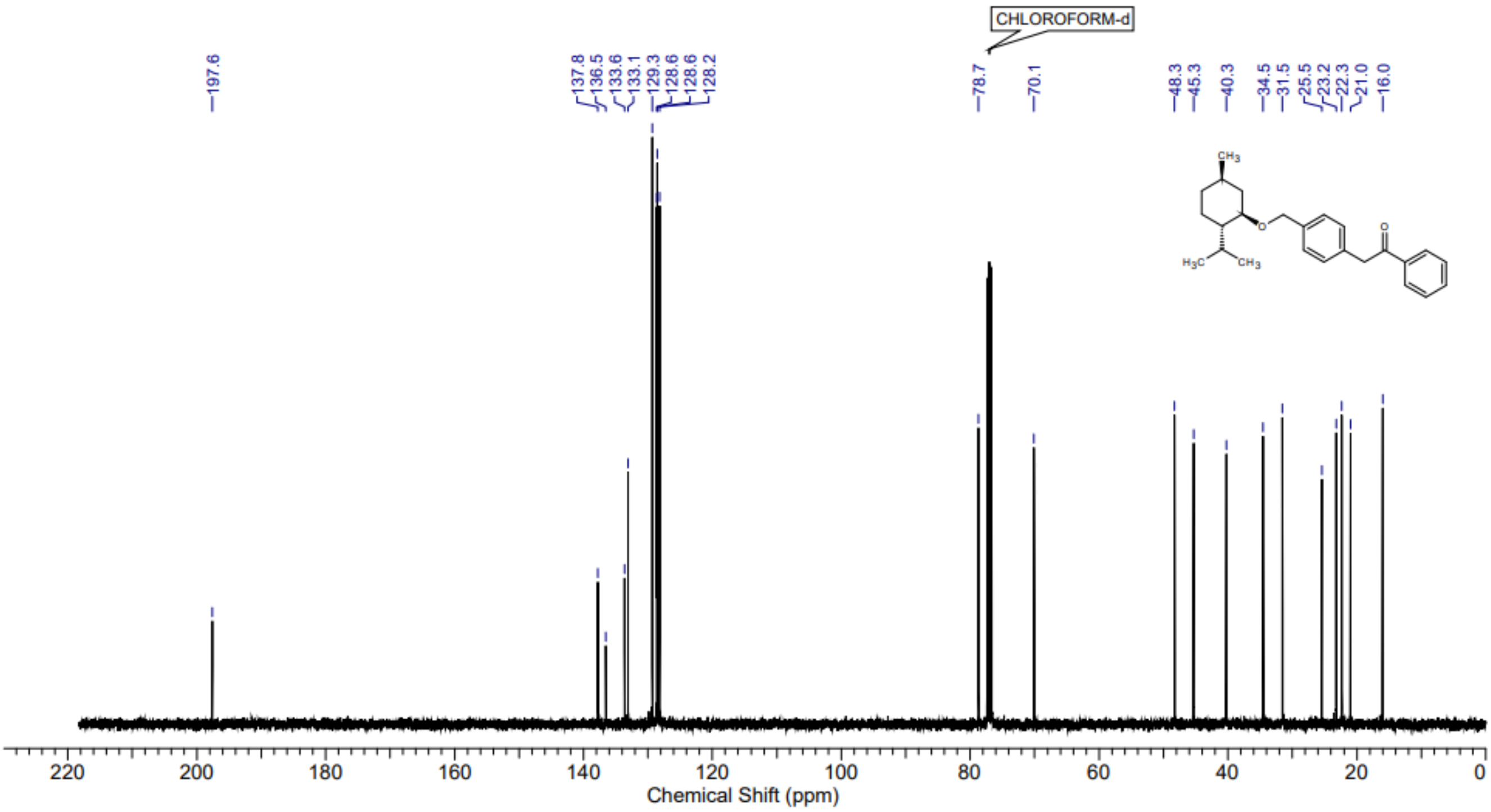

2-[4-[[(3S,8S,9S,10R,13R,14S,17R)-17-[(1R)-1,5-dimethylhexyl]-10,13-dimethyl-2,3,4,7,8,9,11,12,14,15,16,17-dodecahydro-1H-cyclopenta[a]phenanthren-3-yl]oxymethyl]phenyl]-1-phenyl-ethanone (**3ta**)

^{1}H NMR (400 MHz, $CDCl_3$)

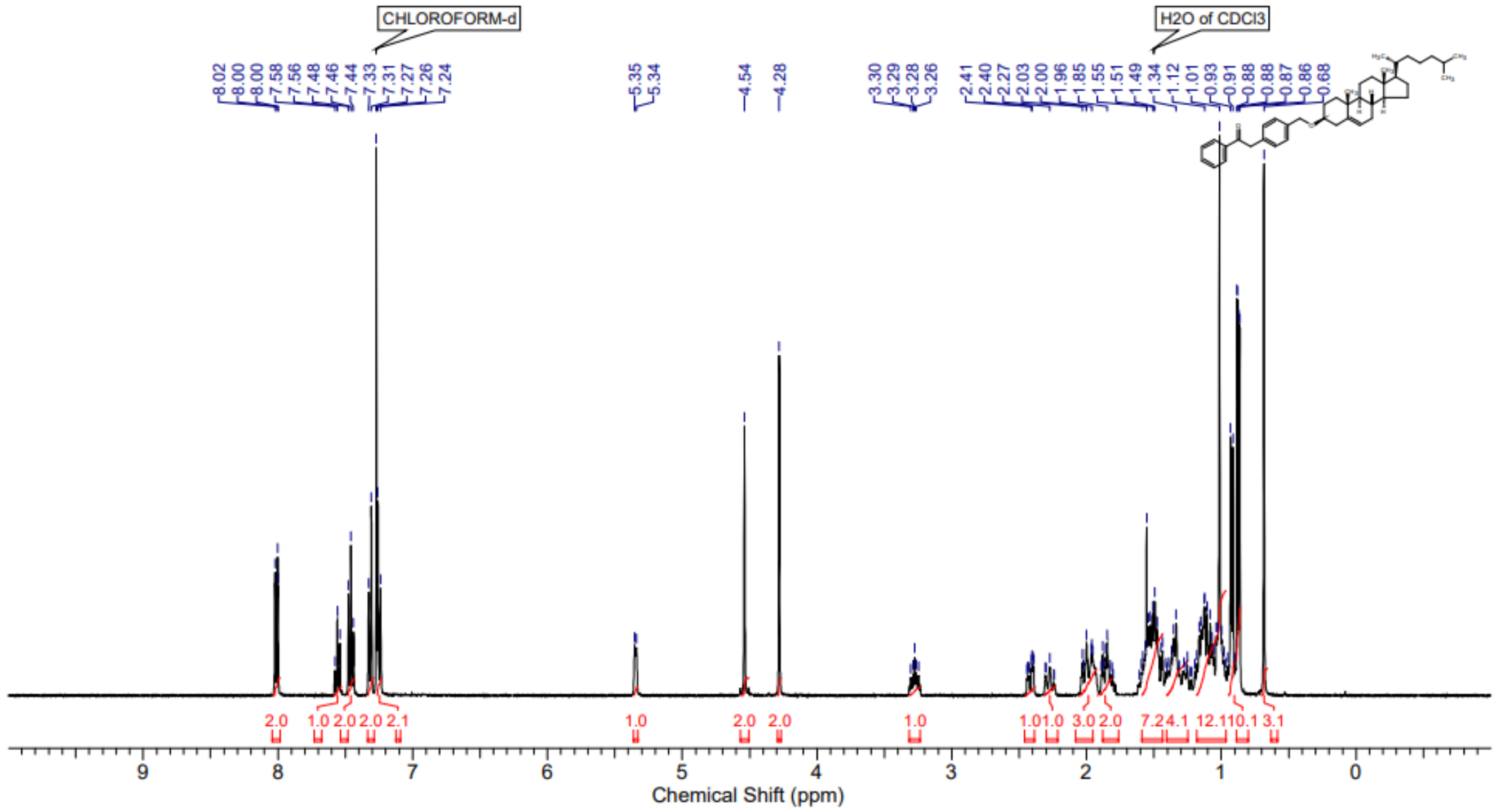

^{13}C NMR (101 MHz, $CDCl_3$)

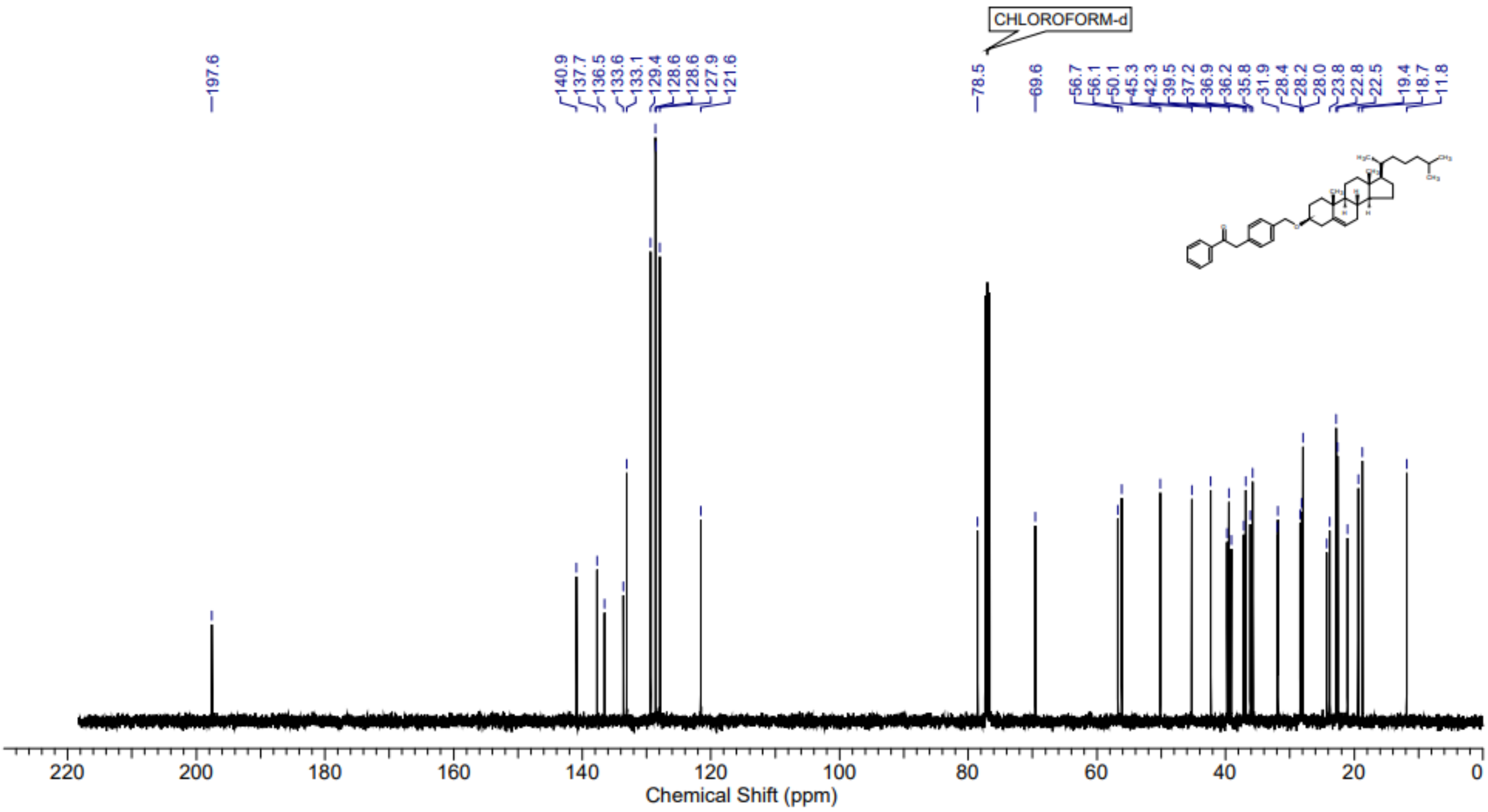

1-phenyl-2-[4-[4-(4-propylcyclohexyl)cyclohexyl]phenyl]ethanone (**3ua**)

^{1}H NMR (400 MHz, $CDCl_3$)

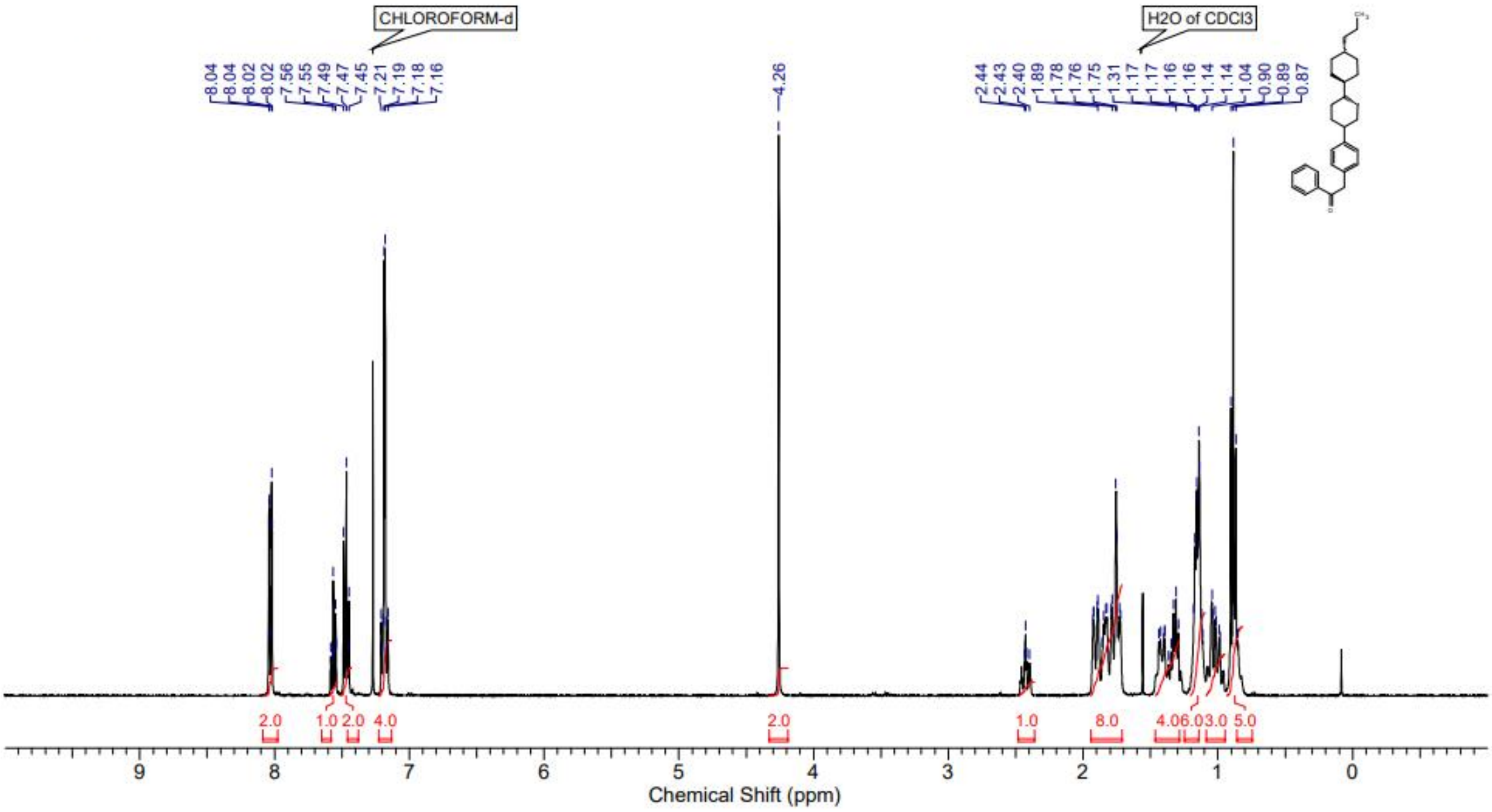

^{13}C NMR (101 MHz, $CDCl_3$)

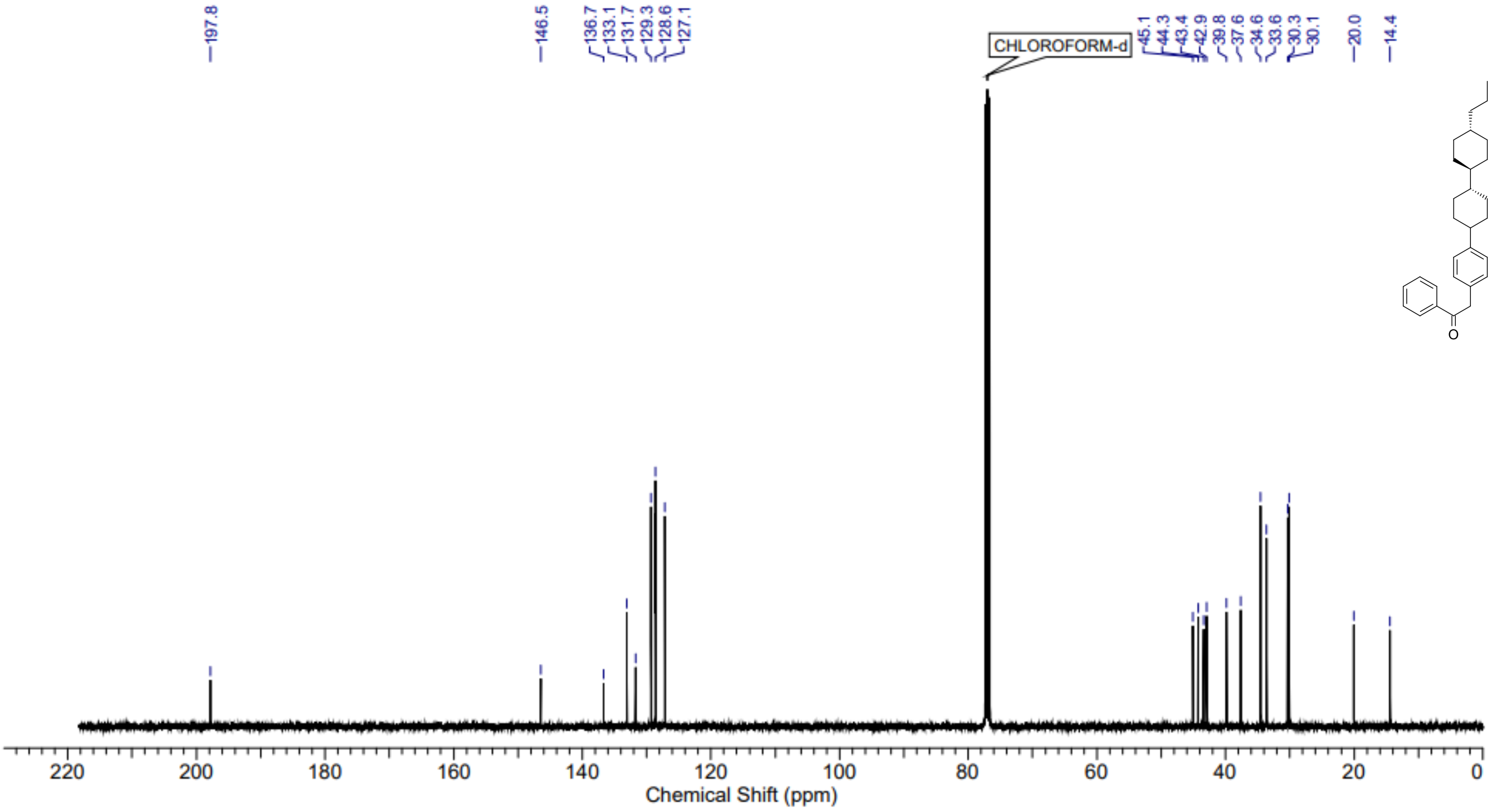

2-[4-(4-morpholinyl)phenyl]-1-phenyl-]ethanone (**3va**) [CAS: 884504-56-7]

^{1}H NMR (400 MHz, $CDCl_3$)

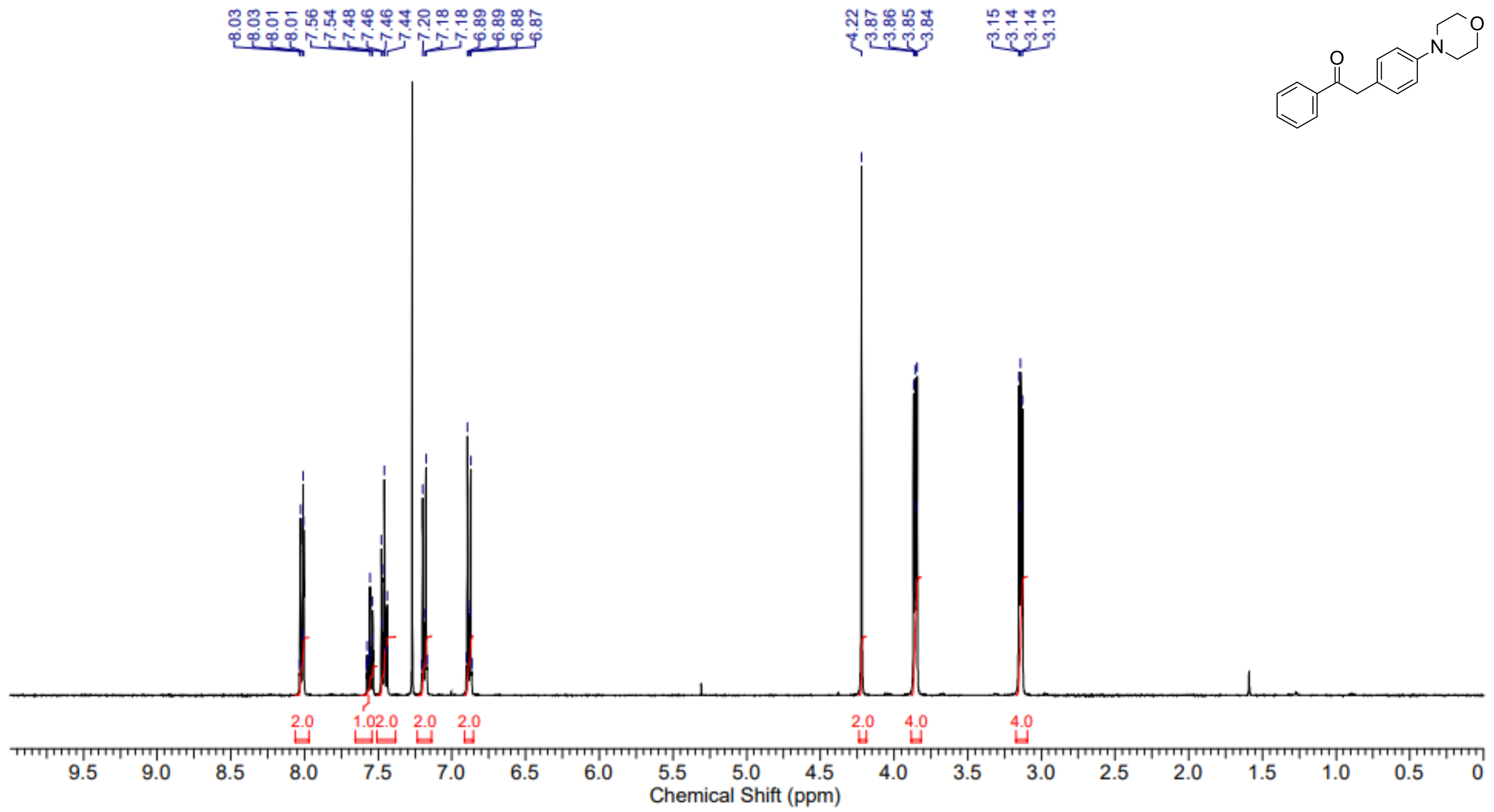

^{13}C NMR (101 MHz, $CDCl_3$)

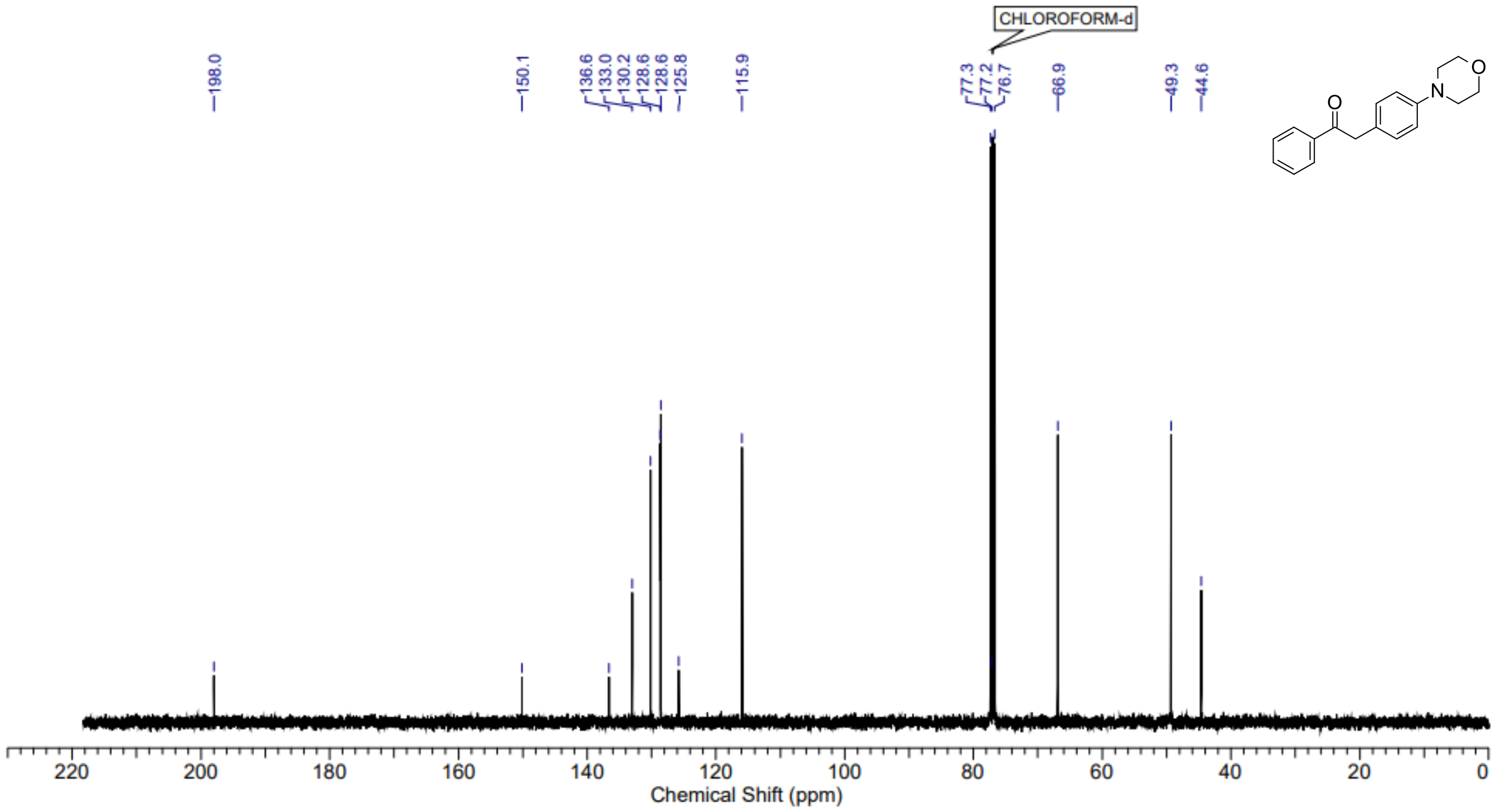

2-[1,1’-Biphenyl]-4-yl-1-phenylethanone (**3wa**) [CAS: 27644-00-4]

^{1}H NMR (400 MHz, $CDCl_3$)

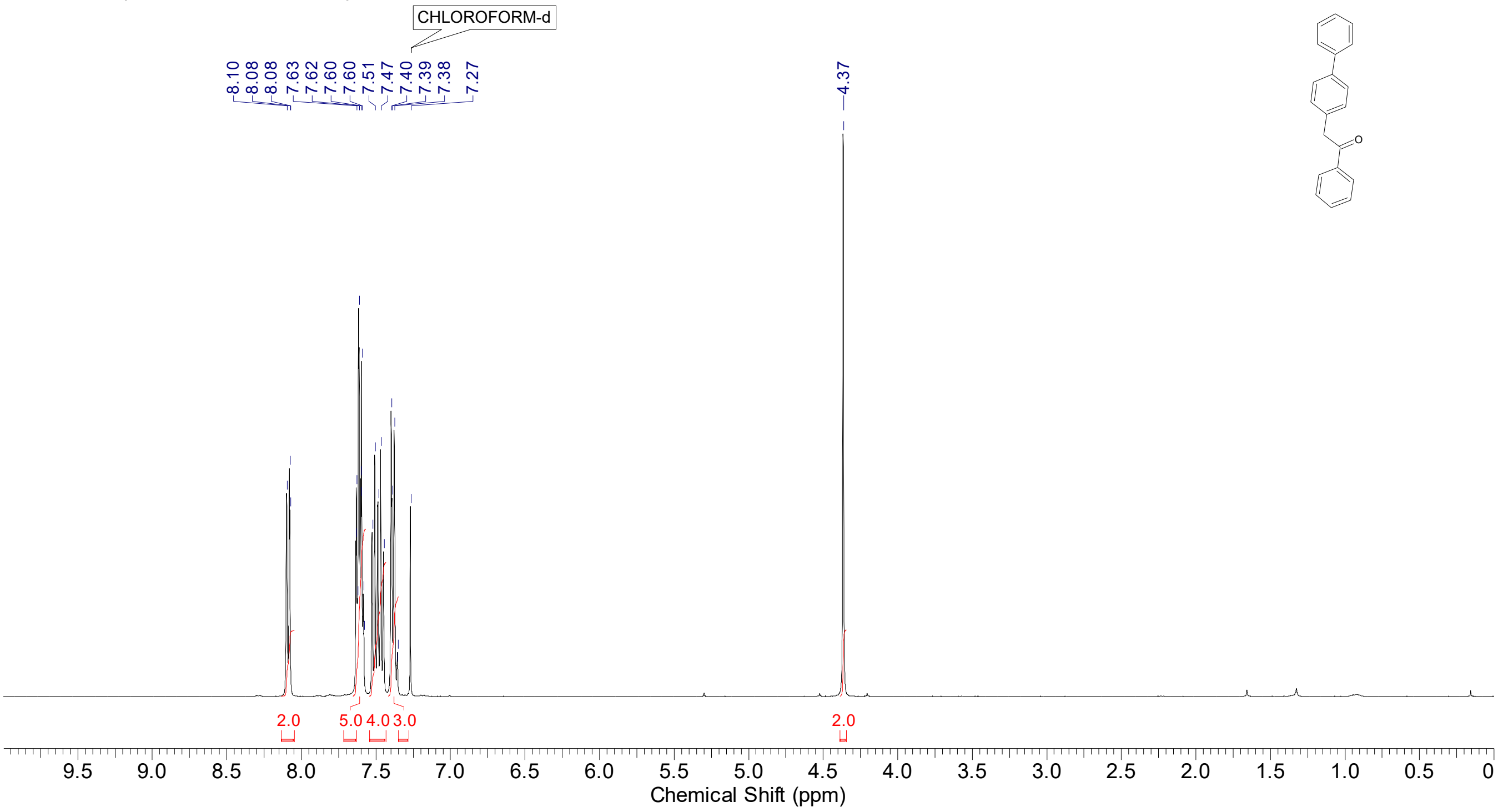

^{13}C NMR (101 MHz, $CDCl_3$)

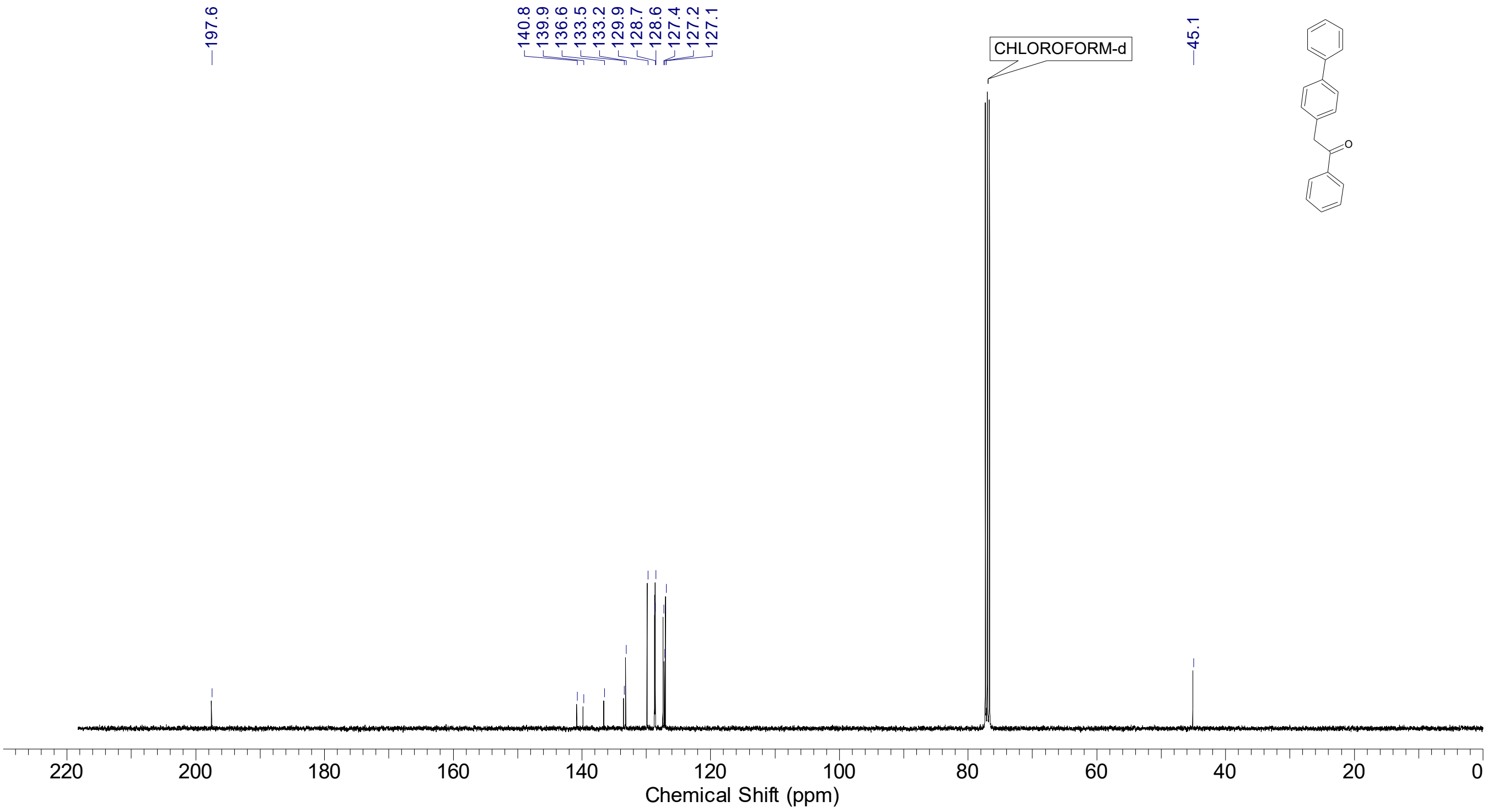

1,2-Bis(4-methoxyphenyl)ethanone (**3ab**) [CAS: 120-44-5]

^{1}H NMR (400 MHz, $CDCl_3$)

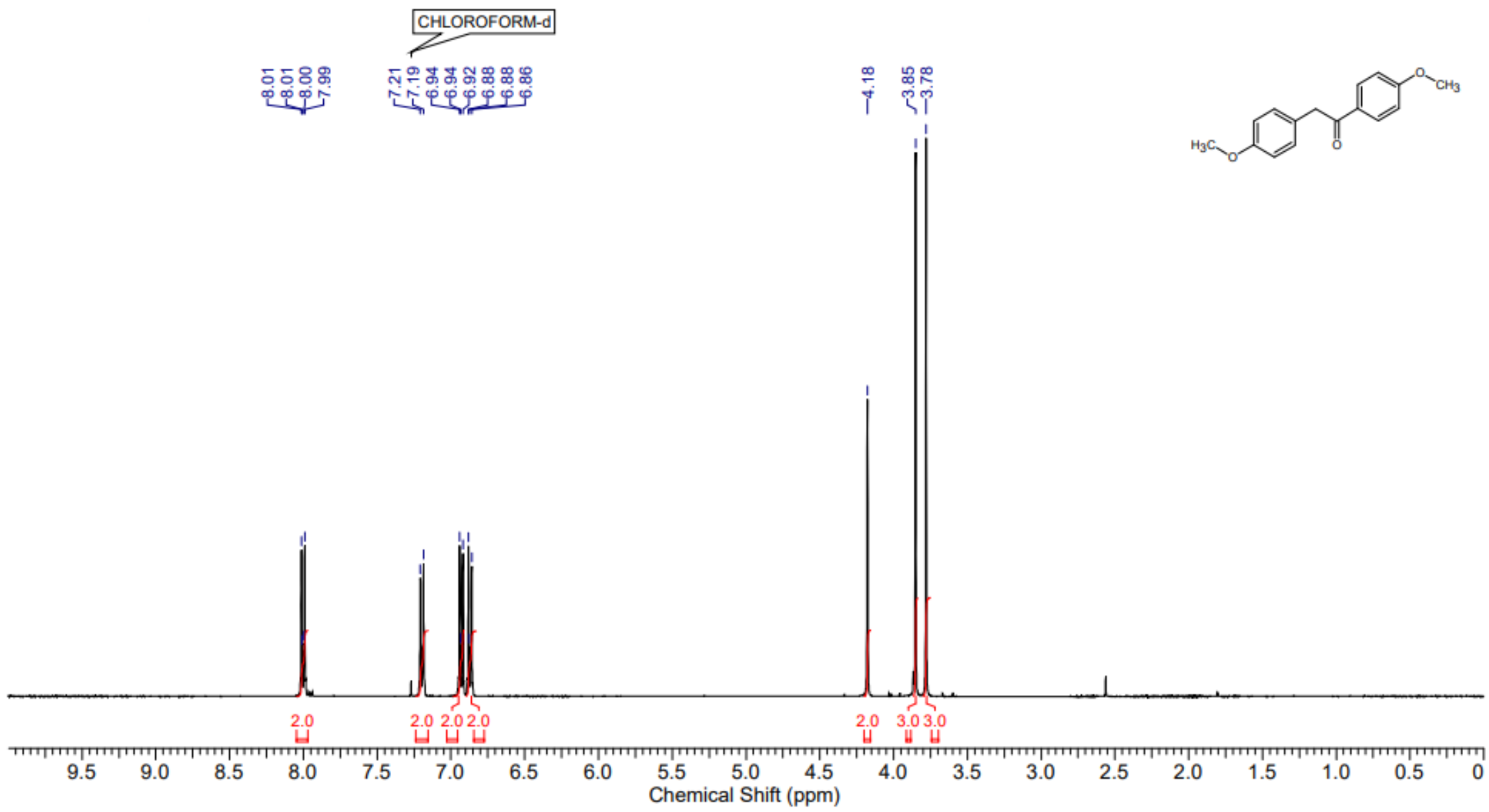

^{13}C NMR (101 MHz, $CDCl_3$)

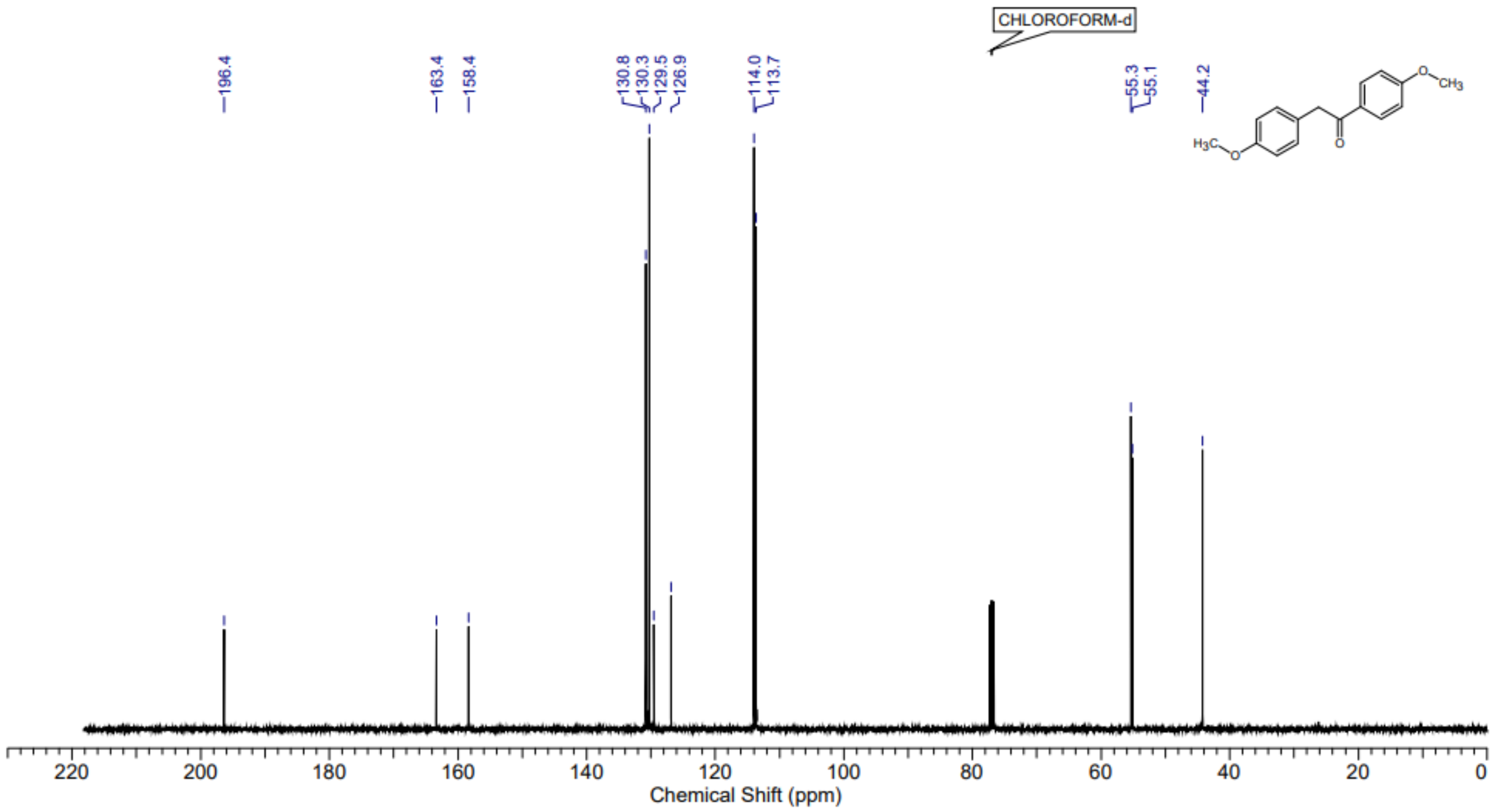

1-[4-(Dimethylamino)phenyl]-2-(4-methoxyphenyl)ethanone (**3ac**) [CAS: 97606-30-9]

^{1}H NMR (400 MHz, $CDCl_3$)

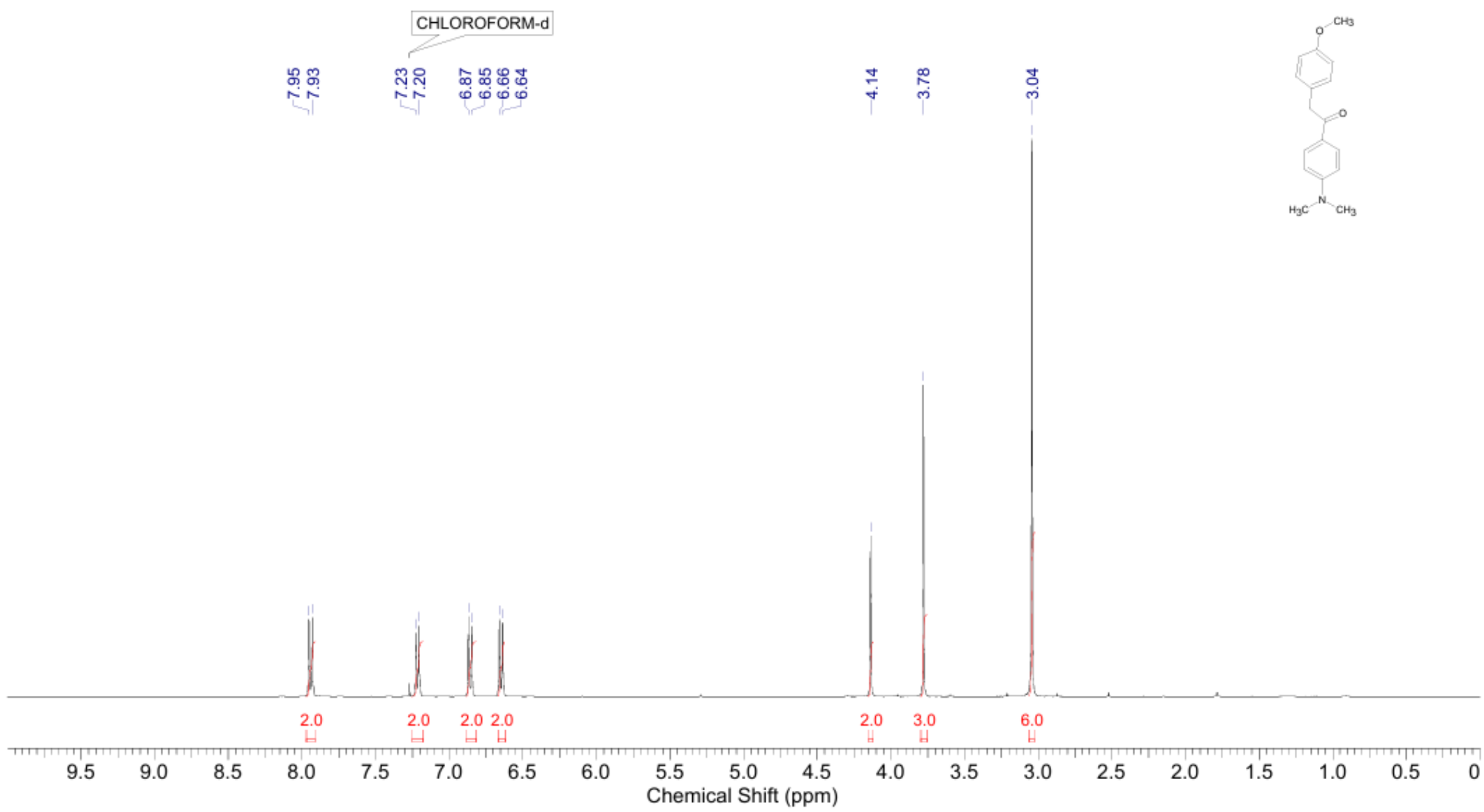

^{13}C NMR (101 MHz, $CDCl_3$)

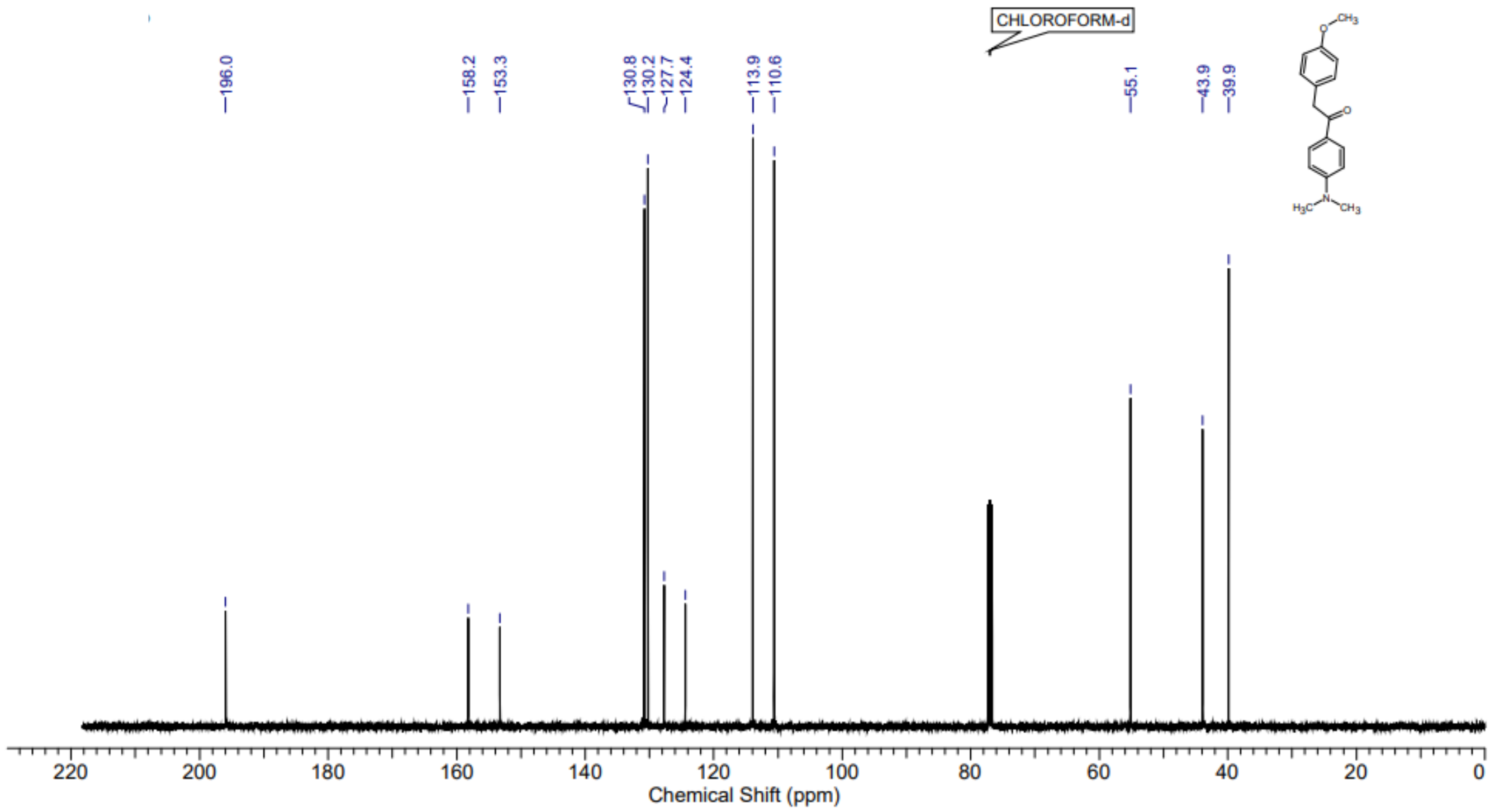

2-(4-Methoxyphenyl)-1-(2-thienyl)ethanone (**3ad**) [CAS: 67947-62-0]

^{1}H NMR (400 MHz, $CDCl_3$)

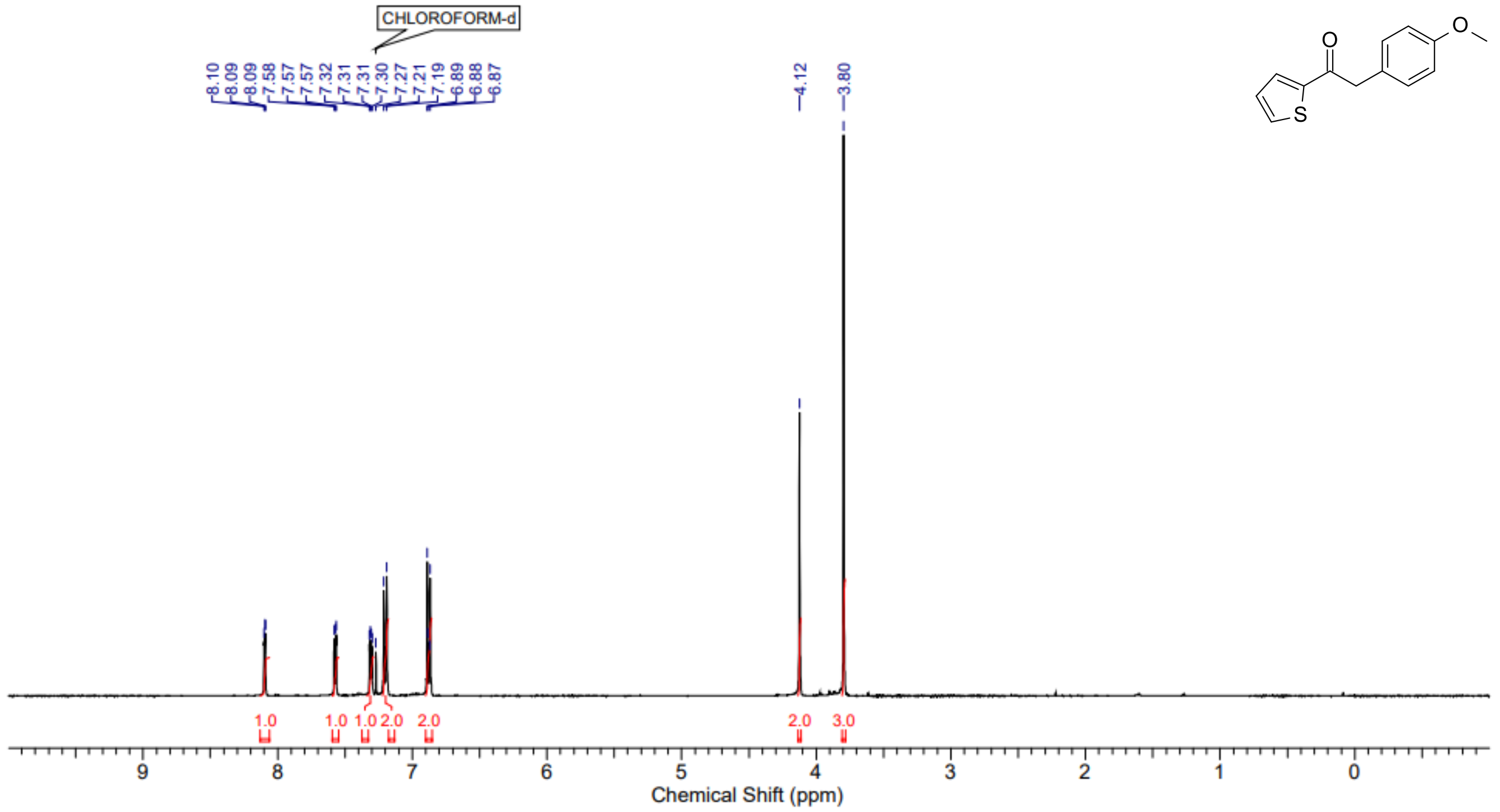

^{13}C NMR (101 MHz, $CDCl_3$)

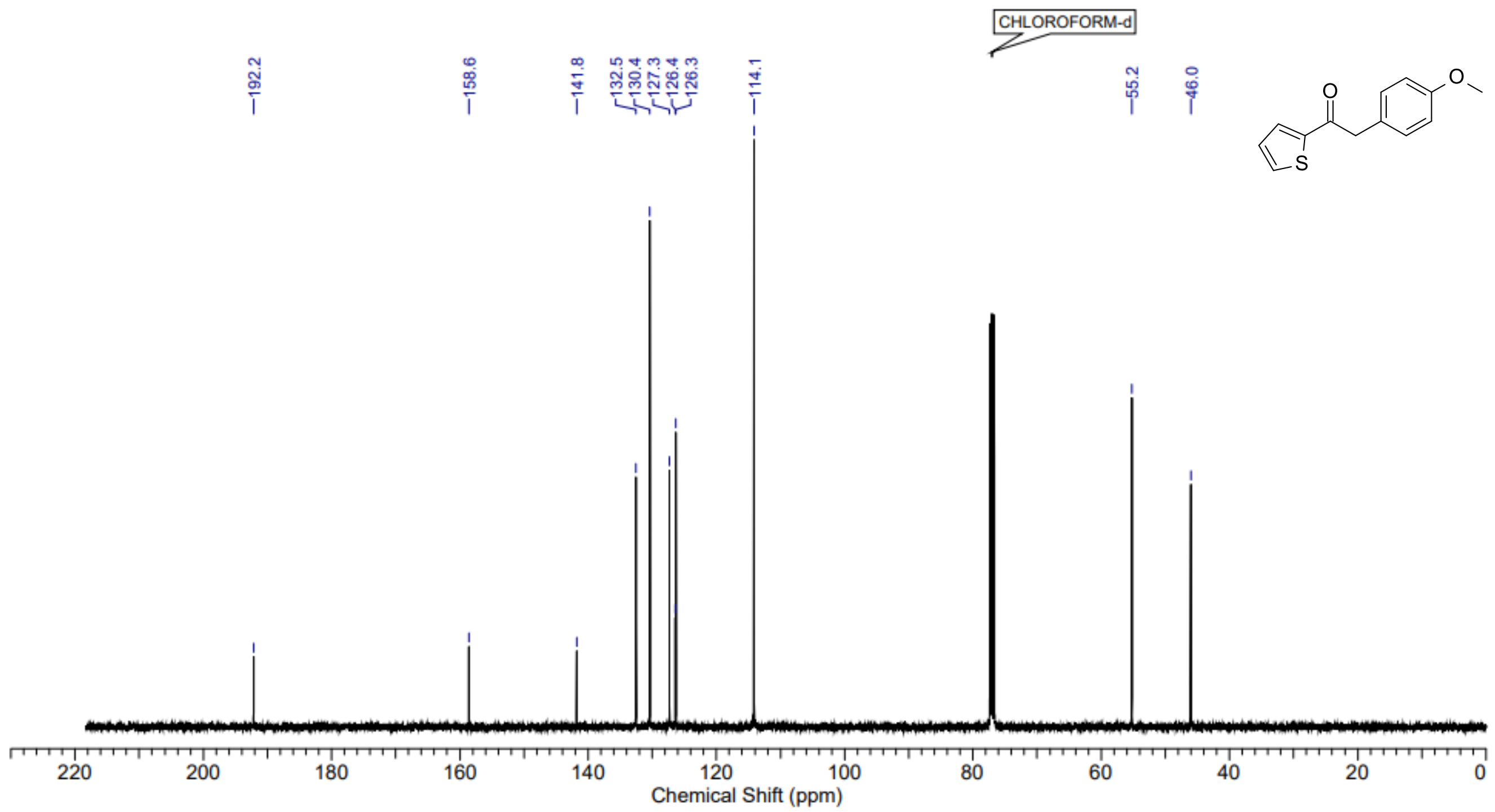

2-(4-Methoxyphenyl)-1-(4-methylphenyl)ethanone (**3ae**) [CAS: 345617-51-8]

^{1}H NMR (400 MHz, $CDCl_3$)

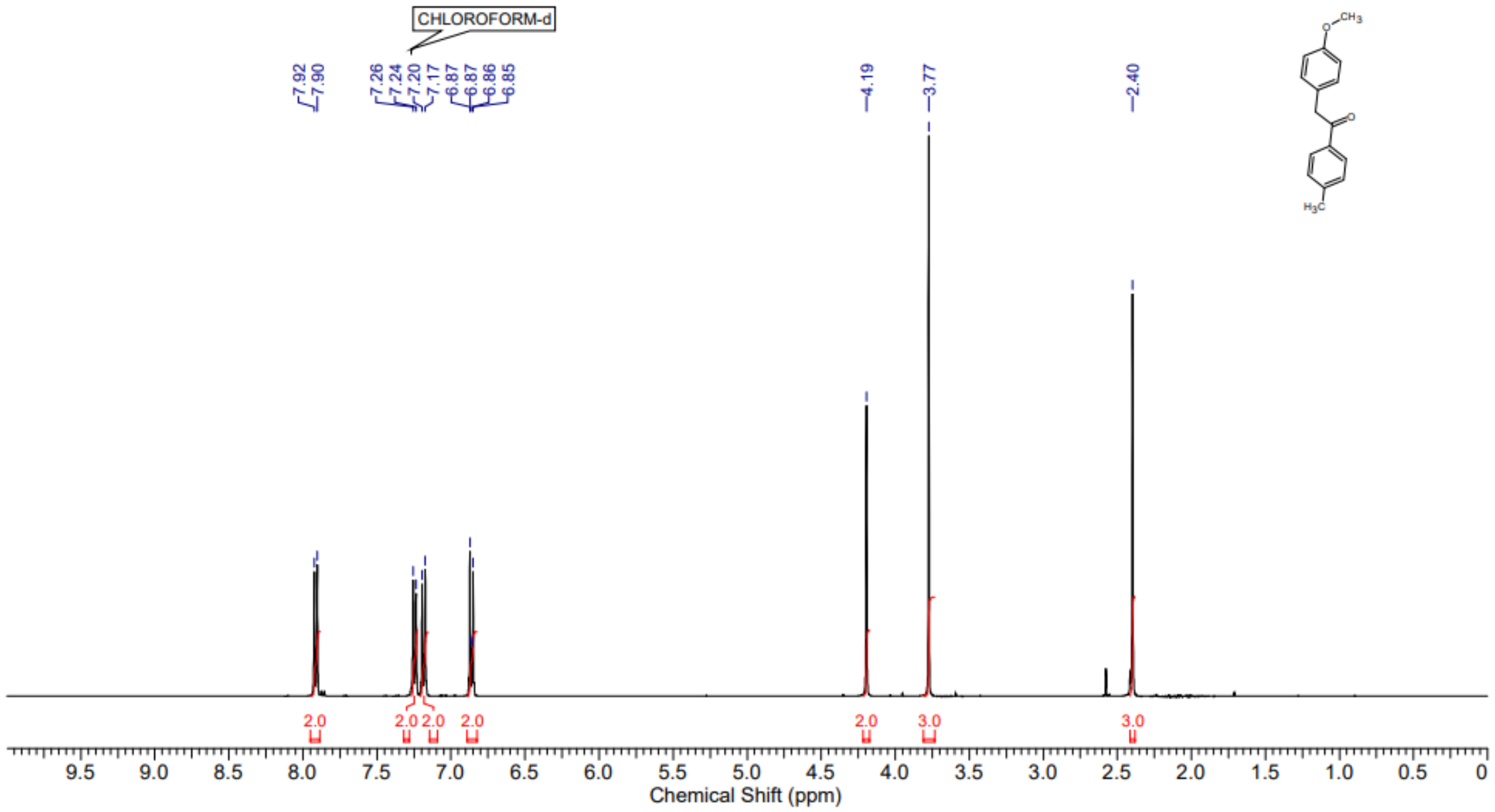

^{13}C NMR (101 MHz, $CDCl_3$)

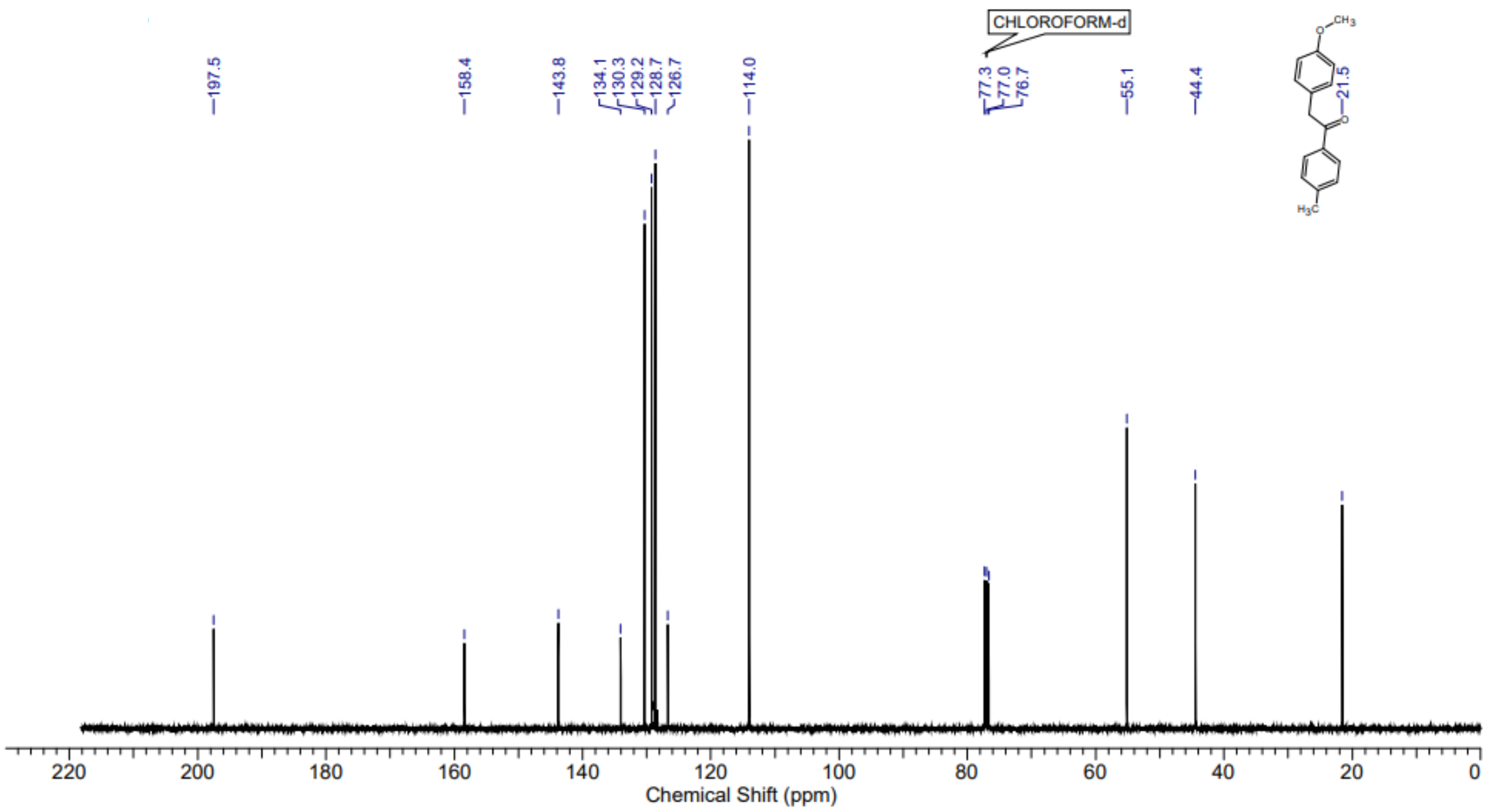

2-(4-Methoxyphenyl)-1-phenyl-1-propanone (**3af**) [CAS: 84839-92-9]

^{1}H NMR (400 MHz, $CDCl_3$)

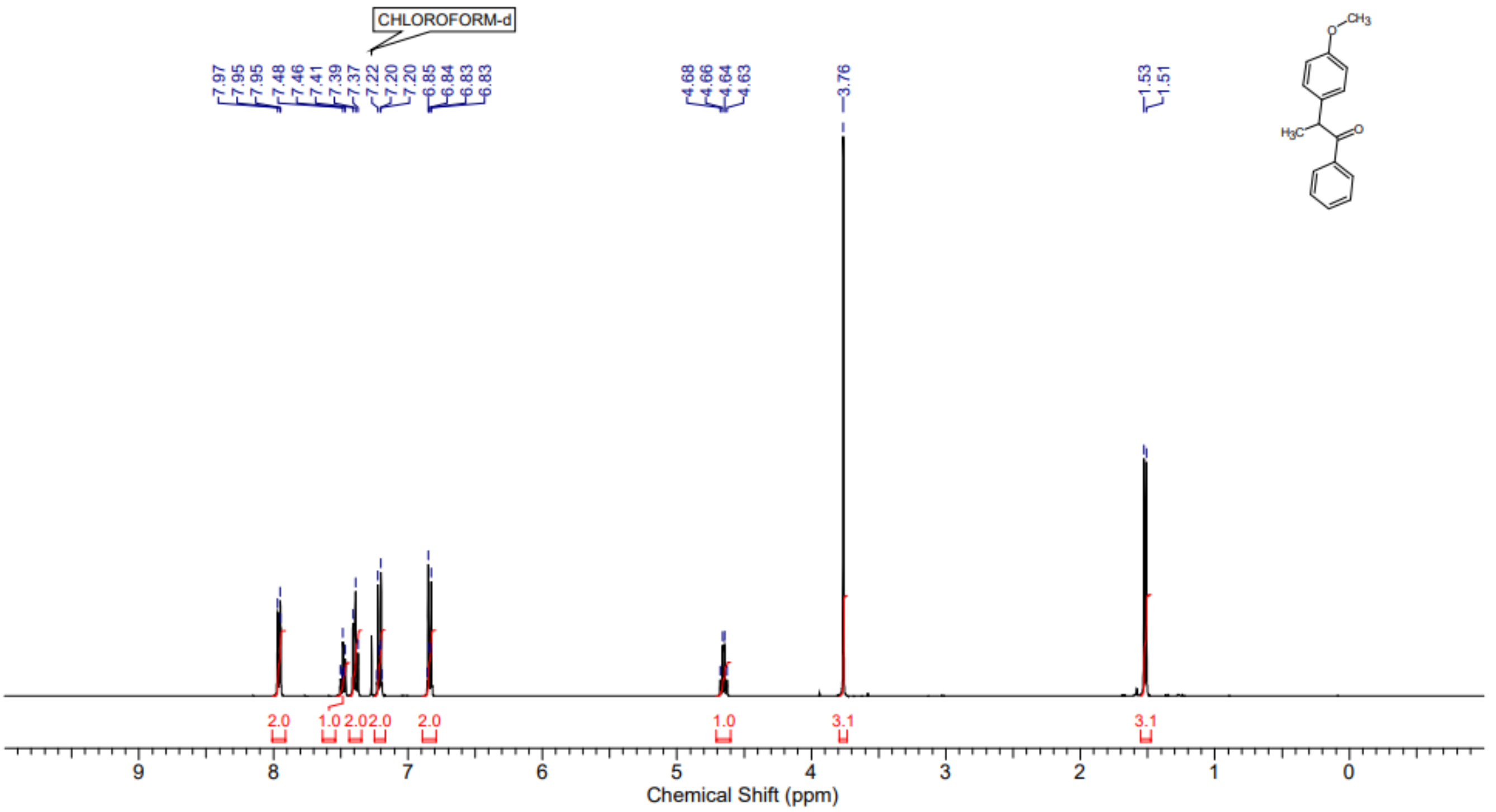

^{13}C NMR (101 MHz, $CDCl_3$)

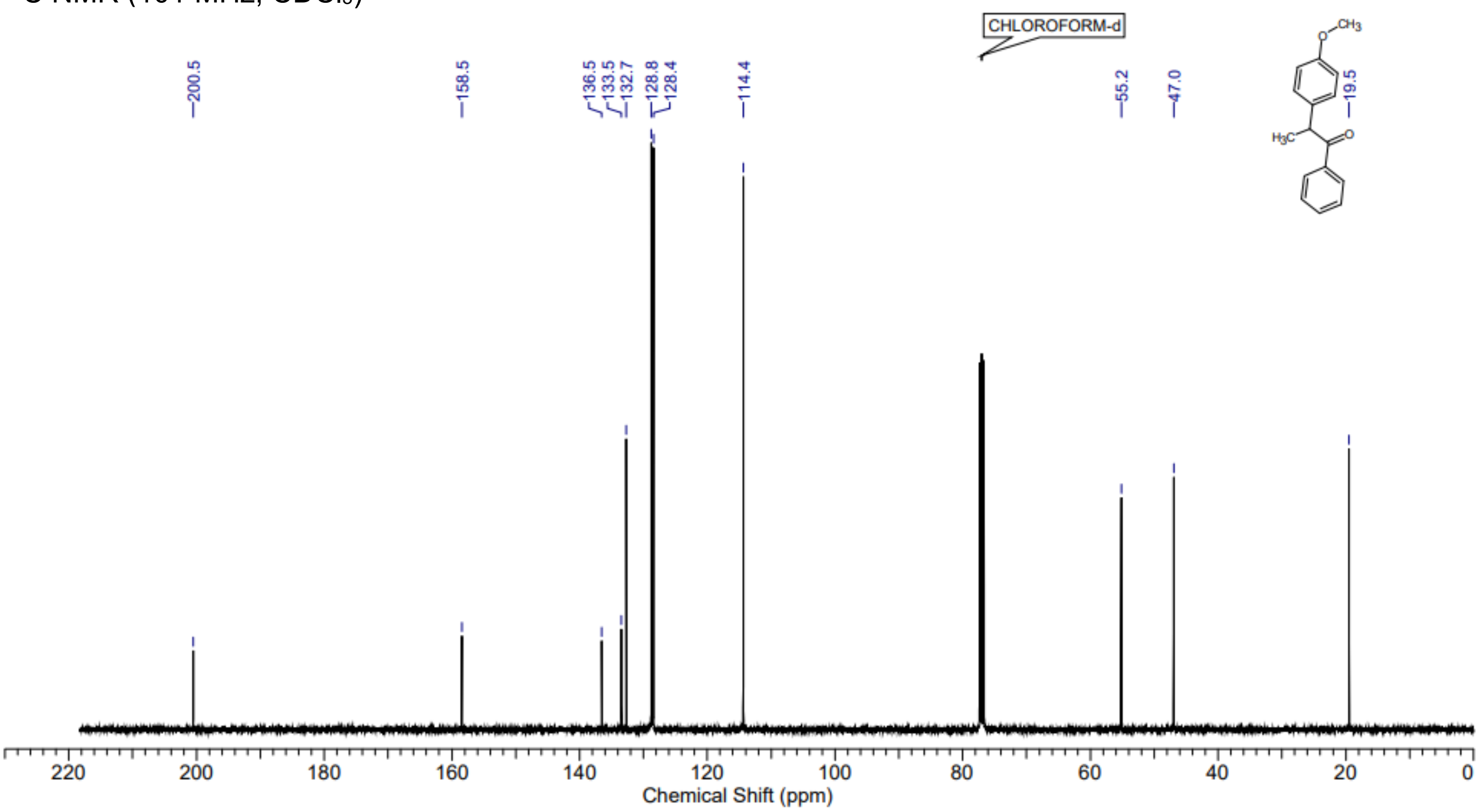

2-(4-Methoxyphenyl)-1-(2-naphthalenyl)ethanone (**3ag**) [CAS: 1184749-94-7]

^{1}H NMR (400 MHz, $CDCl_3$)

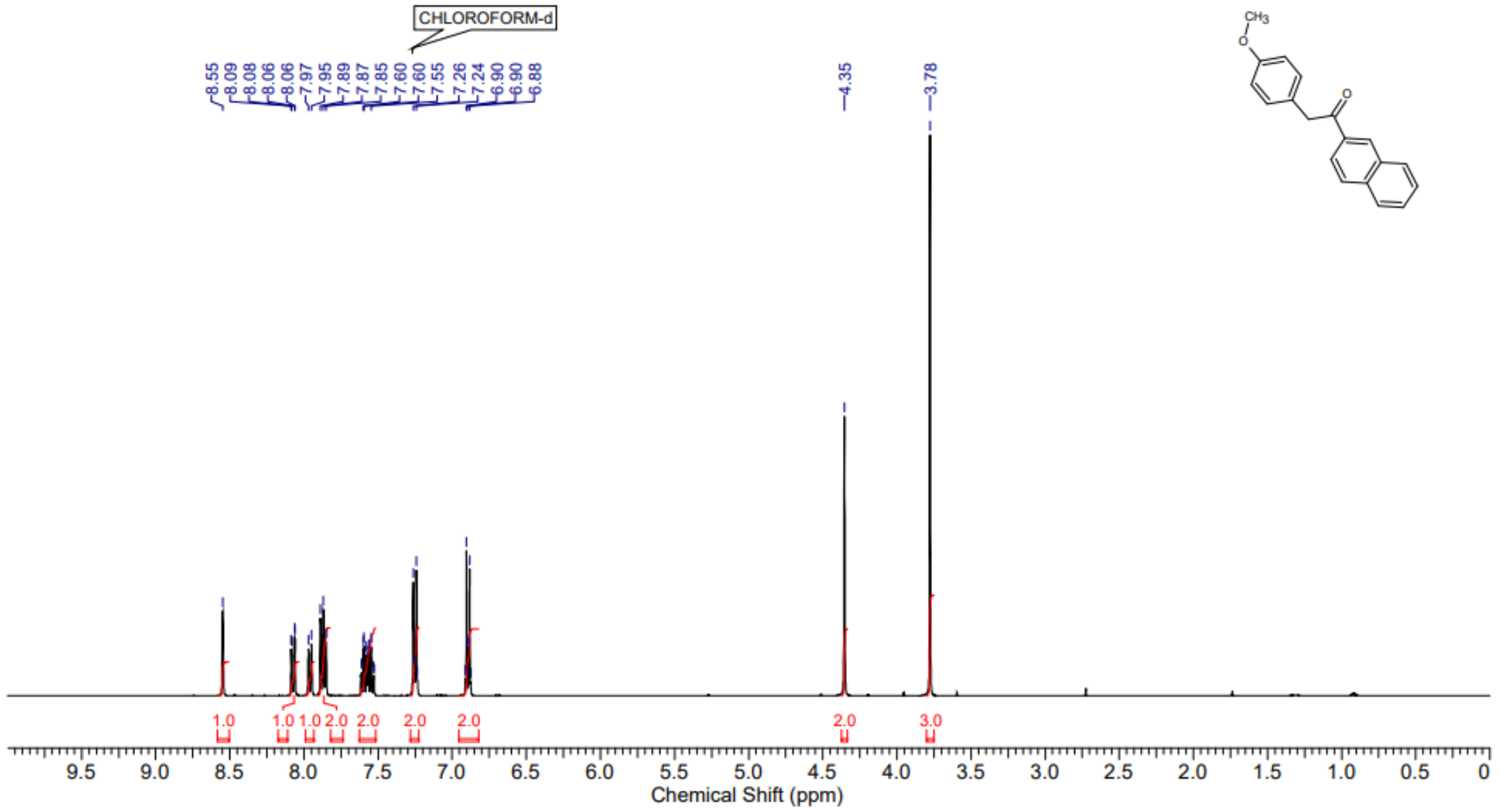

^{13}C NMR (101 MHz, $CDCl_3$)

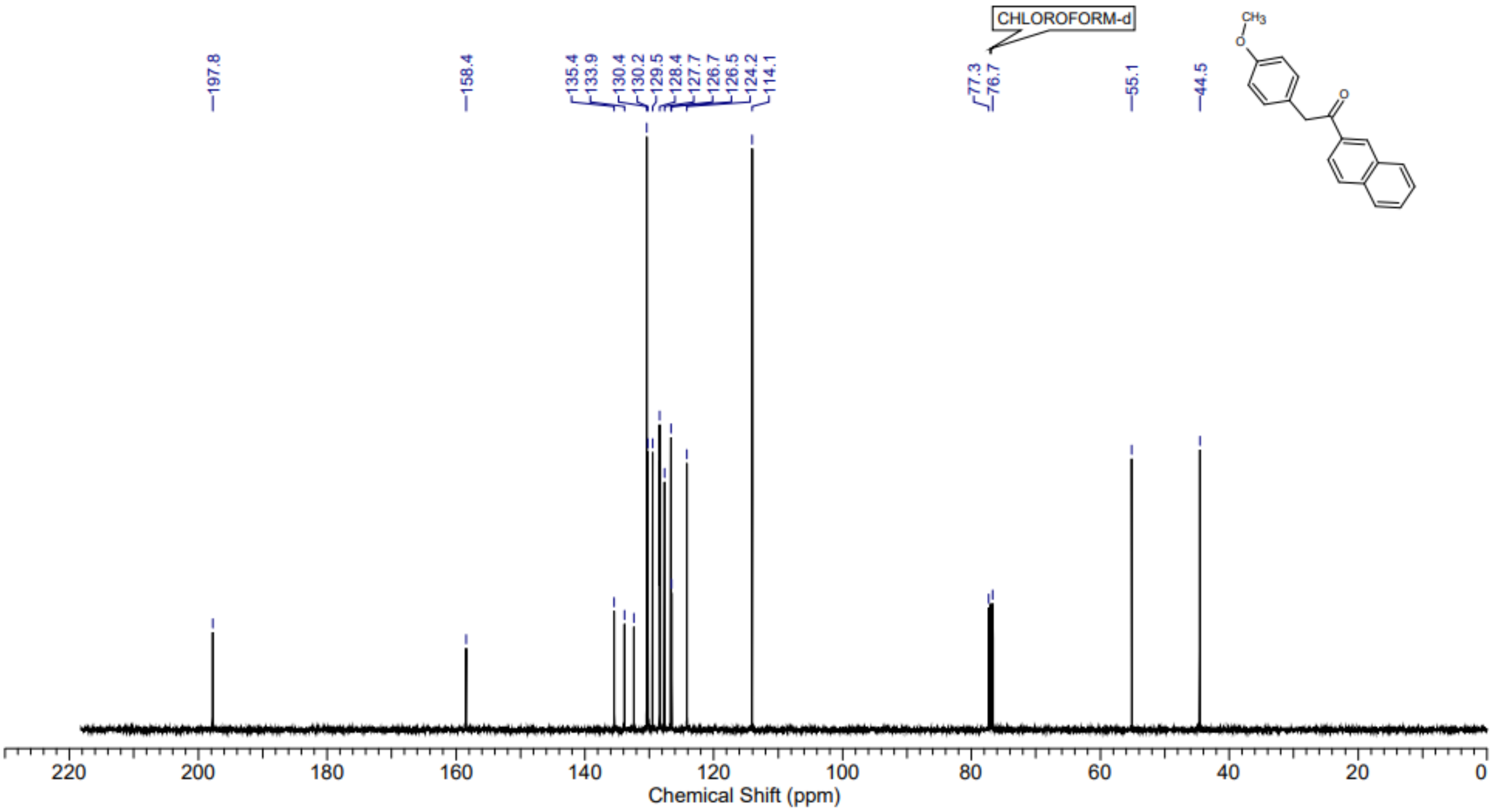

2-(4-Methoxyphenyl)cyclohexanone (**3ah**) [CAS: 37087-68-6]

^{1}H NMR (400 MHz, $CDCl_3$)

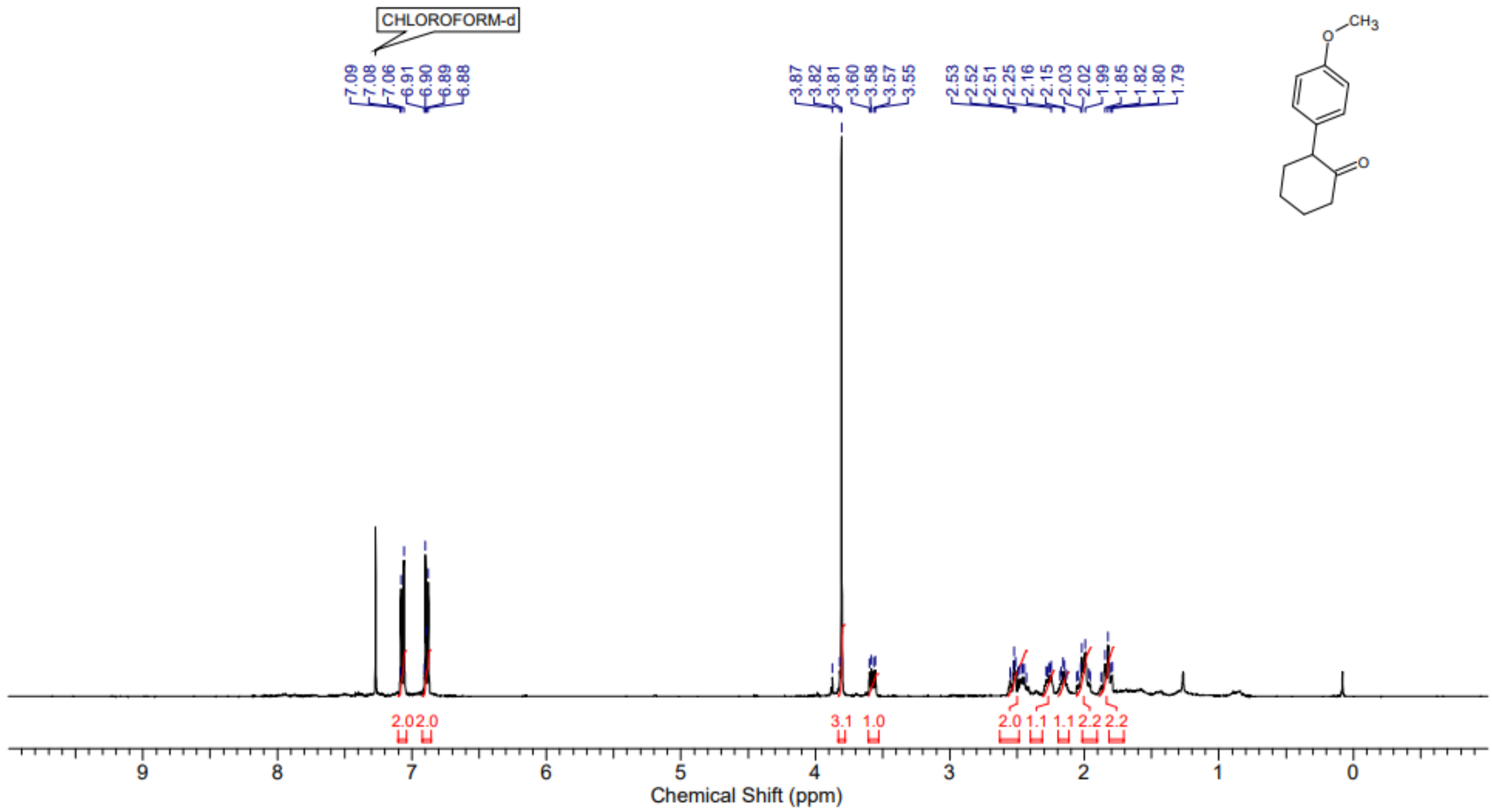

^{13}C NMR (101 MHz, $CDCl_3$)

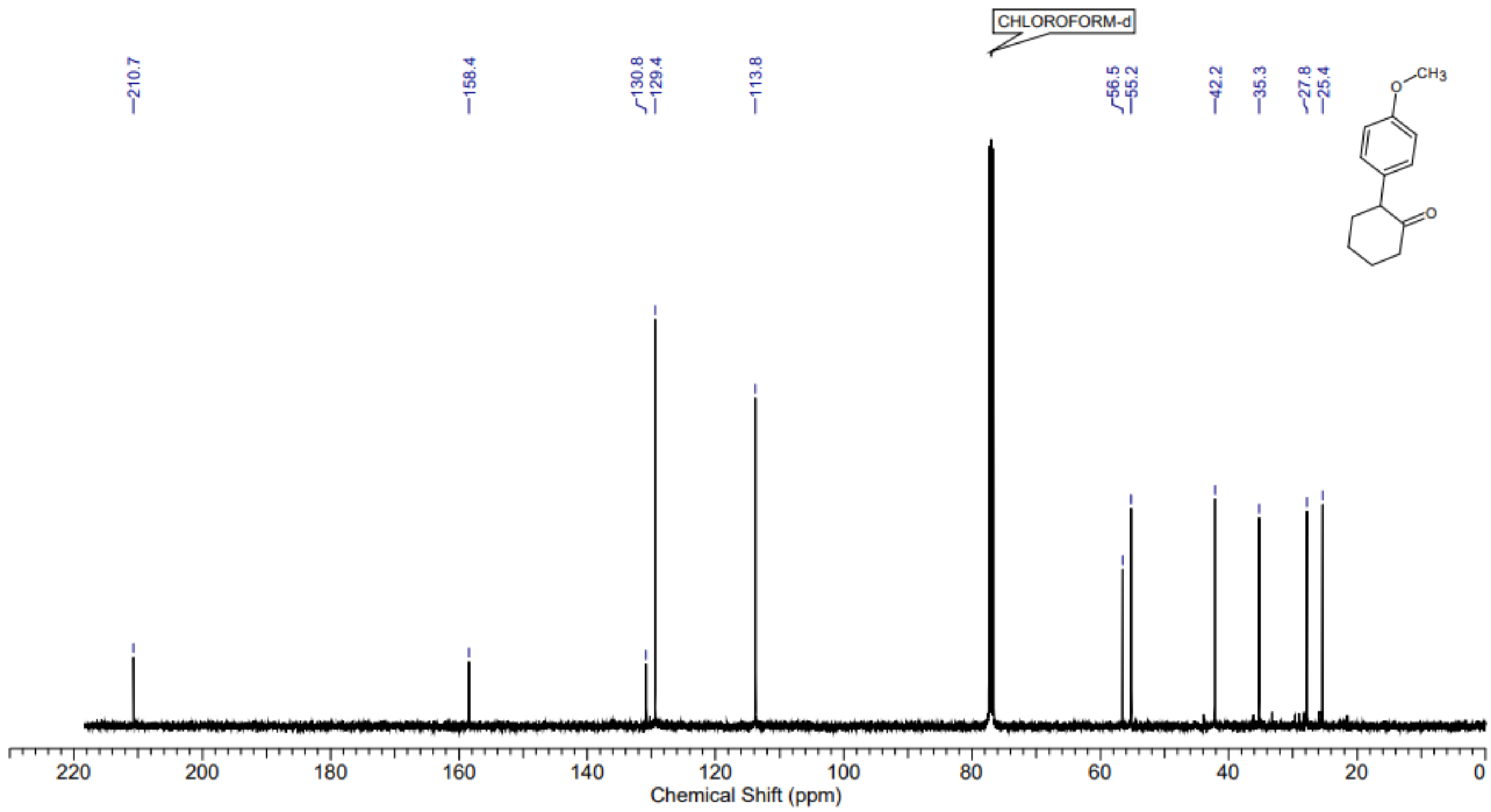

4-Methoxy-α-phenylbenzeneacetonitrile (**3ai**) [CAS: 4578-79-4]

^{1}H NMR (400 MHz, $CDCl_3$)

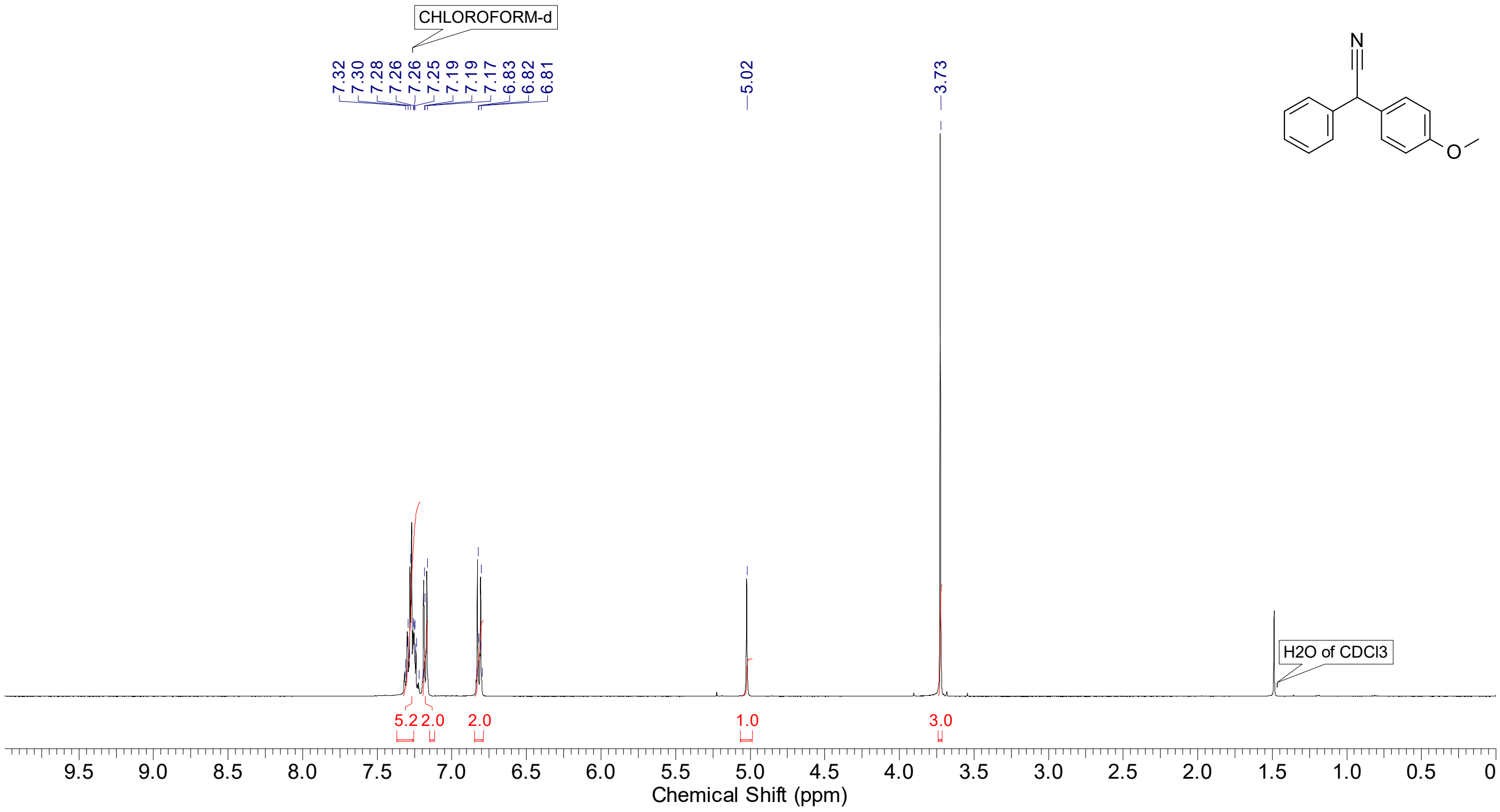

^{13}C NMR (101 MHz, $CDCl_3$)

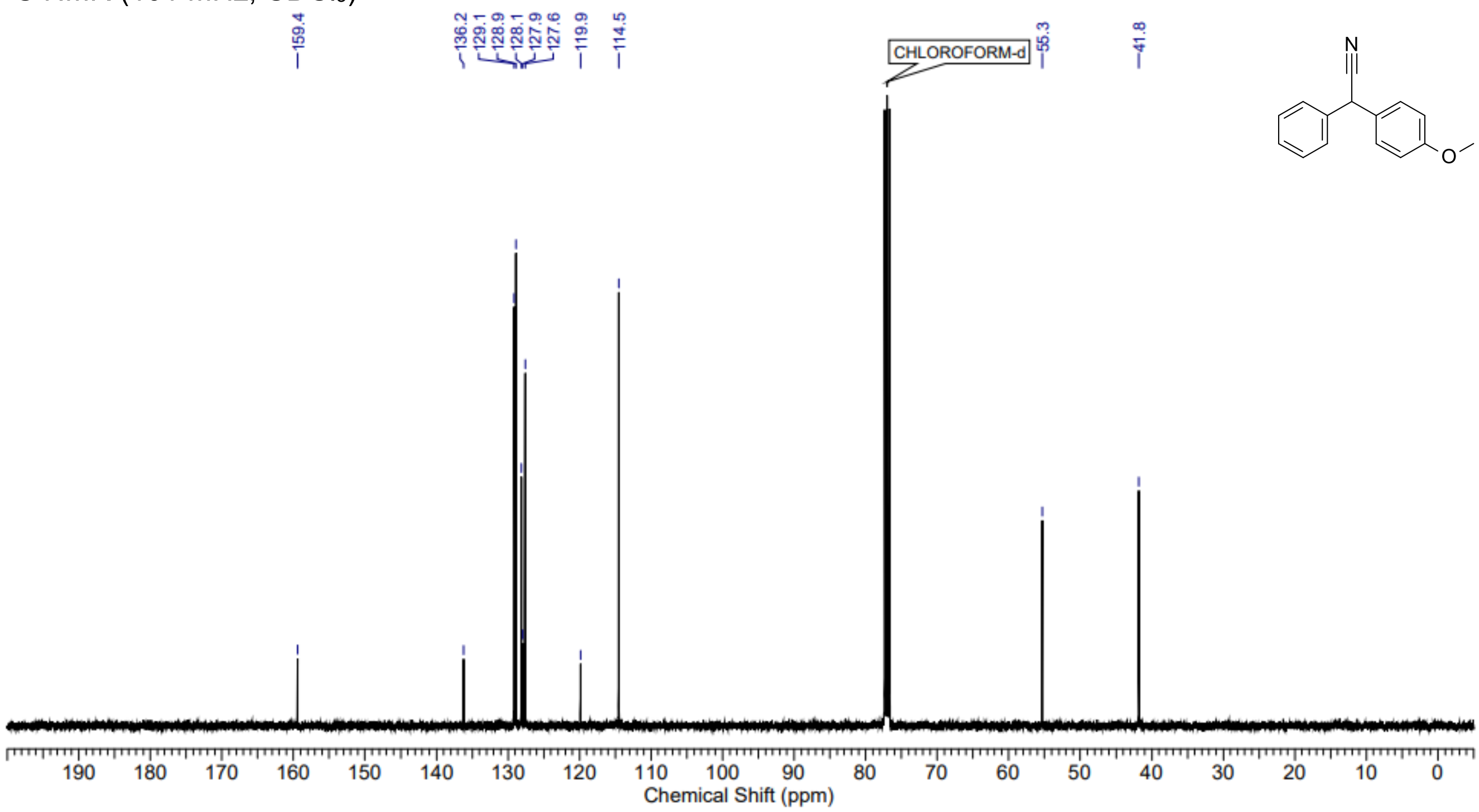

N,N-Diethyl-4-methoxybenzeneacetamide (**3aj**) [CAS: 115348-15-7]

^{1}H NMR (400 MHz, $CDCl_3$)

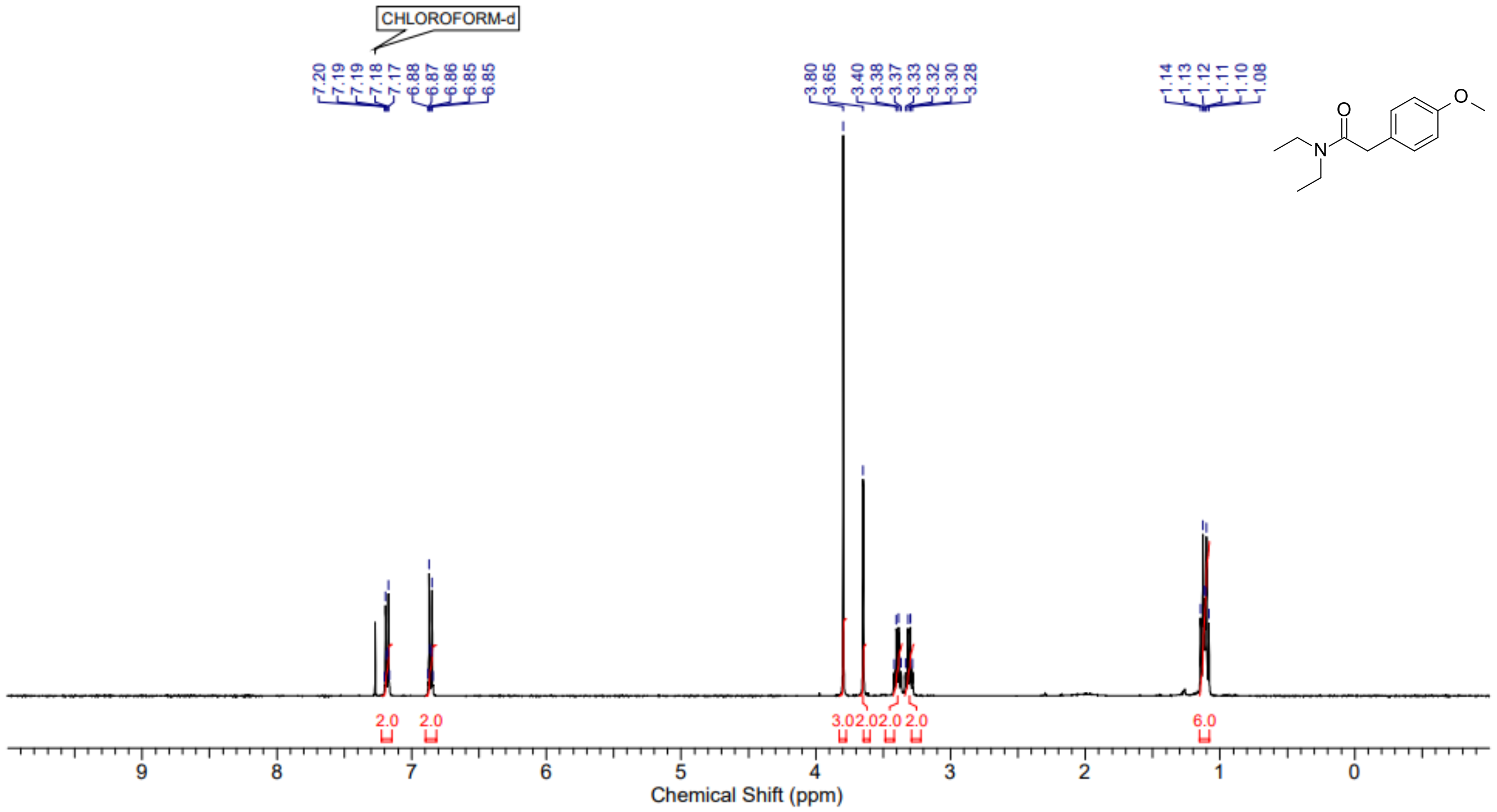

^{13}C NMR (101 MHz, $CDCl_3$)

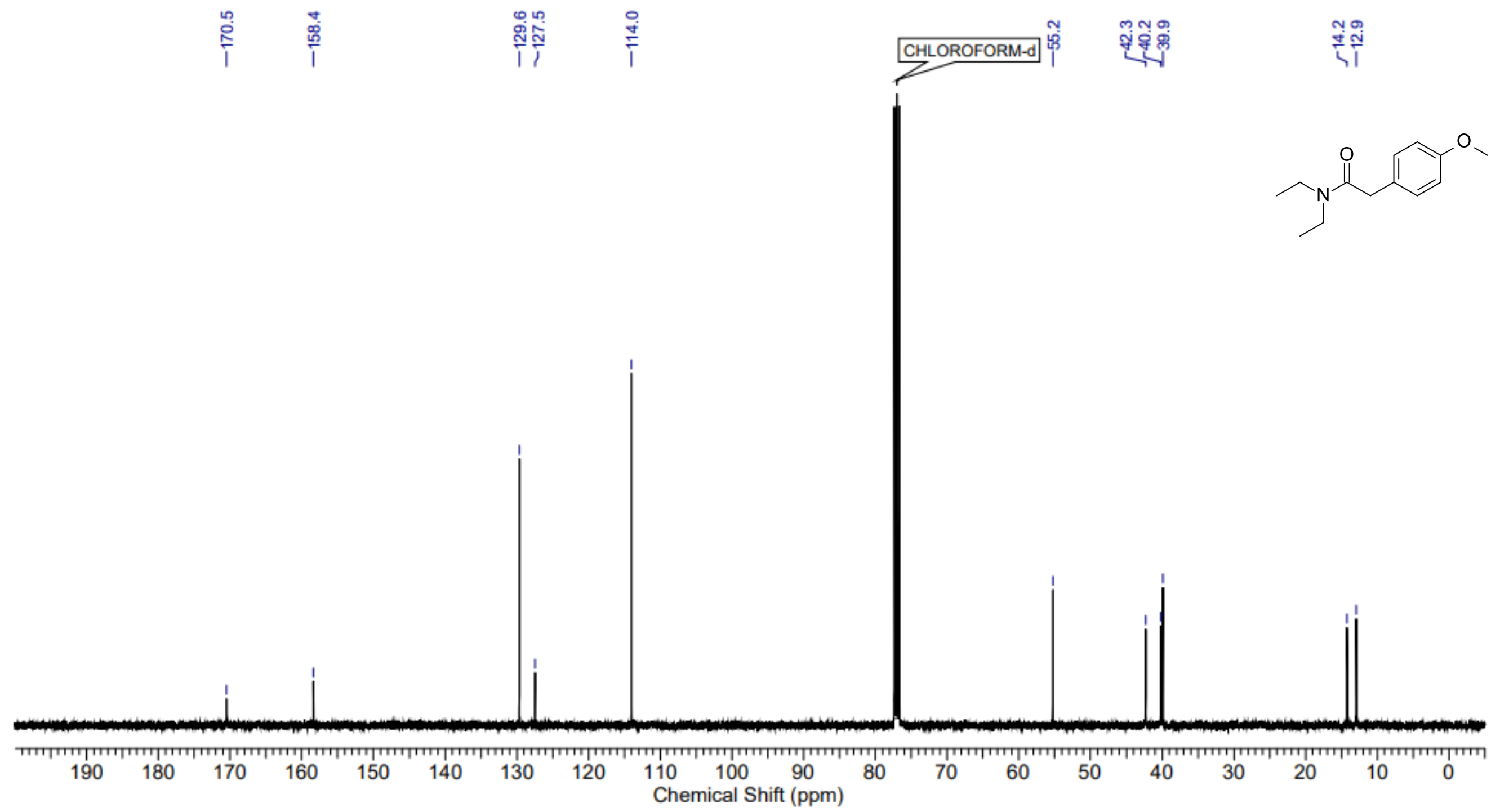

1,1-Dimethylethyl 4-methoxybenzeneacetate (**3ak**) [CAS: 33155-61-2]

^{1}H NMR (400 MHz, $CDCl_3$)

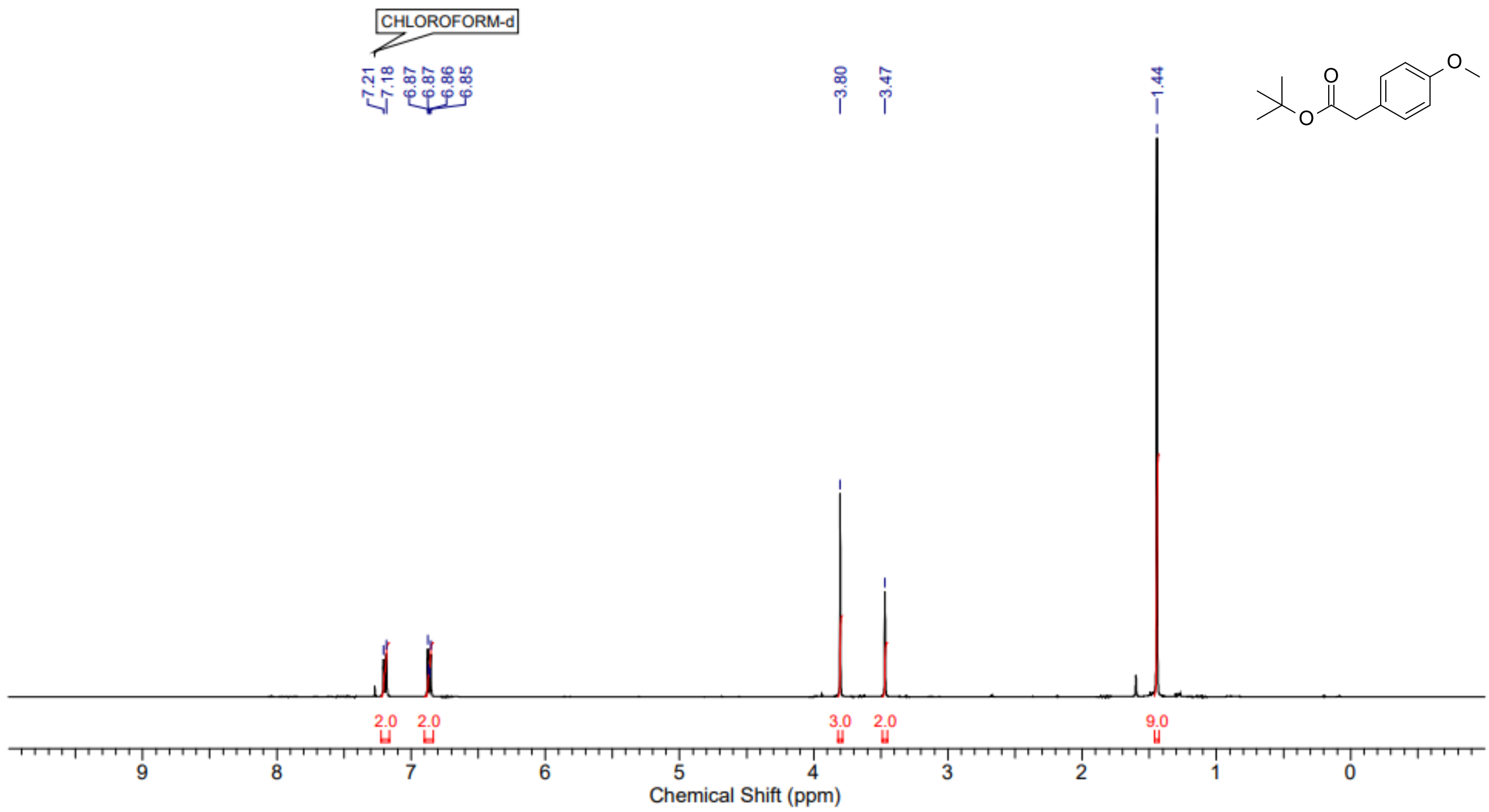

^{13}C NMR (101 MHz, $CDCl_3$)

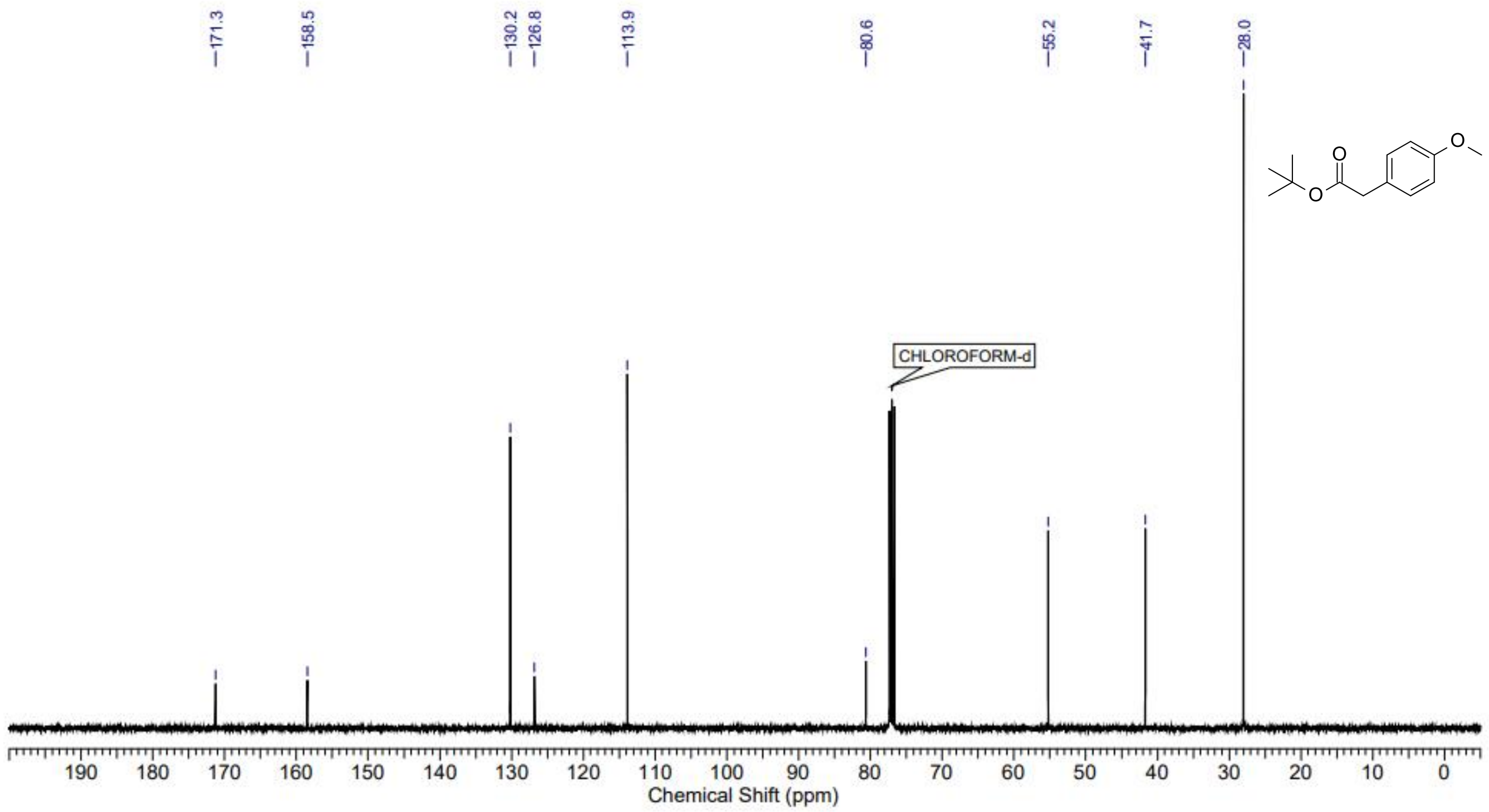

1-fluoro-4-[[(1R,2S,5R)-2-isopropyl-5-methyl-cyclohexoxy]methyl]benzene (**1s**) [CAS: 3060493-85-5]

^{1}H NMR (400 MHz, $CDCl_3$)

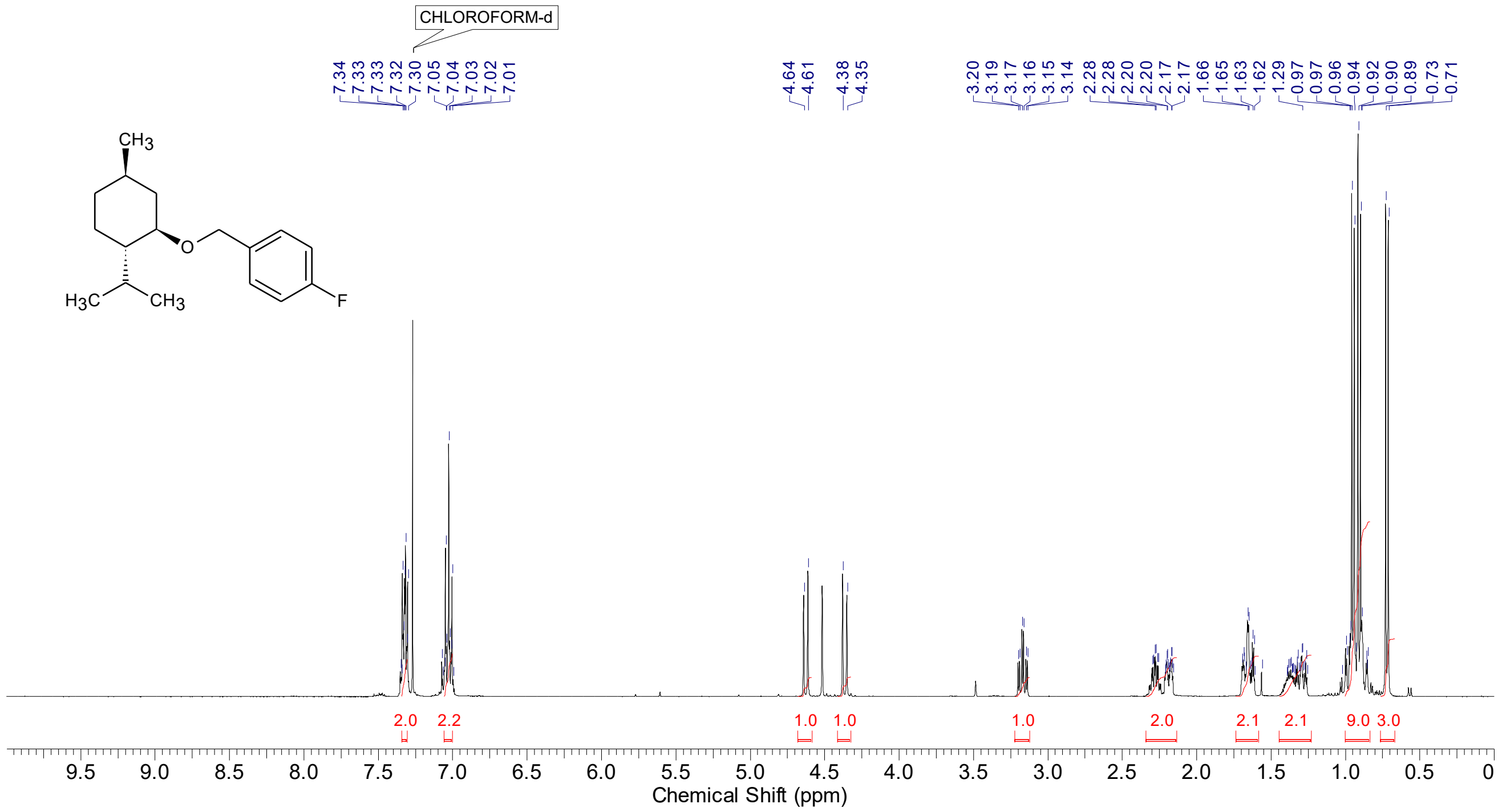

^{13}C NMR (101 MHz, $CDCl_3$)

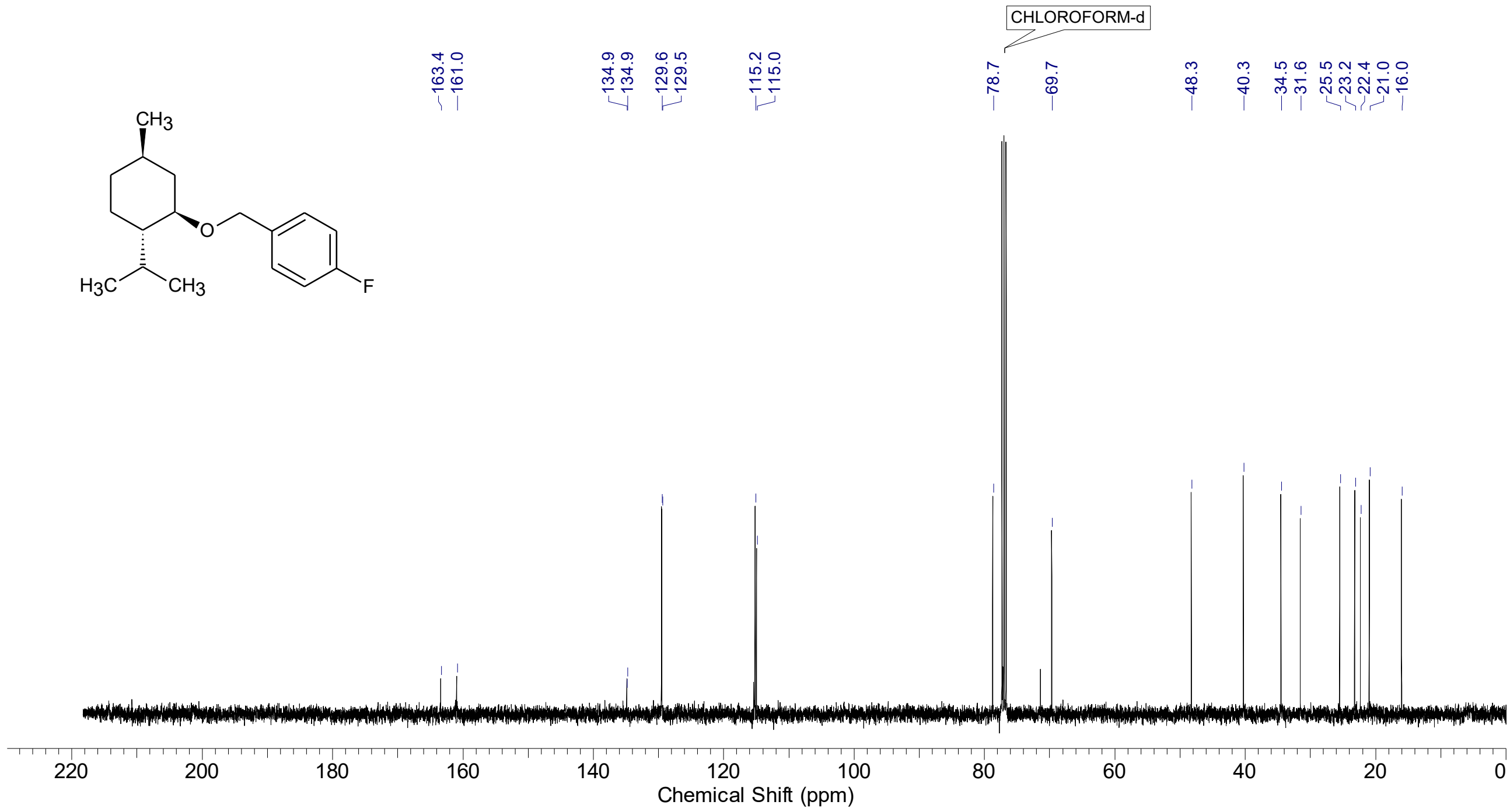

^{19}F NMR (377 MHz, $CDCl_3$)

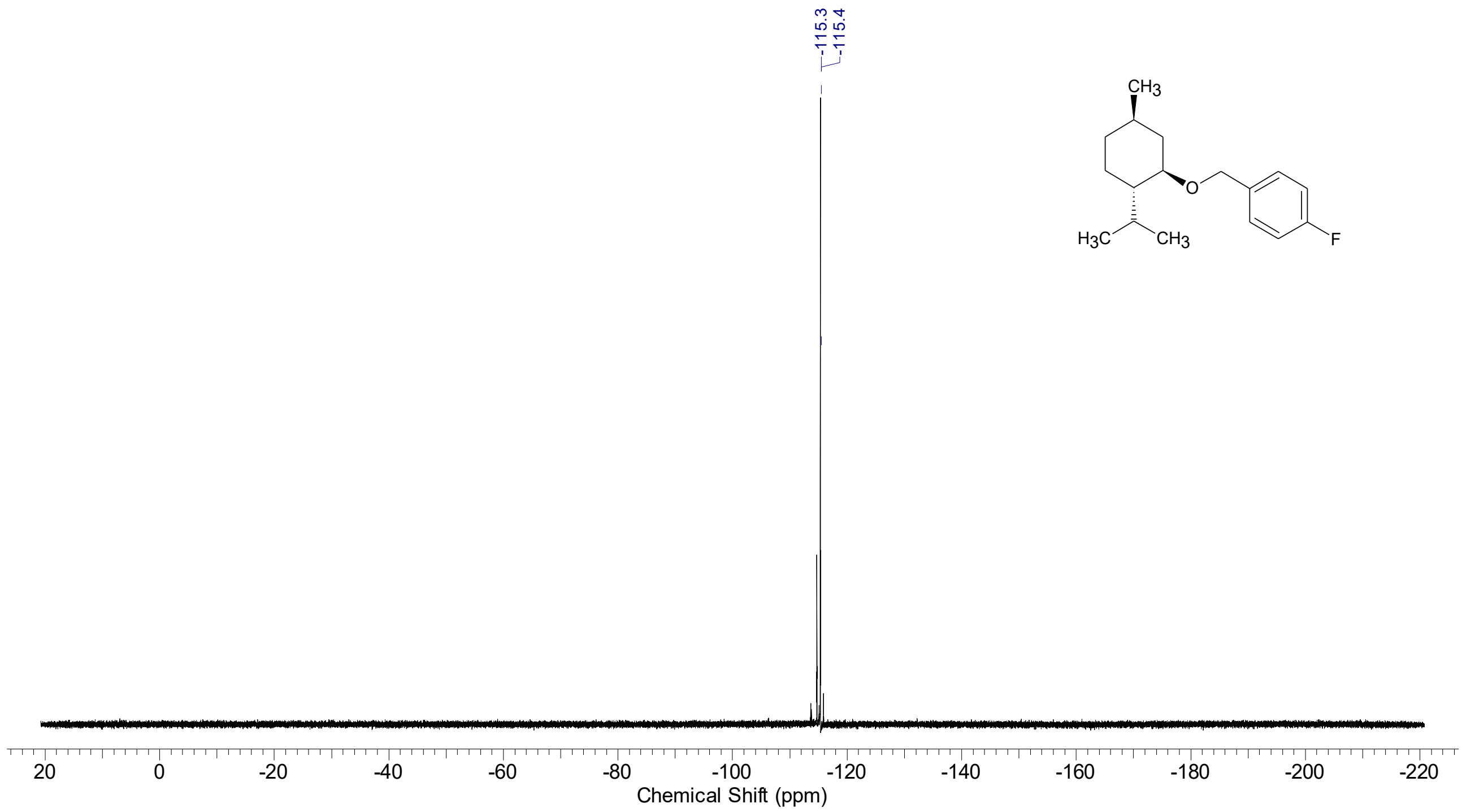

(3S,8S,9S,10R,13R,14S,17R)-17-[(1R)-1,5-dimethylhexyl]-3-[(4-fluorophenyl)methoxy]-10,13-dimethyl-2,3,4,7,8,9,11,12,14,15,16,17-dodecahydro-1H-cyclopenta[a]phenanthrene (**1t**) [CAS: 3060493-87-7]

^{1}H NMR (400 MHz, $CDCl_3$)

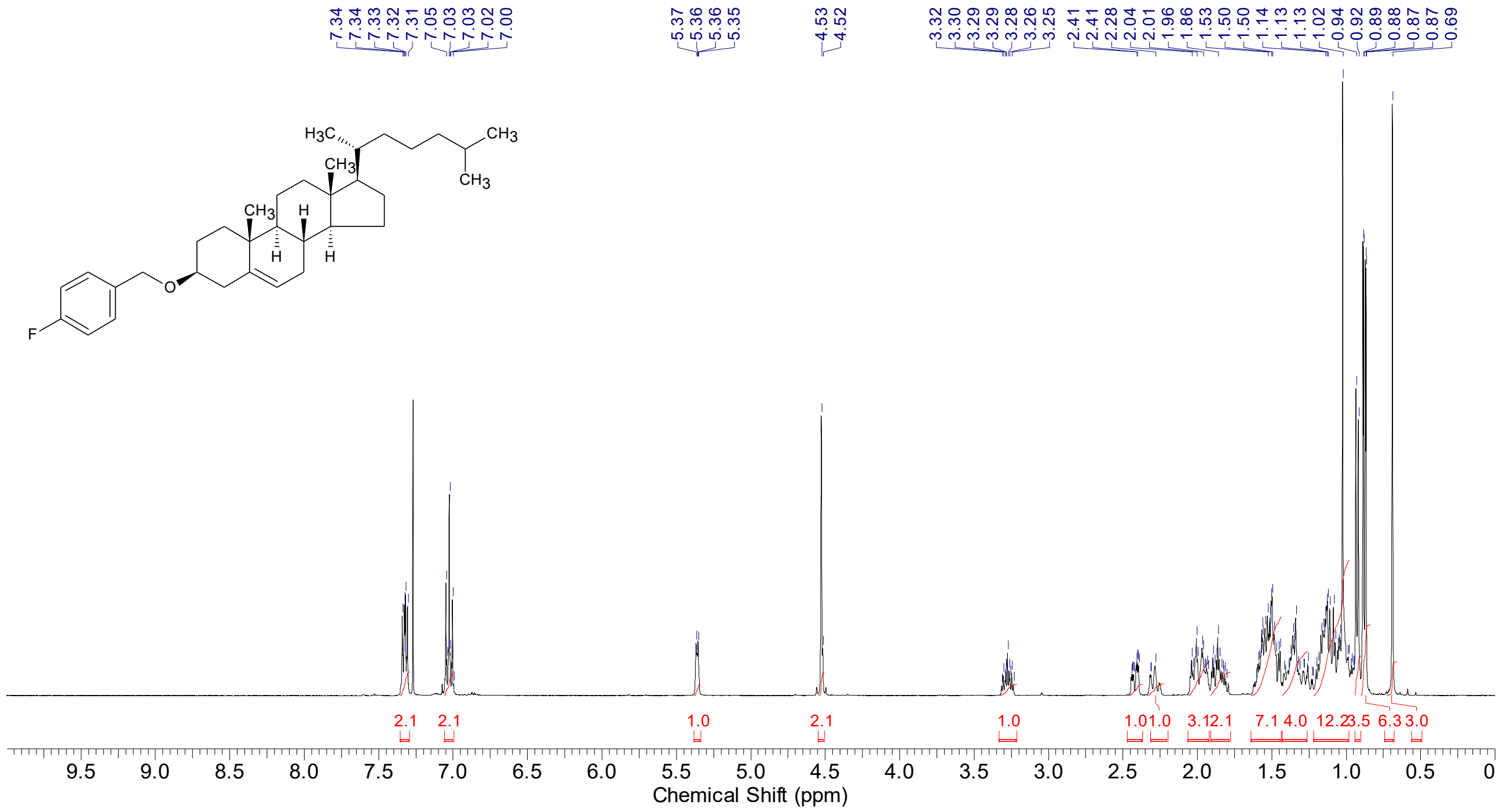

^{13}C NMR (101 MHz, $CDCl_3$)

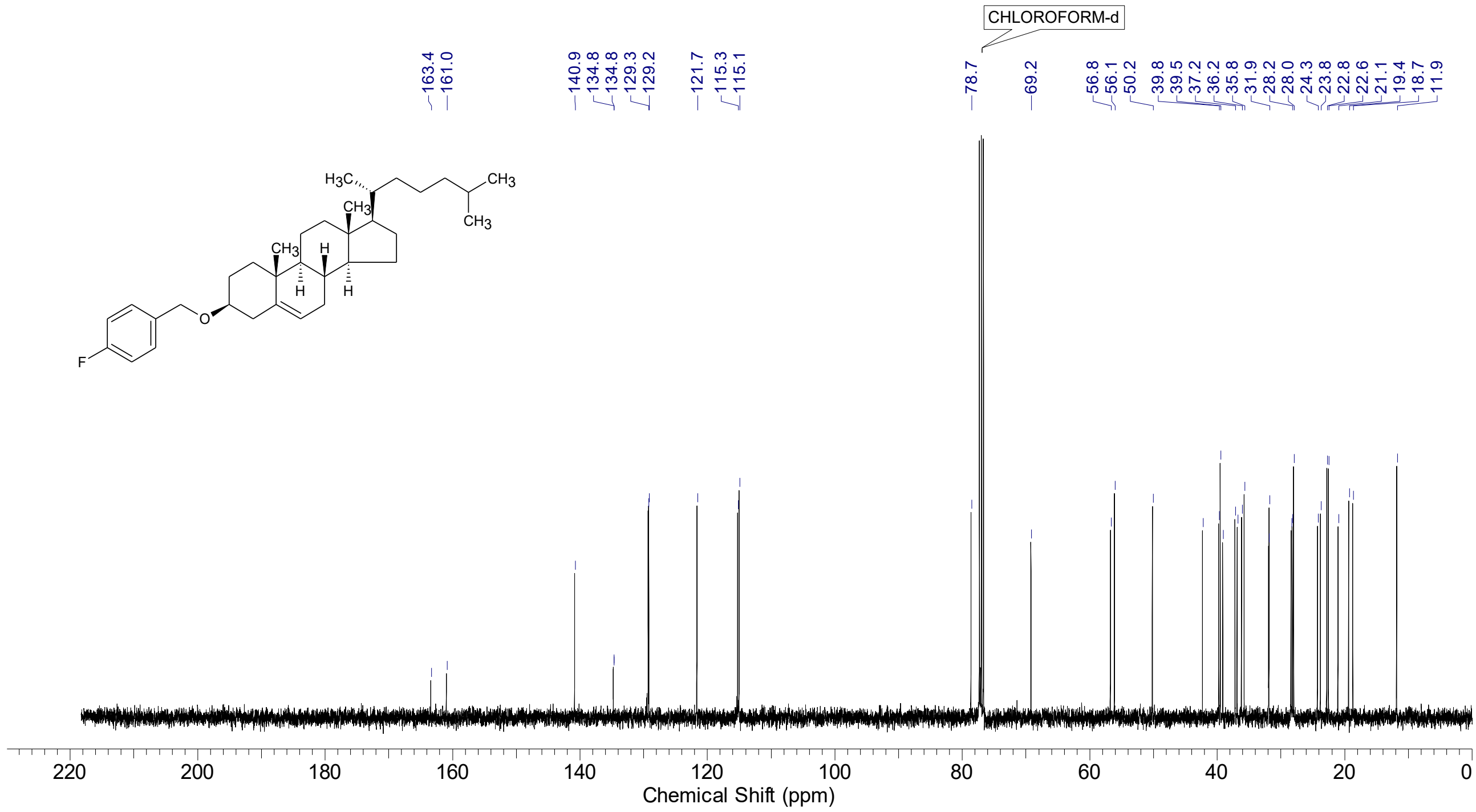

^{19}F NMR (377 MHz, $CDCl_3$)

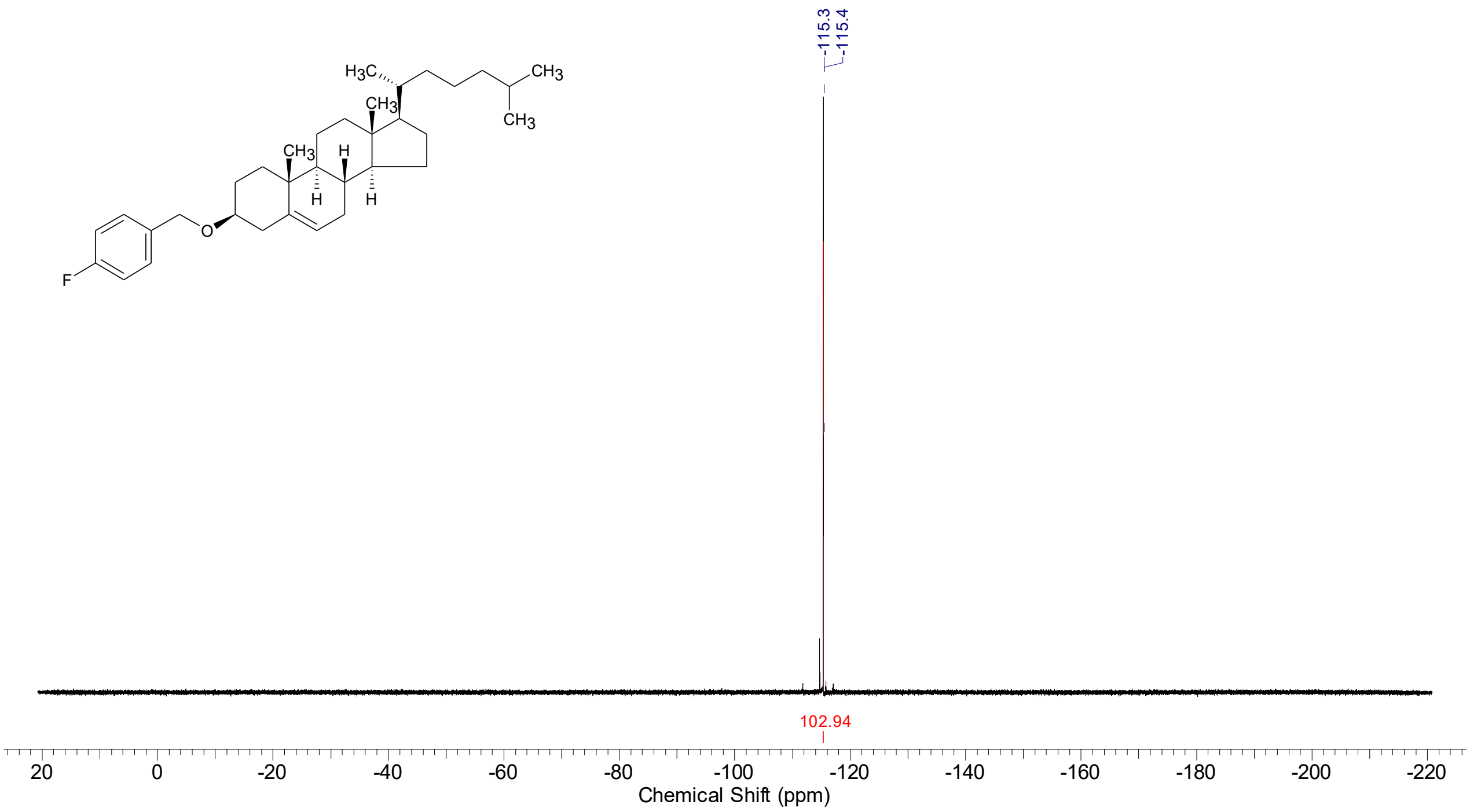

4-(4-fluorophenyl)morpholine (**1v**) [CAS: 4280-40-4]

^{1}H NMR (400 MHz, $CDCl_3$)

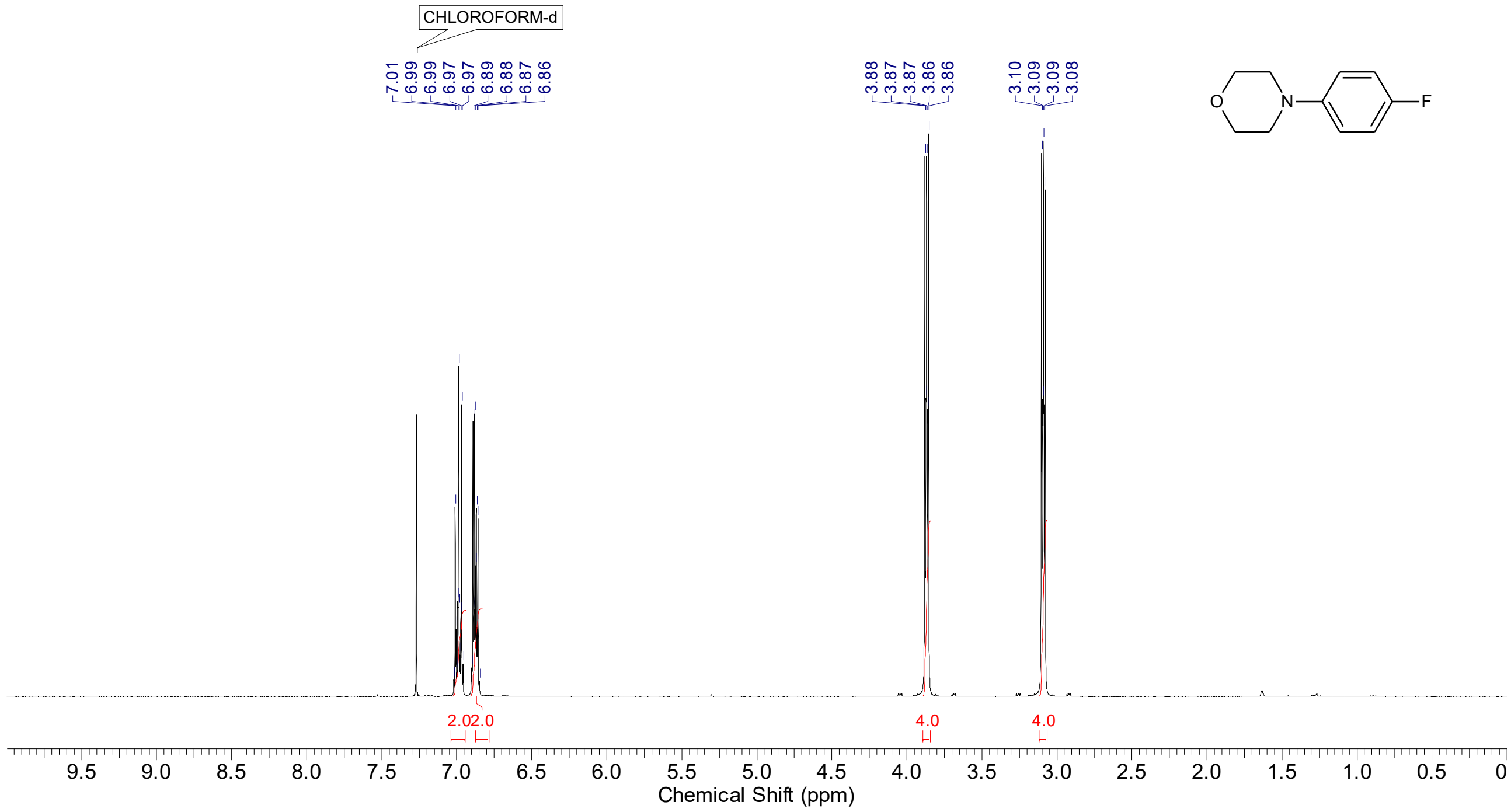

^{13}C NMR (101 MHz, $CDCl_3$)

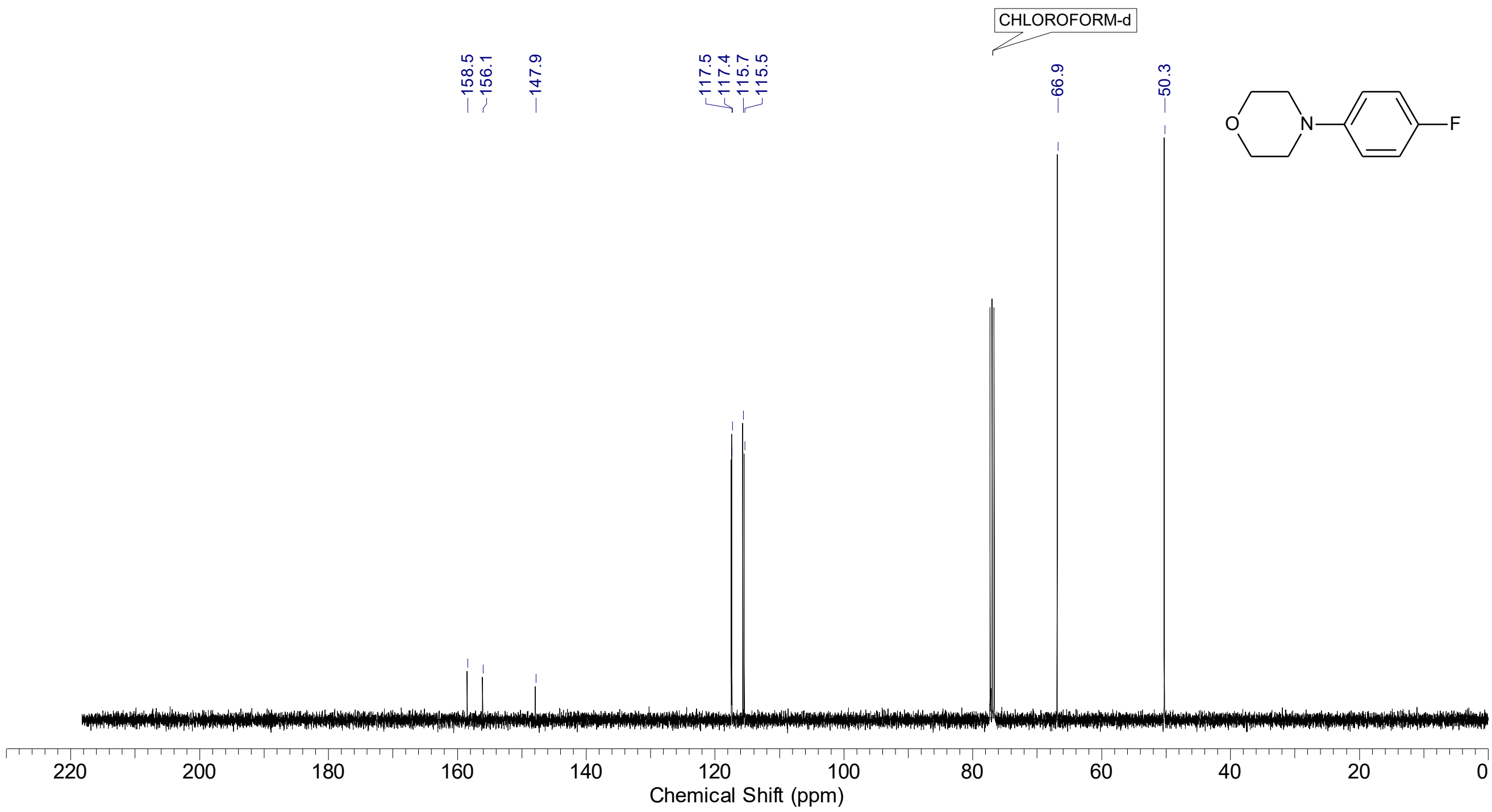

^{19}F NMR (377 MHz, $CDCl_3$)

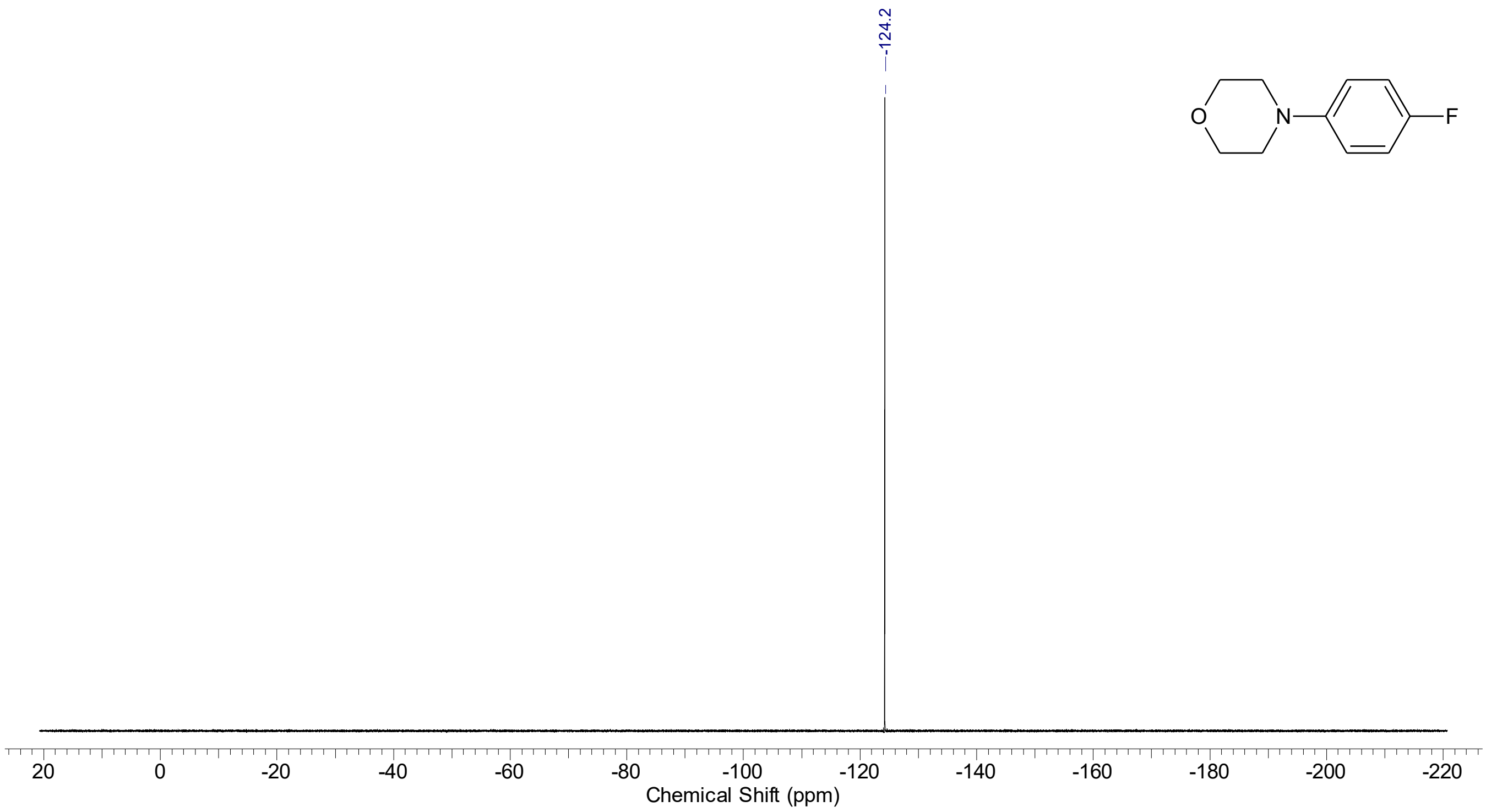

1-fluoro-4-phenyl-benzene (**1w**) [CAS: 324-74-3]

^{1}H NMR (400 MHz, $CDCl_3$)

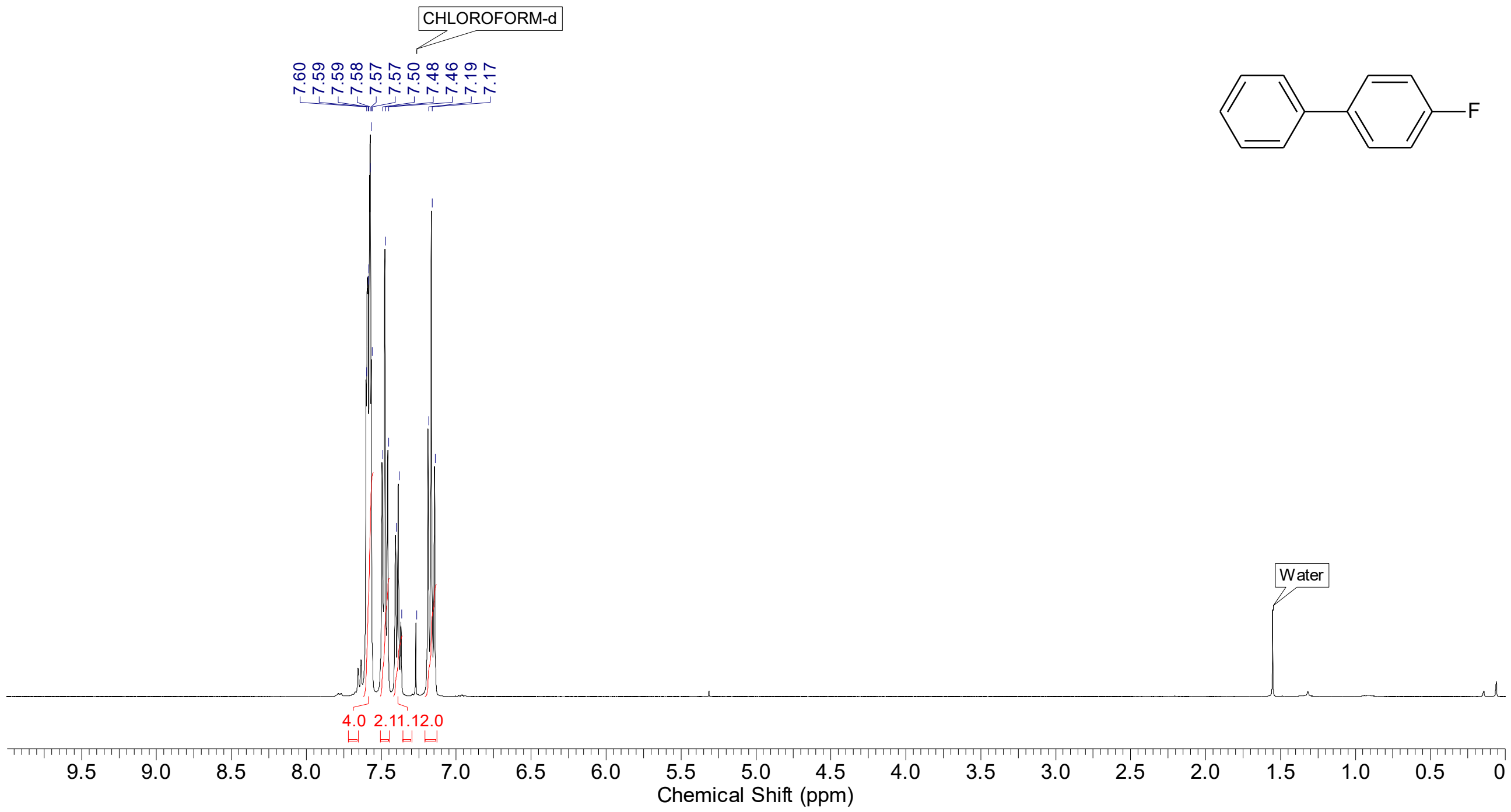

^{13}C NMR (101 MHz, $CDCl_3$)

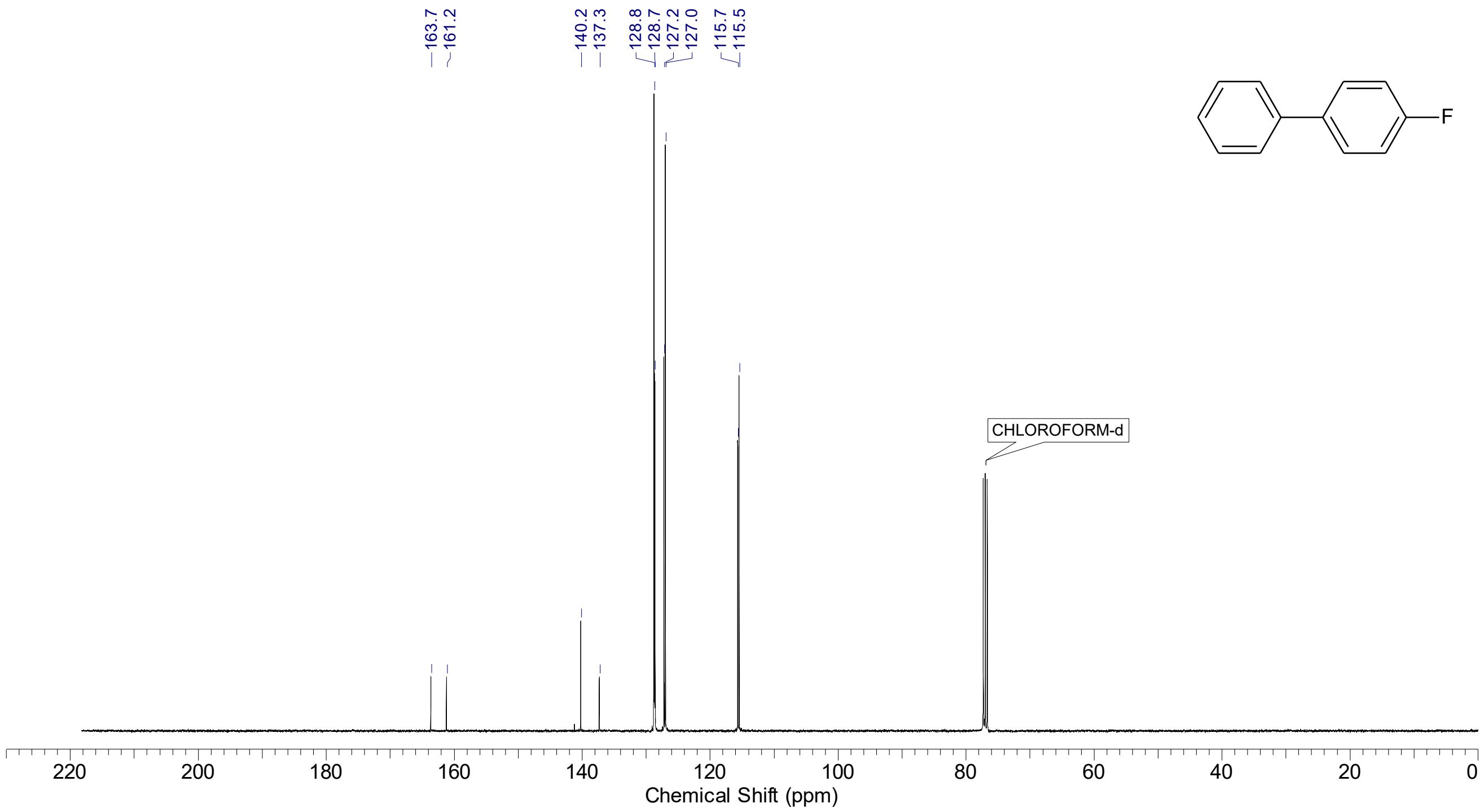

^{19}F NMR (76 MHz, $CDCl_3$)

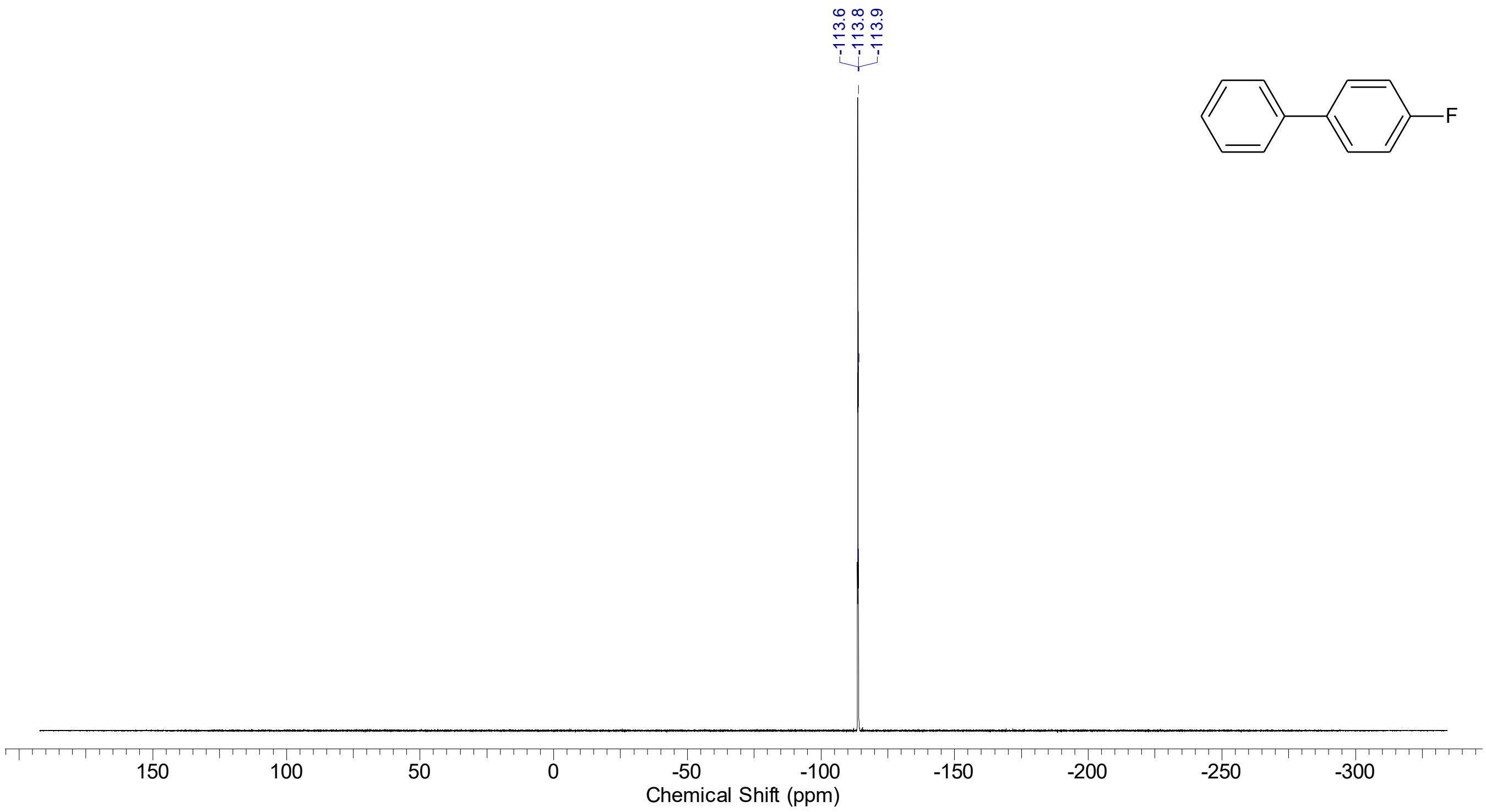

[Pd(1-MeNAP)TFA]$_2$

^{1}H NMR (400 MHz, DMSO-d6)

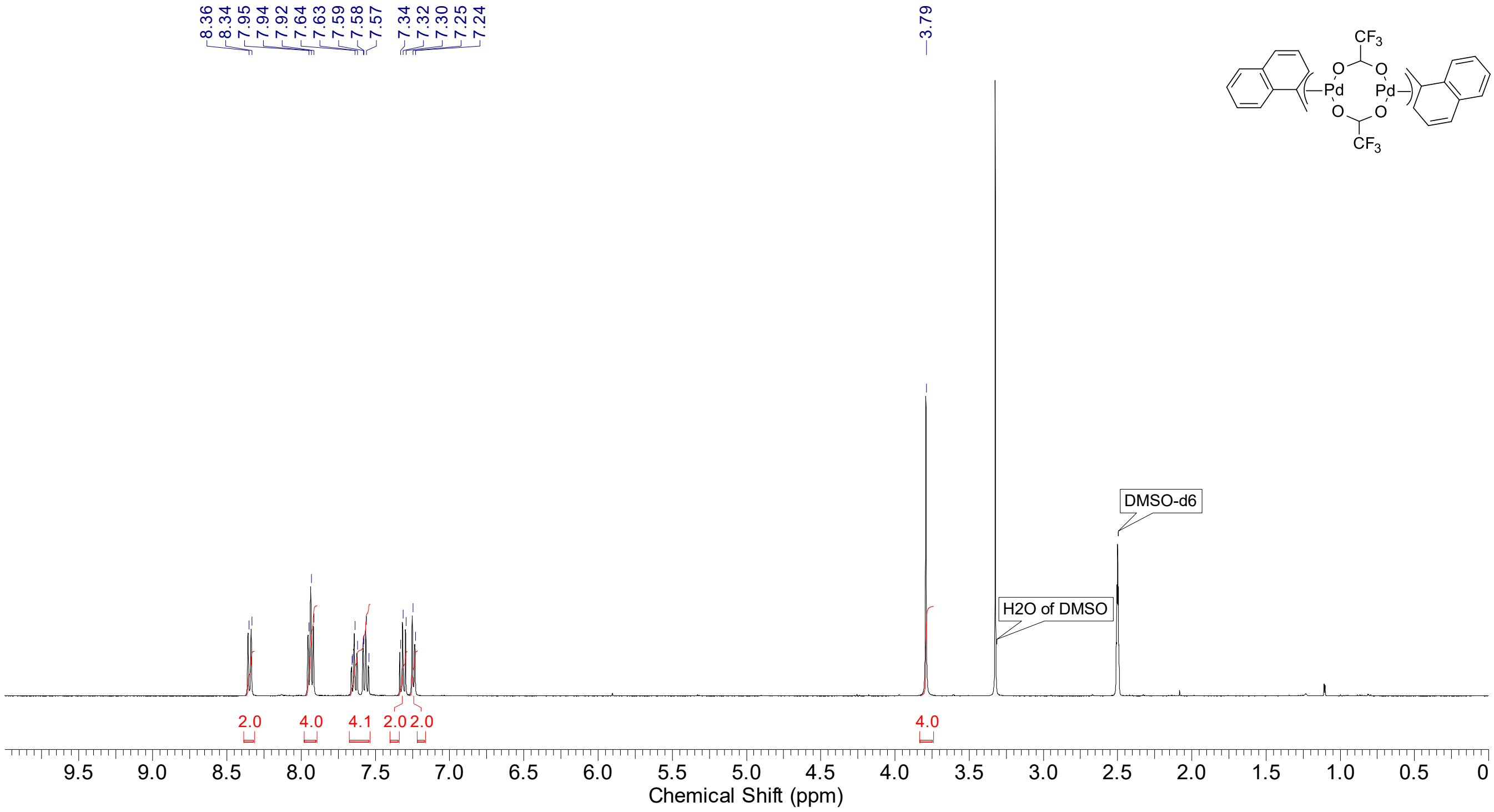

^{13}C NMR (101 MHz, DMSO-d6)

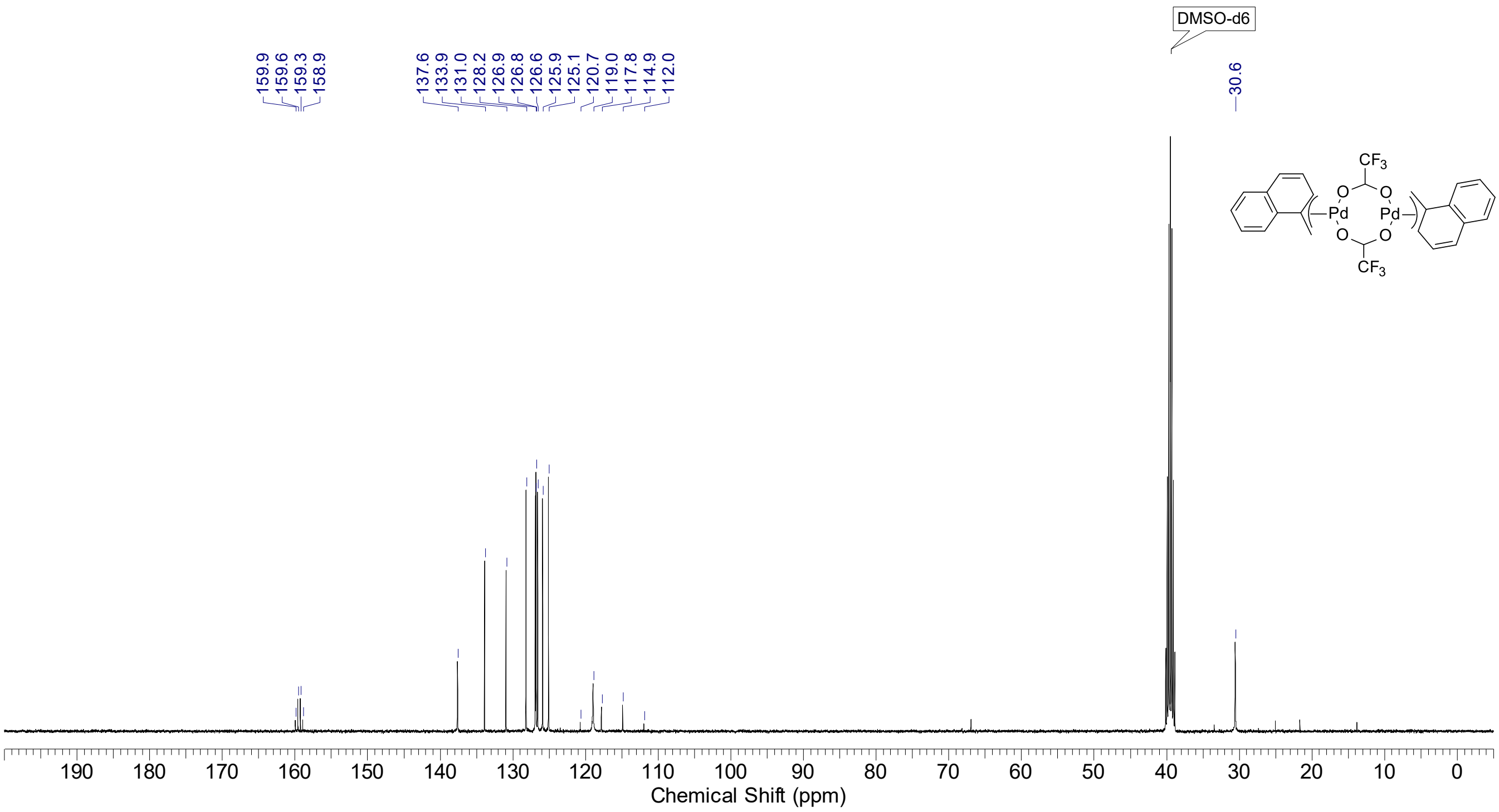

^{19}F NMR (76 MHz, DMSO-d6)

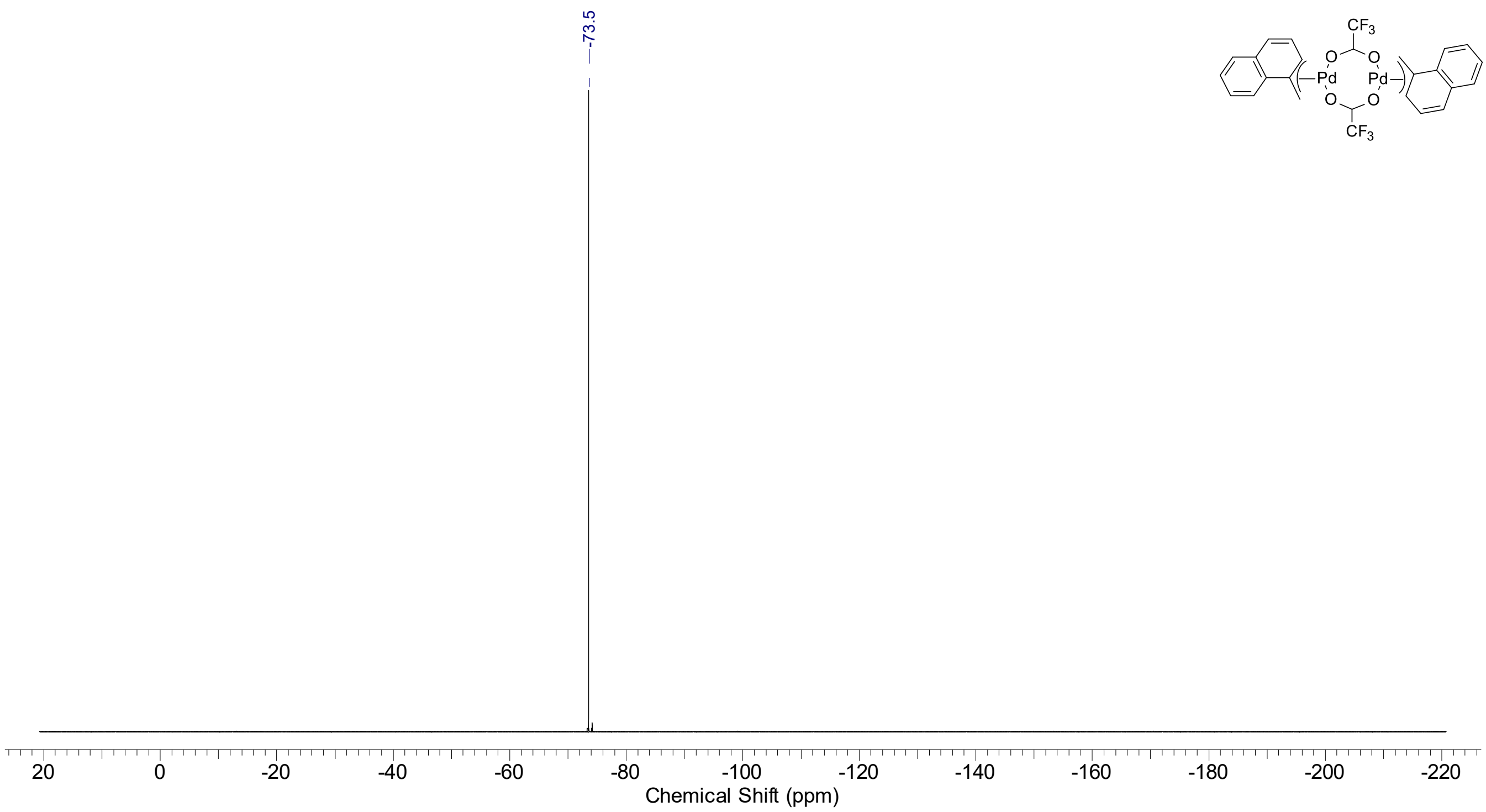

References

[1] W. L. F. Armarego, D. D. Perrin, "Purification of Laboratory Chemicals" *Oxford* **1998**.

[2] J. Zhou, B. Jiang, Y. Fujihira, Z. Zhao, T. Imai, N. Shibata, "Catalyst-free carbosilylation of alkenes using silyl boronates and organic fluorides via selective C-F bond activation" *Nat. Commun.* **2021**, *12*, 3749.

[3] N. Sivendran, N. Pirkl, Z. Hu, A. Doppiu, L. J. Gooßen, "Halogen-Bridged Methylnaphthyl Palladium Dimers as Versatile Catalyst Precursors in Coupling Reactions" *Angew. Chem. Int. Ed.* **2021**, *60*, 25151–25160.

[4] F. Neese, "Software Update: The ORCA Program System—Version 6.0" *WIREs Comput. Mol. Sci.* **2025**, *15*, e70019.

[5] S. Lehtola, C. Steigemann, M. J. T. Oliveira, M. A. L. Marques, "Recent developments in libxc — A comprehensive library of functionals for density functional theory" *SoftwareX* **2018**, *7*, 1–5.

[6] V. Ásgeirsson, B. O. Birgisson, R. Bjornsson, U. Becker, F. Neese, C. Riplinger, H. Jónsson, "Nudged Elastic Band Method for Molecular Reactions Using Energy-Weighted Springs Combined with Eigenvector Following" *J. Chem. Theory Comput.* **2021**, *17*, 4929–4945.

[7] F. Weigend, R. Ahlrichs, "Balanced basis sets of split valence, triple zeta valence and quadruple zeta valence quality for H to Rn: Design and assessment of accuracy" *Phys. Chem. Chem. Phys.* **2005**, *7*, 3297–3305.

[8] E. Caldeweyher, S. Ehlert, A. Hansen, H. Neugebauer, S. Spicher, C. Bannwarth, S. Grimme, "A generally applicable atomic-charge dependent London dispersion correction" *J. Chem. Phys.* **2019**, *150*, 154122.

[9] A. V. Marenich, C. J. Cramer, D. G. Truhlar, "Universal Solvation Model Based on Solute Electron Density and on a Continuum Model of the Solvent Defined by the Bulk Dielectric Constant and Atomic Surface Tensions" *J. Phys. Chem. B* **2009**, *113*, 6378–6396.

[10] X. Chen, Z. Chen, C. M. So, "Exploration of Aryl Phosphates in Palladium-Catalyzed Mono-α-arylation of Aryl and Heteroaryl Ketones" *J. Org. Chem.* **2019**, *84*, 6337–6346.

[11] B. Landers, C. Berini, C. Wang, O. Navarro, "(N-Heterocyclic Carbene)-Pd-Catalyzed Anaerobic Oxidation of Secondary Alcohols and Domino Oxidation−Arylation Reactions" *J. Org. Chem.* **2011**, *76*, 1390–1397.

[12] C. Cao, L. Wang, Z. Cai, L. Zhang, J. Guo, G. Pang, Y. Shi, "Palladium-Catalyzed α-Ketone Arylation under Mild Conditions" *Eur. J. Org. Chem.* **2011**, *2011*, 1570–1574.

[13] L. V. Desai, D. T. Ren, T. Rosner, "Mild and General Method for the α-Arylation of Heteroaromatic Ketones" *Org. Lett.* **2010**, *12*, 1032–1035.

[14] W. C. Fu, C. M. So, O. Y. Yuen, I. T. C. Lee, F. Y. Kwong, "Exploiting Aryl Mesylates and Tosylates in Catalytic Mono-α-arylation of Aryl- and Heteroarylketones" *Org. Lett.* **2016**, *18*, 1872–1875.

[15] M. S. Viciu, R. F. Germaneau, S. P. Nolan, "Well-Defined, Air-Stable (NHC)Pd(Allyl)Cl (NHC = N-Heterocyclic Carbene) Catalysts for the Arylation of Ketones" *Org. Lett.* **2002**, *4*, 4053–4056.

[16] L.-Z. Yuan, D. Renko, I. Khelifi, O. Provot, J.-D. Brion, A. Hamze, M. Alami, "Selective Metal-Free Deoxygenation of Unsymmetrical 1,2-Dicarbonyl Compounds by Chlorotrimethylsilane and Sodium Iodide" *Org. Lett.* **2016**, *18*, 3238–3241.

[17] F. Zeng, B. Ma, Y. Wang, H. Li, M. Zhang, Z. Li, "Titanium Catalyzed the Cross-Coupling of Nitriles and Alkyl Halides for the Synthesis of Ketones" *Adv. Synth. Catal.* **2025**, *367*, e202500222.

[18] K. Gao, H. Yorimitsu, A. Osuka, "α-Arylation of Ketimines with Aryl Sulfides at a Low Palladium Catalyst Loading" *Angew. Chem. Int. Ed.* **2016**, *55*, 4573–4576.

[19] R.-G. Tian, L. Wang, L. Zheng, S.-K. Tian, "Nickel-Catalyzed Inter- and Intramolecular Reductive Cross-Coupling Reactions of Arylbenzylammonium Salts through Highly Site-Selective C–N Bond Cleavage" *ACS Catal.* **2024**, *14*, 5039–5046.

[20] S. M. Crawford, P. G. Alsabeh, M. Stradiotto, “Palladium-Catalyzed Mono-α-arylation of Carbonyl-Containing Compounds with Aryl Halides using DalPhos Ligands” *Eur. J. Org. Chem.* **2012**, *2012*, 6042–6050.
[21] X. Zhou, P.-F. Zhong, Z. Li, P. Hu, J. Tu, C. Yang, L. Guo, W. Xia, T. Chen, “Photoelectrochemical Iron–Nickel Synergistic Catalysis for C(sp3)-H and Dehydroxymethylative Acylation” *Org. Lett.* **2025**, *27*, 6995–7000.
[22] C. He, S. Guo, L. Huang, A. Lei, “Copper Catalyzed Arylation/C−C Bond Activation: An Approach toward α-Aryl Ketones” *J. Am. Chem. Soc.* **2010**, *132*, 8273–8275.
[23] I. MacLean, D. J. Grenda, E. Echávarri, S. Muth, P. Nuernberger, L. Marzo, “Photochemical Insights on Acyl Azolium Salts Enable the Design of a Tandem Hydrogen Atom Transfer/Halogen Atom Transfer Acylation of Alkyl Bromides and Chlorides” *J. Am. Chem. Soc.* **2025**, *147*, 31324–31331.
[24] I. Collins, C. Moyes, W. B. Davey, M. Rowley, F. A. Bromidge, K. Quirk, J. R. Atack, R. M. McKernan, S.-A. Thompson, K. Wafford, G. R. Dawson, A. Pike, B. Sohal, N. N. Tsou, R. G. Ball, J. L. Castro, “3-Heteroaryl-2-pyridones: Benzodiazepine Site Ligands with Functional Selectivity for α2/α3-Subtypes of Human GABAA Receptor-Ion Channels” *J. Med. Chem.* **2002**, *45*, 1887–1900.
[25] M. R. Biscoe, S. L. Buchwald, “Selective Monoarylation of Acetate Esters and Aryl Methyl Ketones Using Aryl Chlorides” *Org. Lett.* **2009**, *11*, 1773–1775.
[26] L. Ackermann, V. P. Mehta, “Palladium-Catalyzed Mono-α-Arylation of Acetone with Aryl Imidazolylsulfonates” *Chem. – Eur. J.* **2012**, *18*, 10230–10233.
[27] Q.-L. Wang, H. Huang, G. Mao, G.-J. Deng, “Bromine radical-enhanced HAT activity leading to stoichiometric couplings of methylarenes with acid chlorides” *Green Chem.* **2022**, *24*, 8324–8329.
[28] C. H. Cheon, O. Kanno, F. D. Toste, “Chiral Brønsted Acid from a Cationic Gold(I) Complex: Catalytic Enantioselective Protonation of Silyl Enol Ethers of Ketones” *J. Am. Chem. Soc.* **2011**, *133*, 13248–13251.
[29] M. Nambo, M. Yar, J. D. Smith, C. M. Crudden, “The Concise Synthesis of Unsymmetric Triarylacetonitriles via Pd-Catalyzed Sequential Arylation: A New Synthetic Approach to Tri- and Tetraarylmethanes” *Org. Lett.* **2015**, *17*, 50–53.
[30] T. Hama, D. A. Culkin, J. F. Hartwig, “Palladium-Catalyzed Intermolecular α-Arylation of Zinc Amide Enolates under Mild Conditions” *J. Am. Chem. Soc.* **2006**, *128*, 4976–4985.
[31] T. Hama, J. F. Hartwig, “α-Arylation of Esters Catalyzed by the Pd(I) Dimer {[P(t-Bu)3]PdBr}2” *Org. Lett.* **2008**, *10*, 1545–1548.
[32] M. Shigeno, K. Hayashi, O. Sasamoto, R. Hirasawa, T. Korenaga, S. Ishida, K. Nozawa-Kumada, Y. Kondo, “Catalytic Concerted SNAr Reactions of Fluoroarenes by an Organic Superbase” *J. Am. Chem. Soc.* **2024**, *146*, 32452–32462.
[33] D. W. Old, J. P. Wolfe, S. L. Buchwald, “A Highly Active Catalyst for Palladium-Catalyzed Cross-Coupling Reactions: Room-Temperature Suzuki Couplings and Amination of Unactivated Aryl Chlorides” *J. Am. Chem. Soc.* **1998**, *120*, 9722–9723.
[34] J.-H. Huang, L.-M. Yang, “Nickel-Catalyzed Amination of Aryl Phosphates through Cleaving Aryl C–O Bonds” *Org. Lett.* **2011**, *13*, 3750–3753.
[35] J. P. Wolfe, R. A. Singer, B. H. Yang, S. L. Buchwald, “Highly Active Palladium Catalysts for Suzuki Coupling Reactions” *J. Am. Chem. Soc.* **1999**, *121*, 9550–9561.
[36] T. Niwa, Y. Uetake, M. Isoda, T. Takimoto, M. Nakaoka, D. Hashizume, H. Sakurai, T. Hosoya, “Lewis acid-mediated Suzuki–Miyaura cross-coupling reaction” *Nat. Catal.* **2021**, *4*, 1080–1088.
[37] S. Manna, H. Janning, N. V. Tzouras, J. A. S. Sagayaraj, F. F. Mannighayil, A. Doppiu, L. J. Gooßen, “Unlocking Catalyst Activation as a Critical Bottleneck in Cross-Coupling Reactions through [Pd(1-MeNAP)TFA]$_2$ Precatalysts” *Angew. Chem. Int. Ed.* **2026**, DOI accepted.

Author Contributions

NKR and JSH supported by LJG, developed the concept and methodology for the Catalytic C–F Bond Activation at Room Temperature: Enolate Cross-Couplings Enabled by $[Pd(1\text{-}MeNAP)TFA]_2$ and LiHMDS. Reactions were carried out by NKR, JSH and SMA. Product isolation and characterization were performed by NKR, JSH and LON. NKR and JSH performed mechanistic investigations. NKR and JSH took care of the project administration. NKR and LJG performed the DFT calculations. LJG supervised the project. NKR and LJG wrote the manuscript draft, which was revised by all authors.
